# Supplementary figures and images for: Sensitivity to the visual field origin of natural image patches in human low-level visual cortex
Source: PeerJ. 2015 Jun 23;3:e1038. doi: 10.7717/peerj.1038 (PMC4485252; doi:10.7717/peerj.1038)

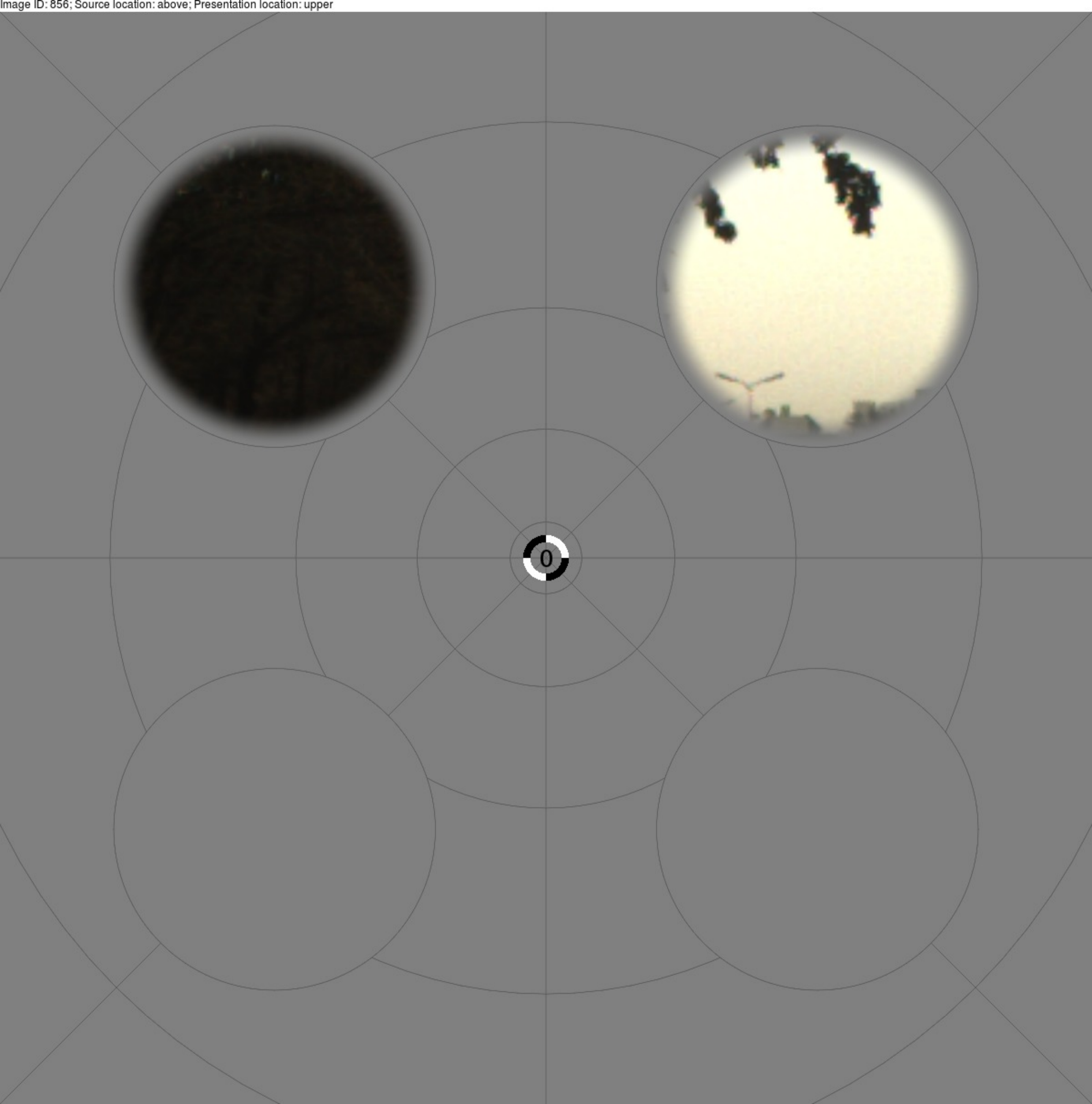

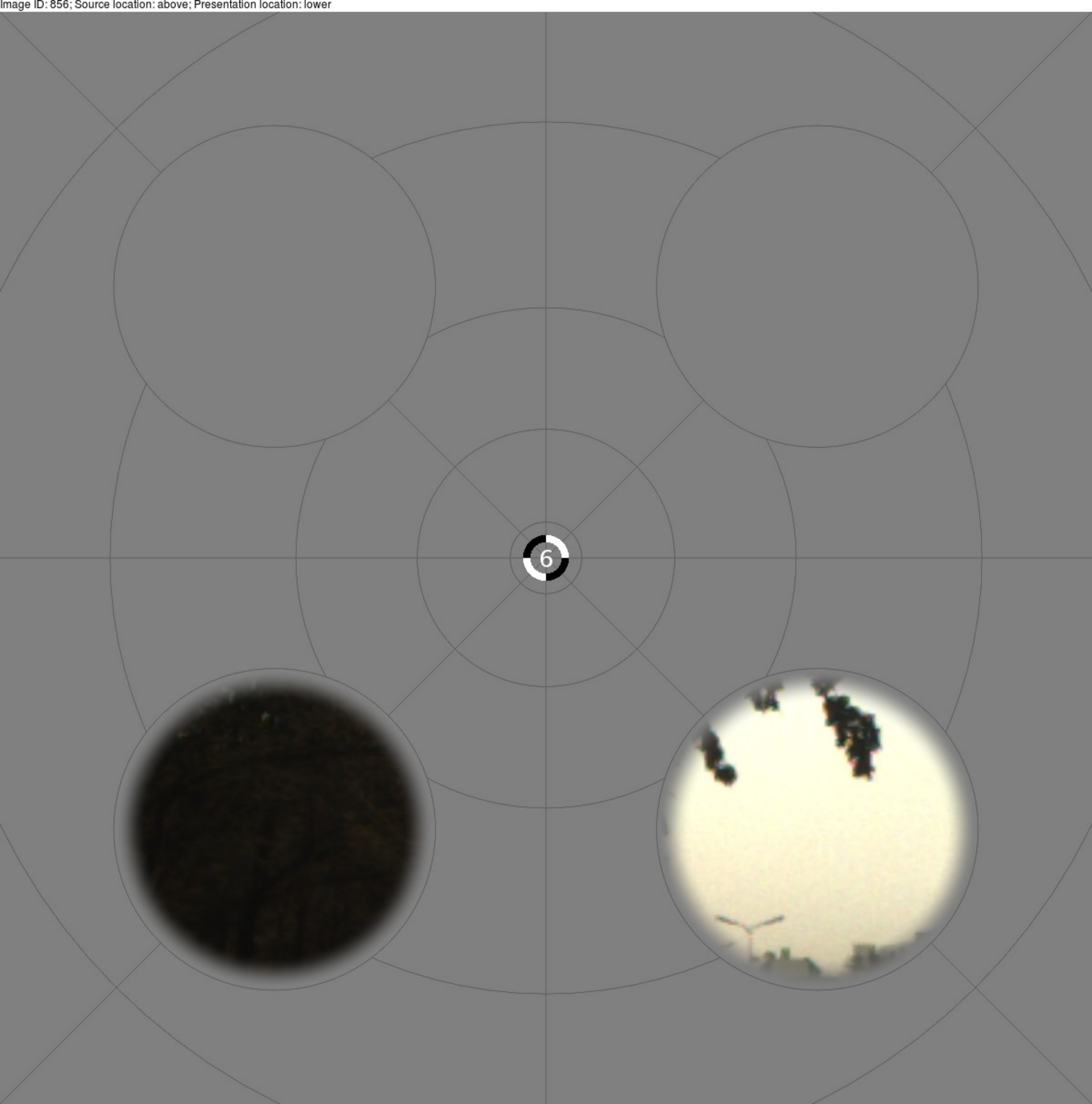

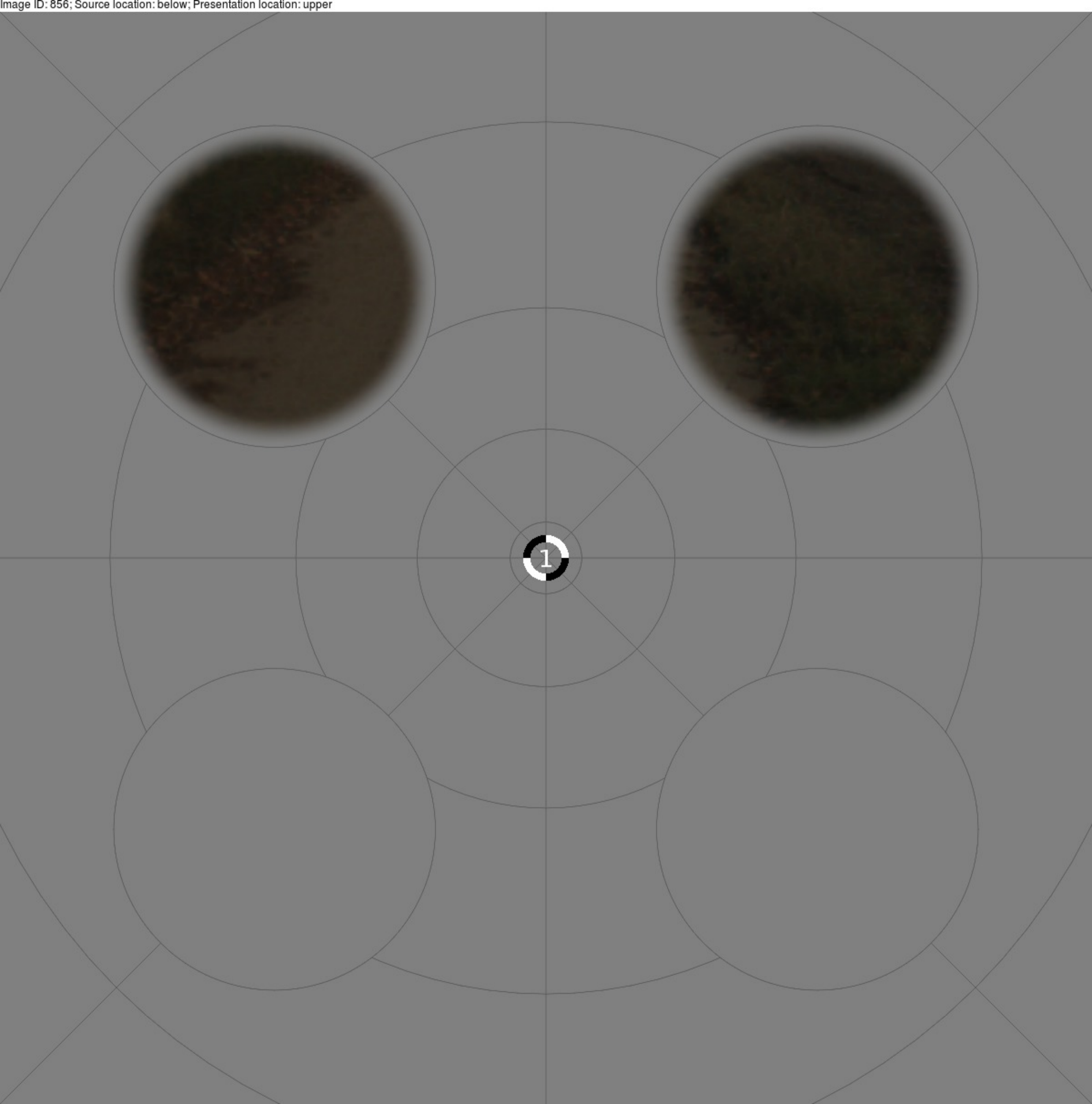

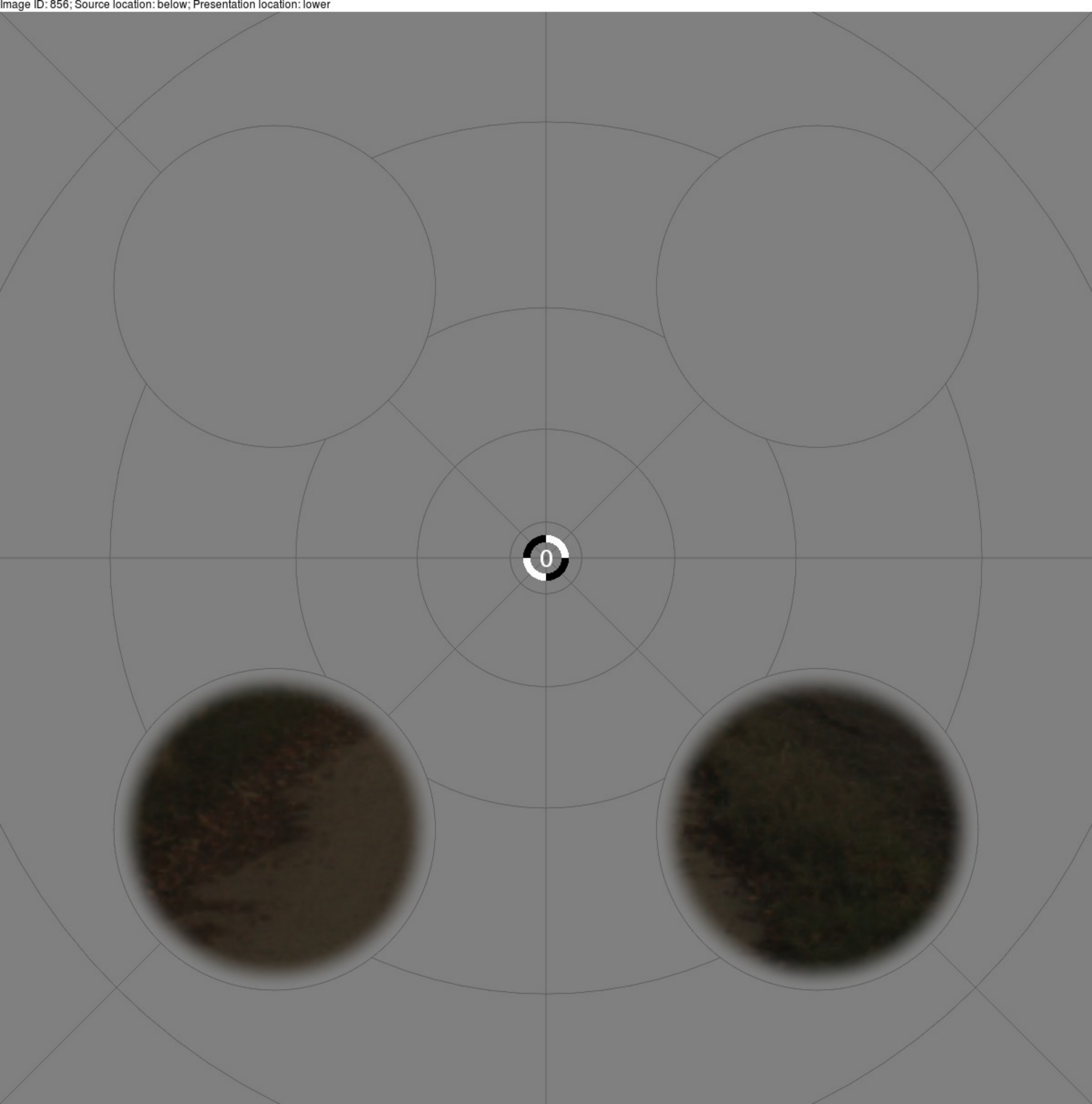

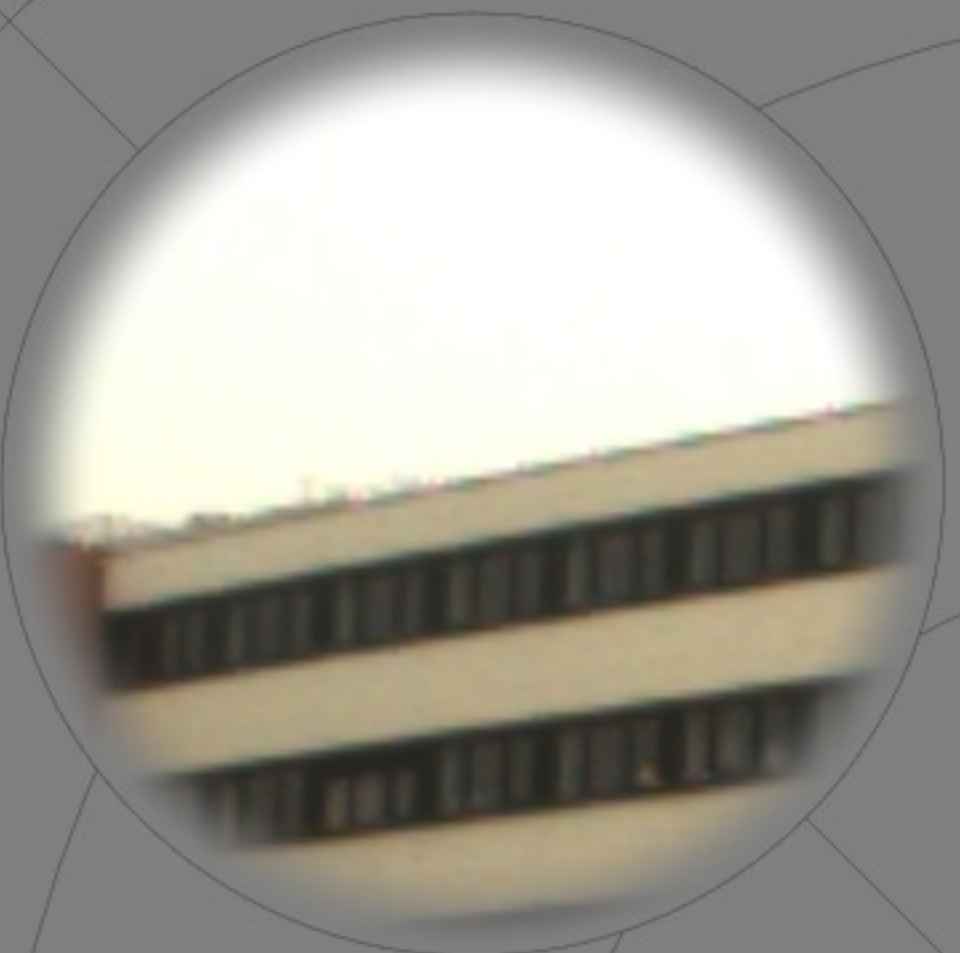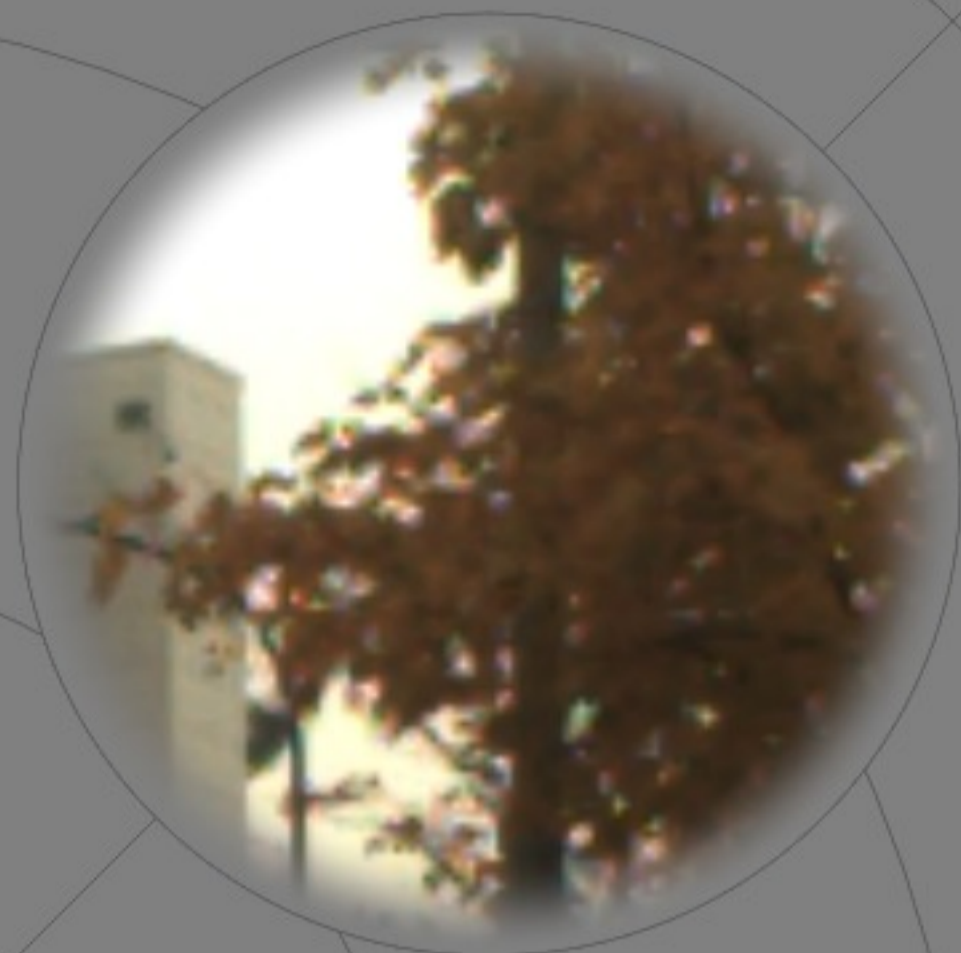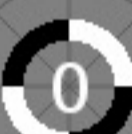

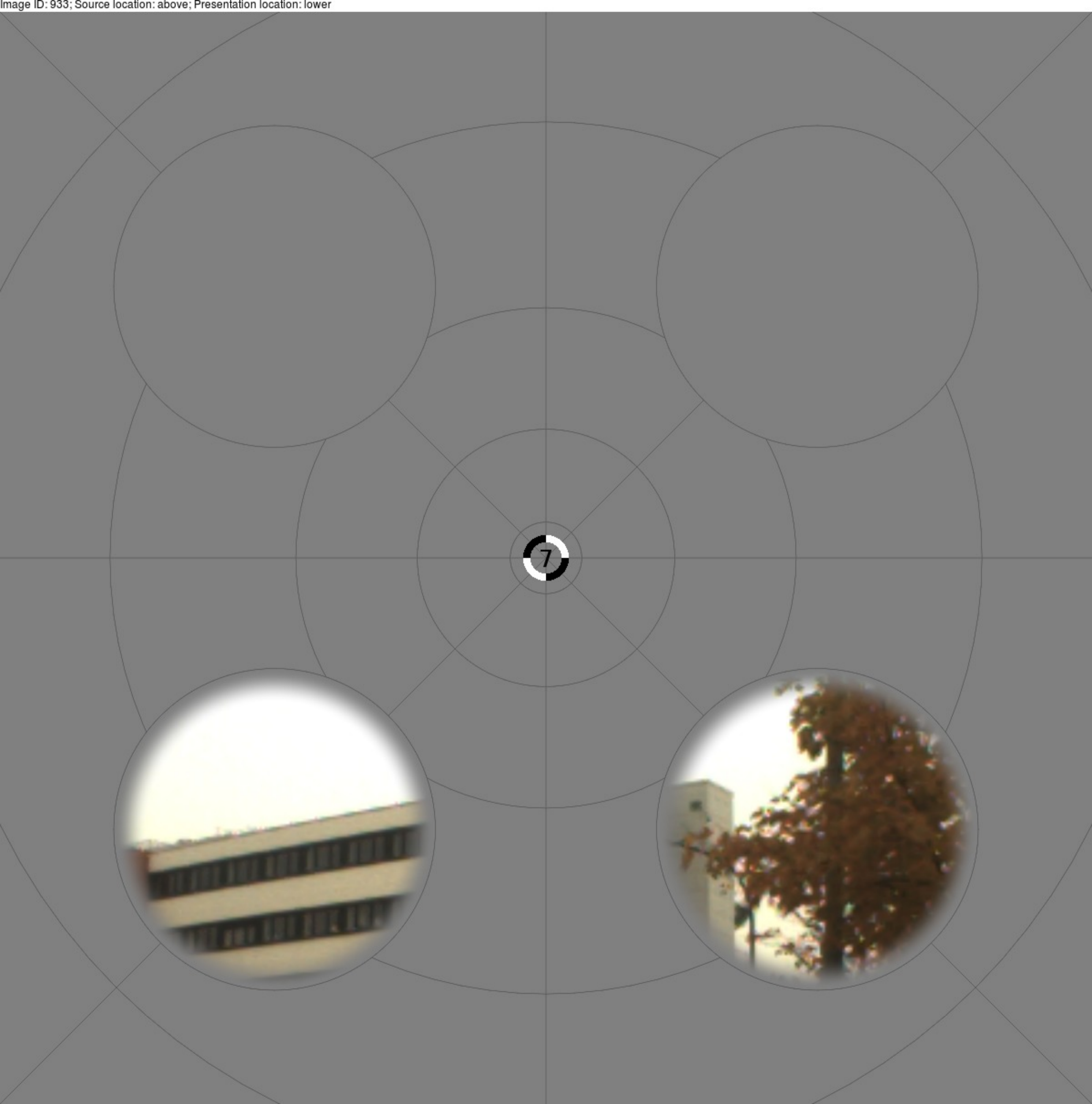

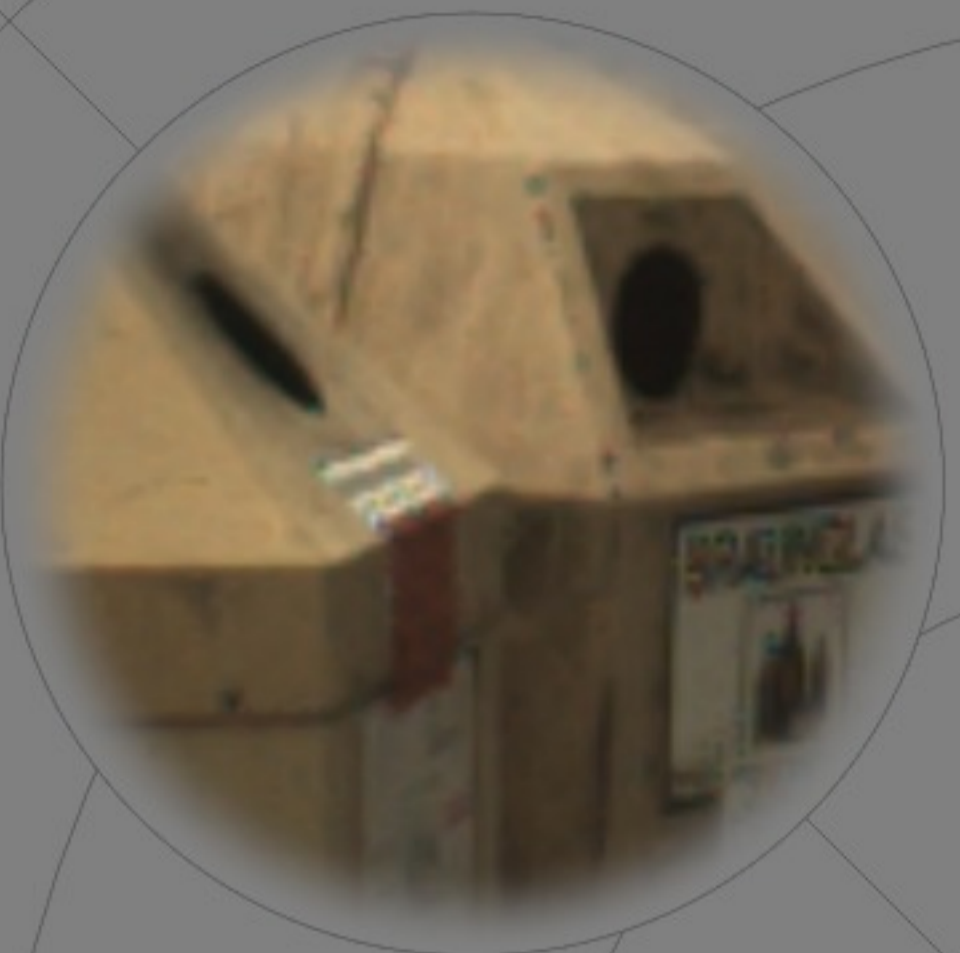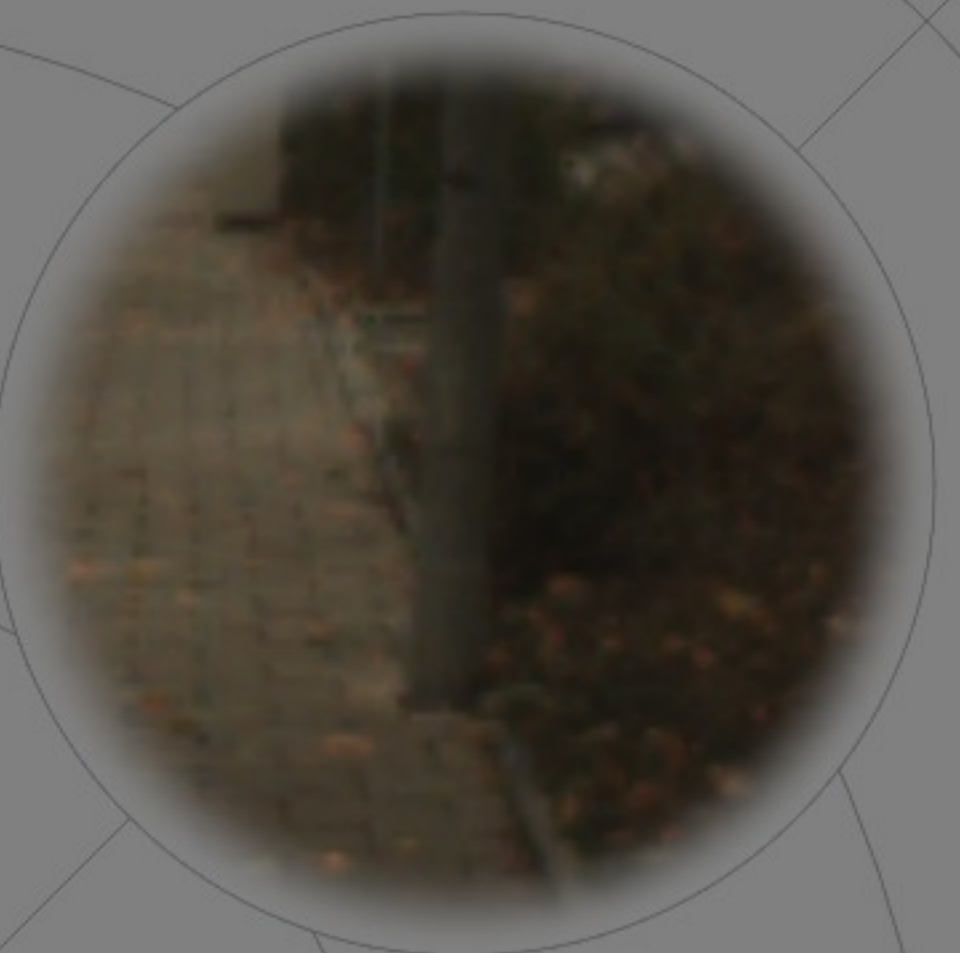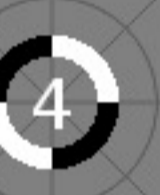

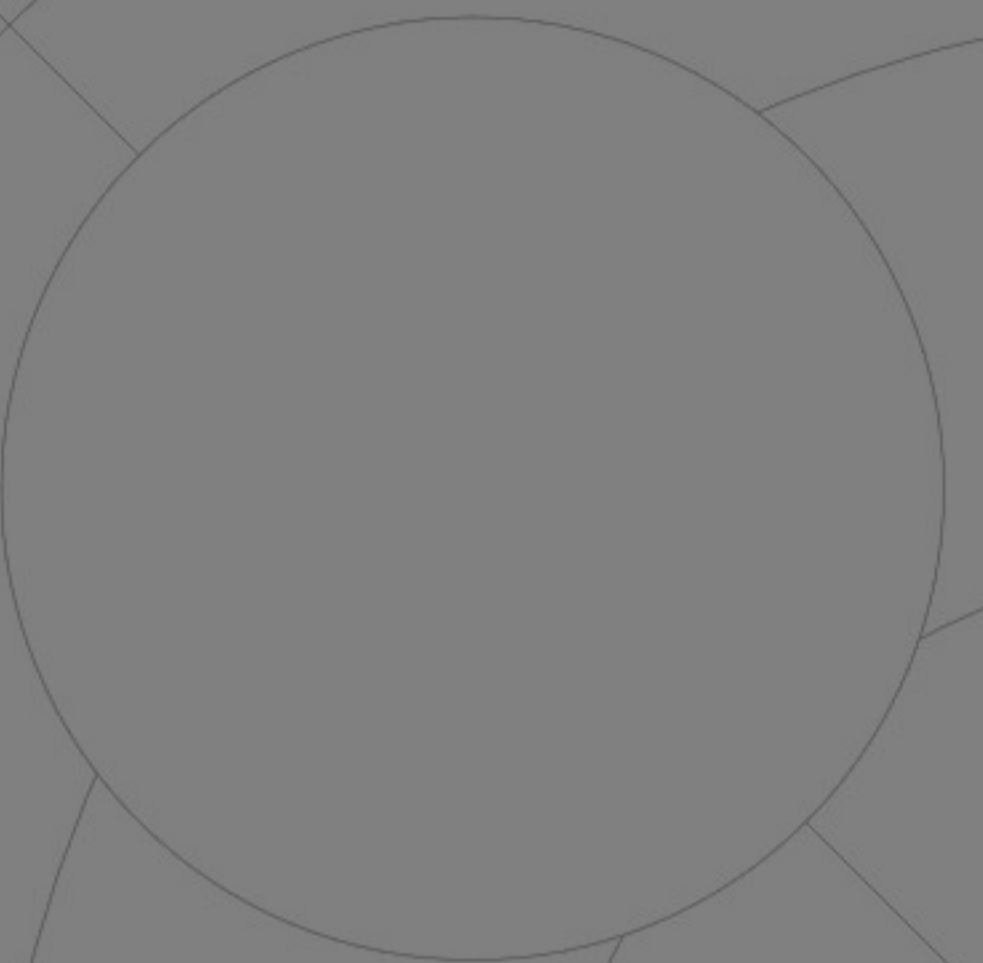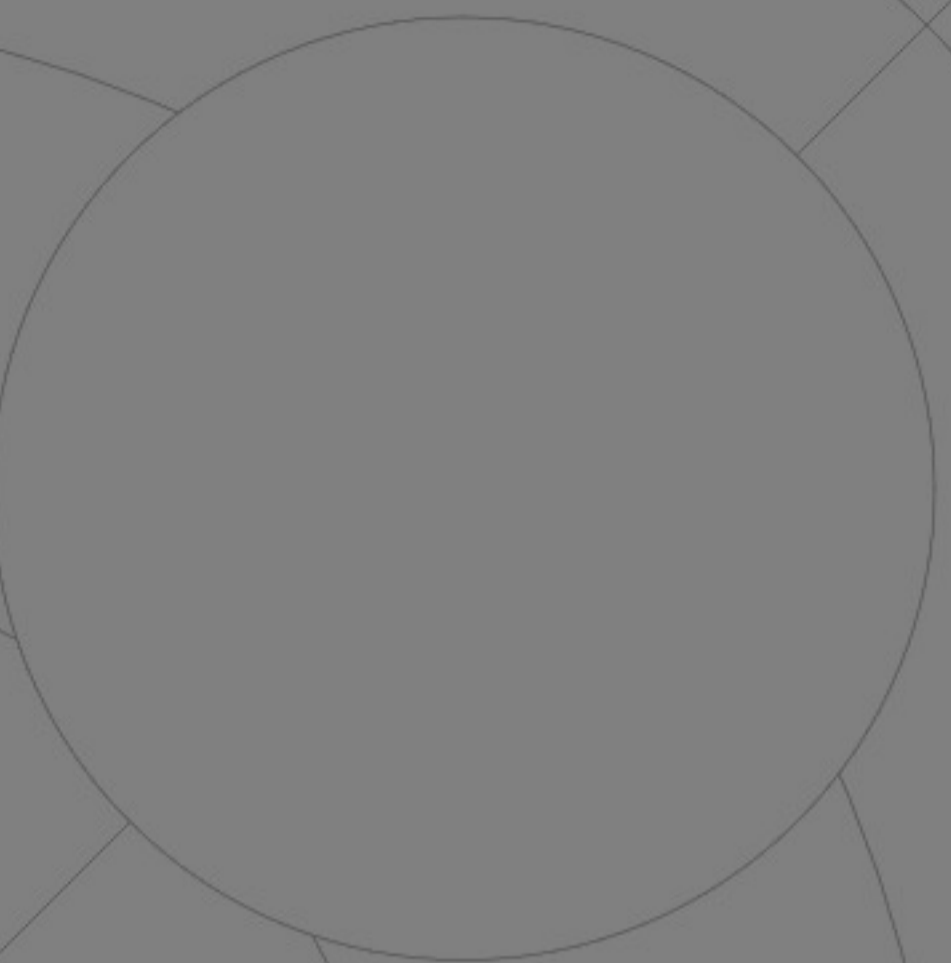

0

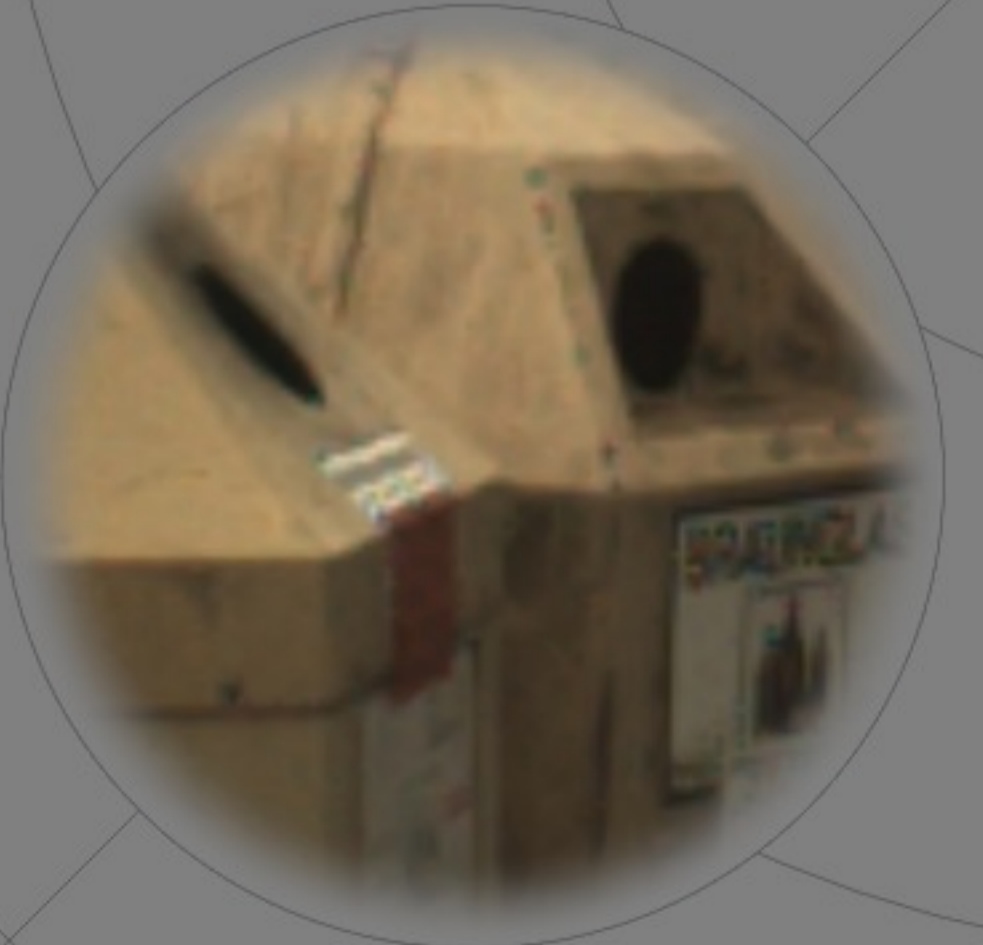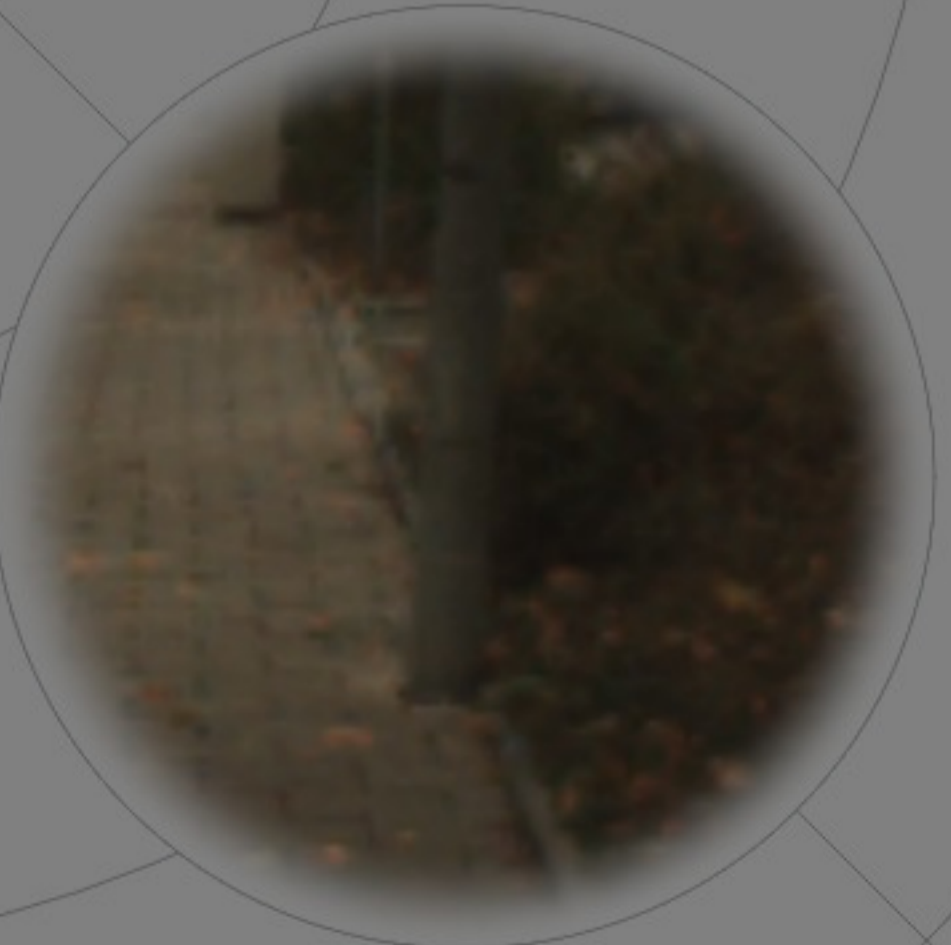

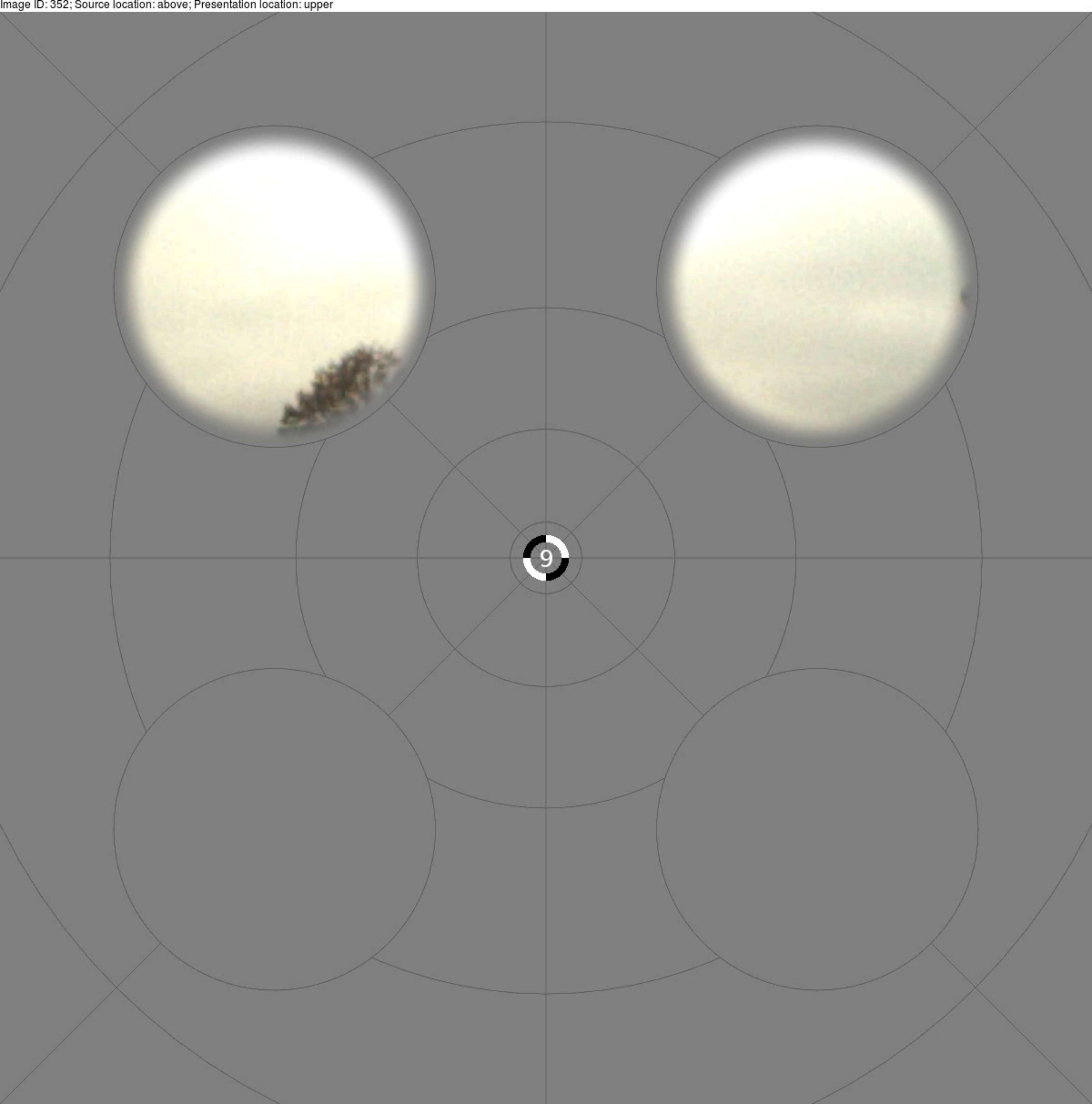

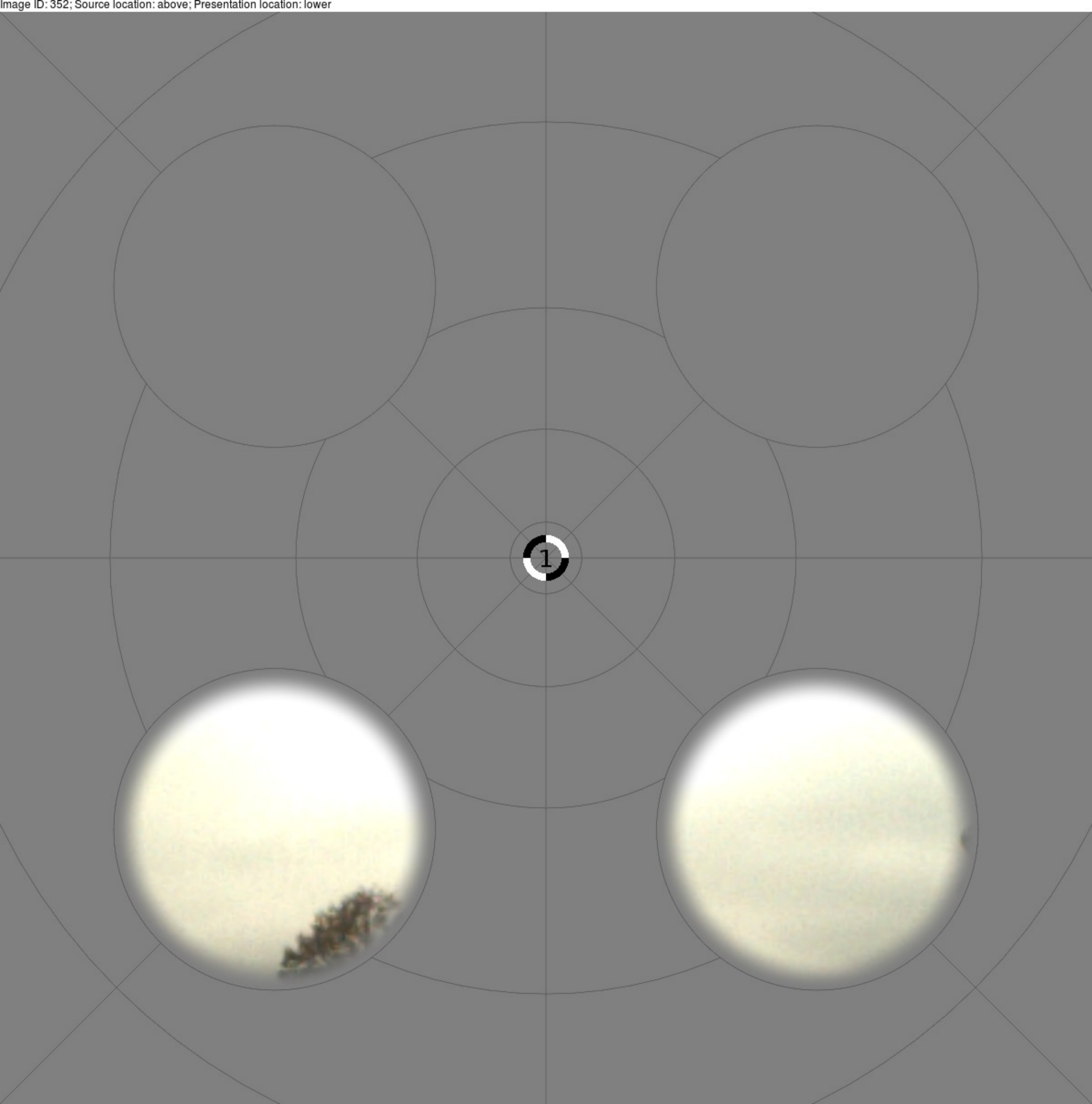

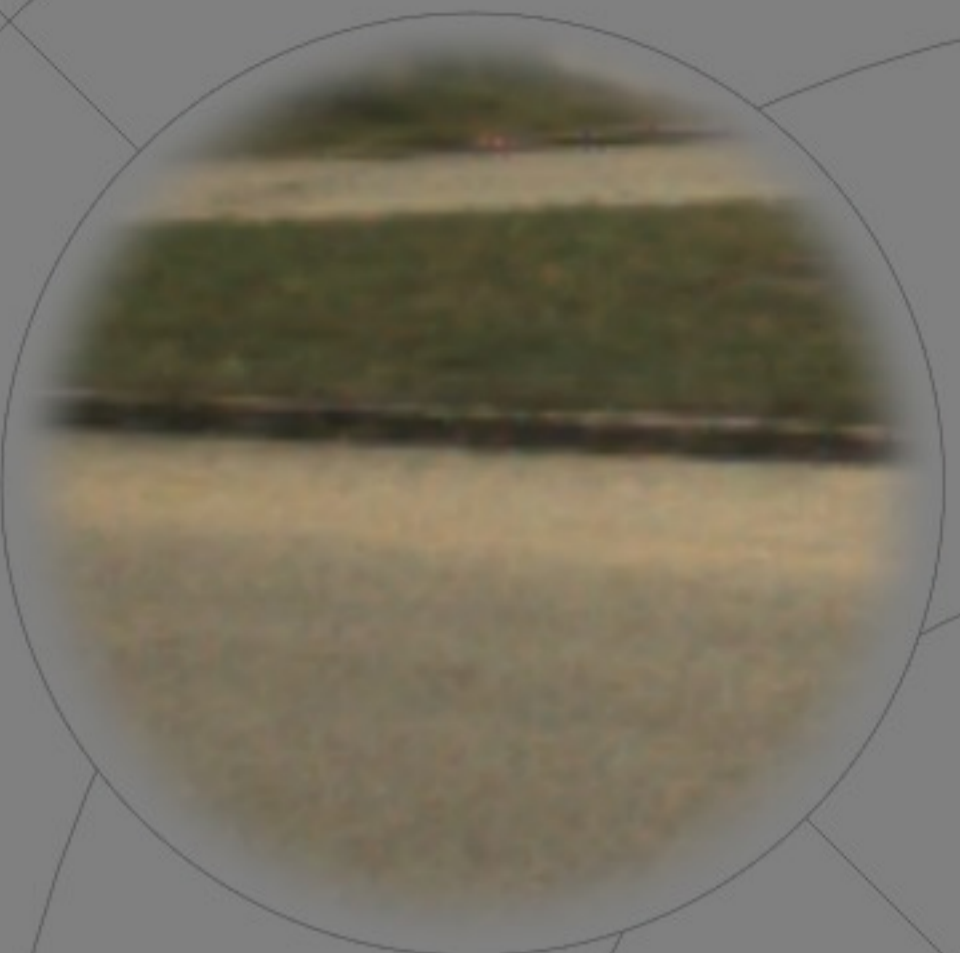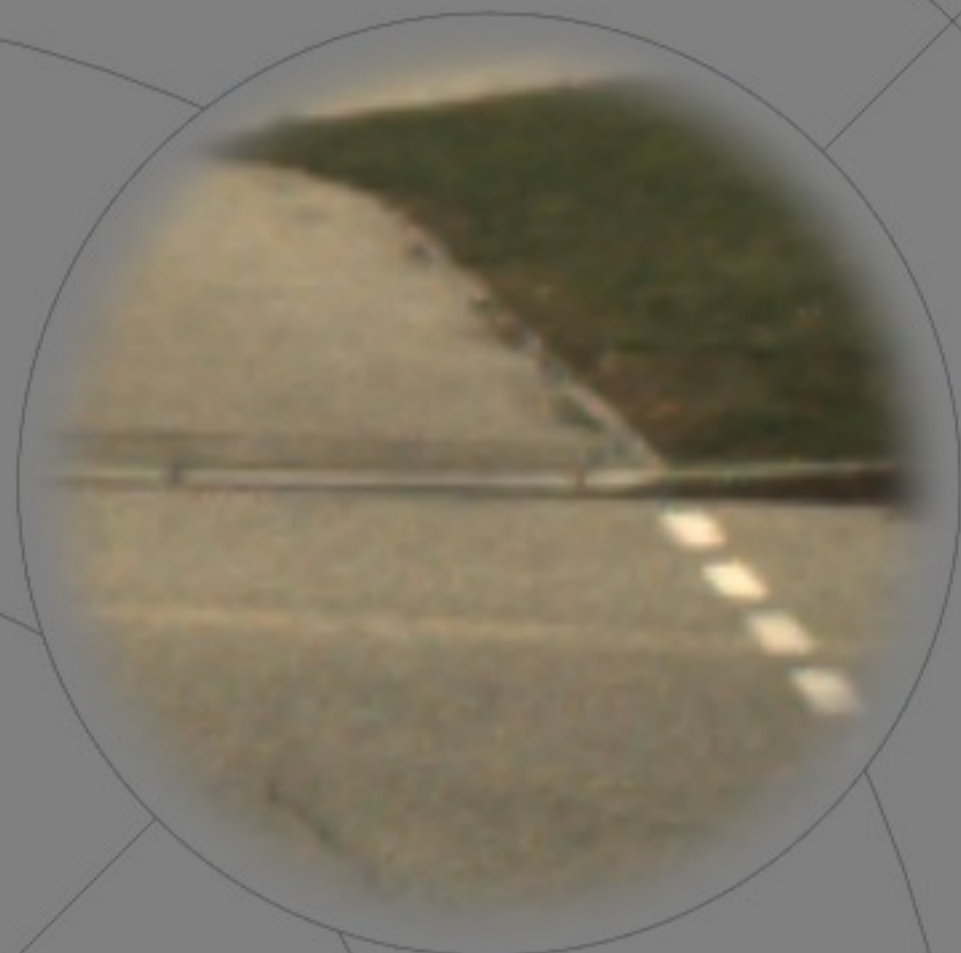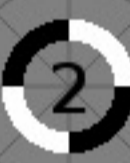

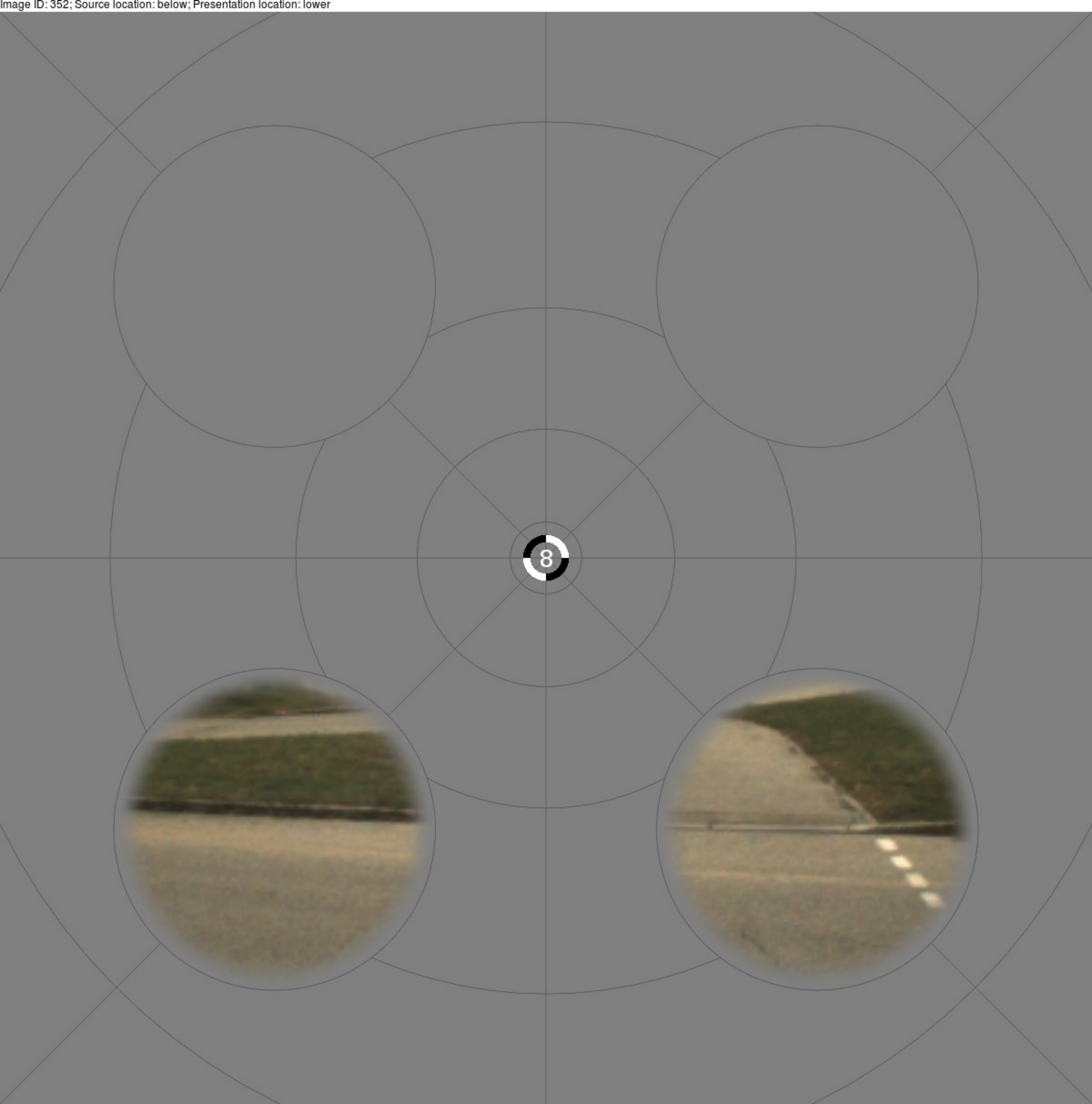

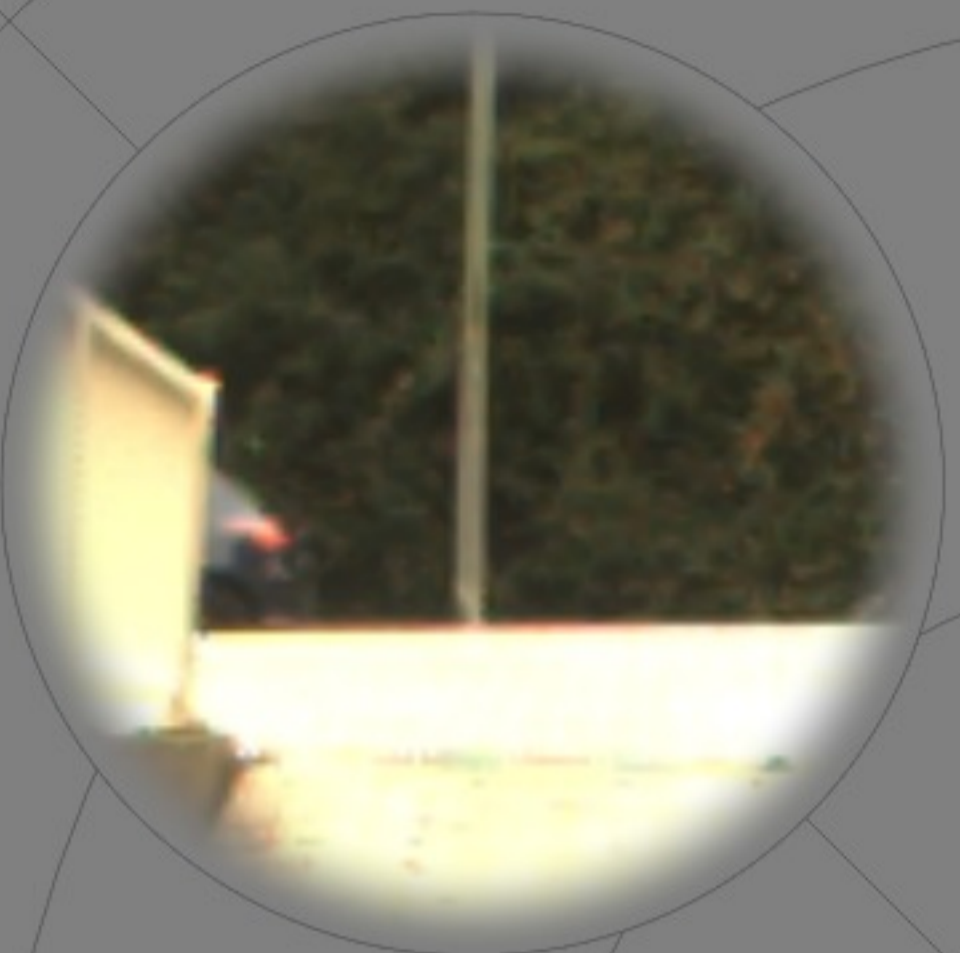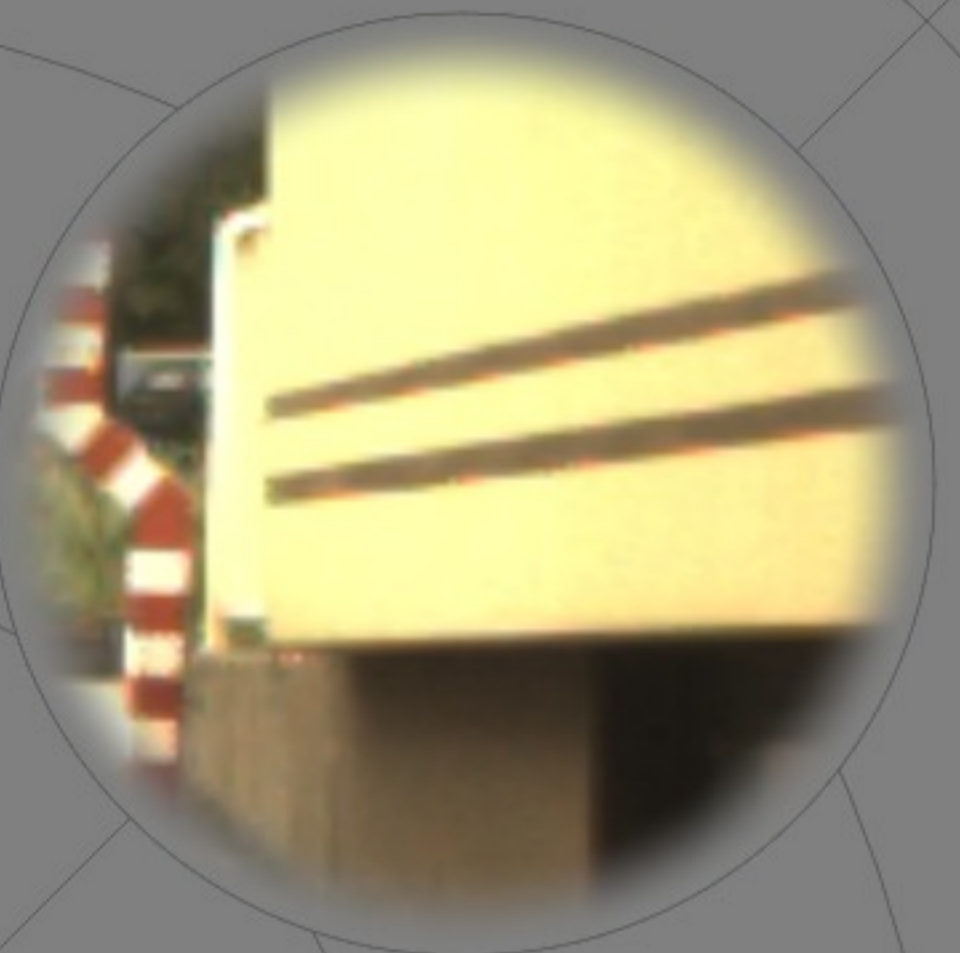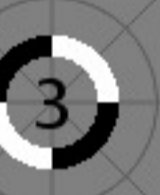

2

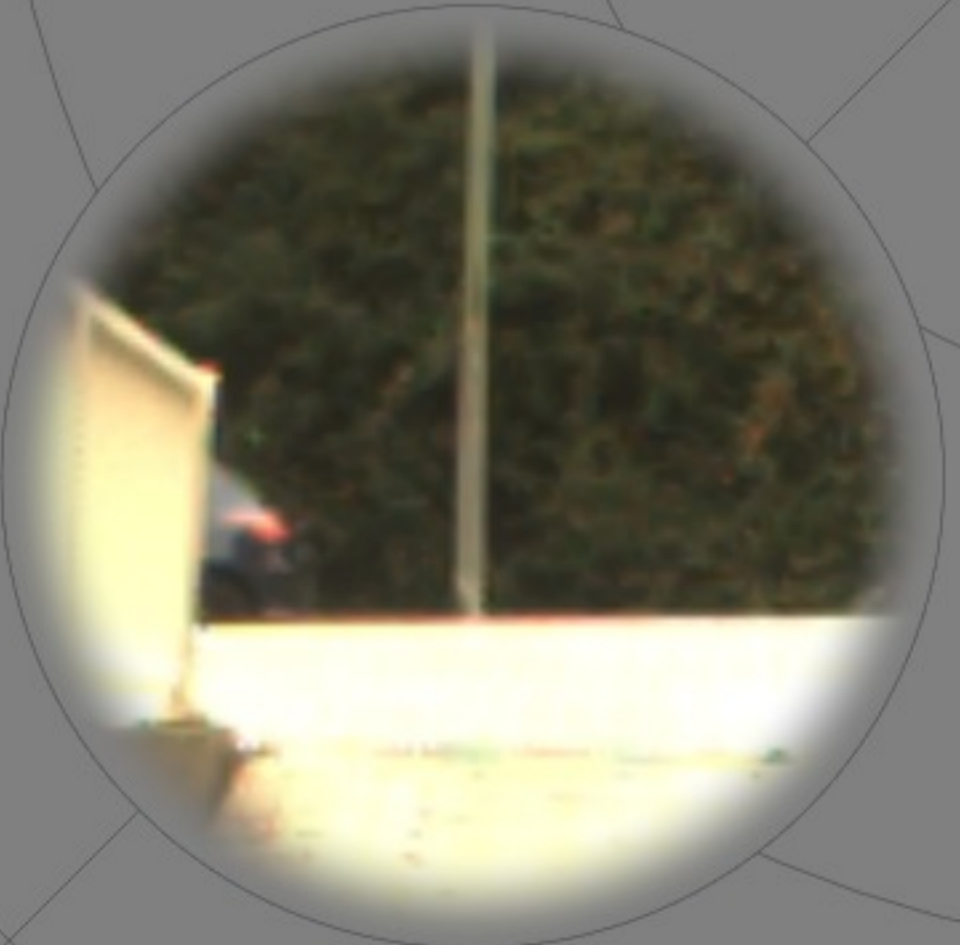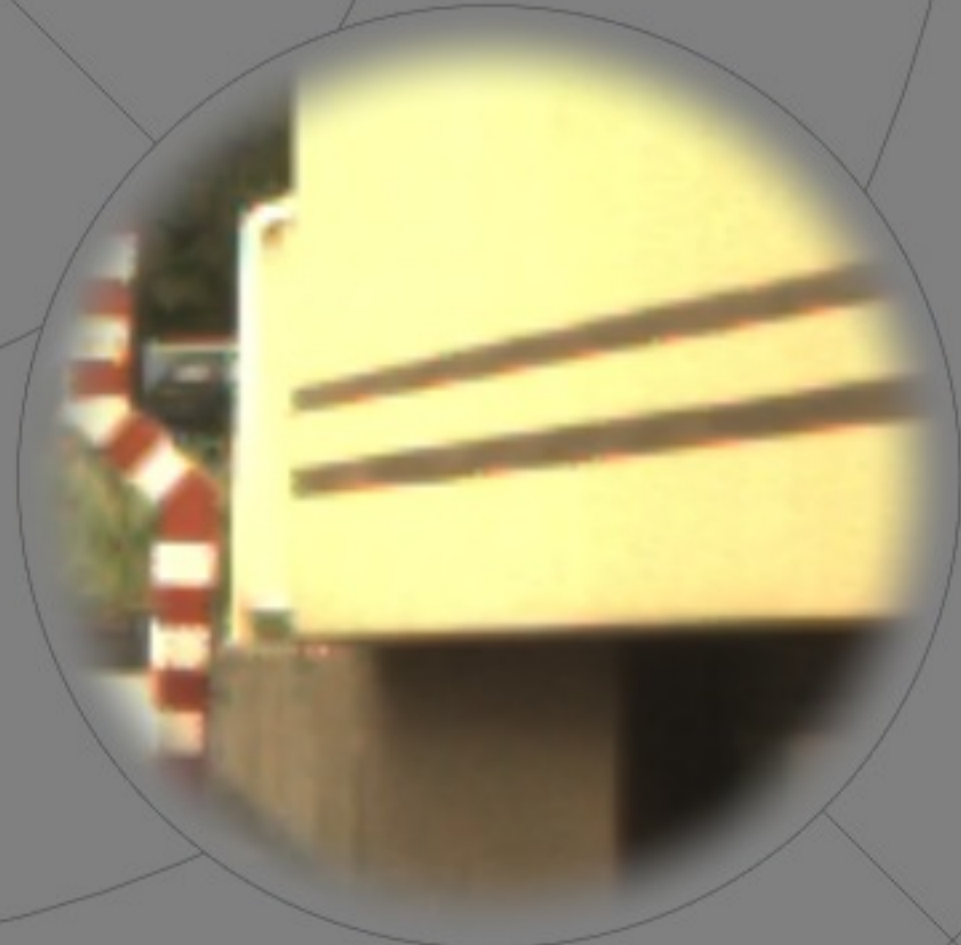

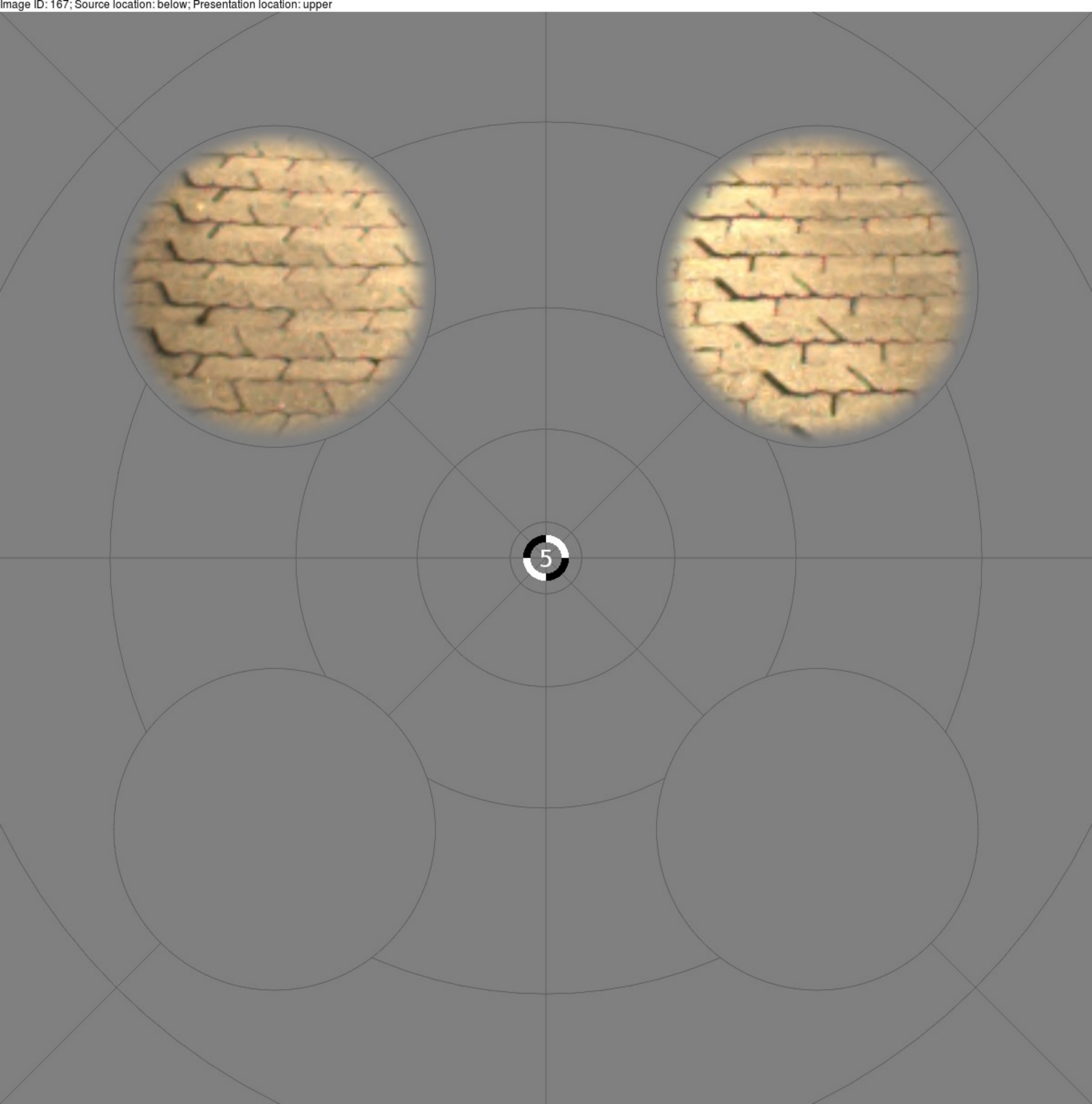

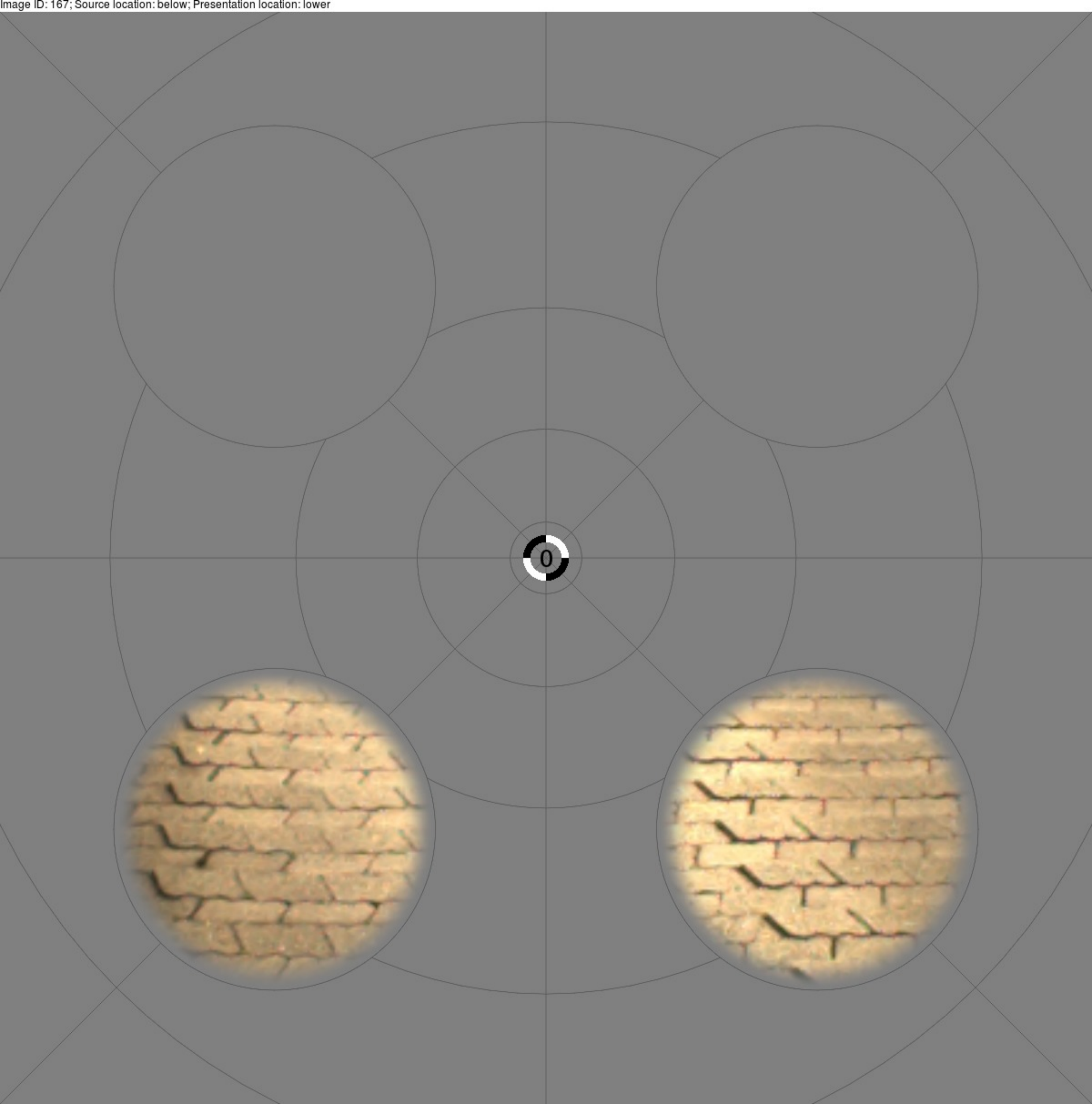

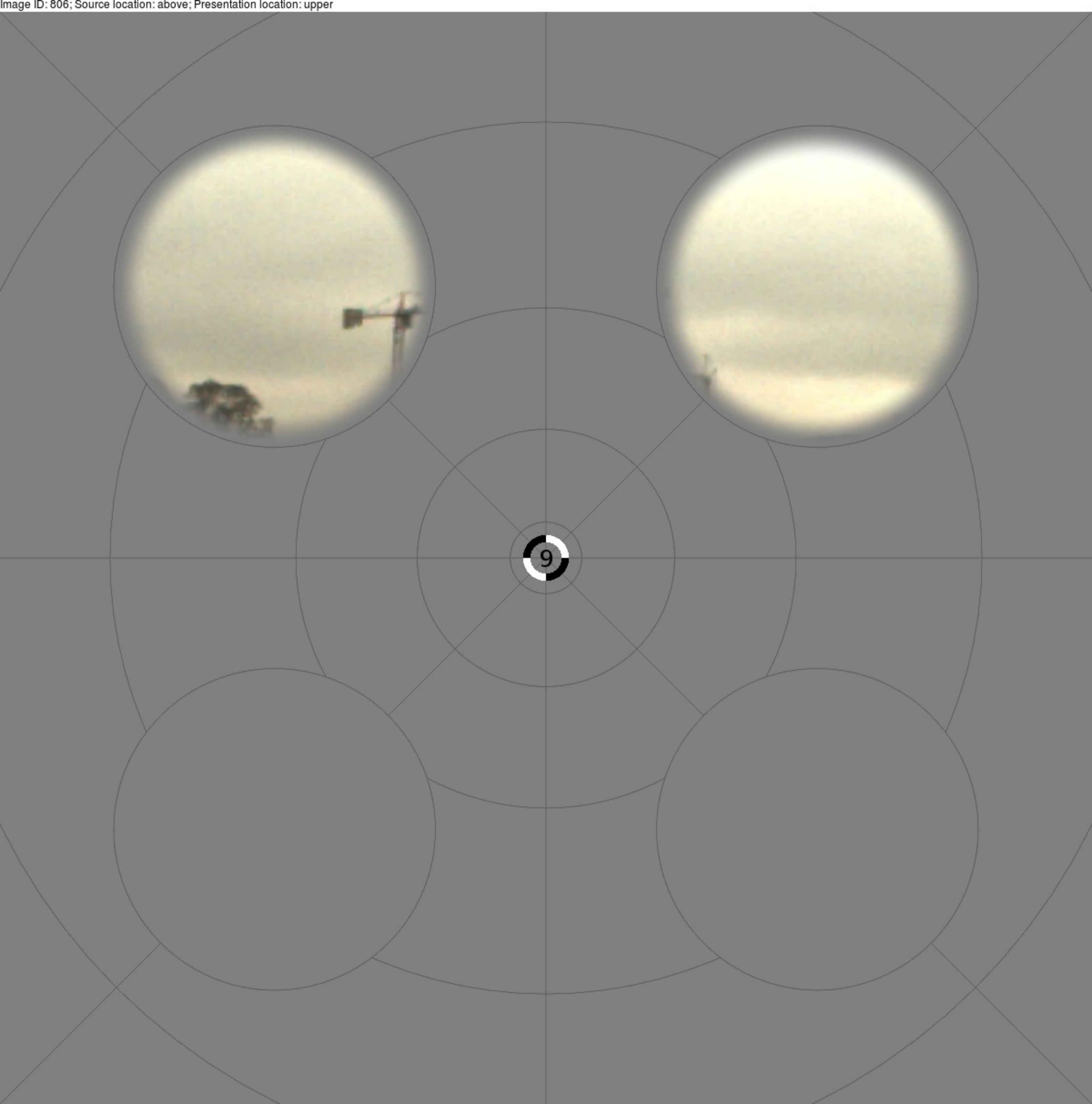

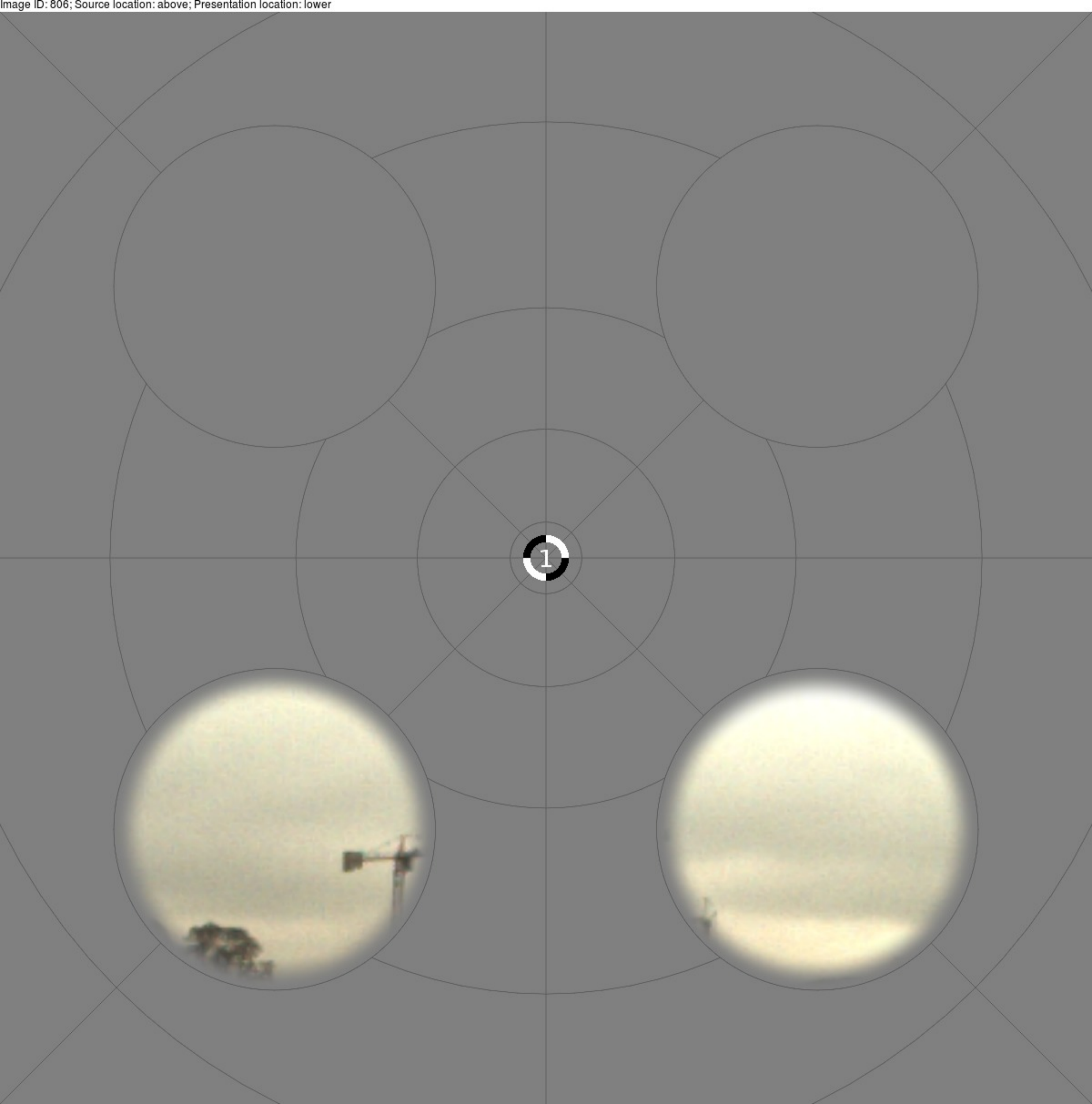

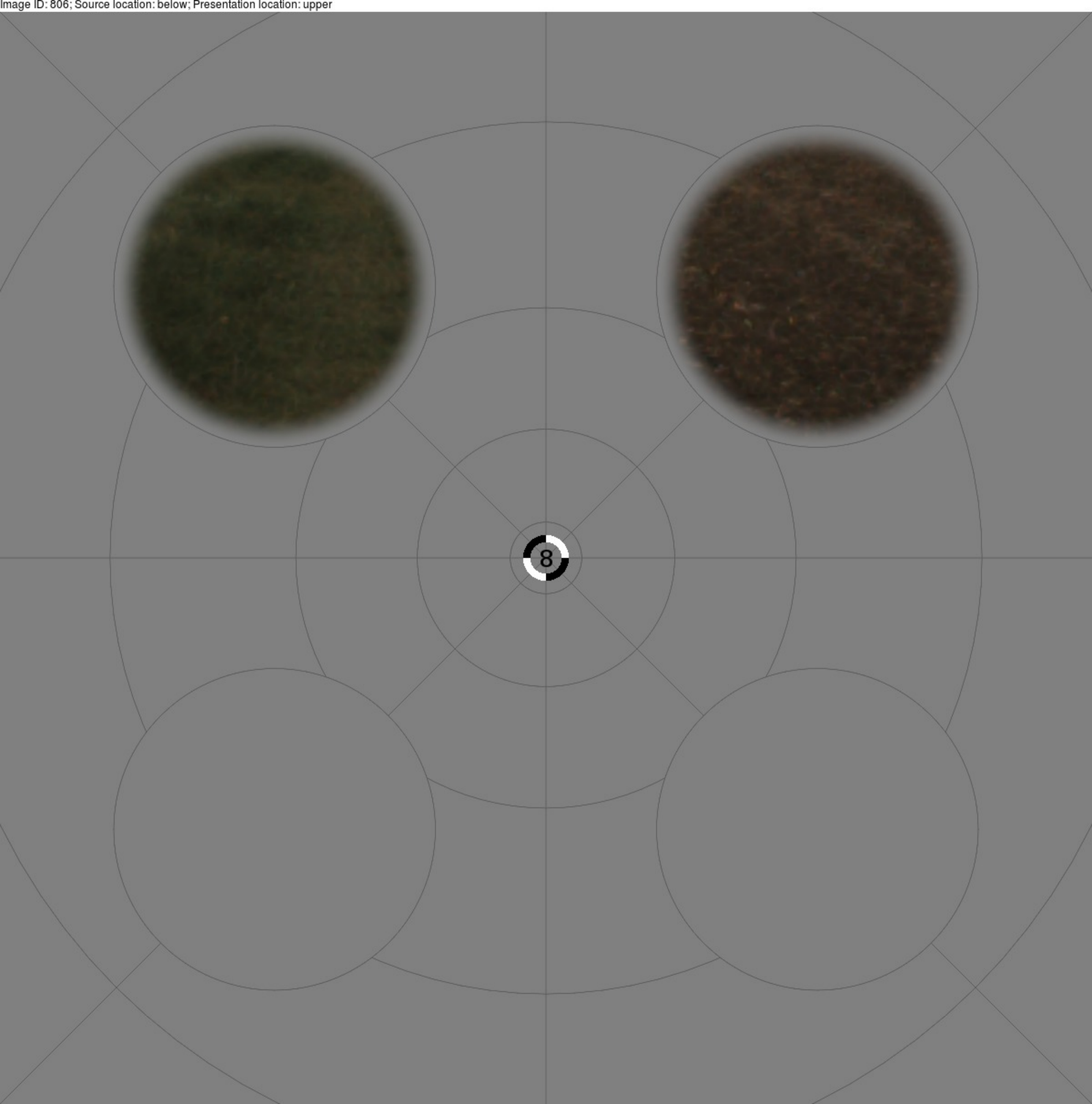

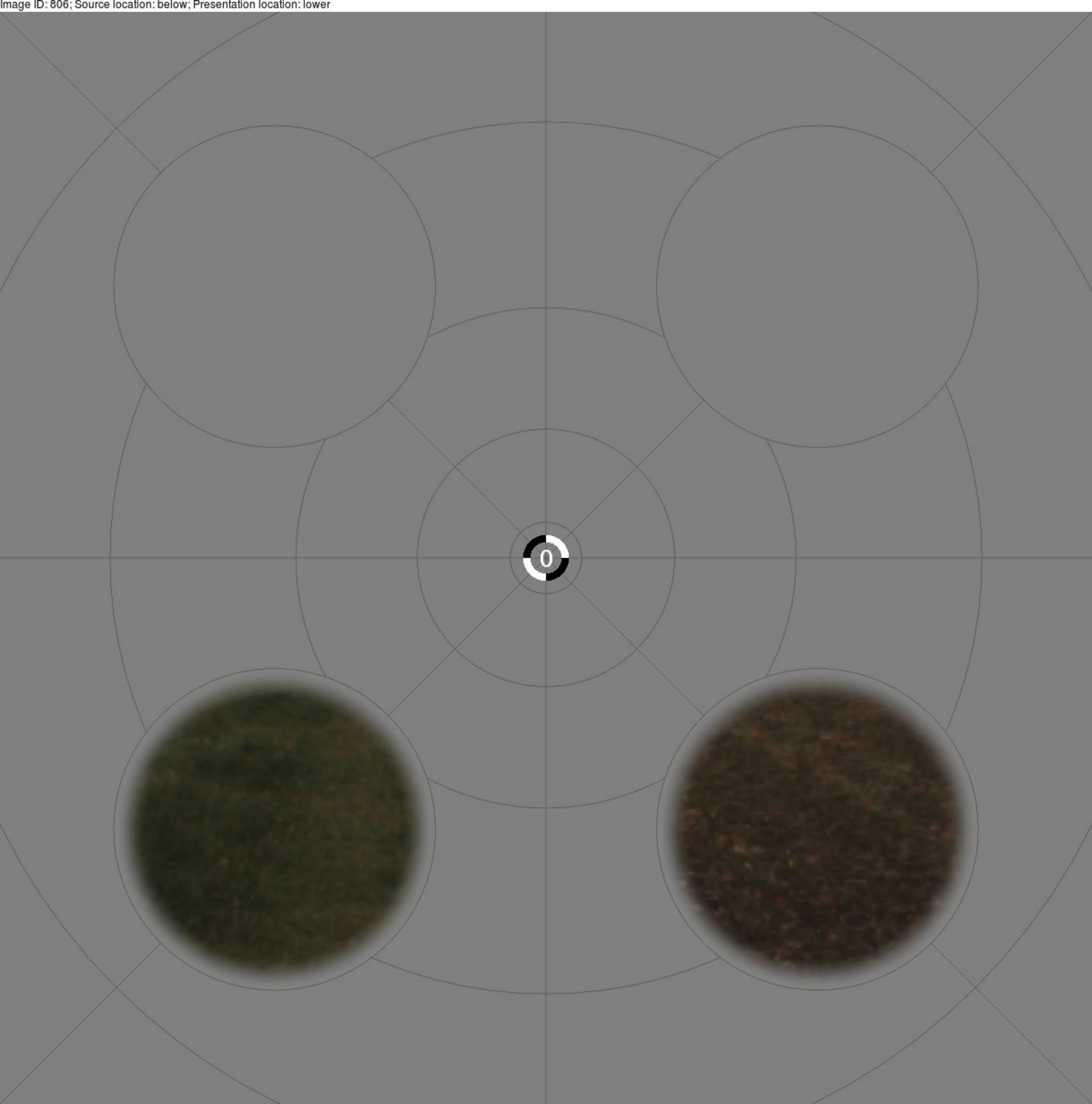

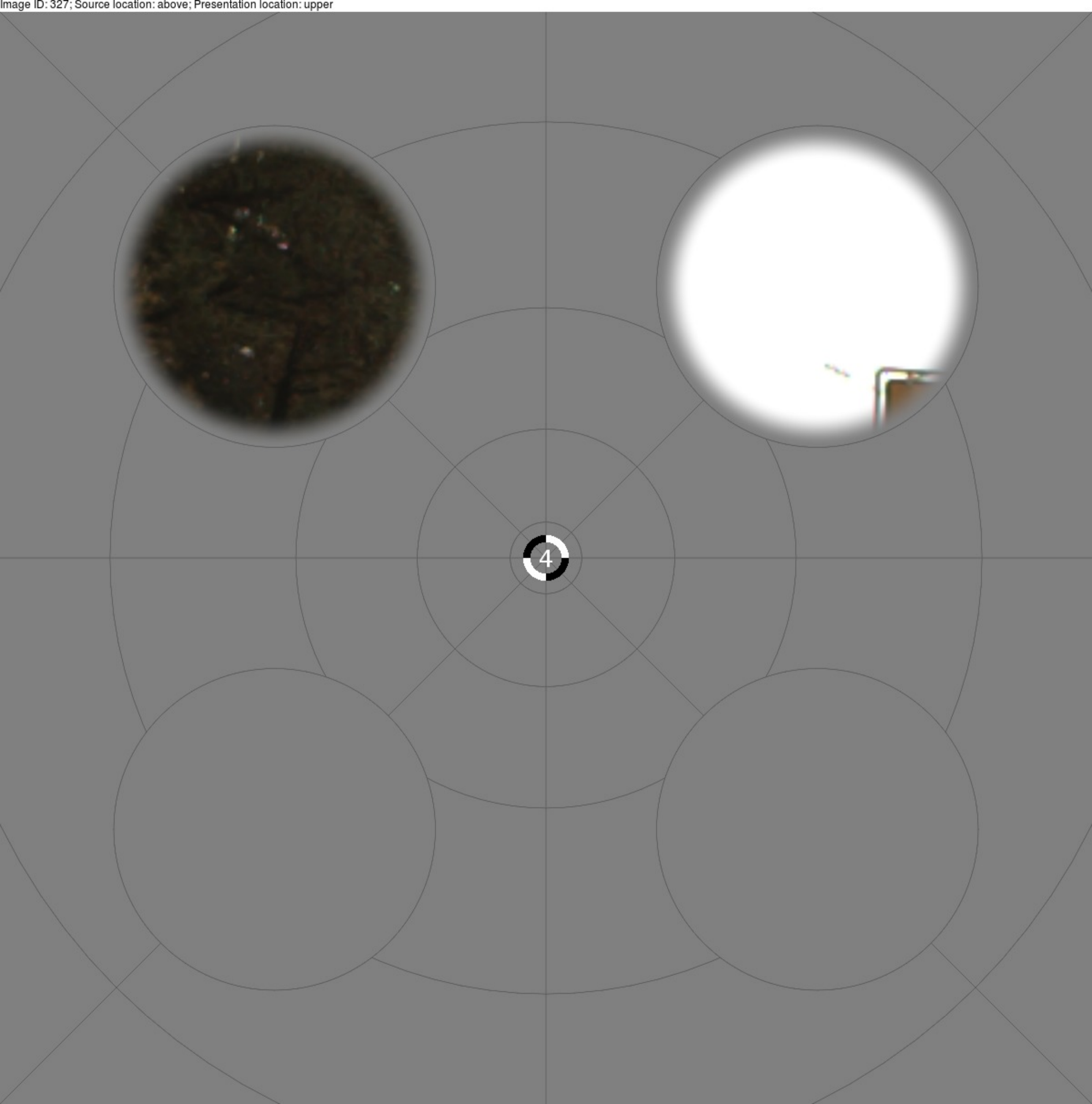

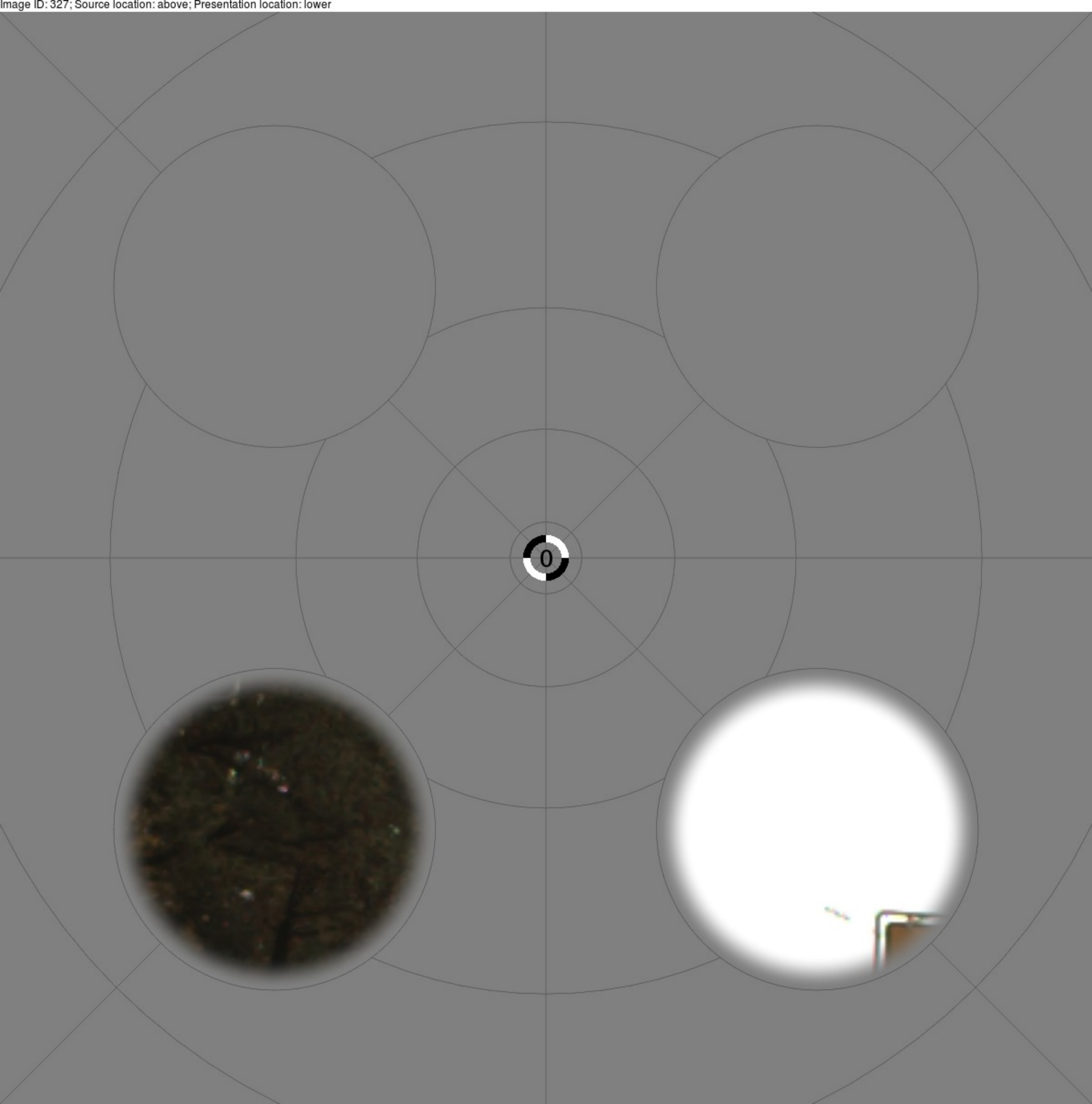

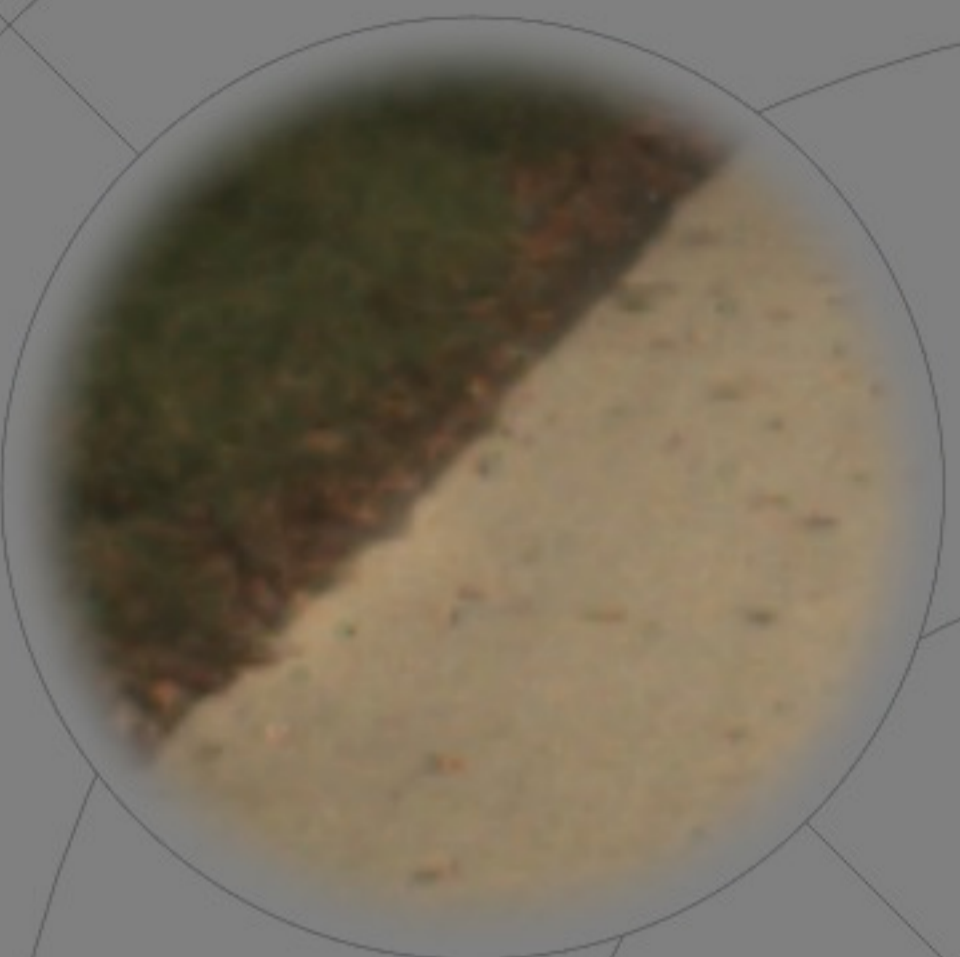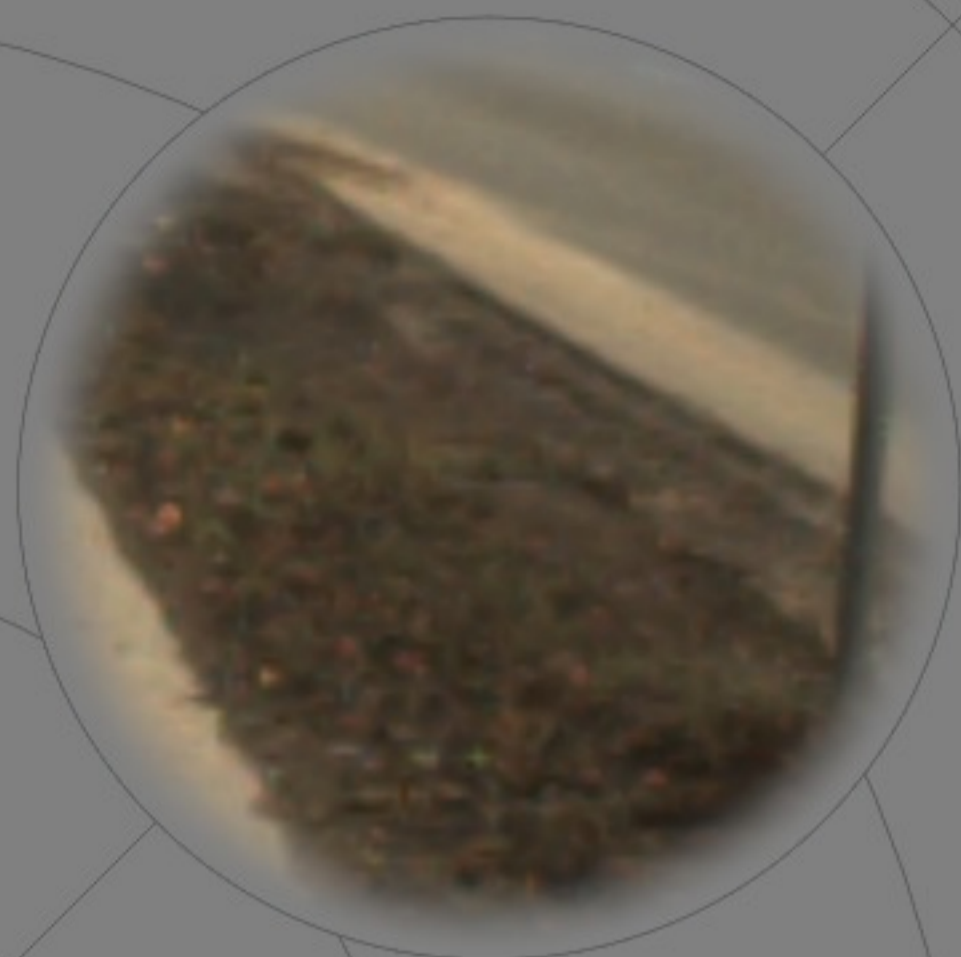

2

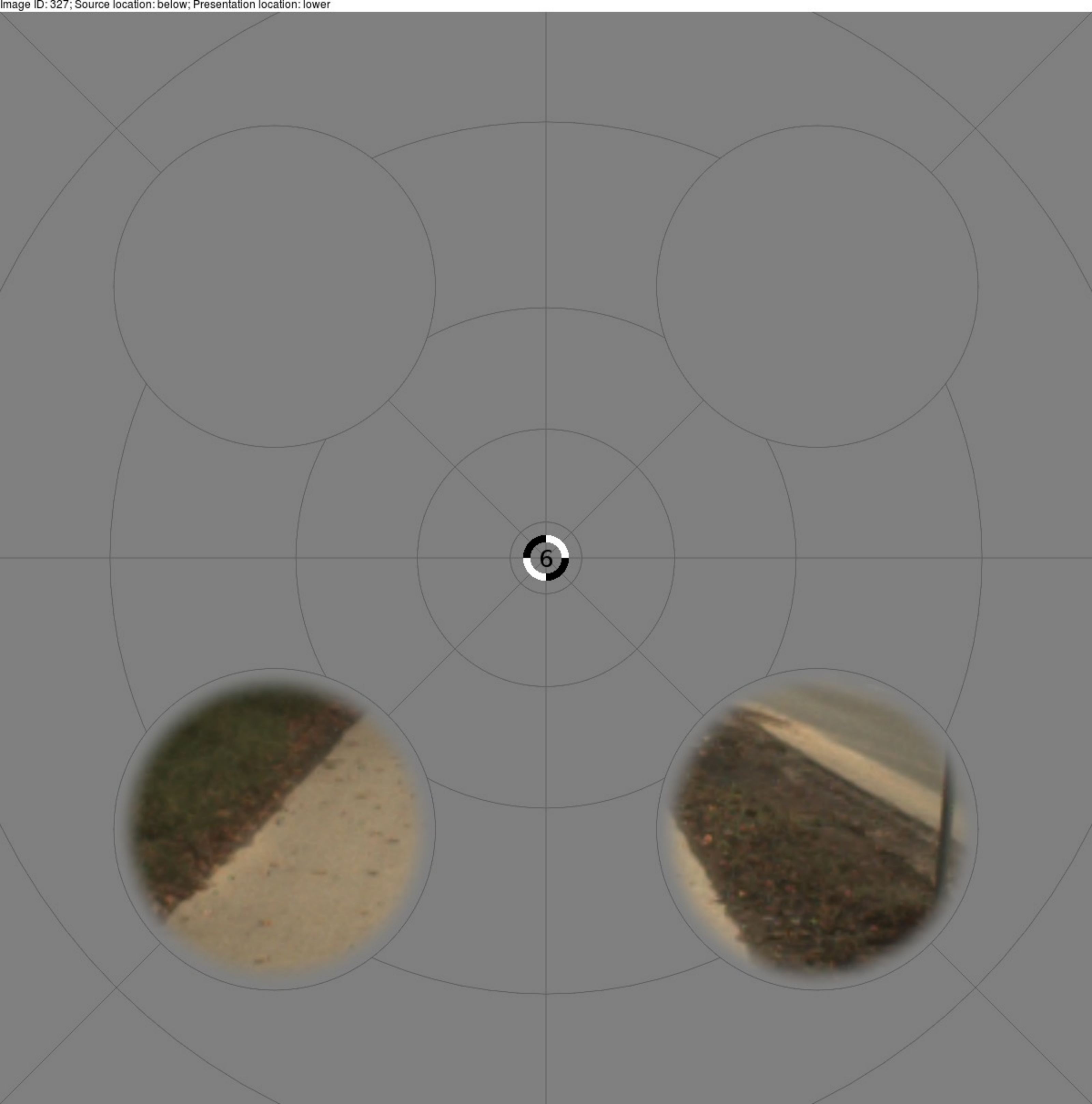

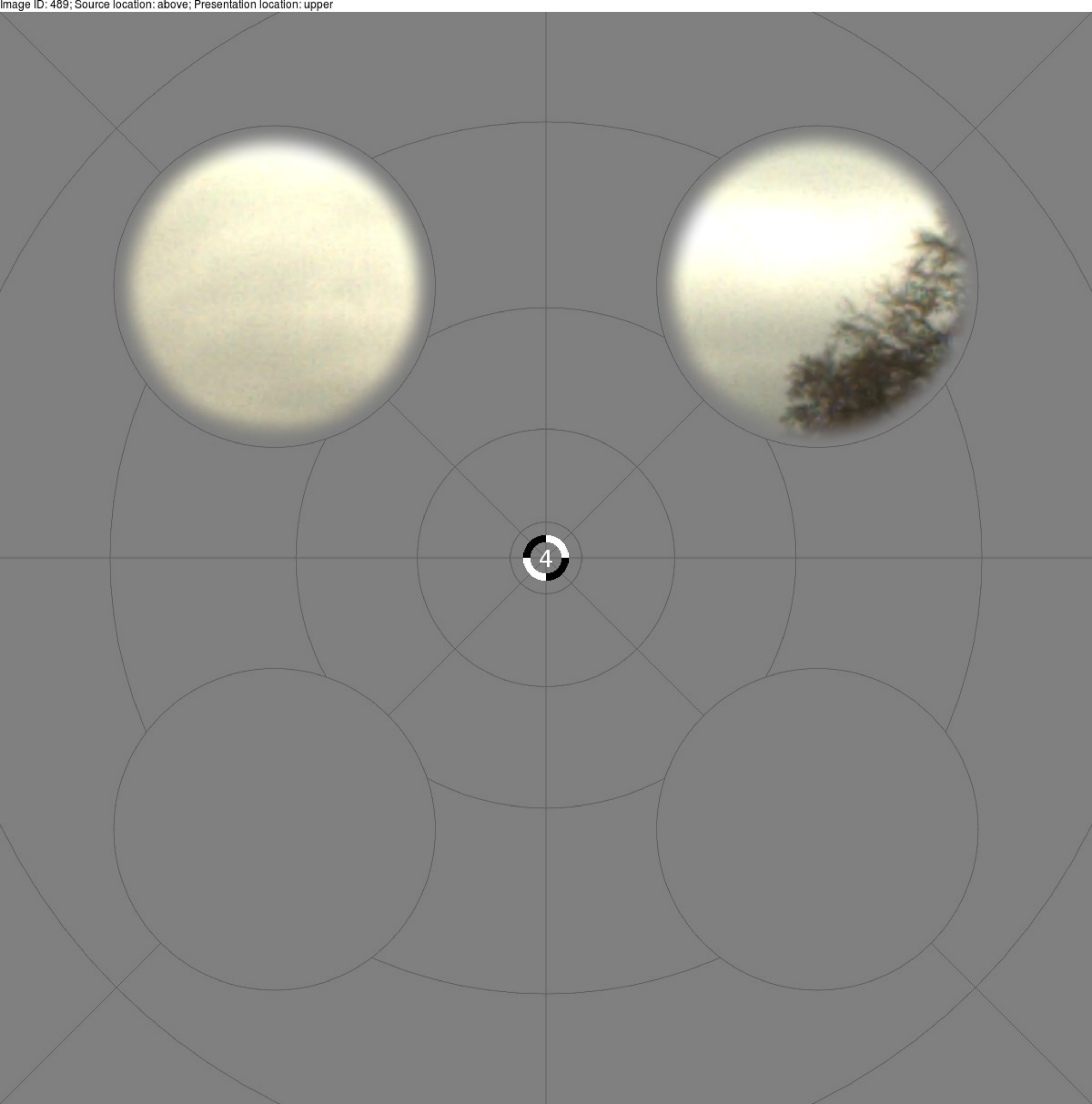

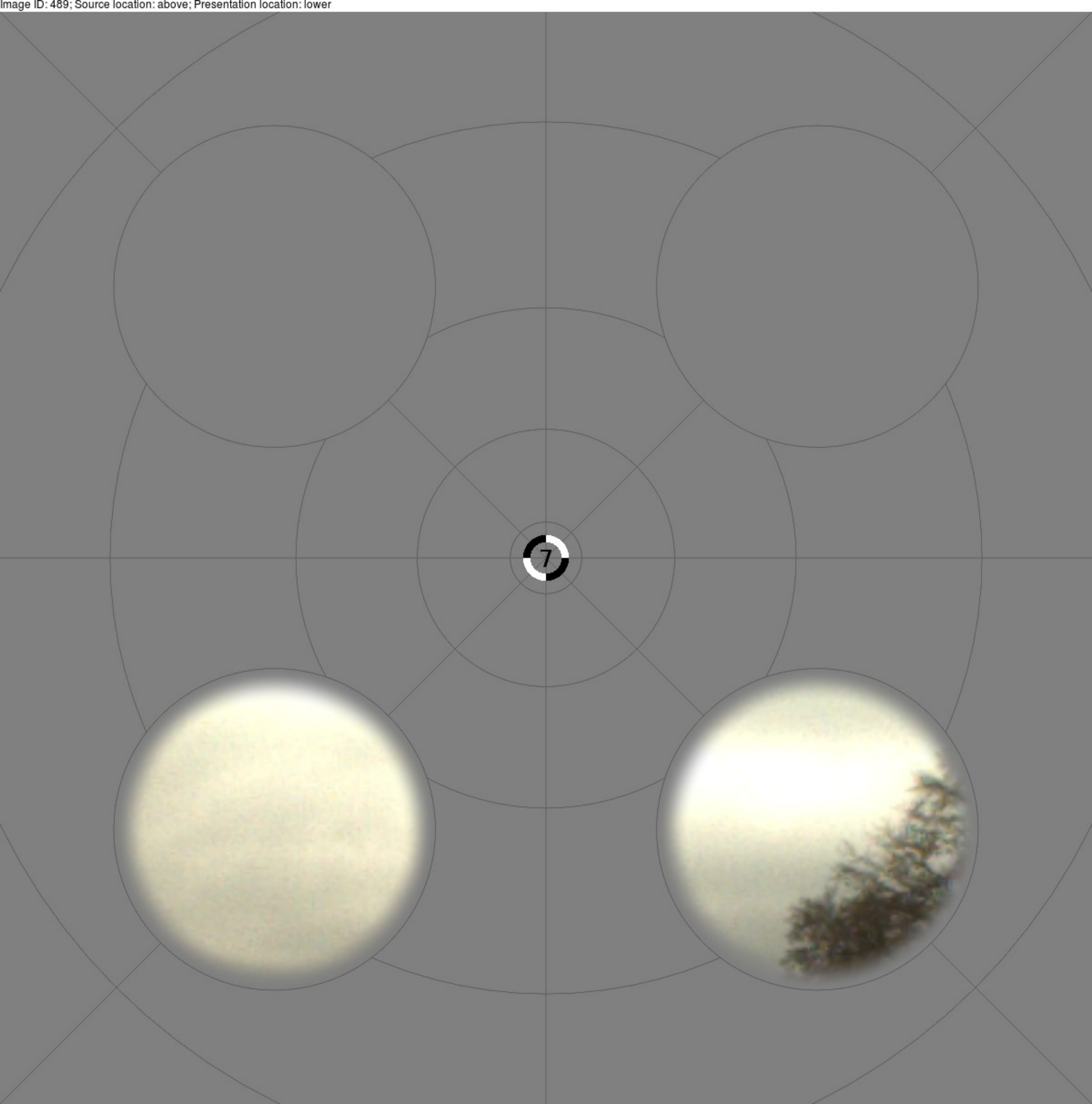

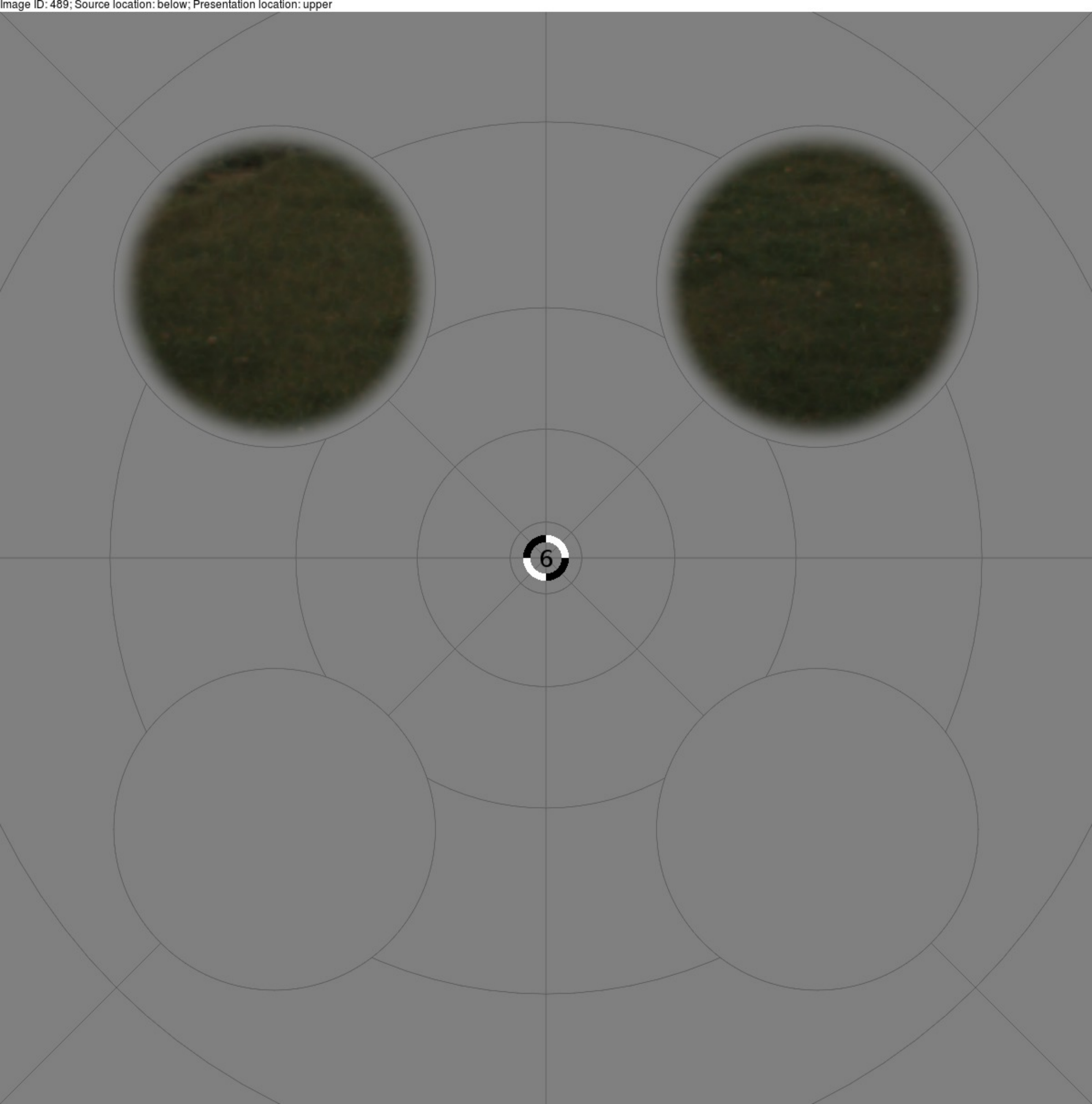

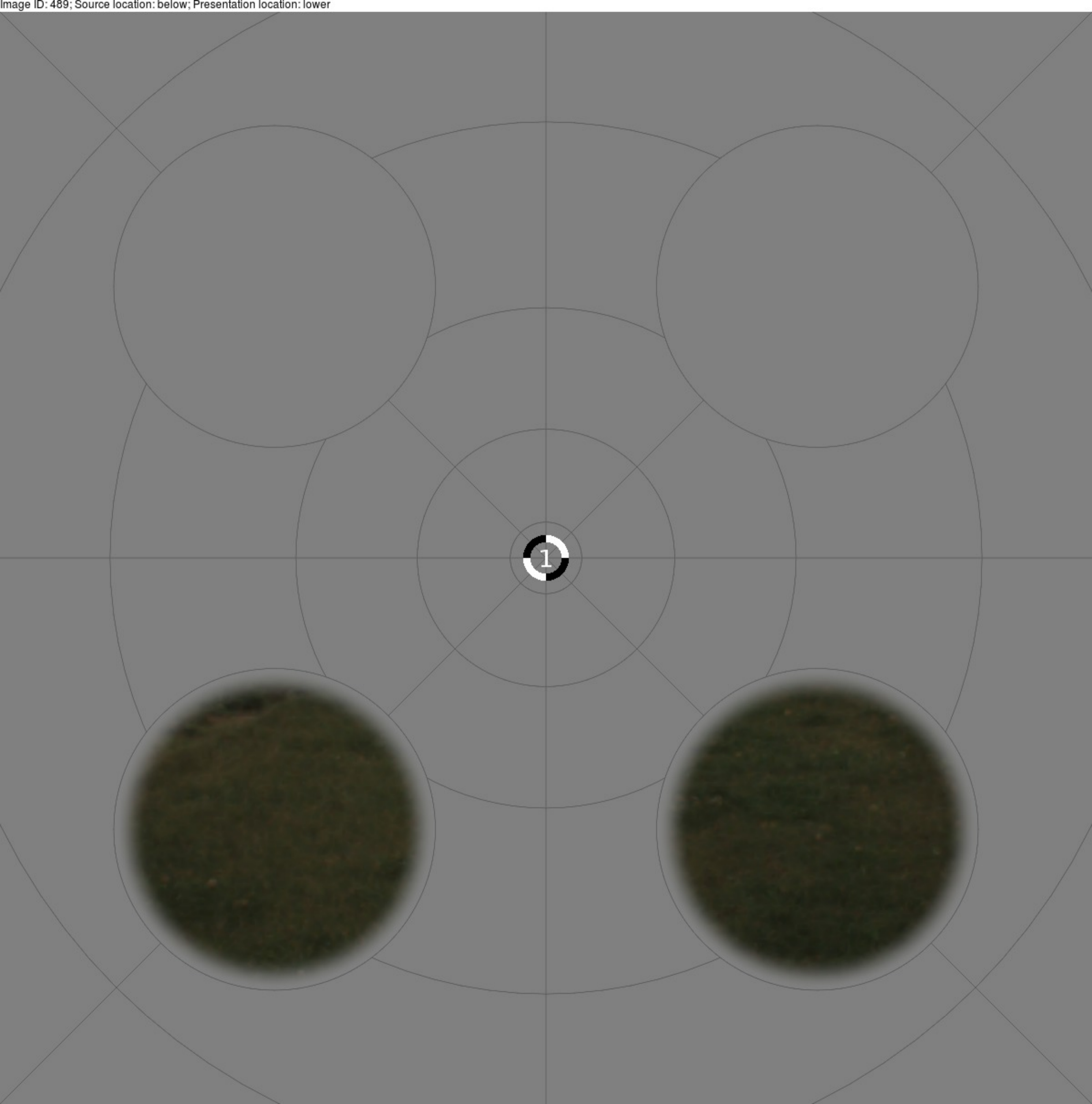

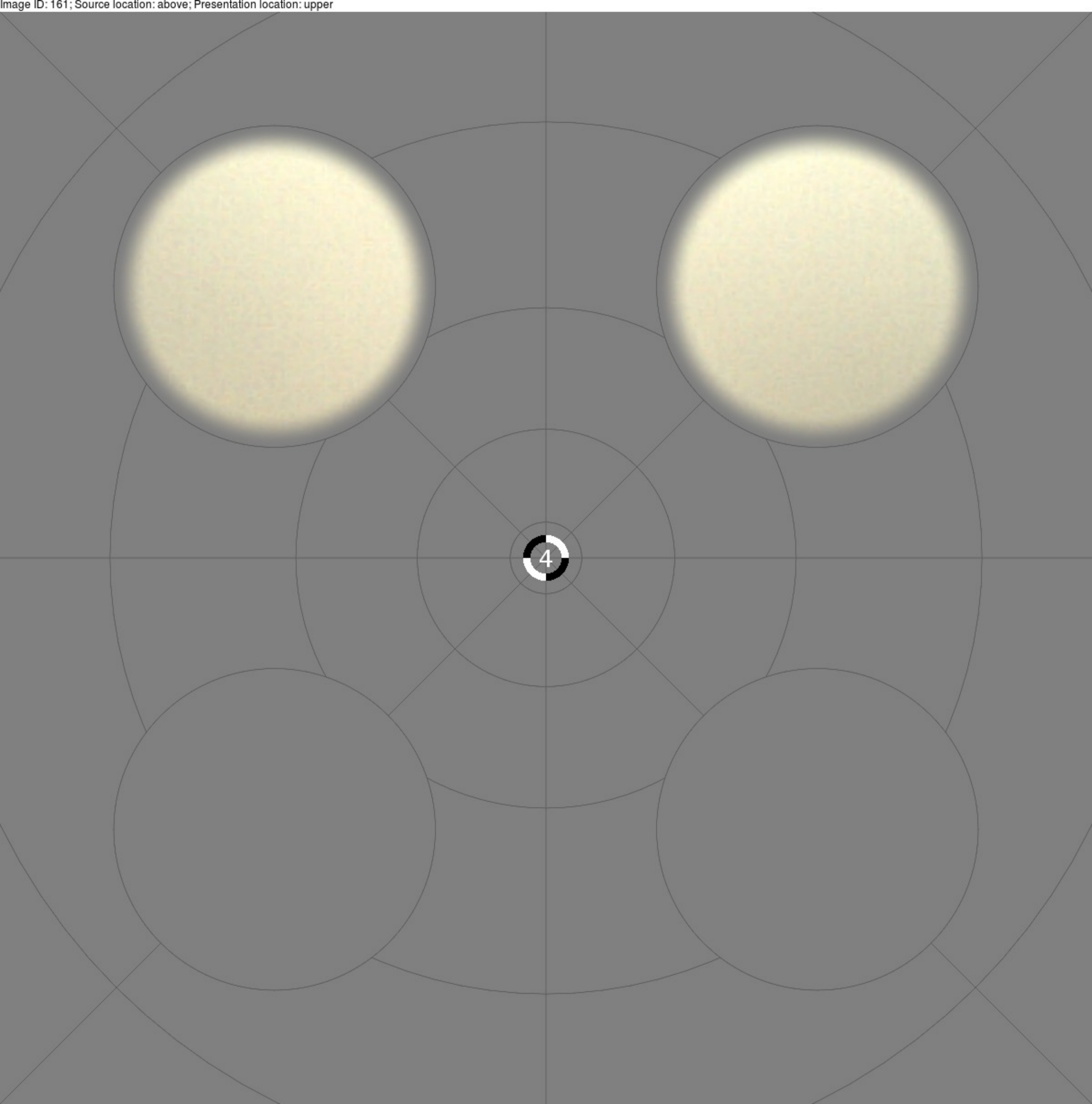

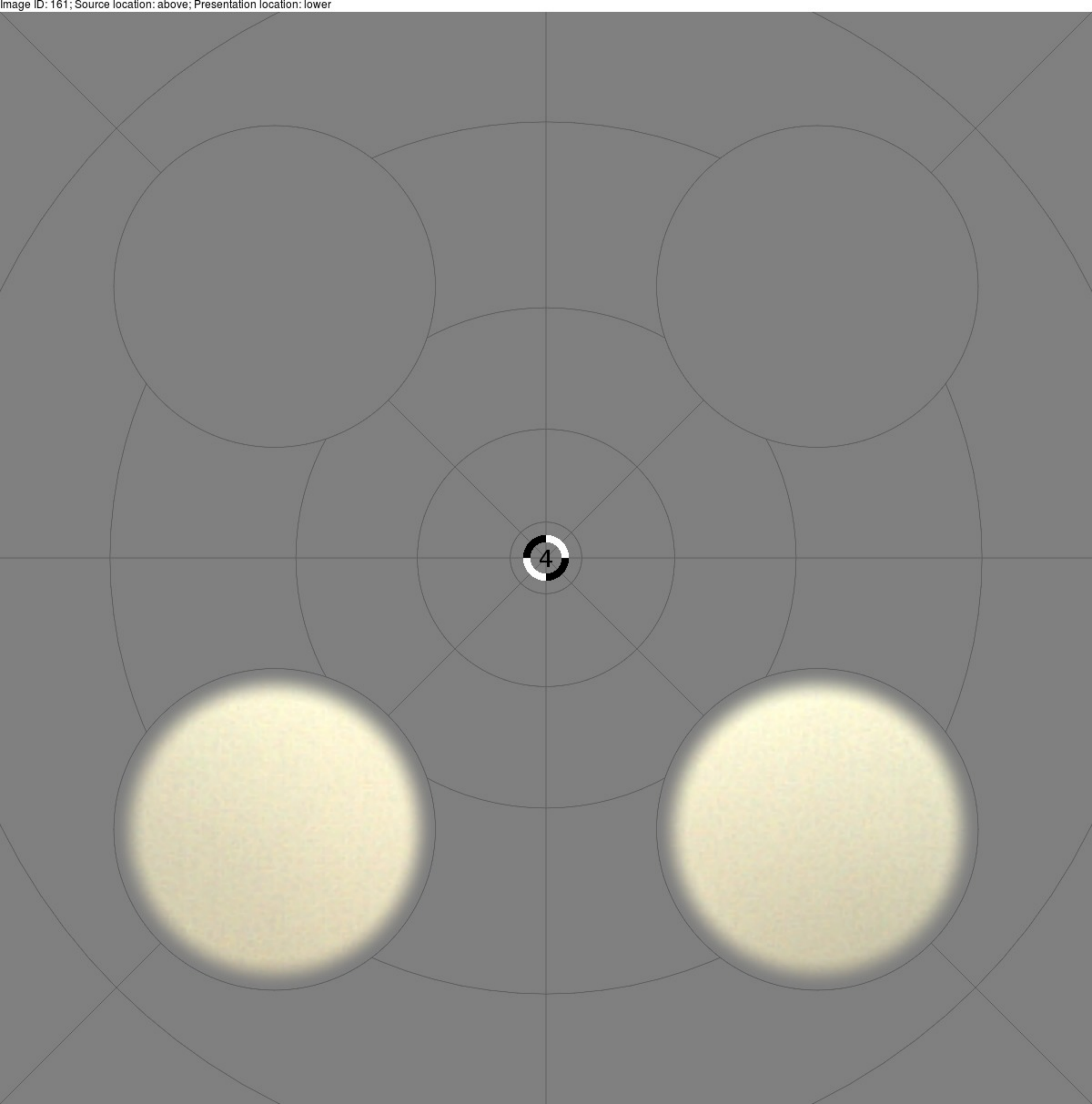

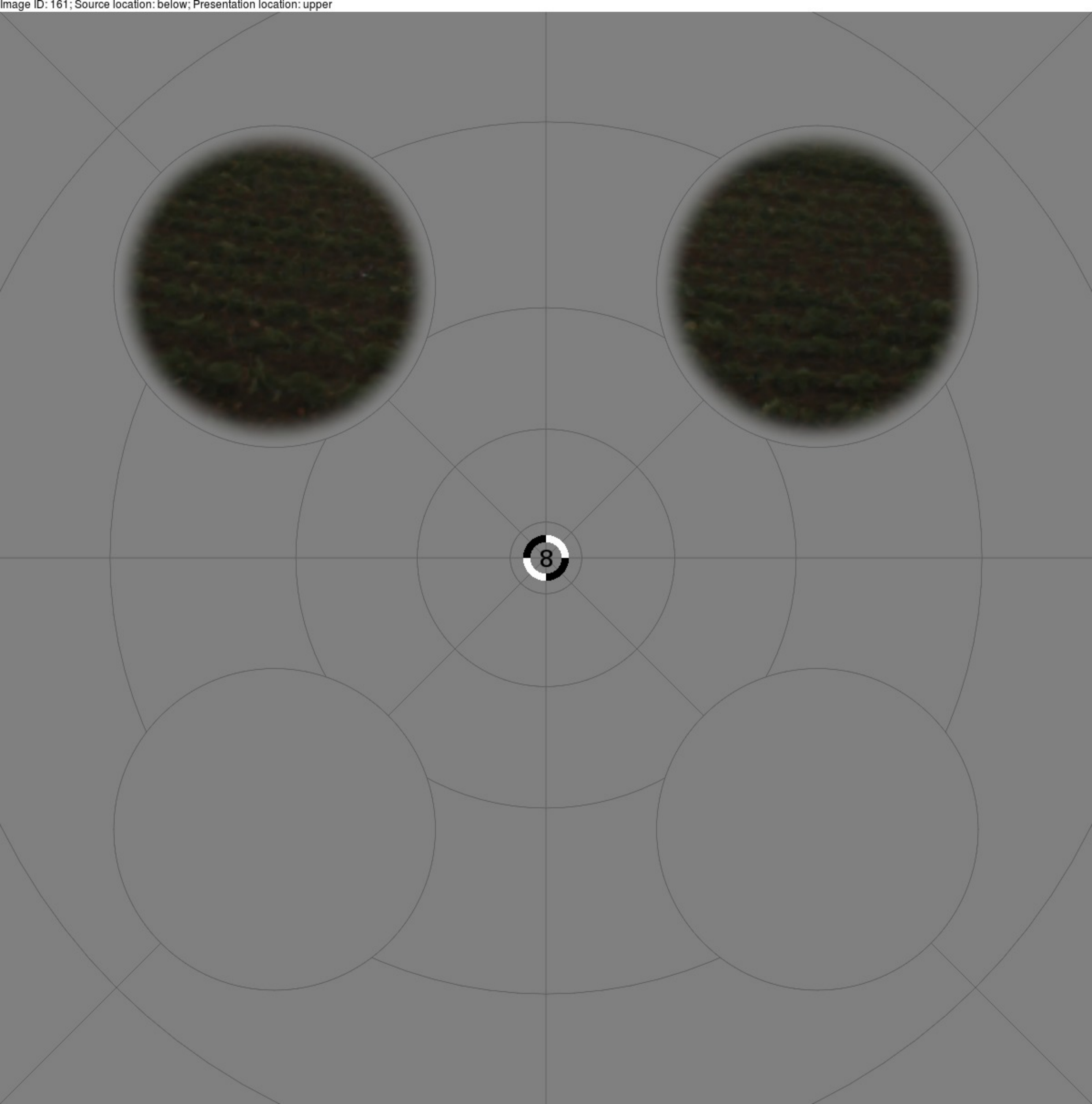

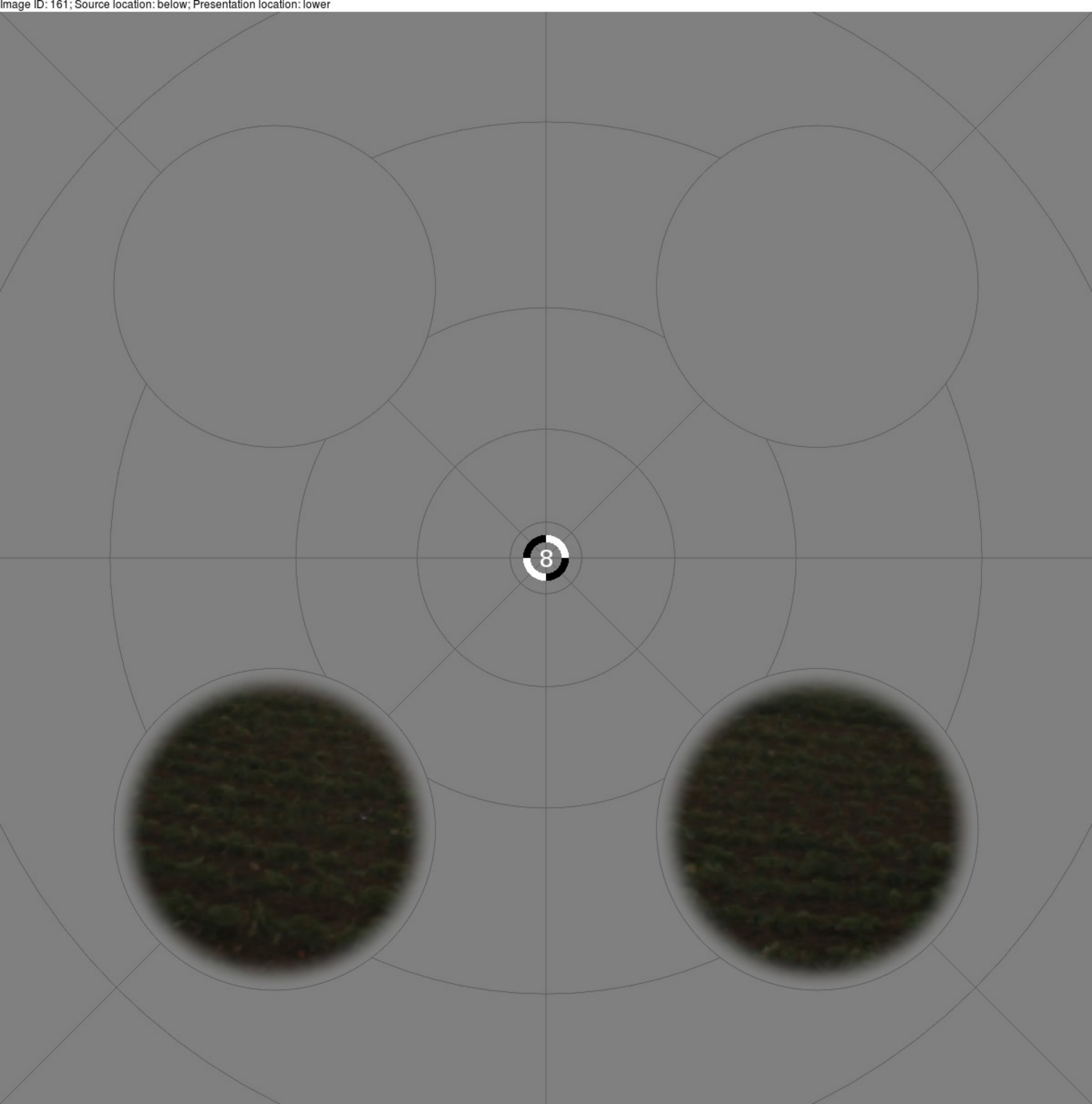

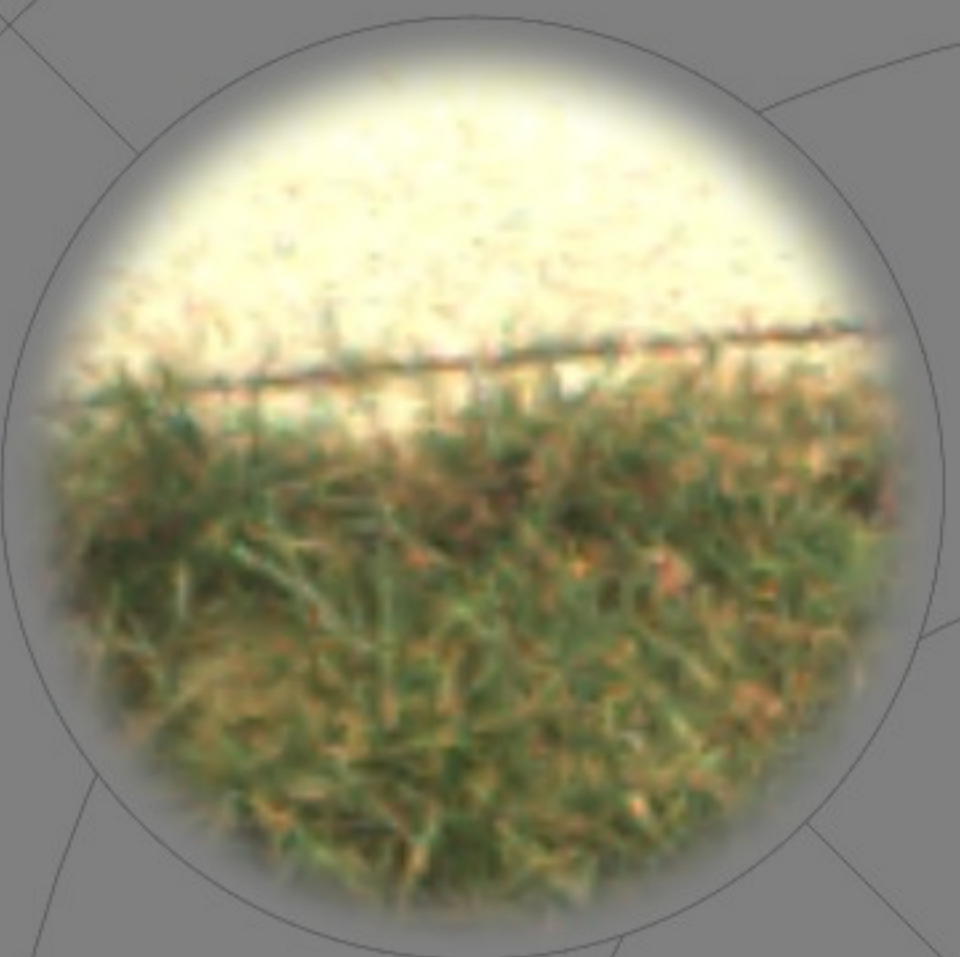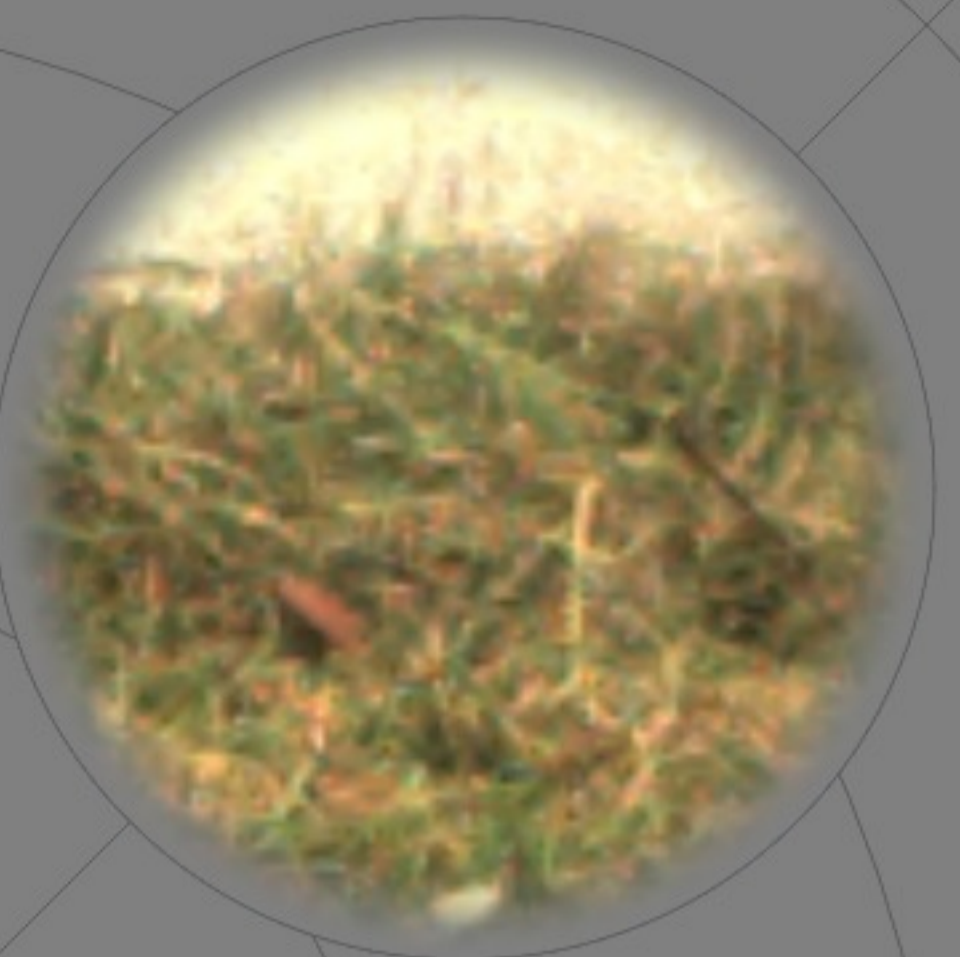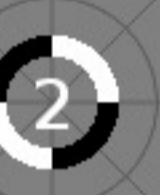

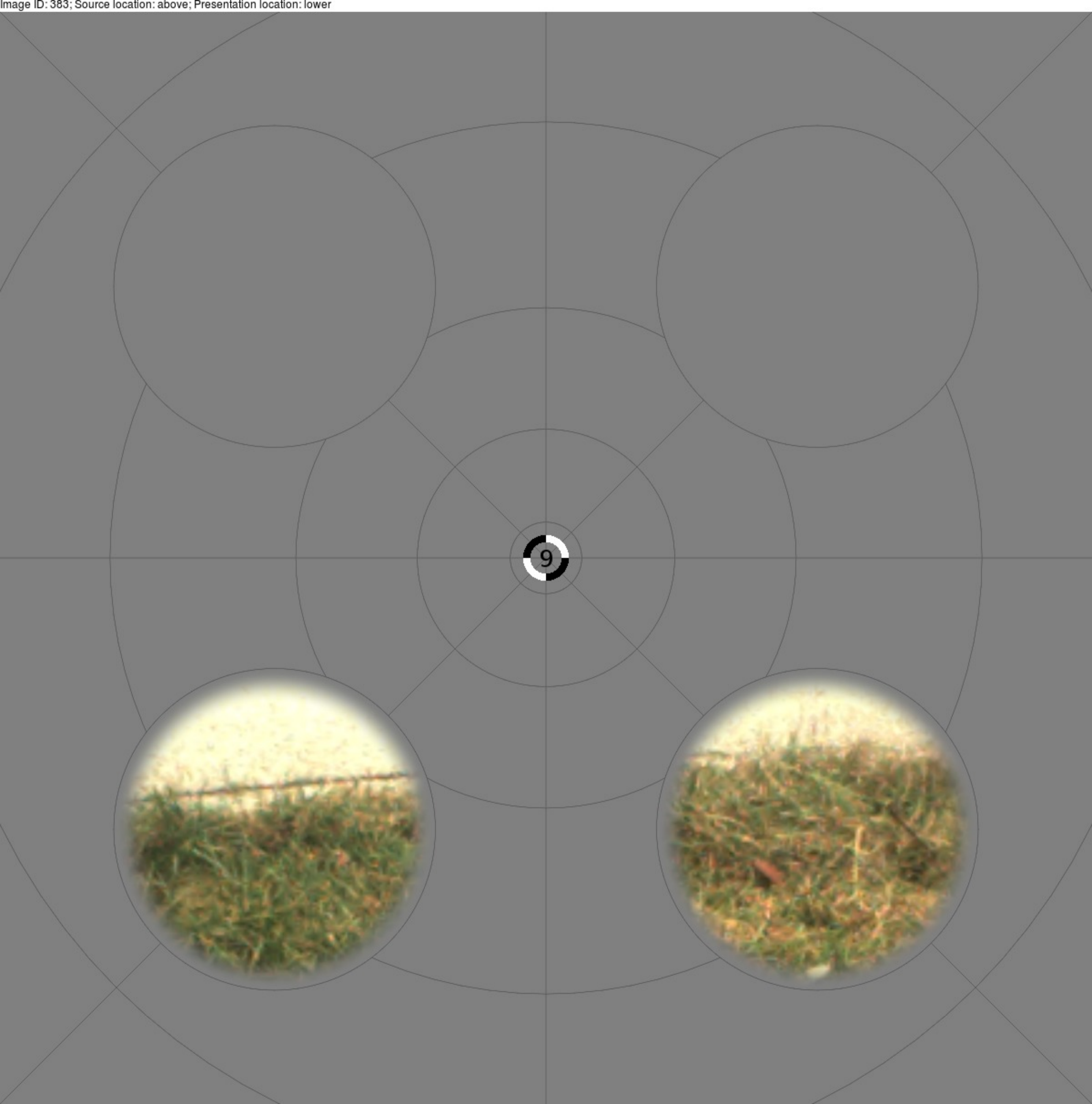

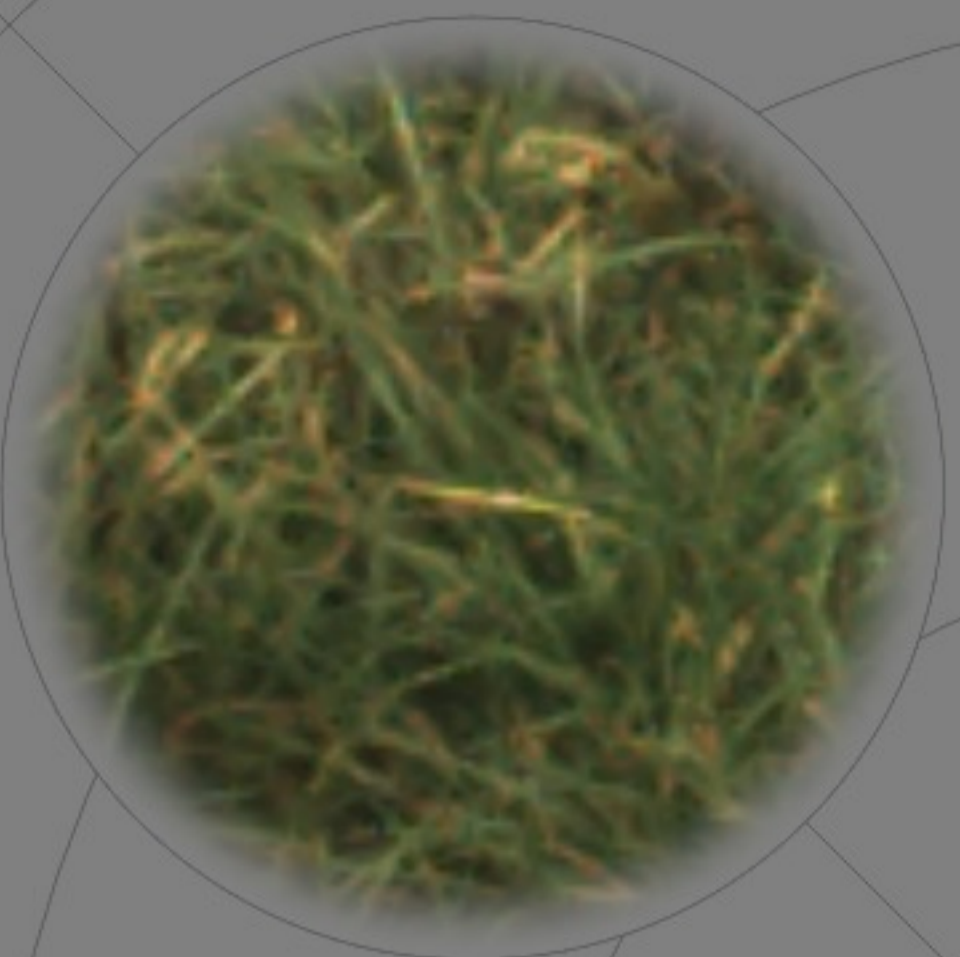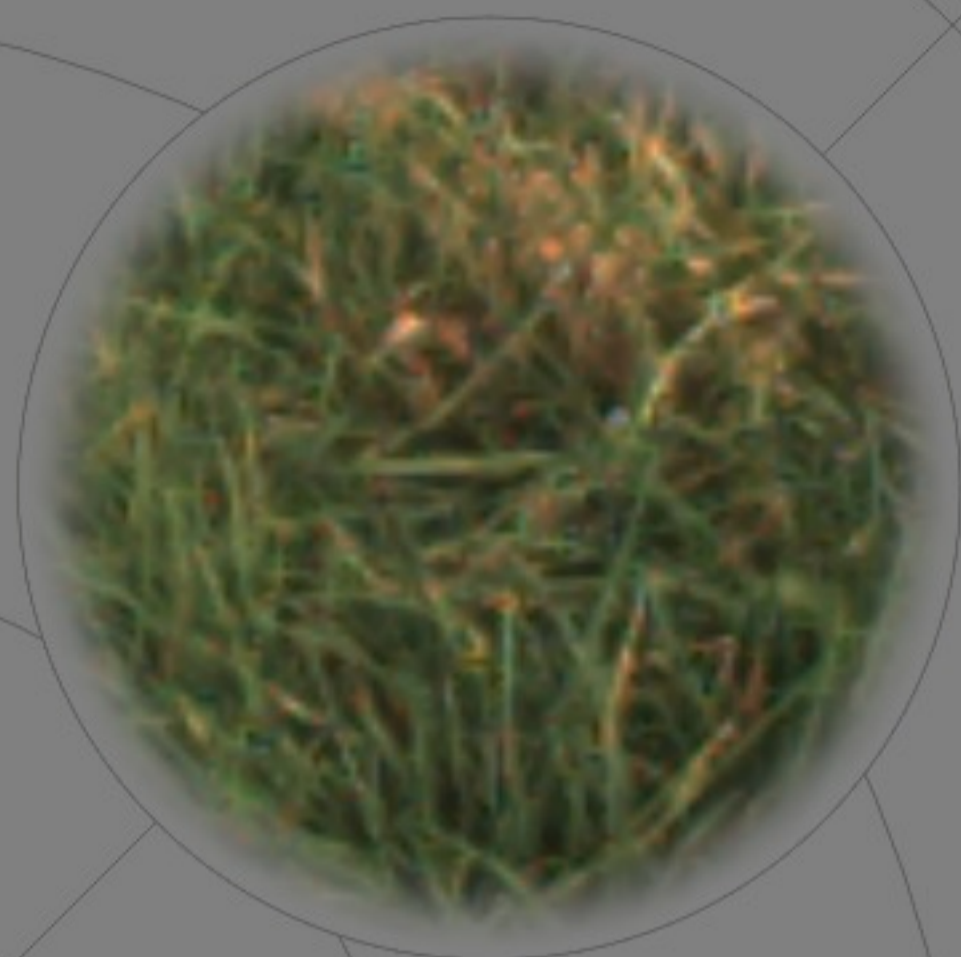

1

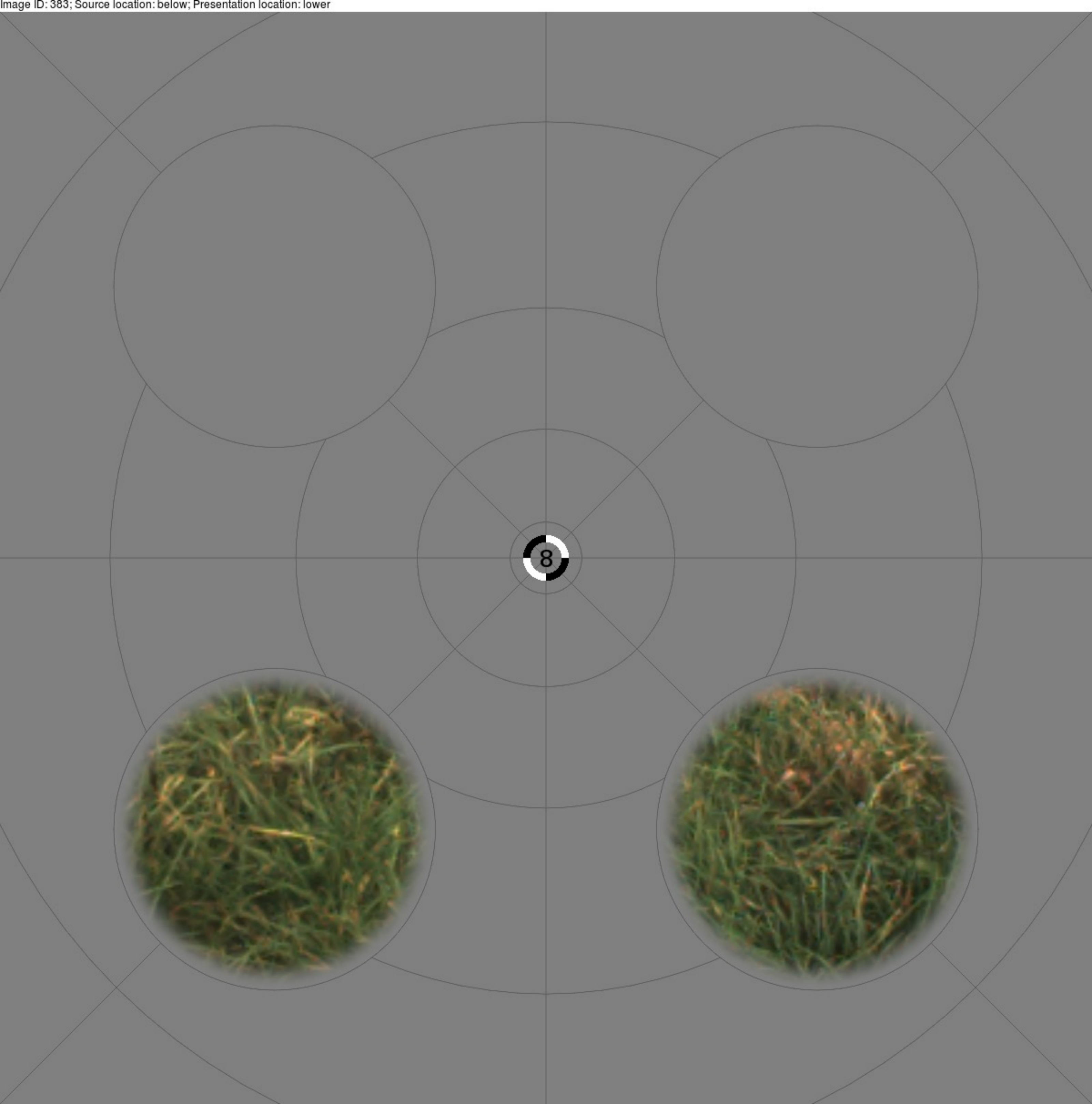

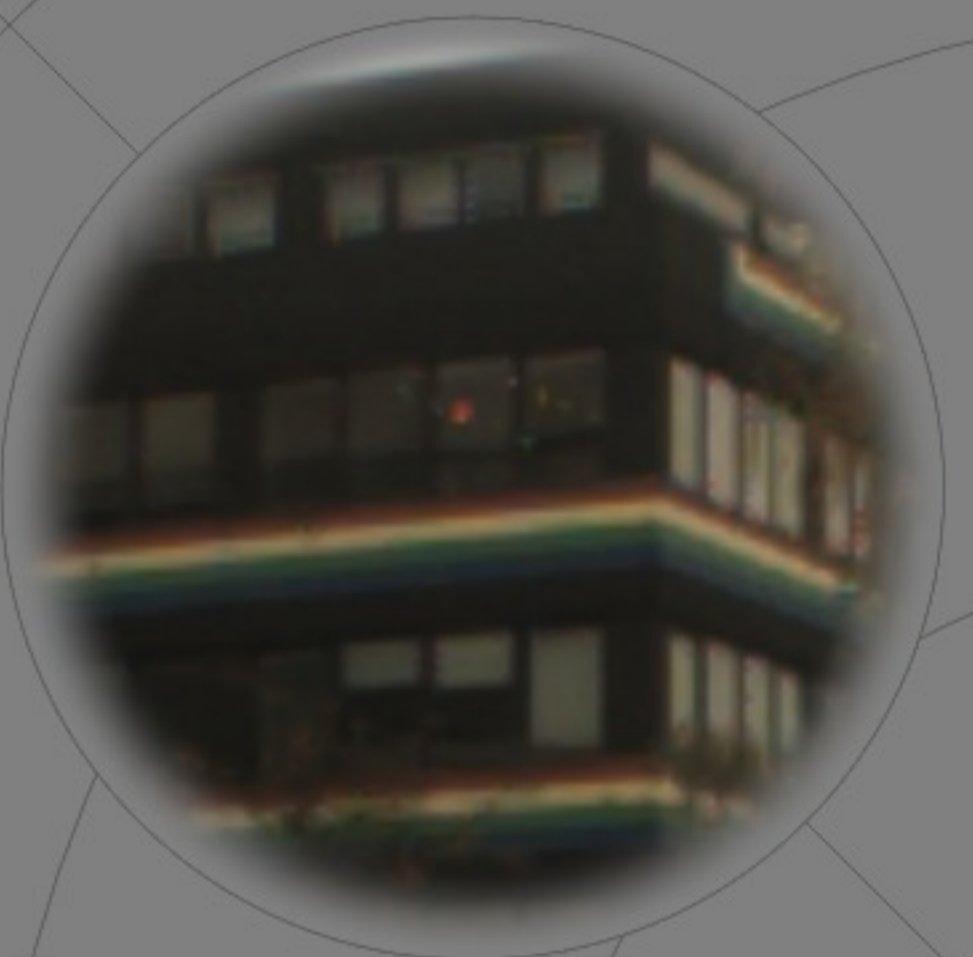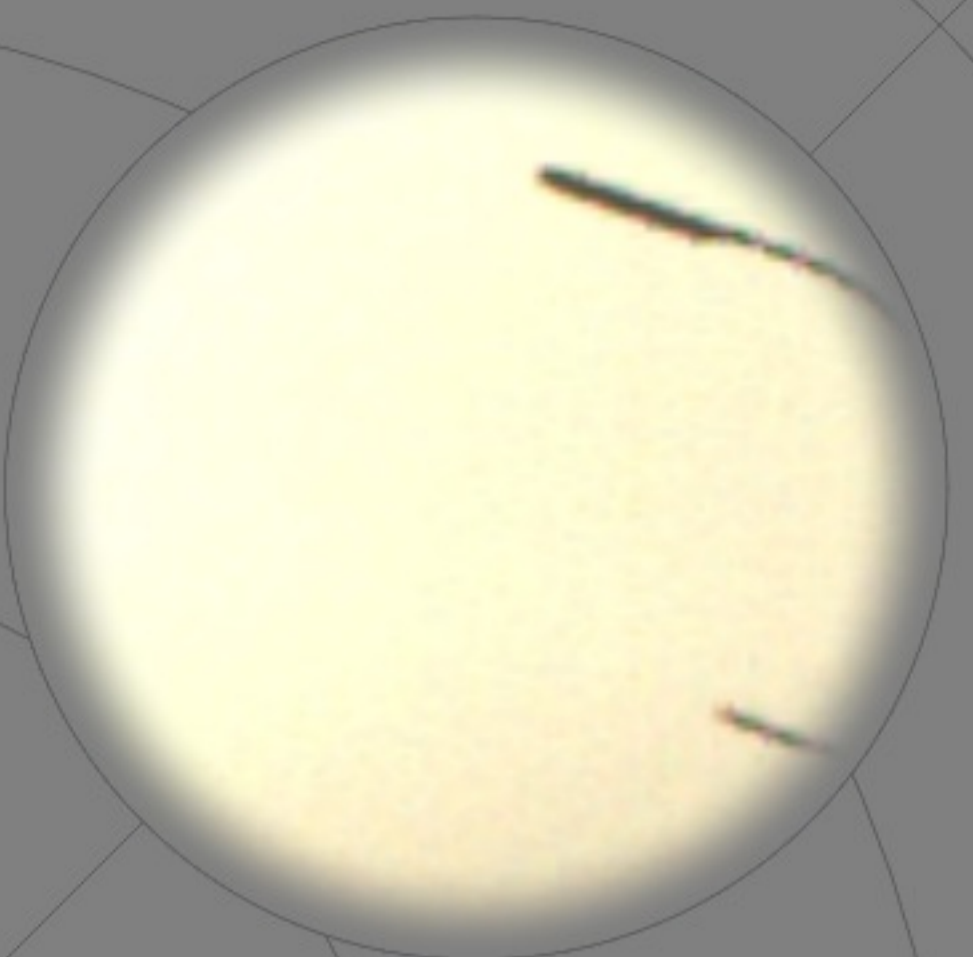

1

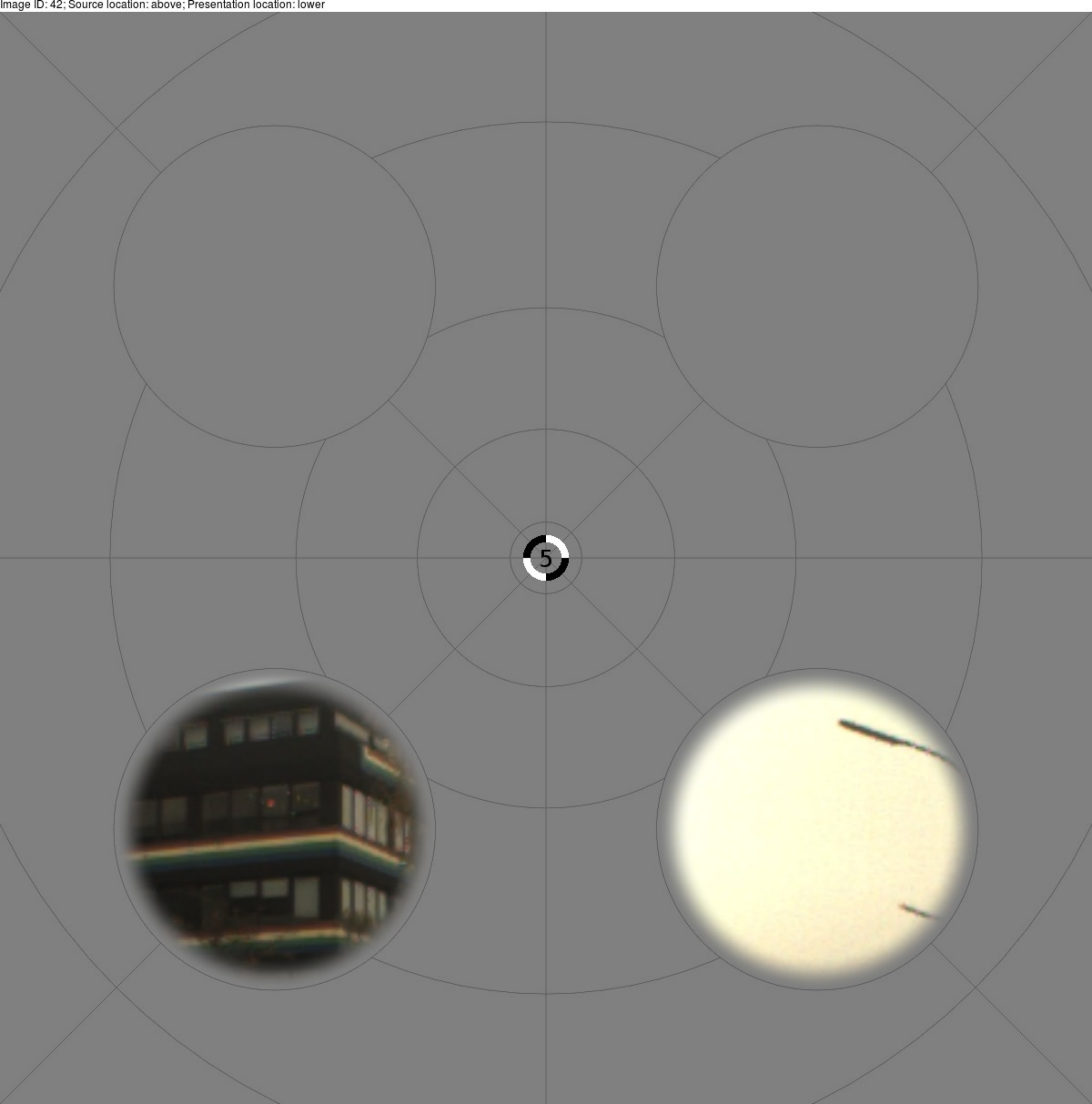

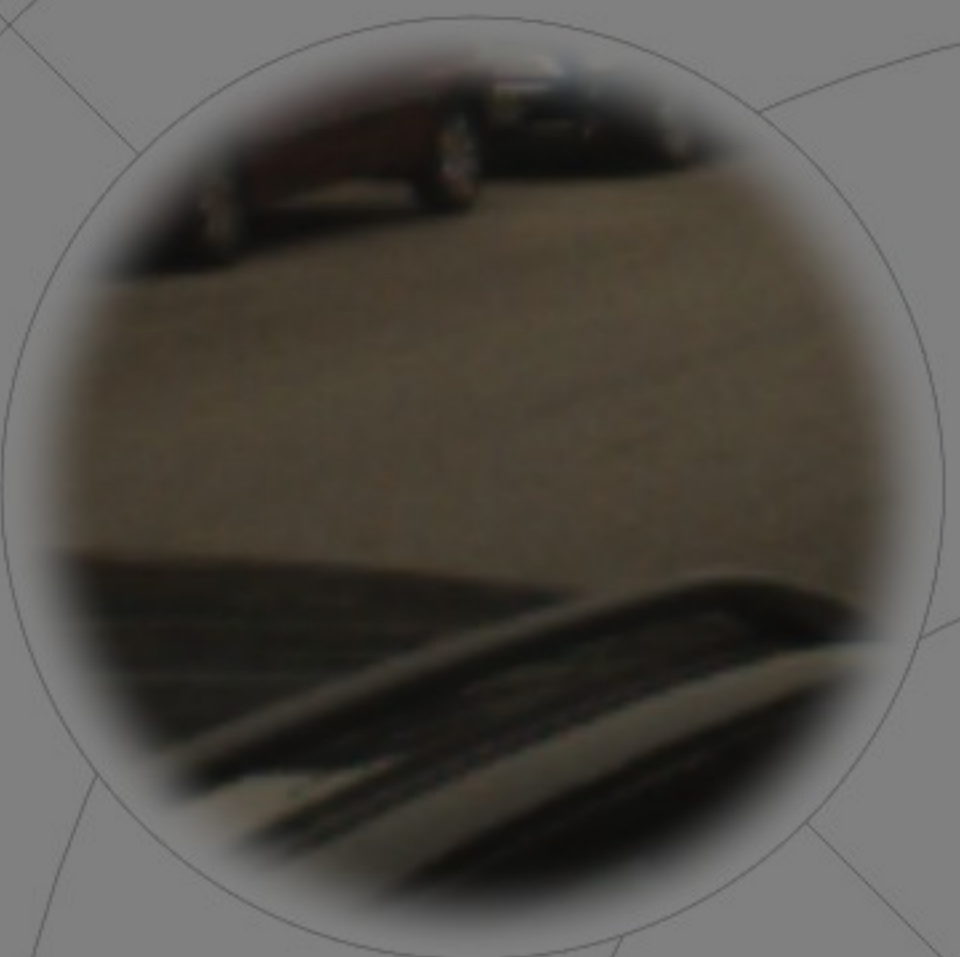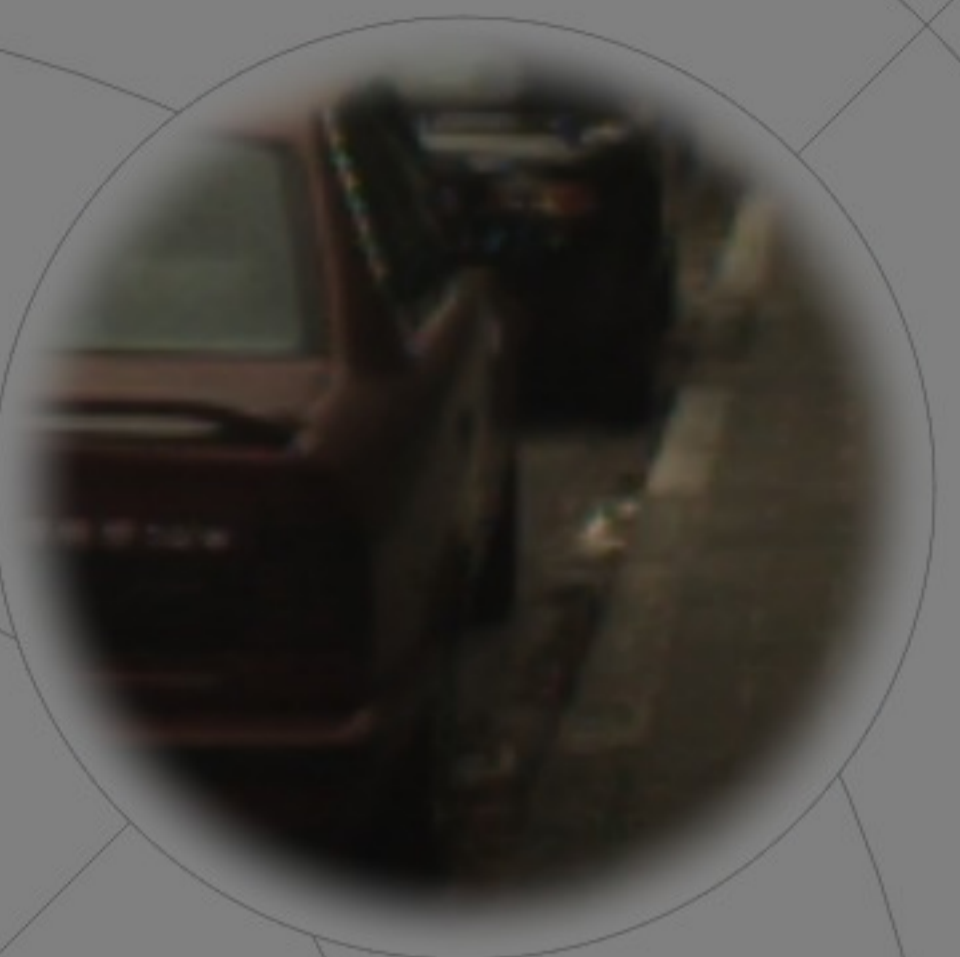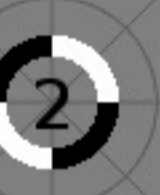

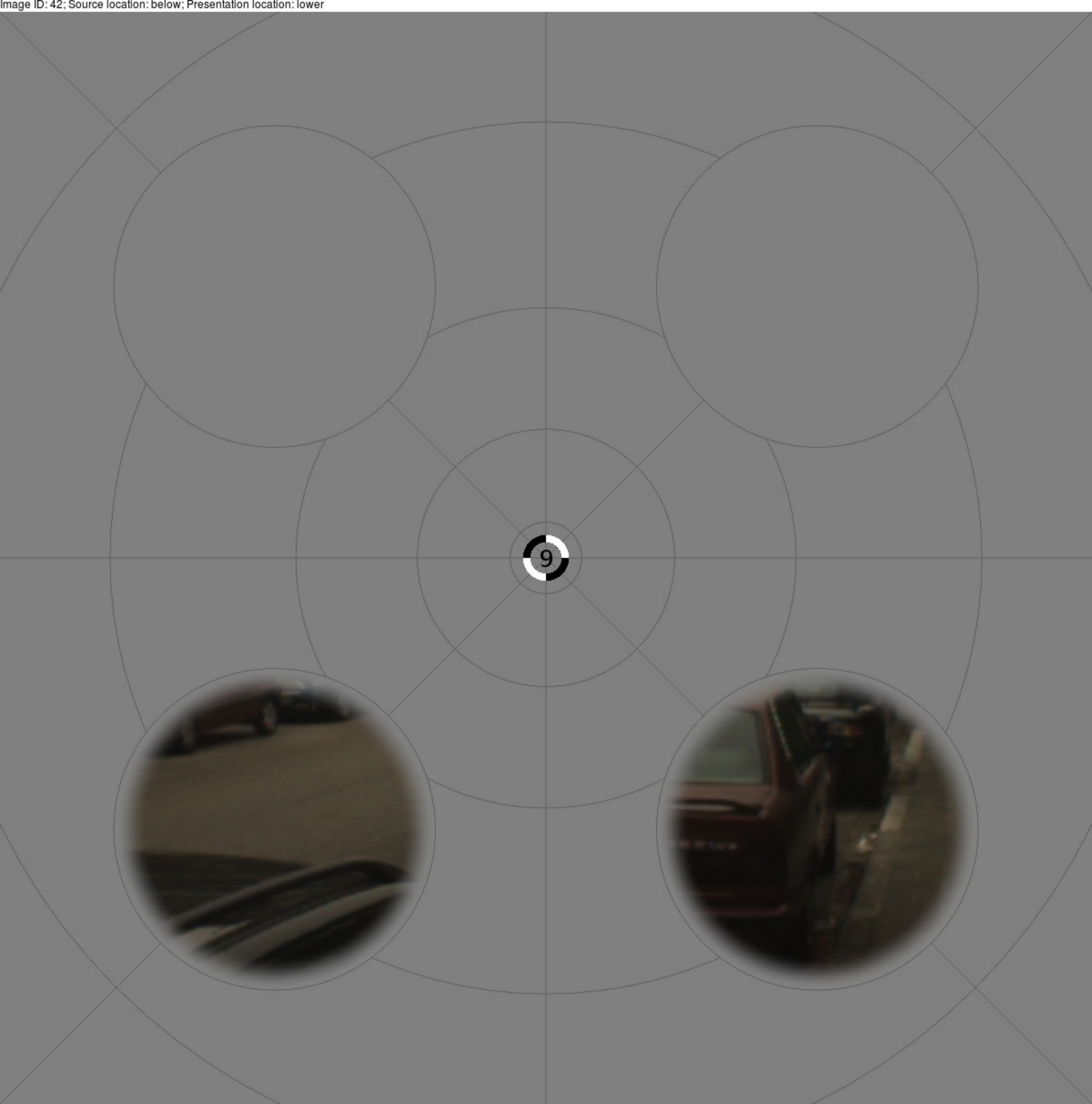

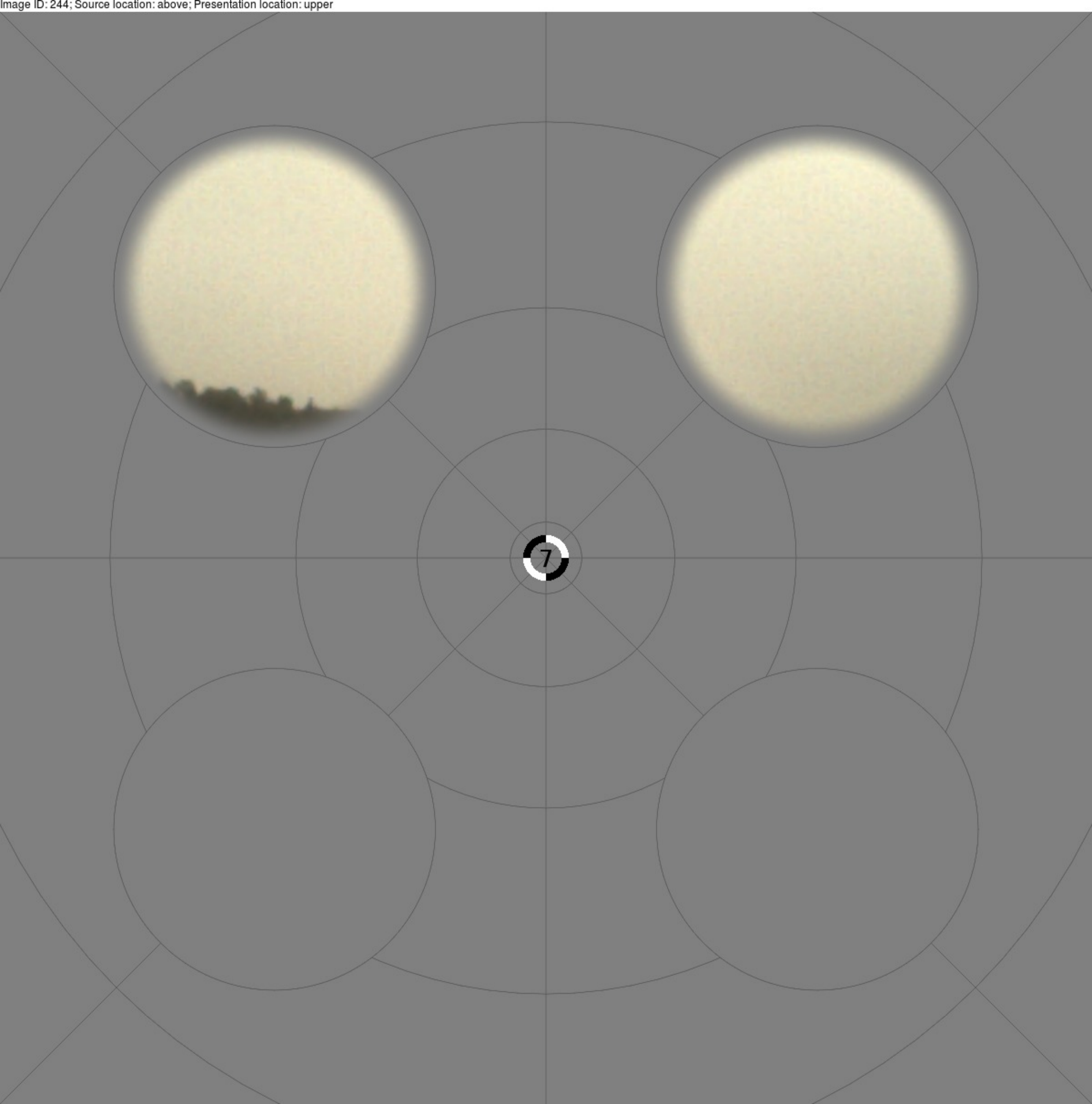

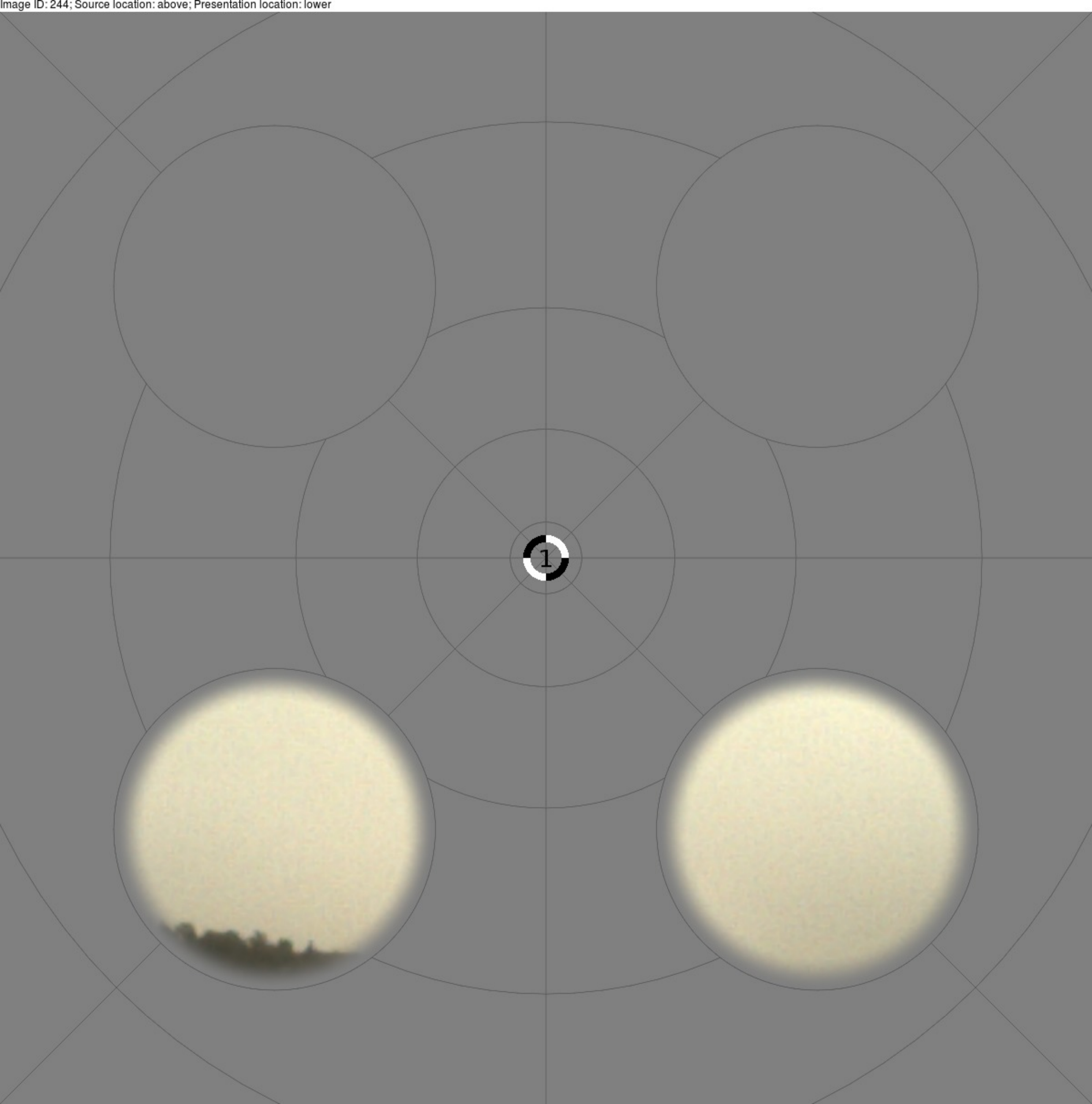

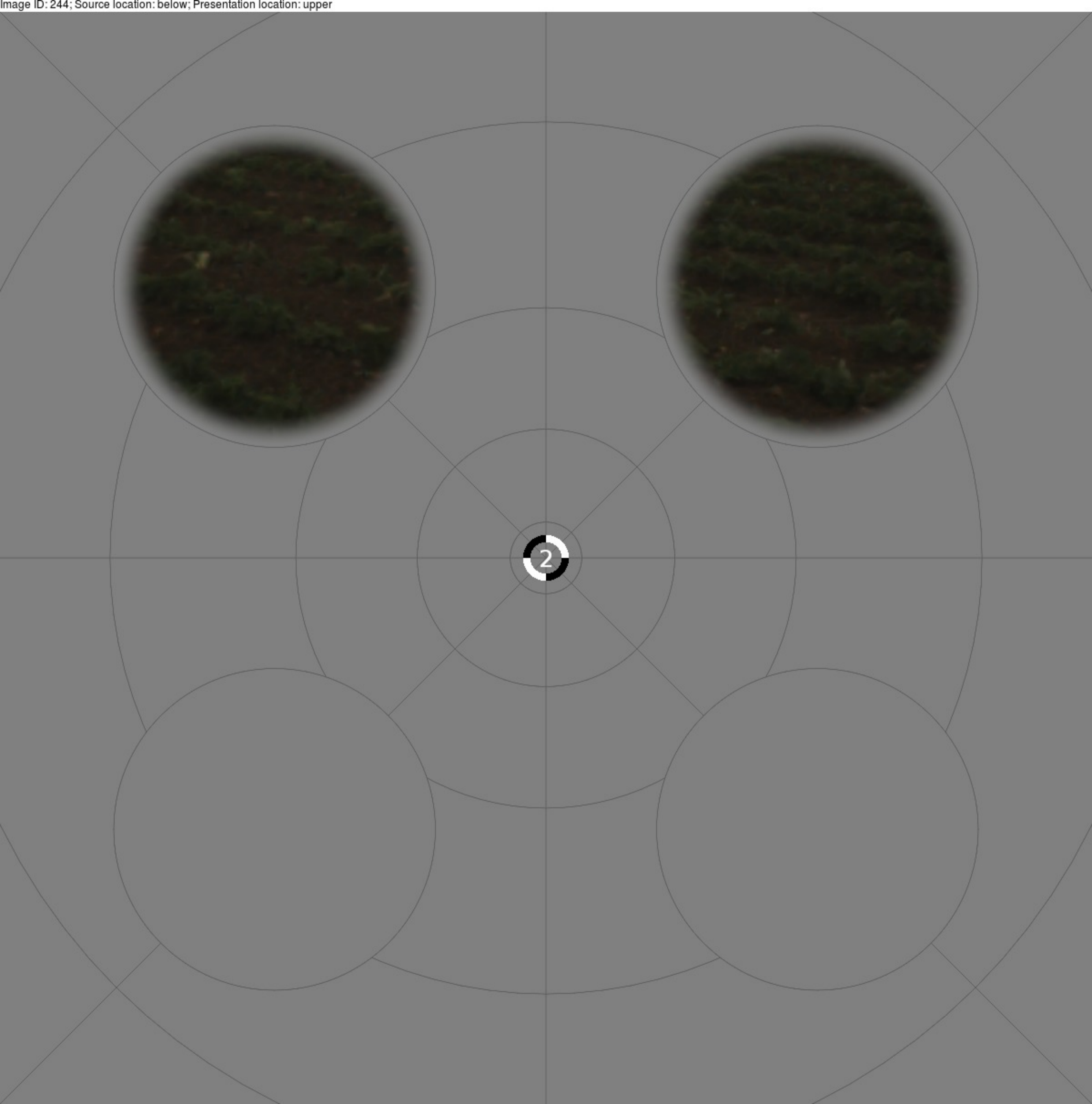

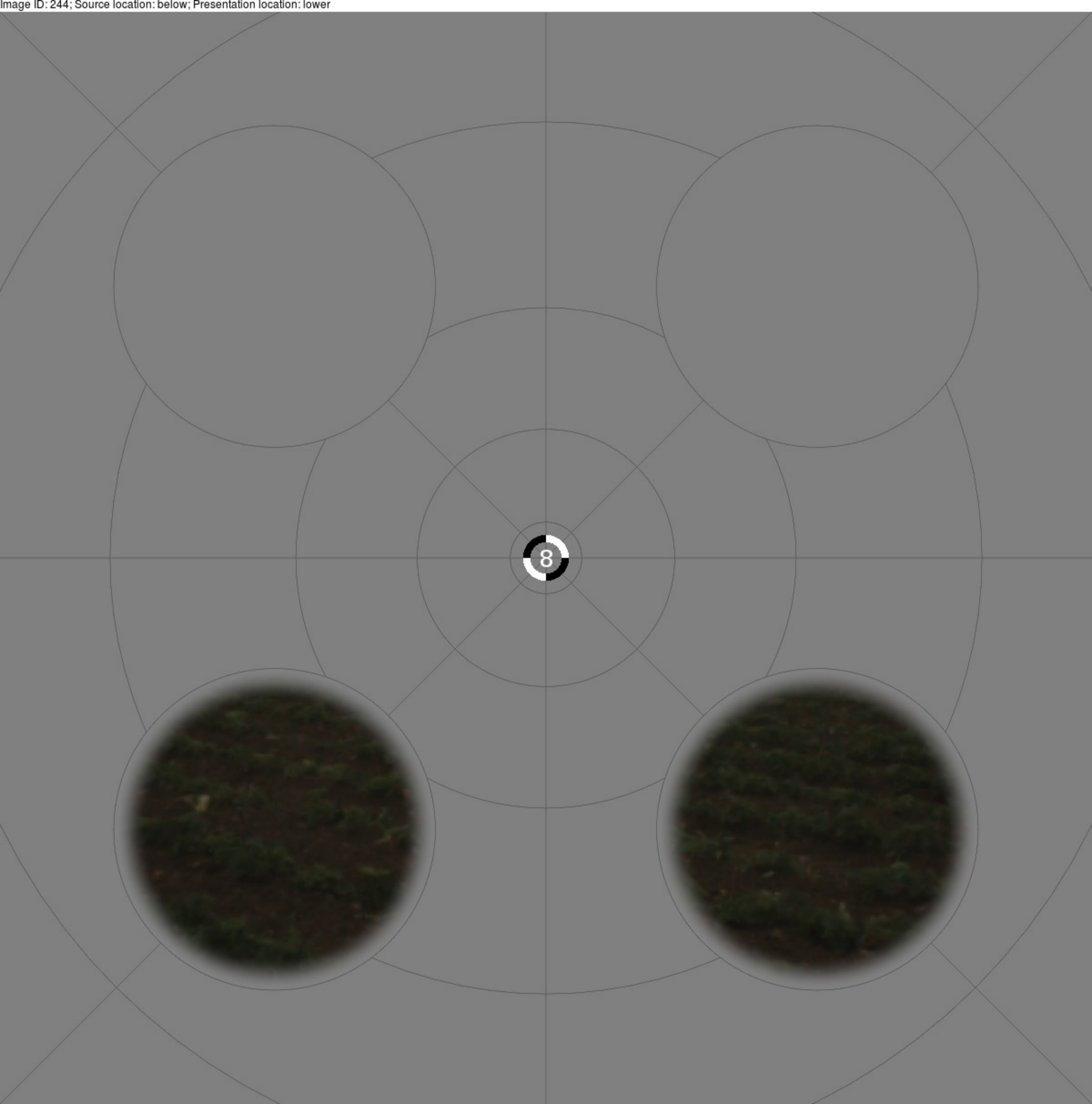

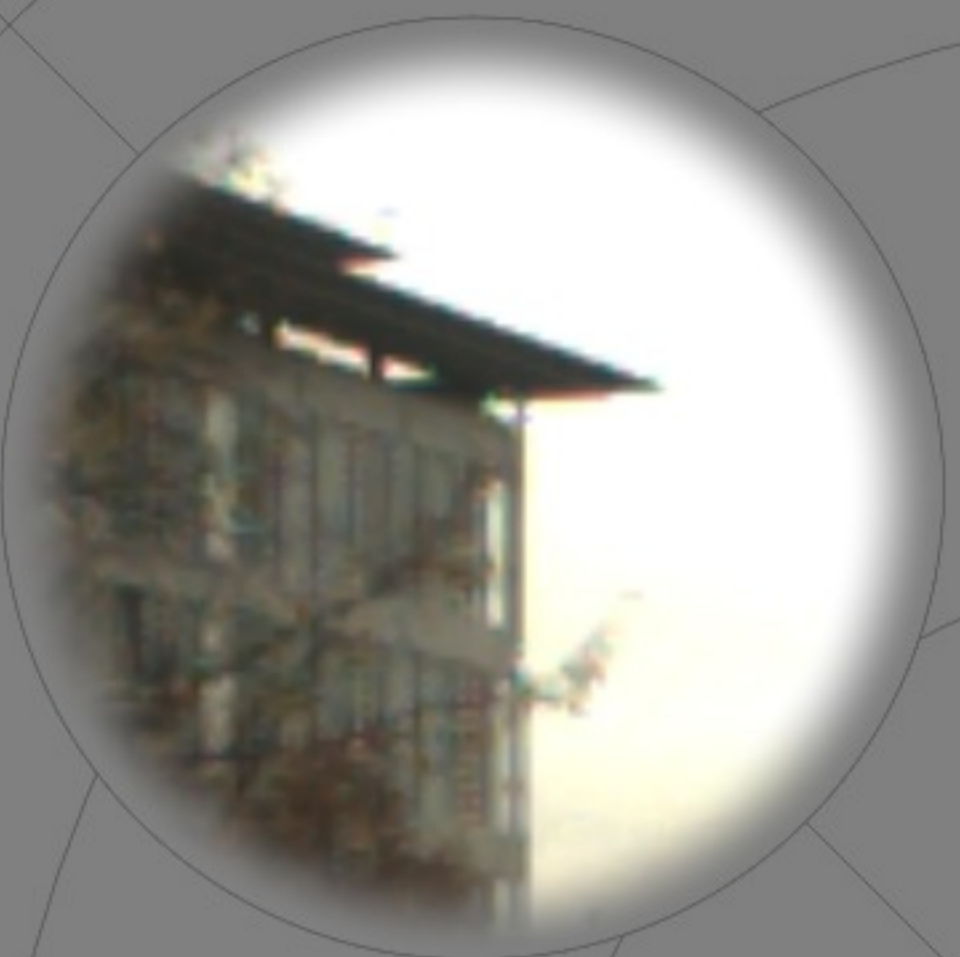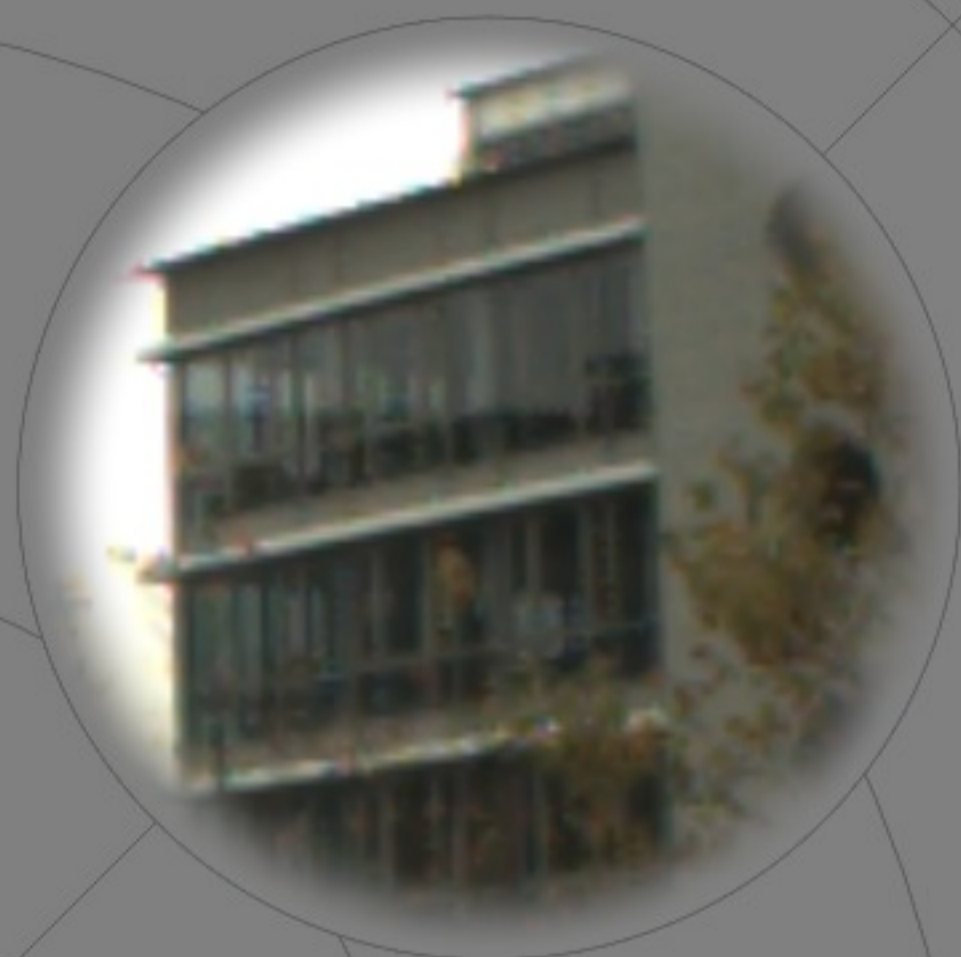

6

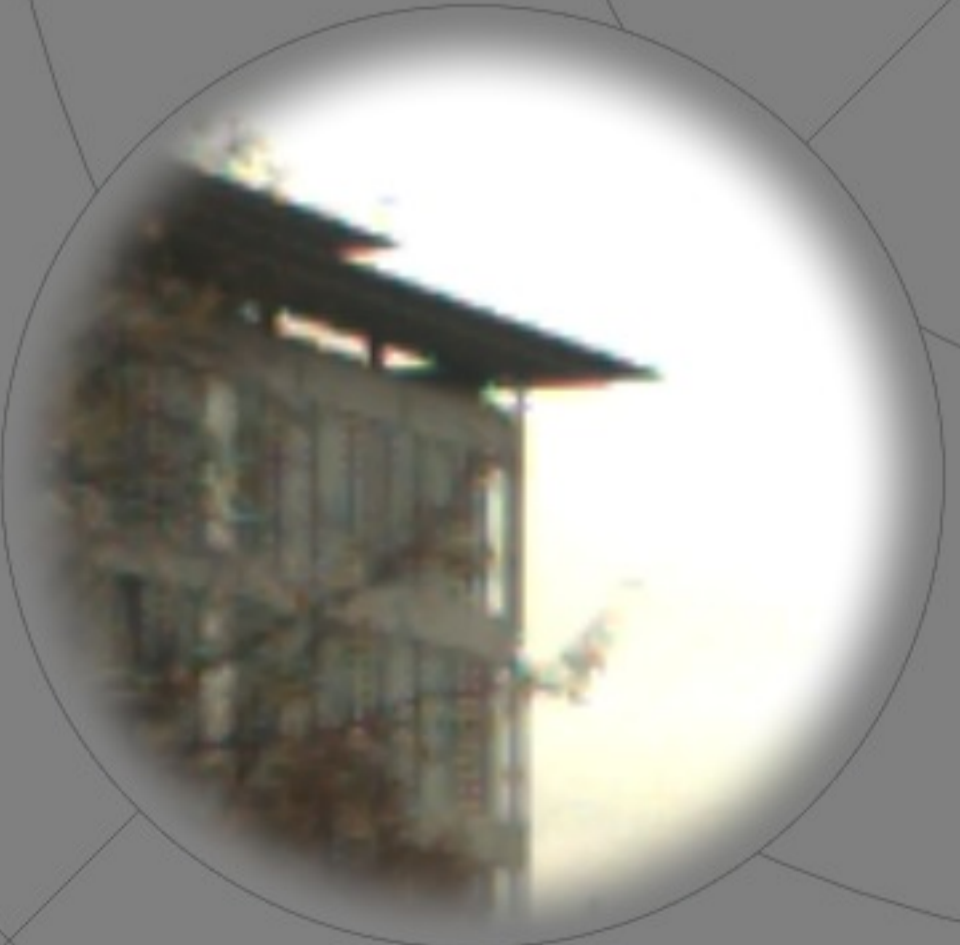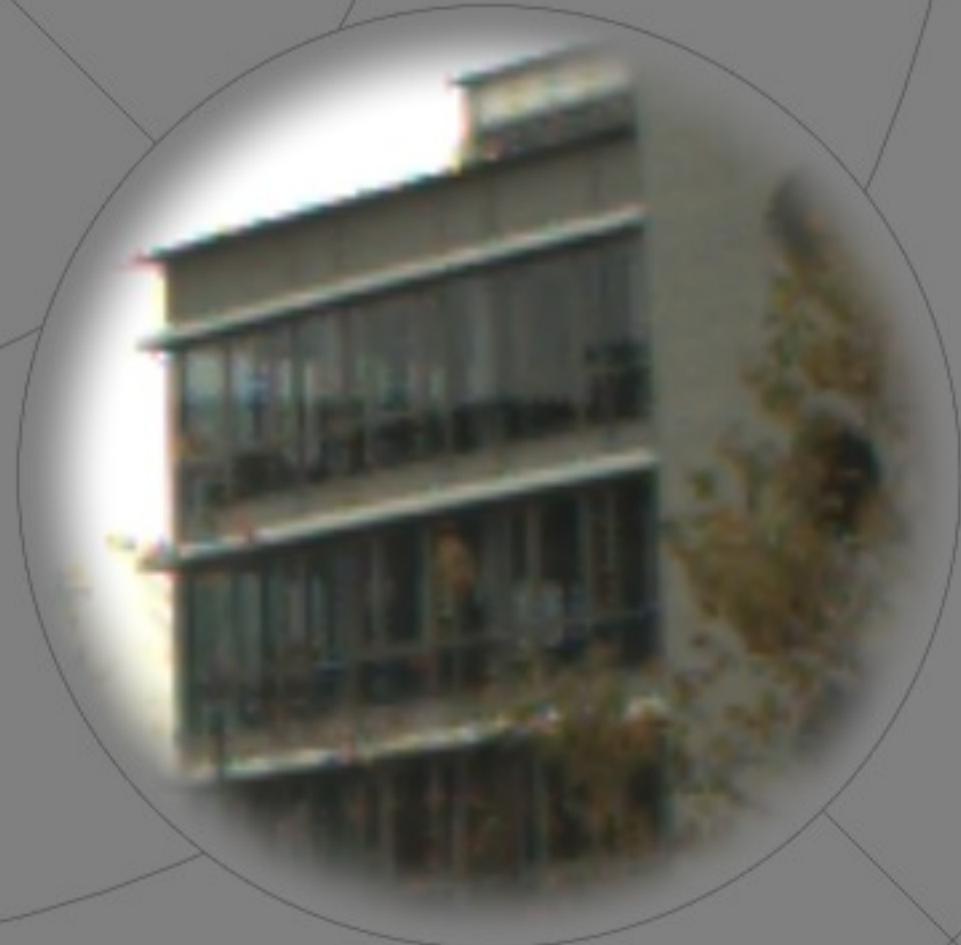

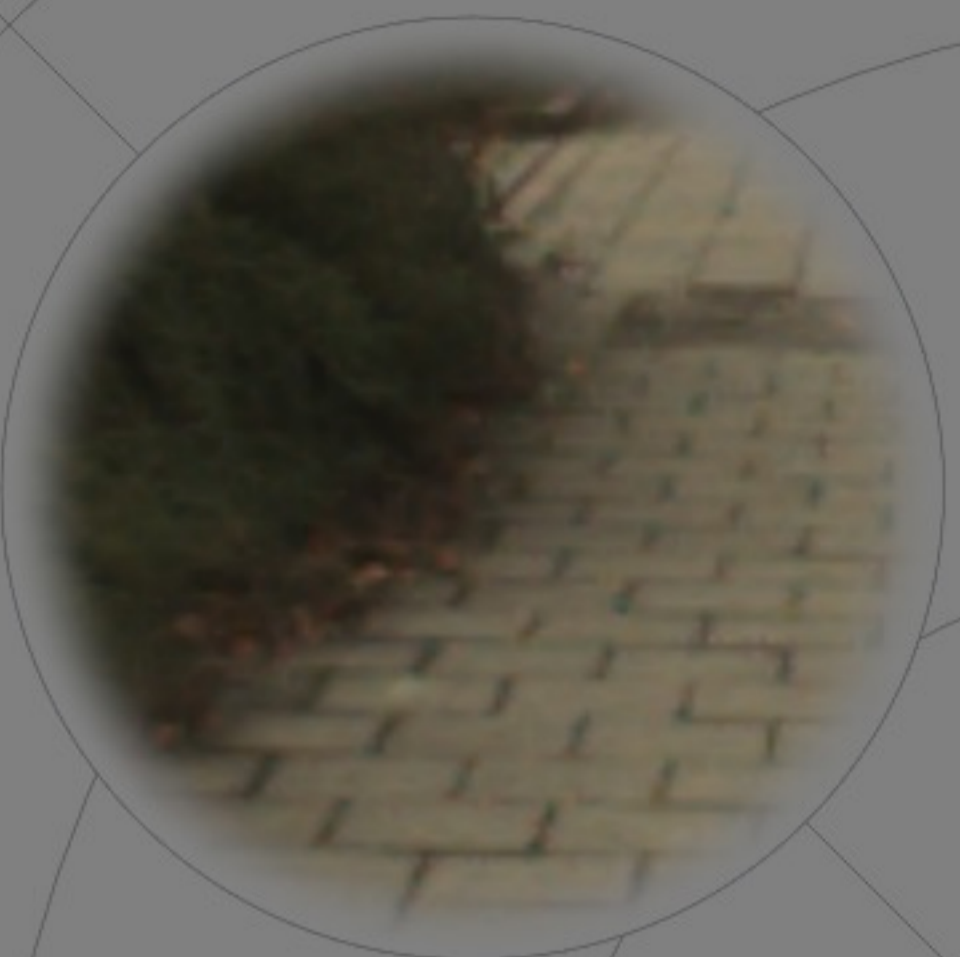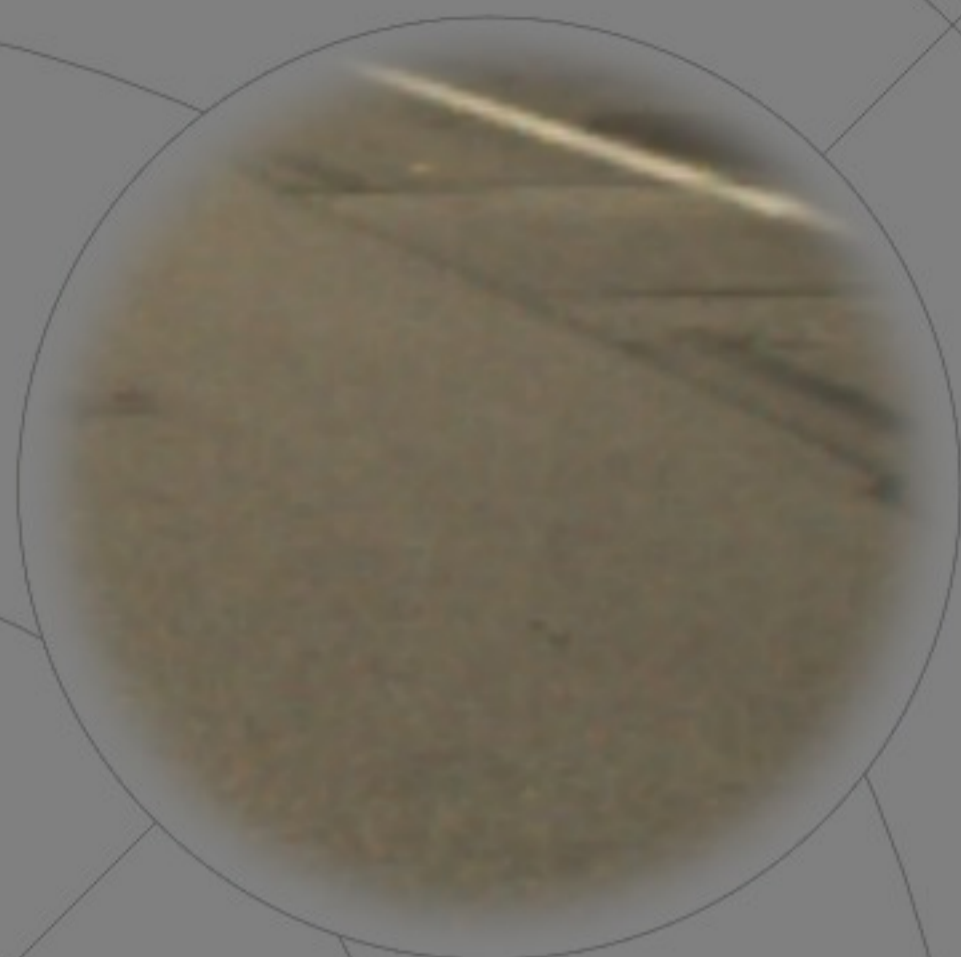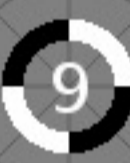

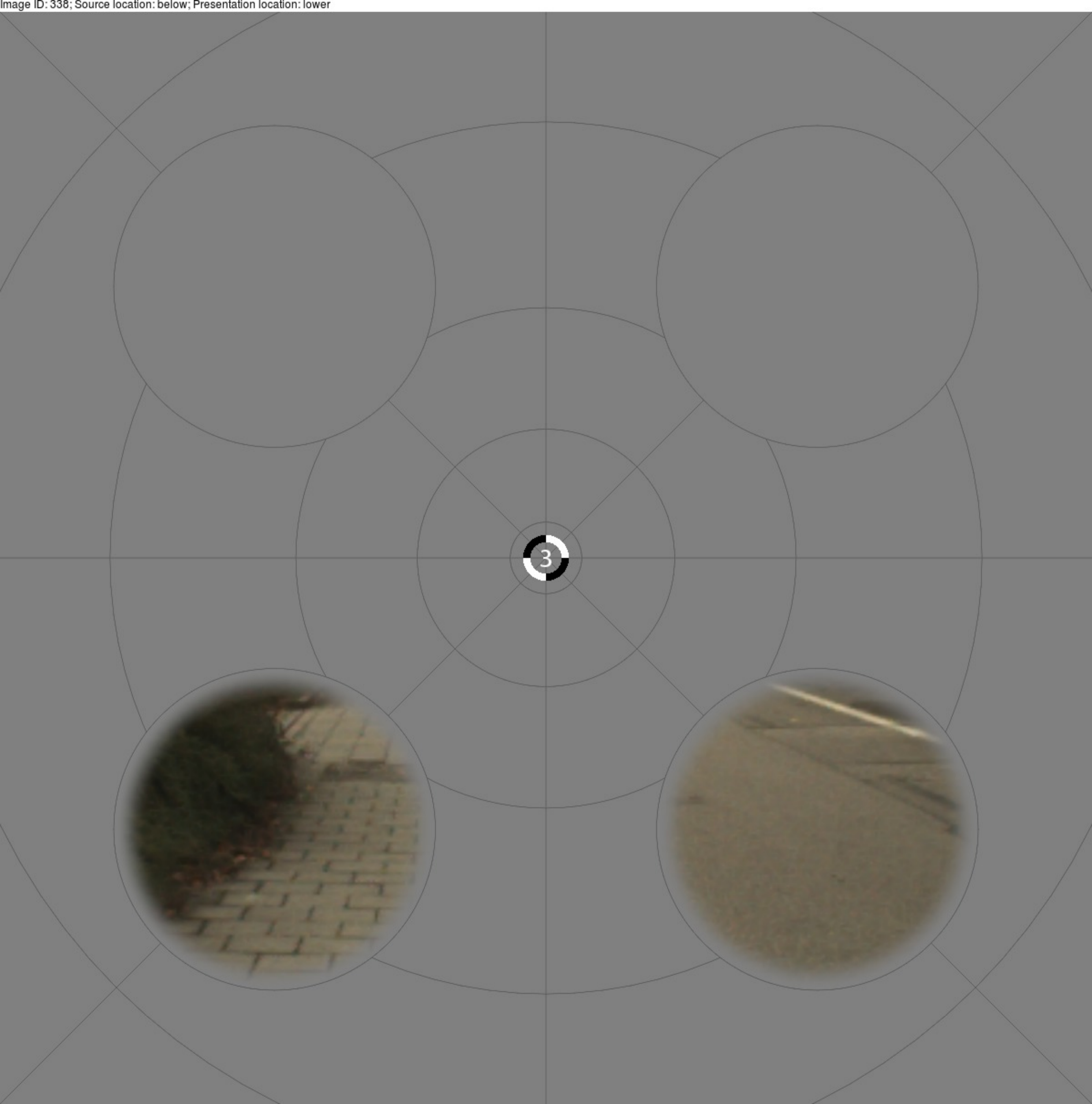

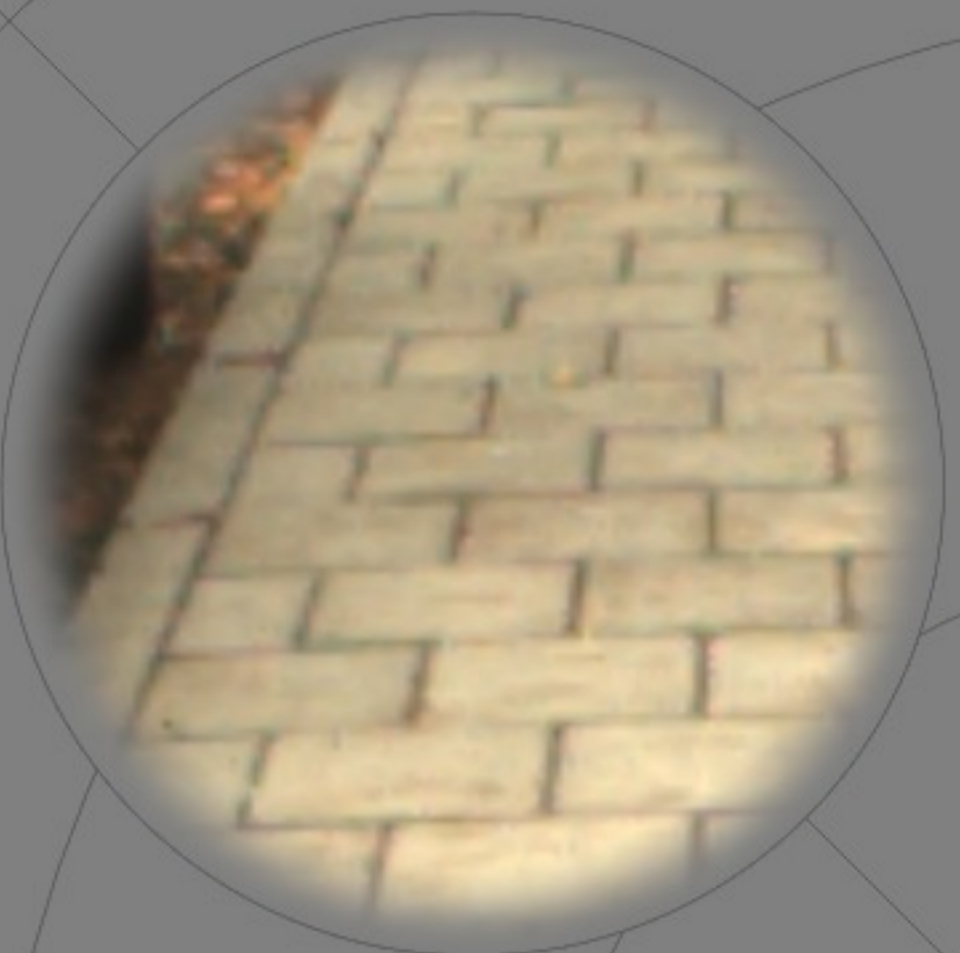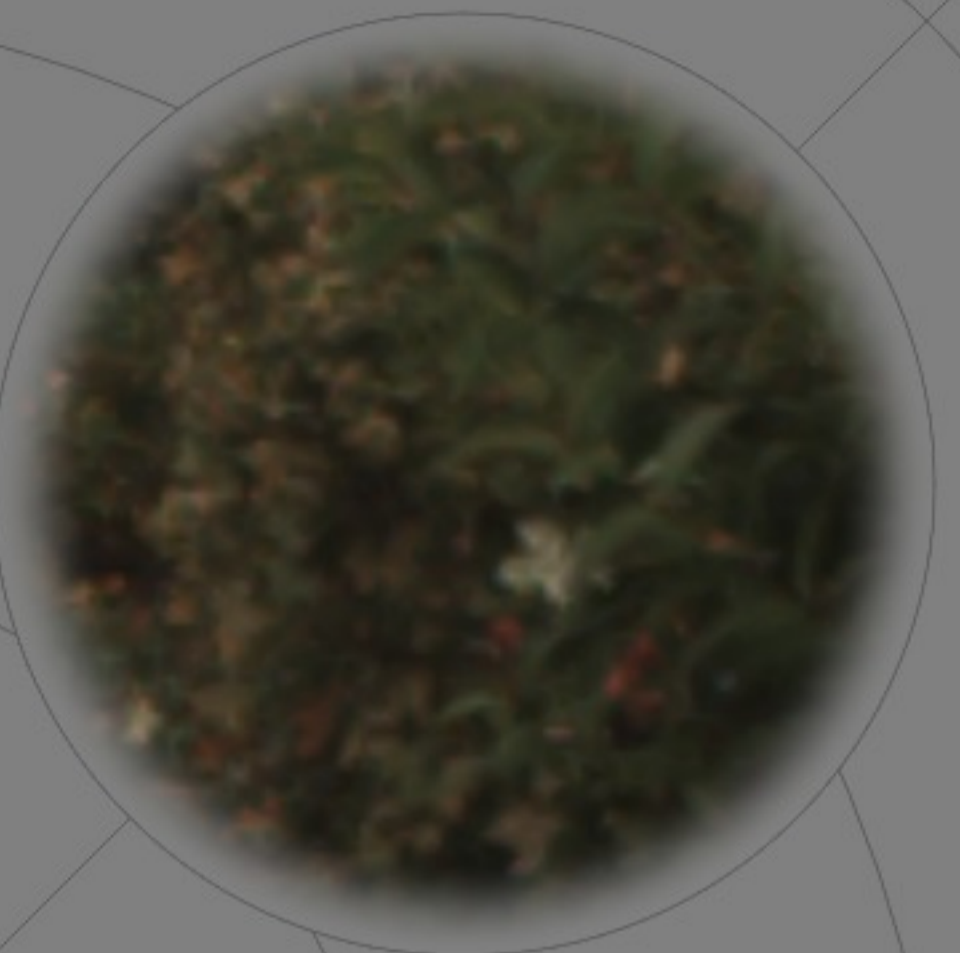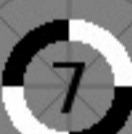

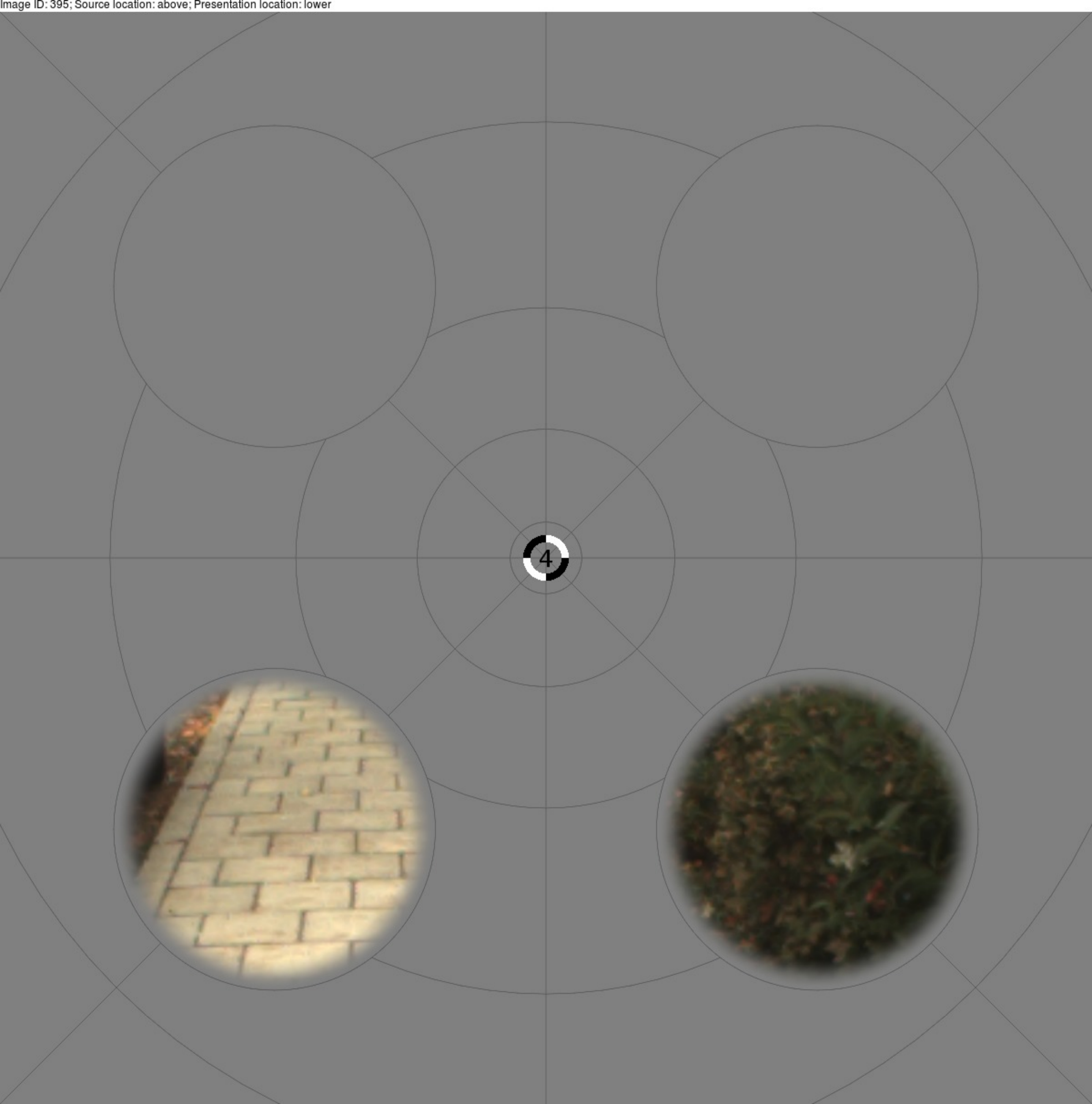

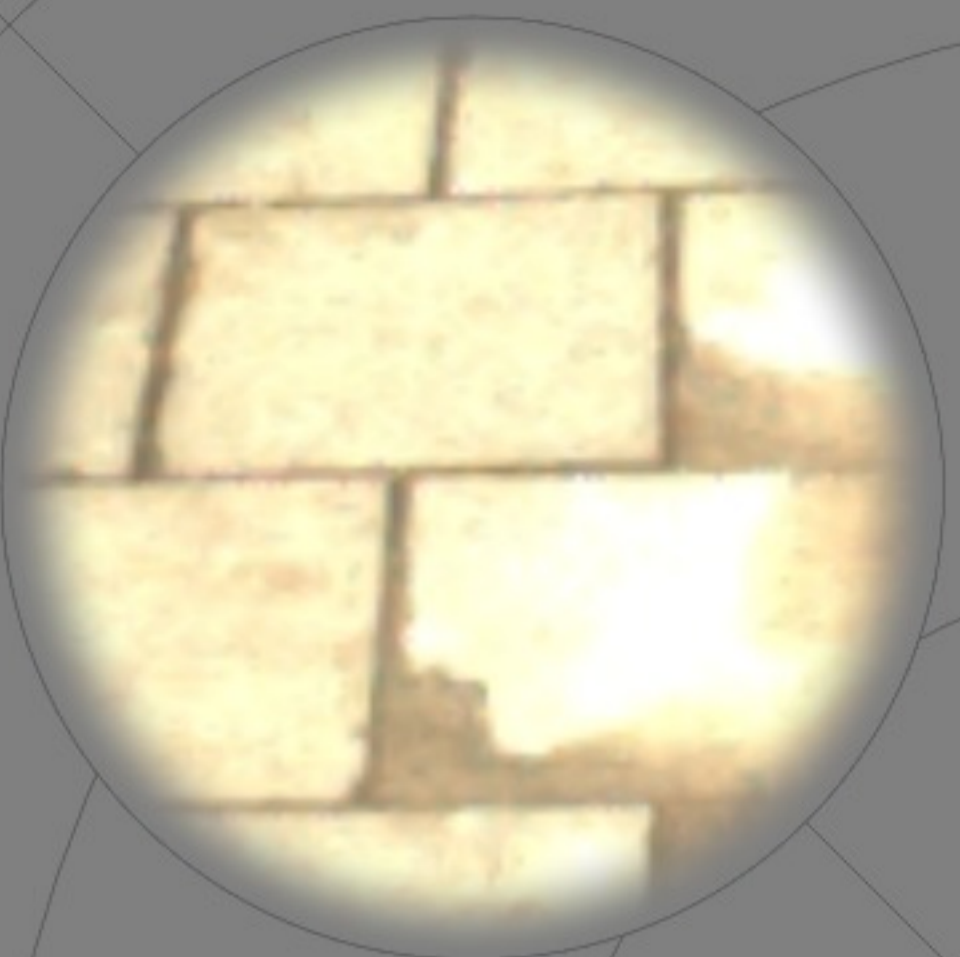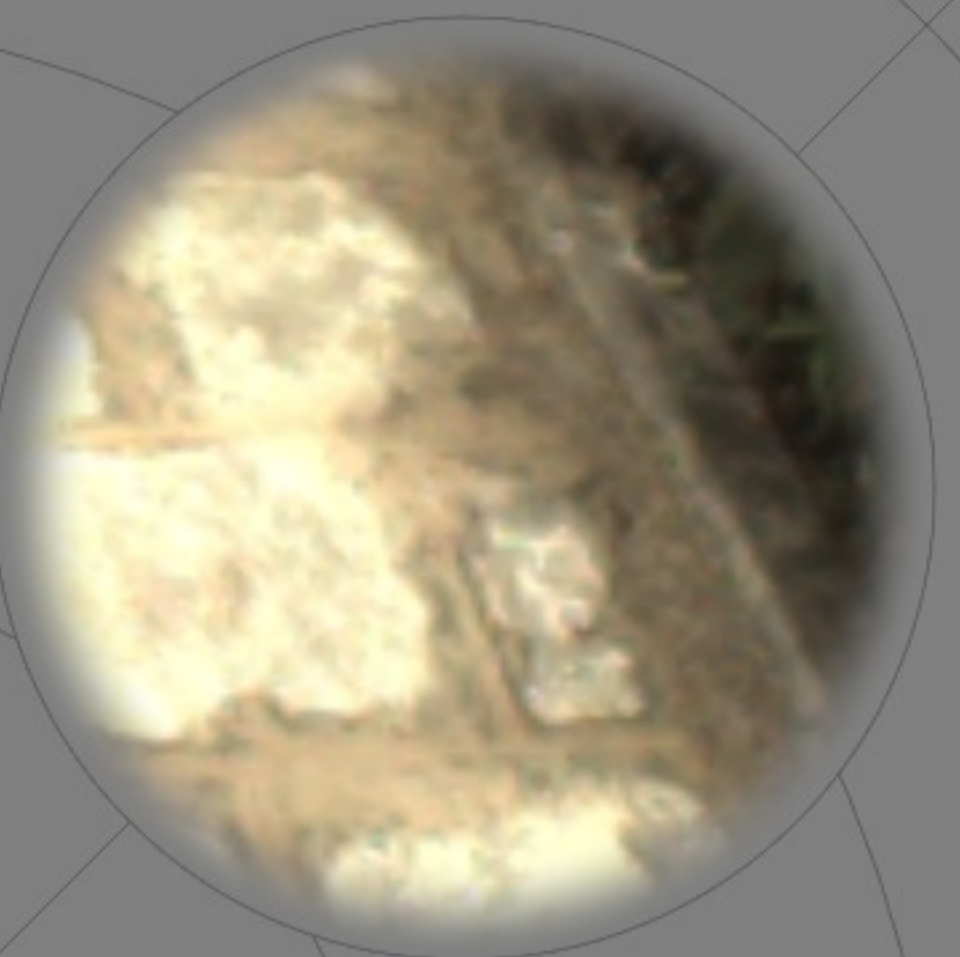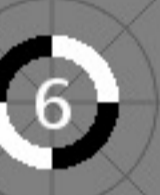

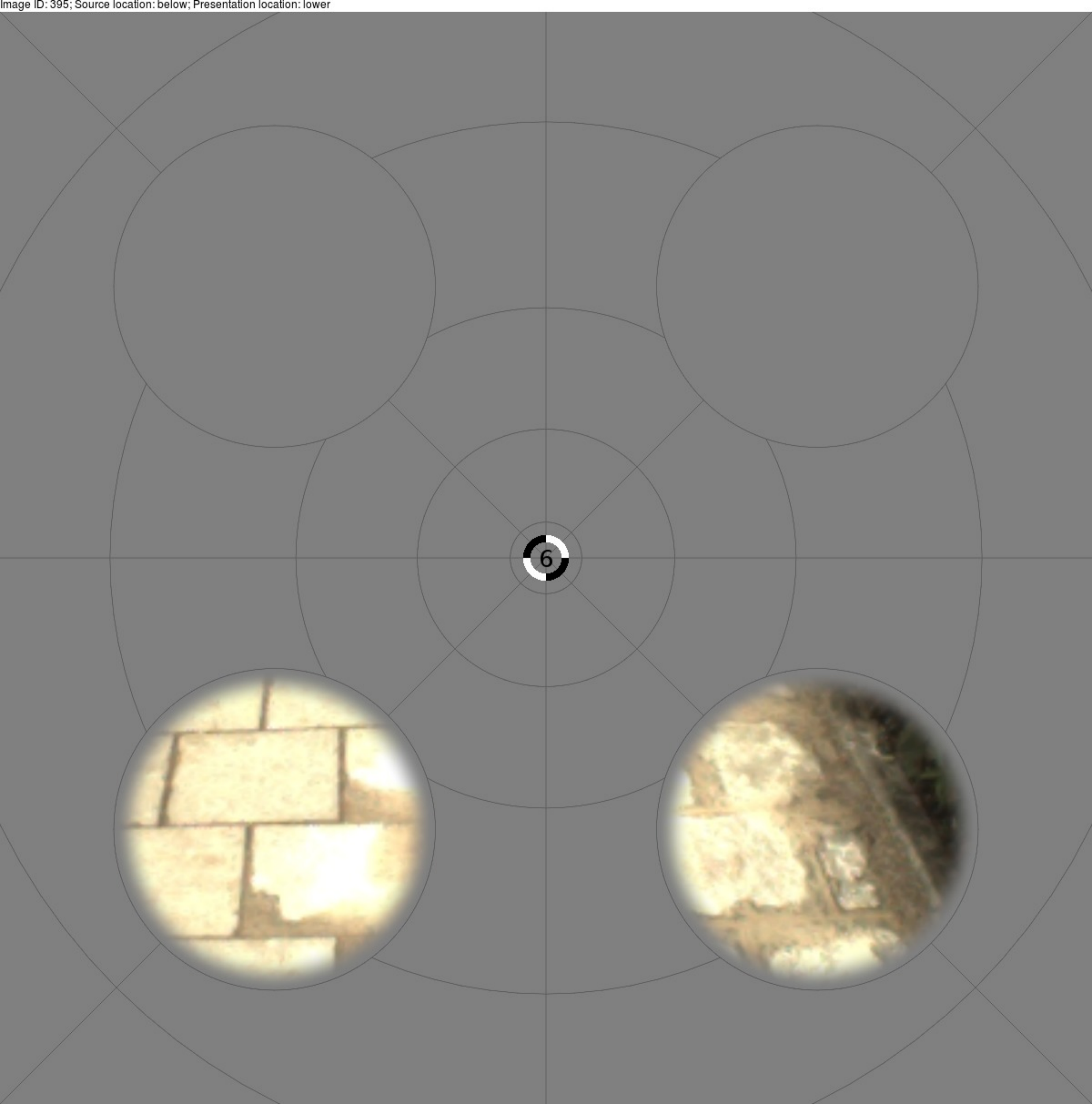

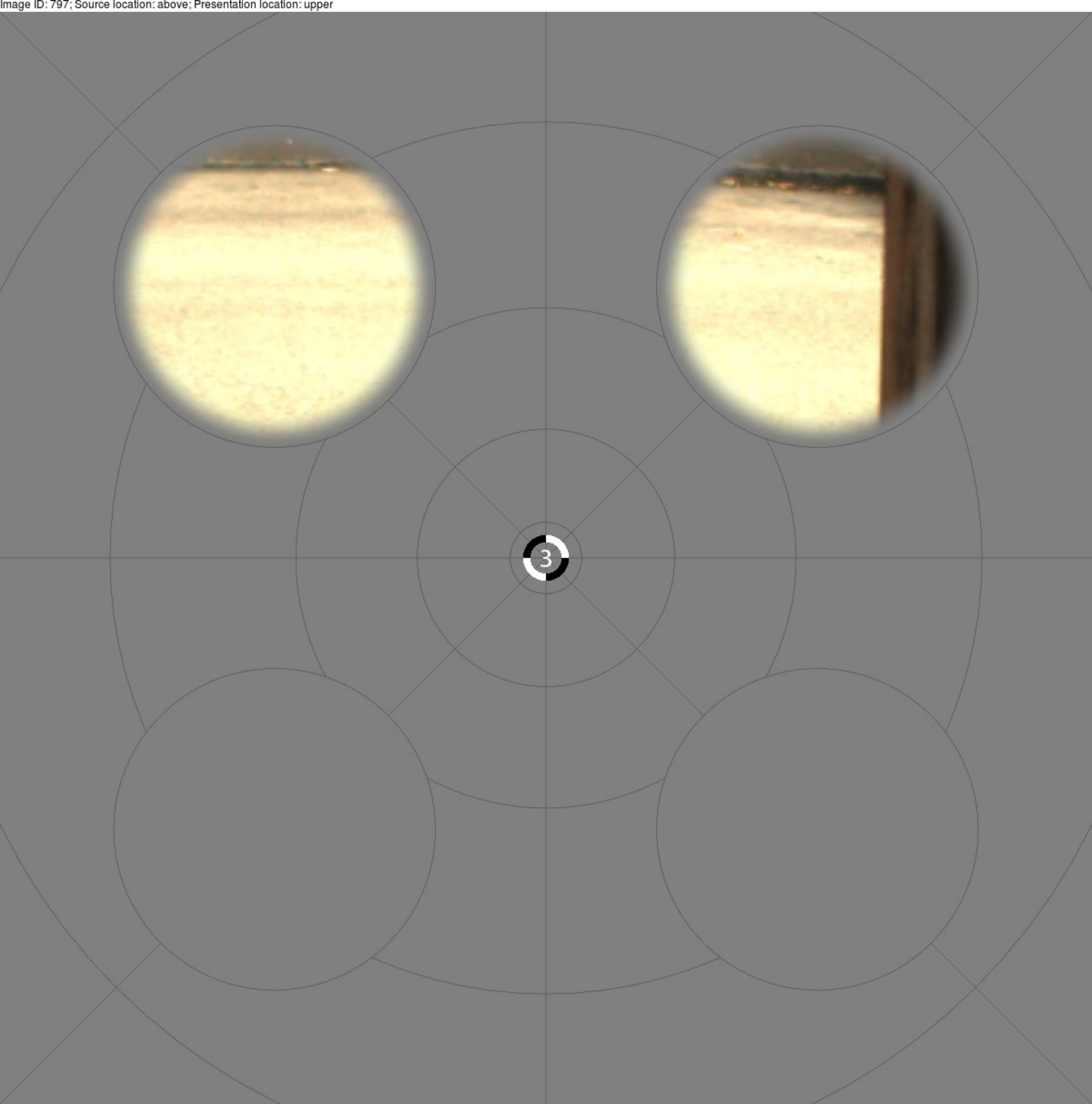

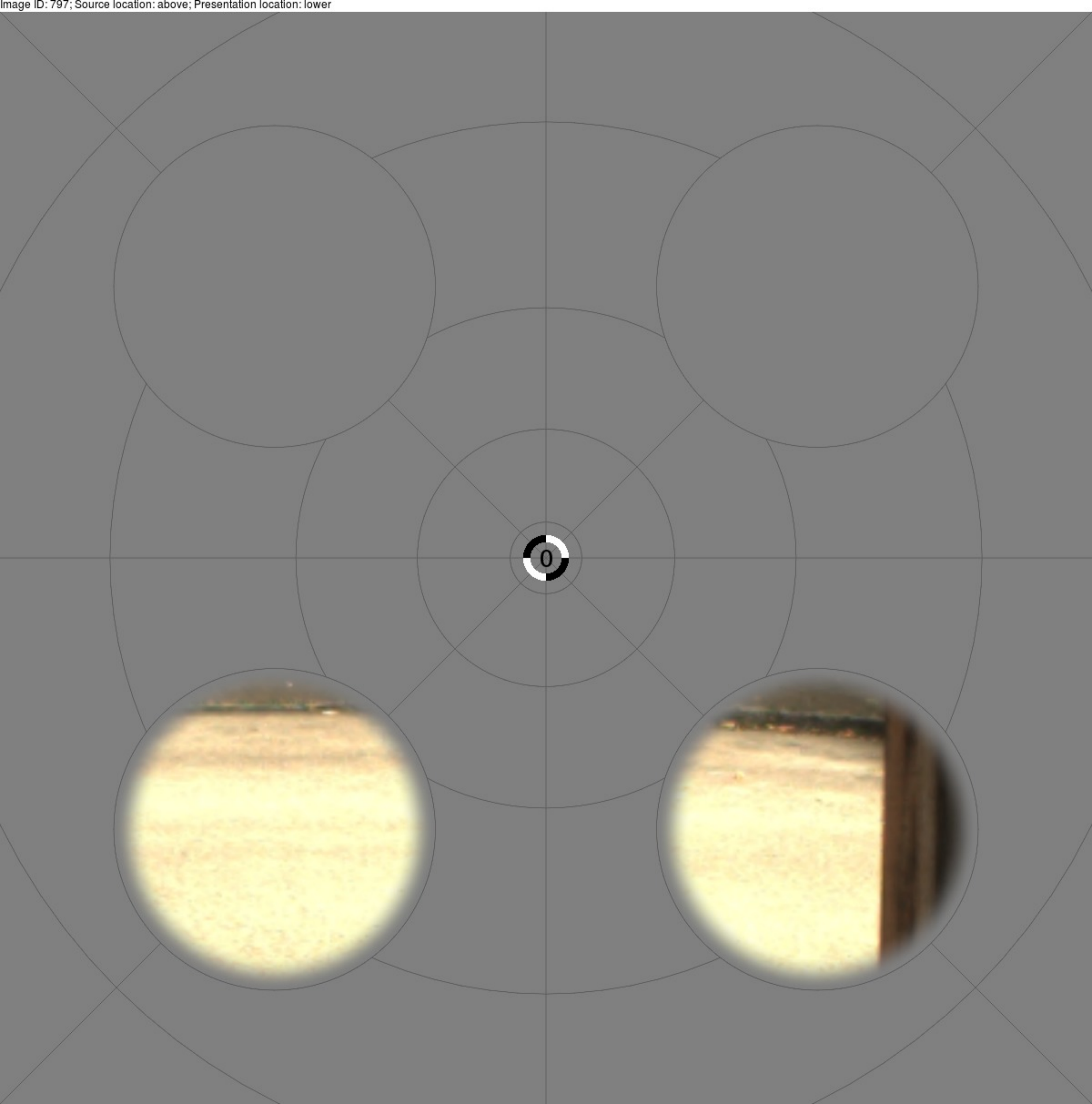

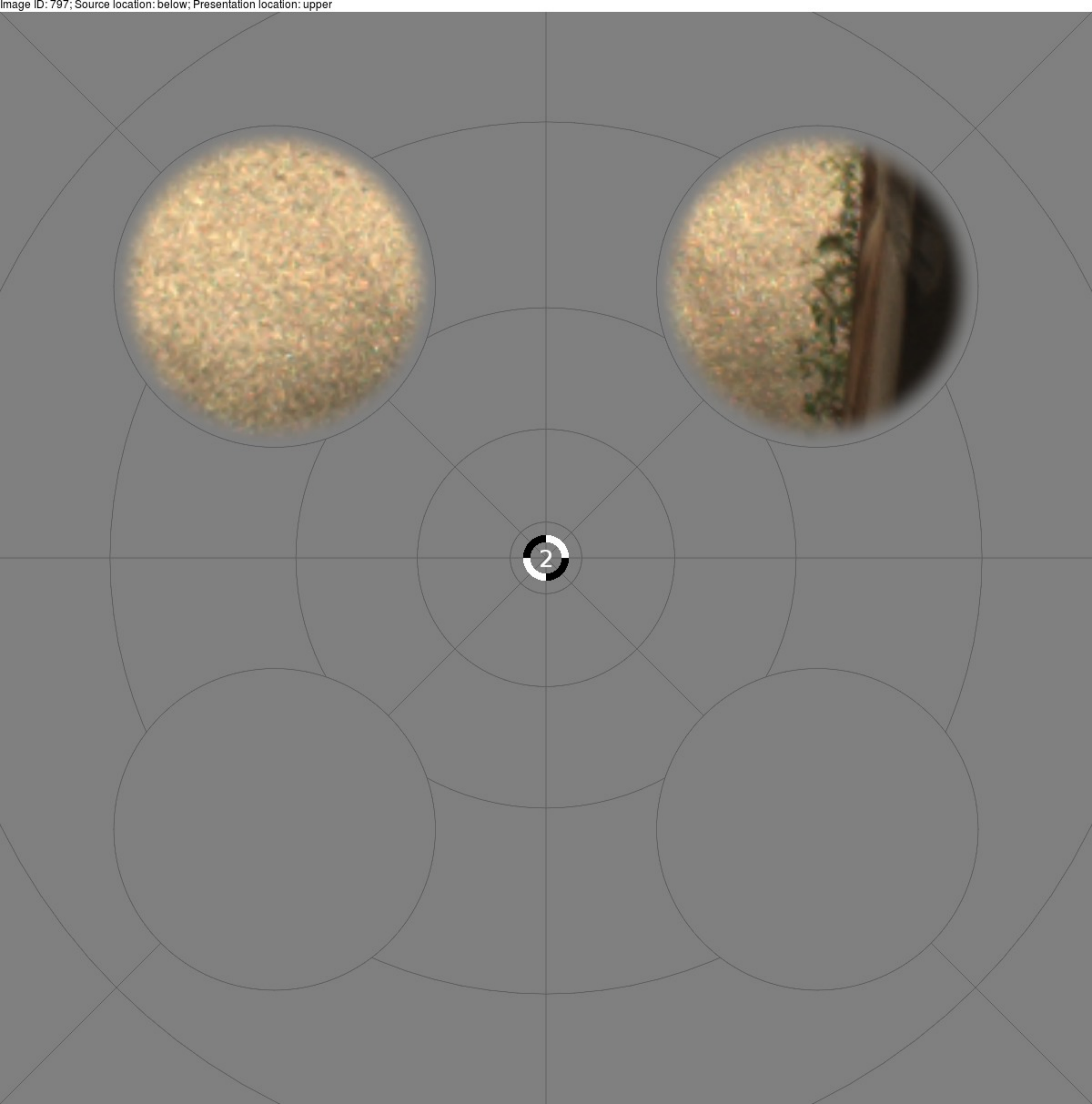

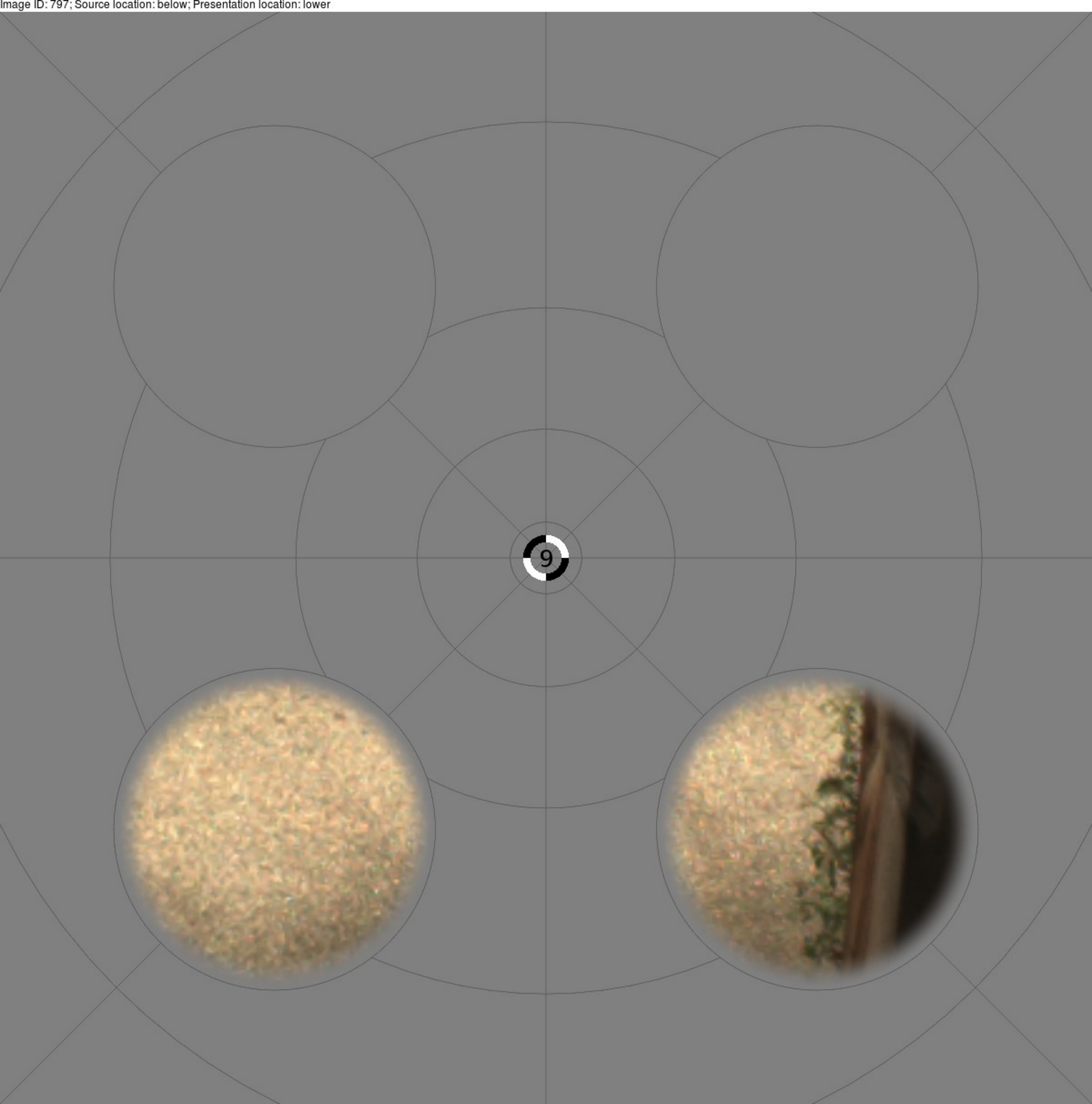

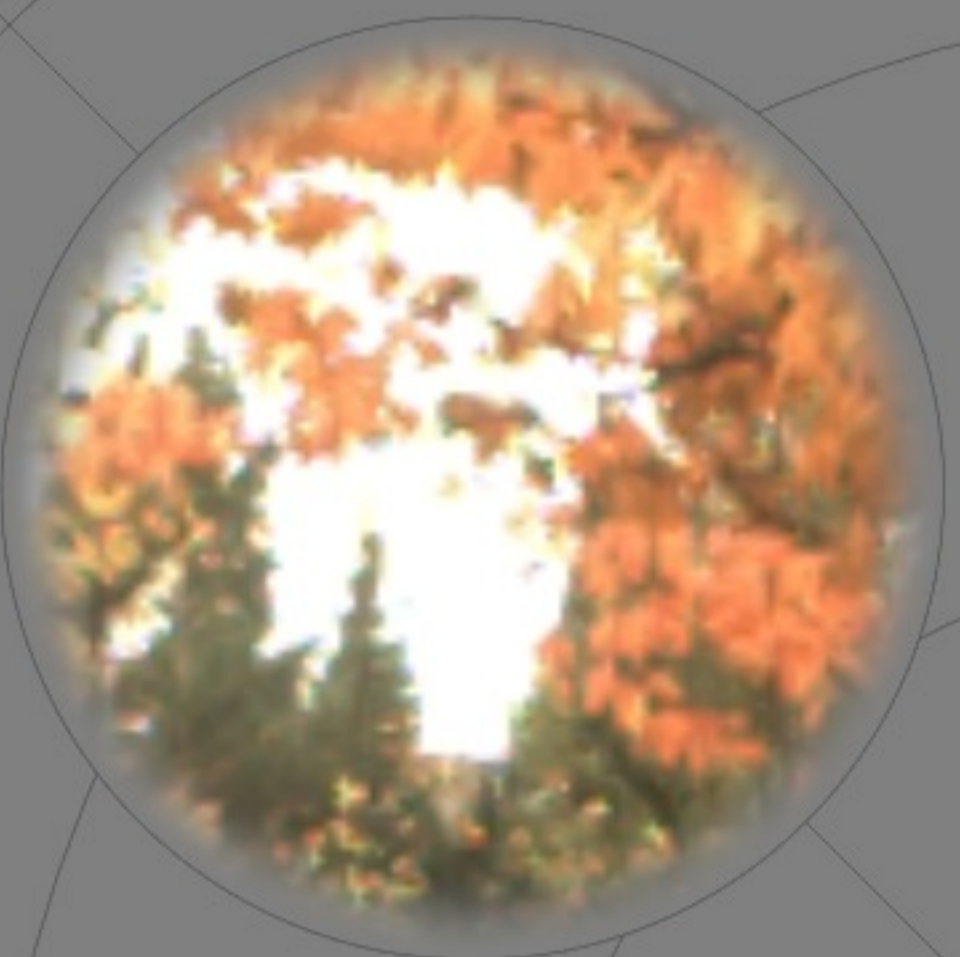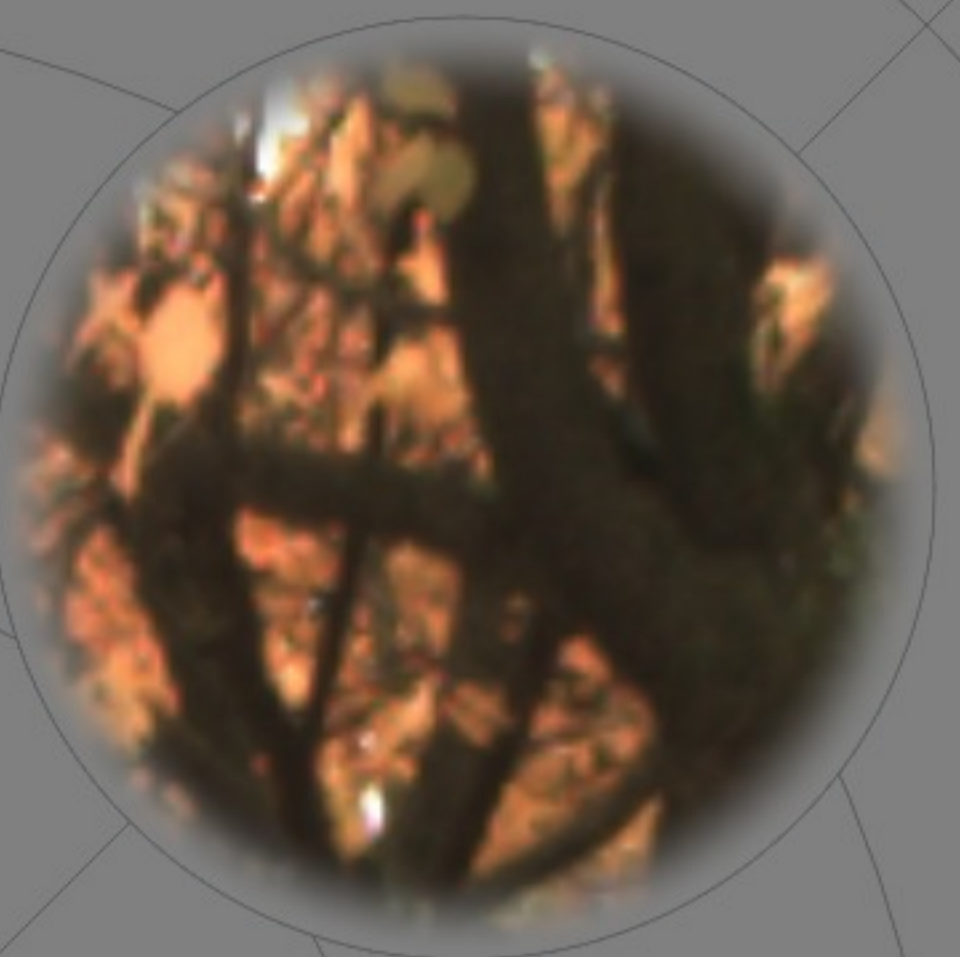

1

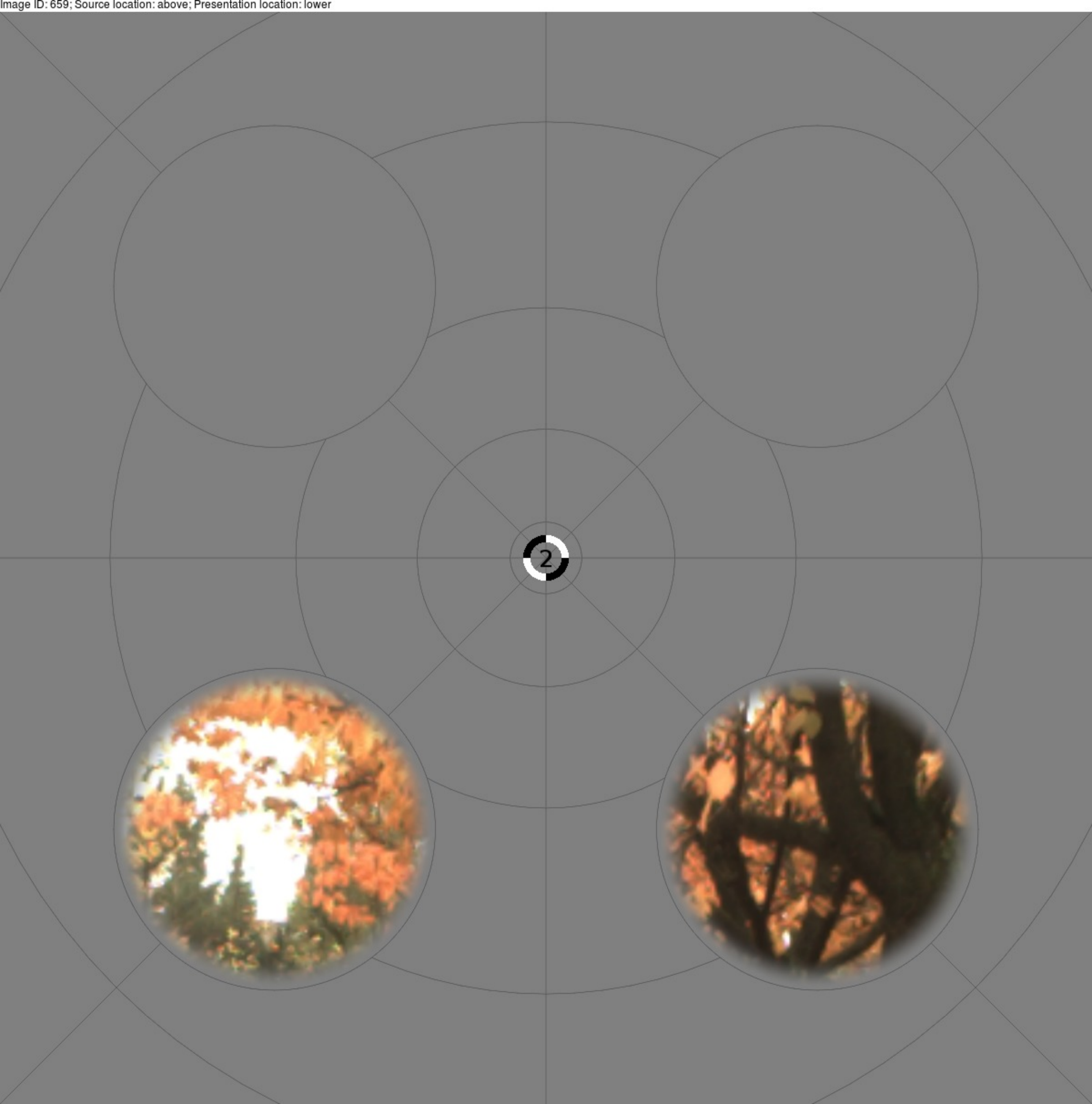

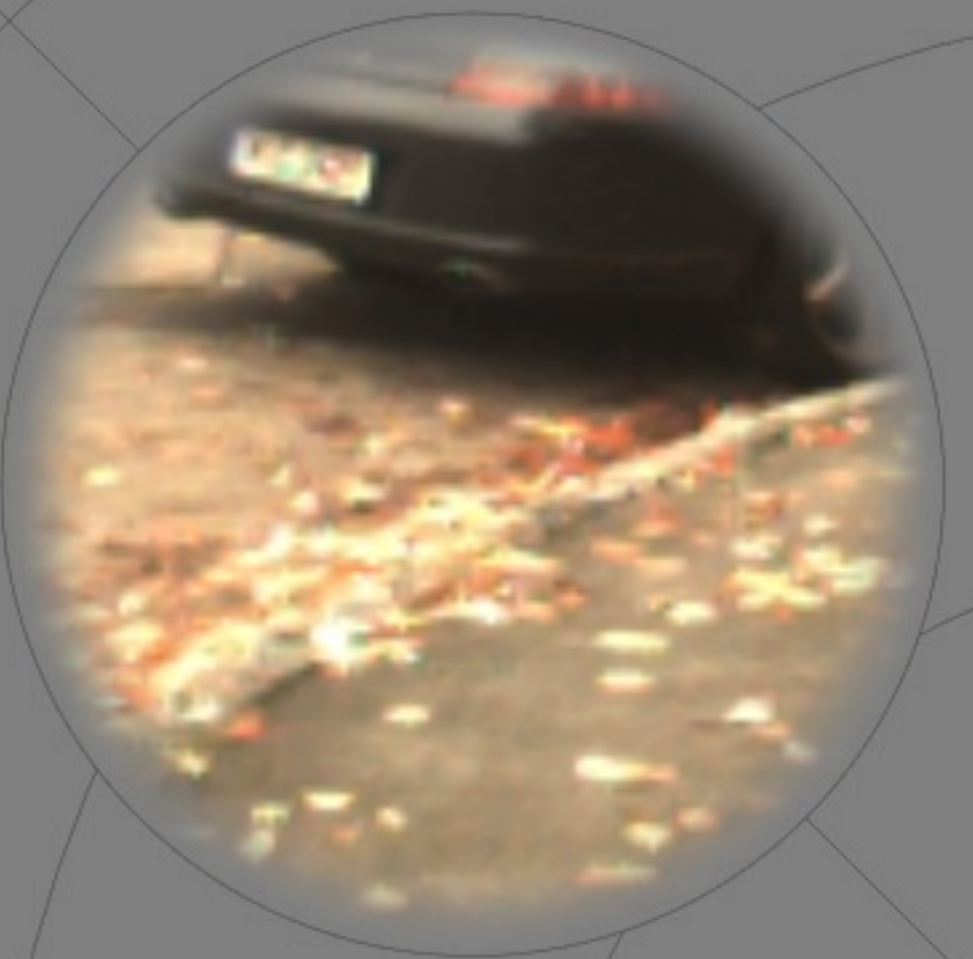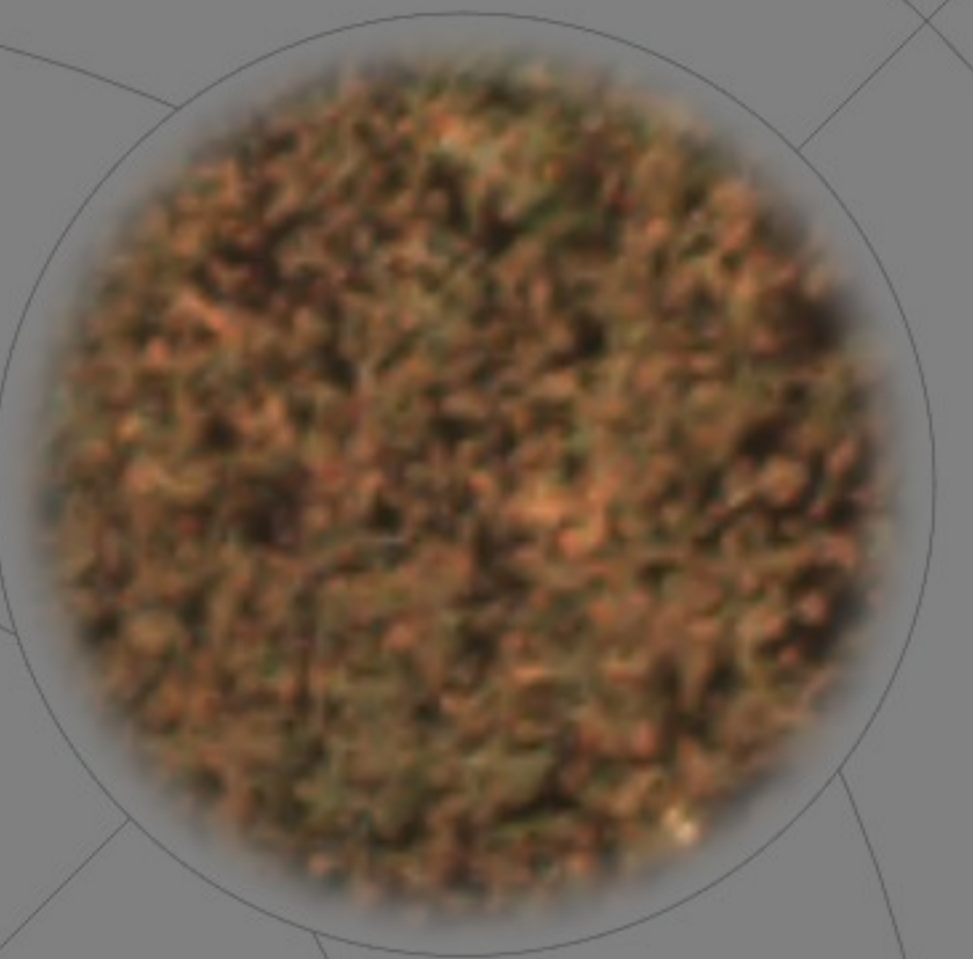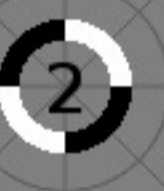

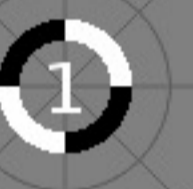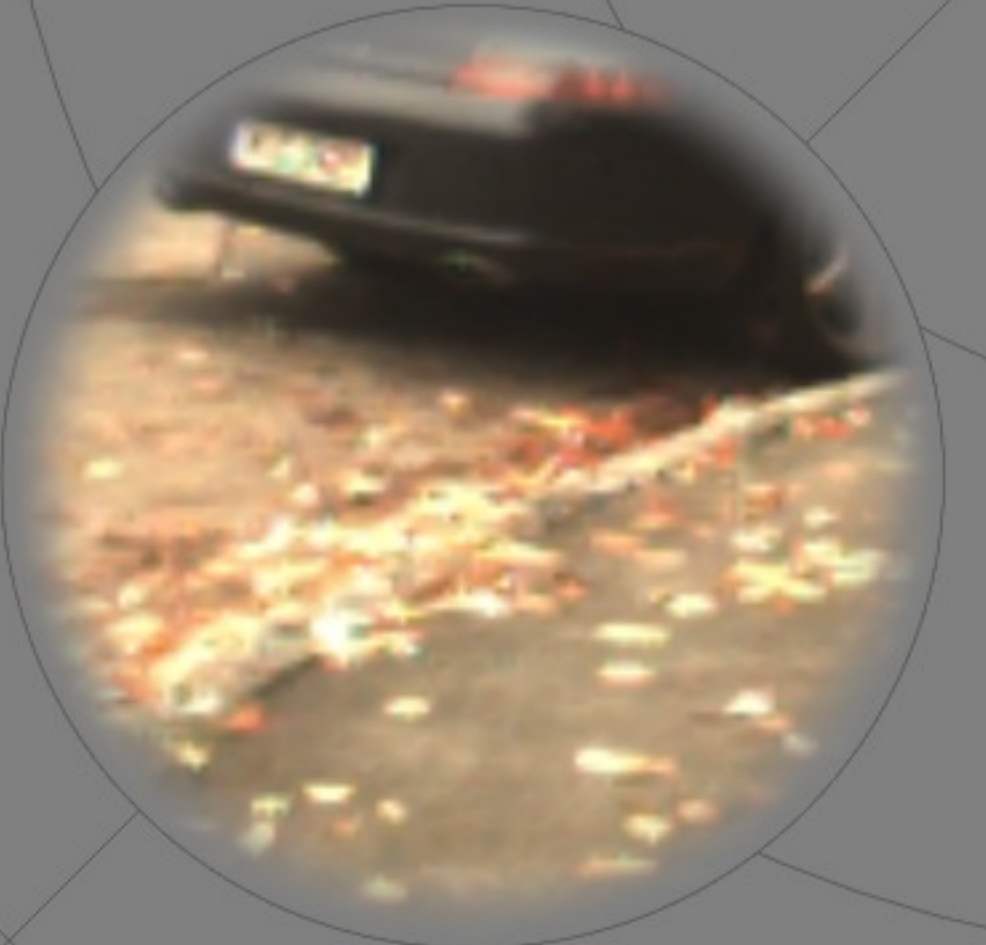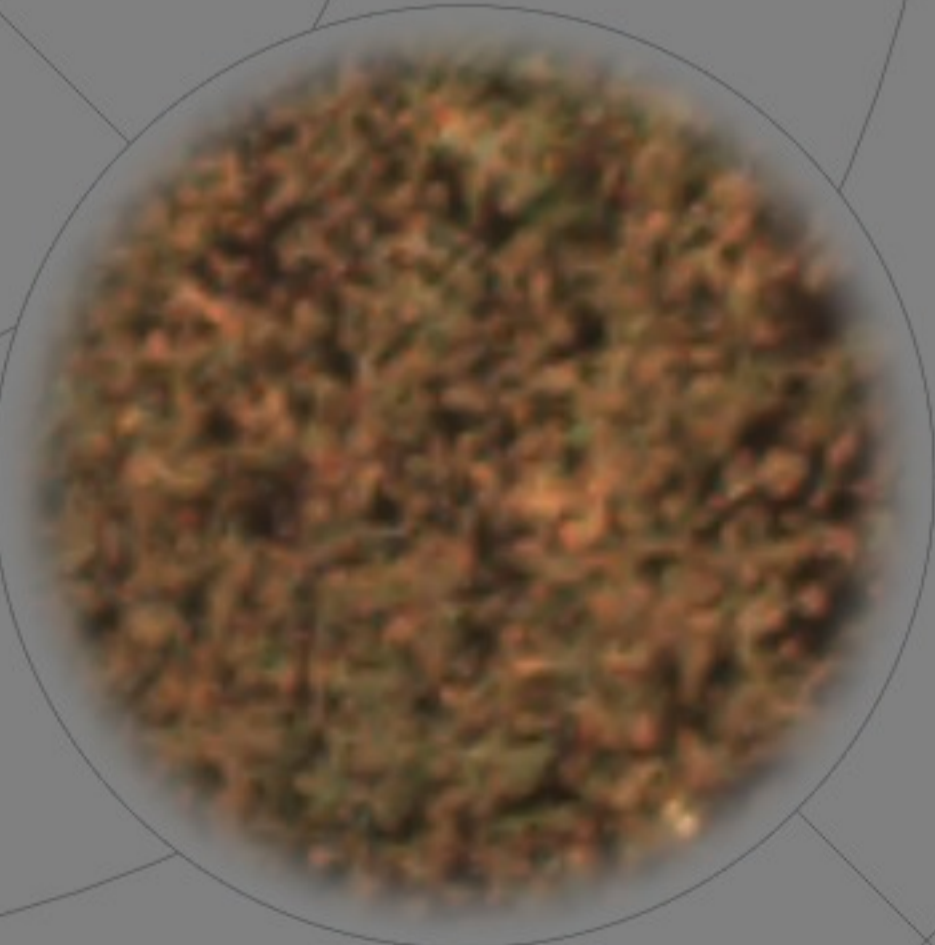

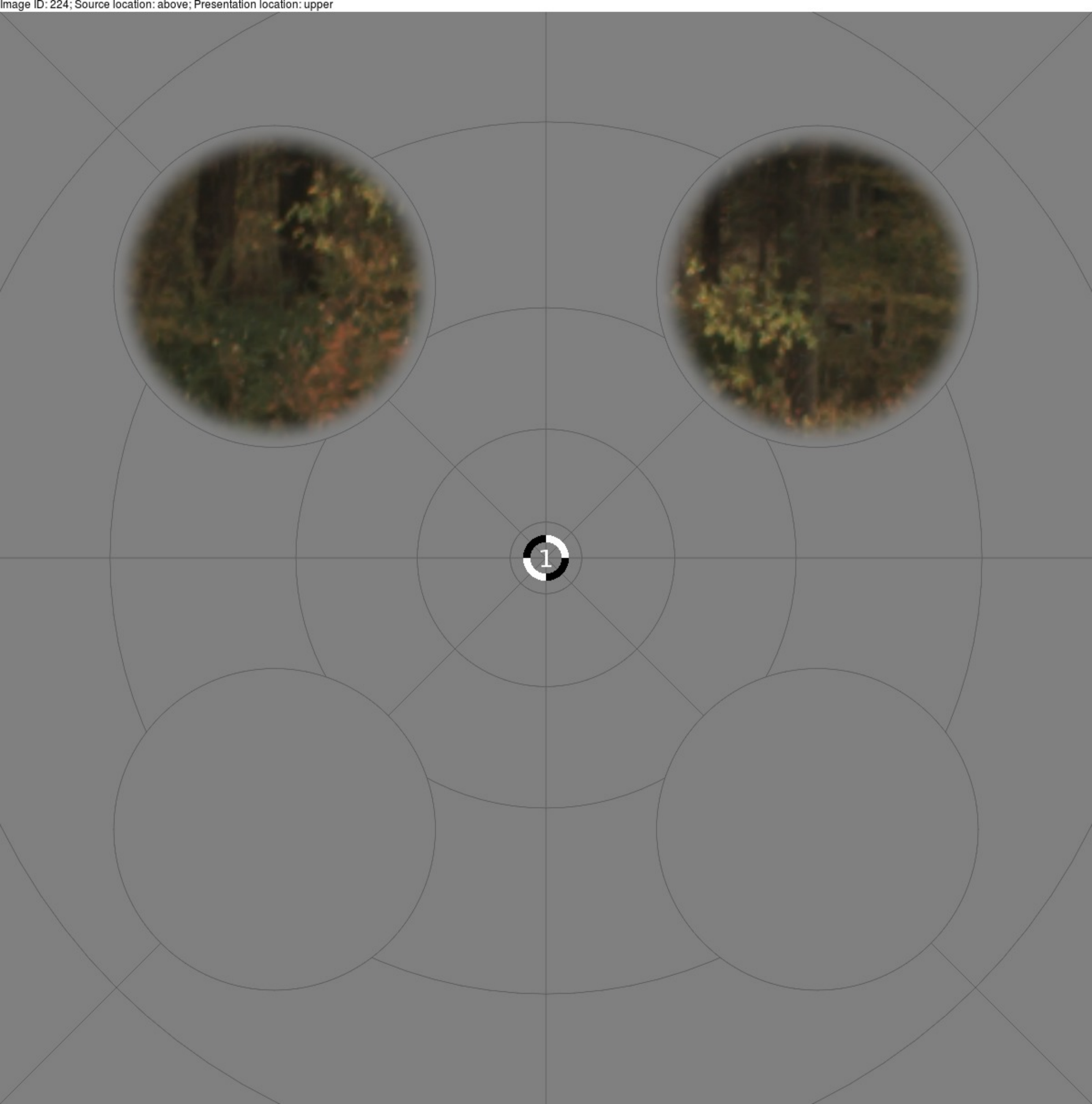

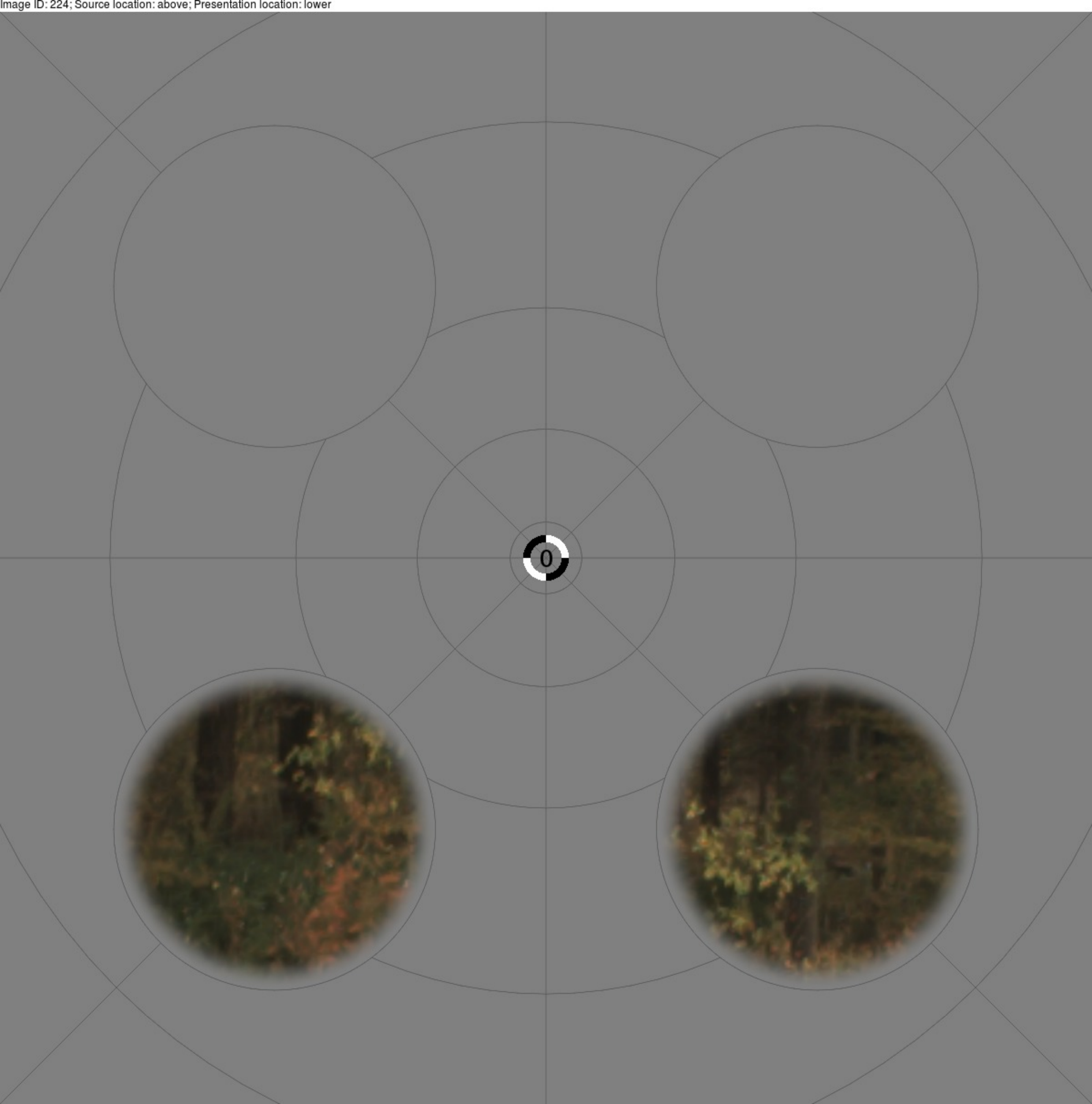

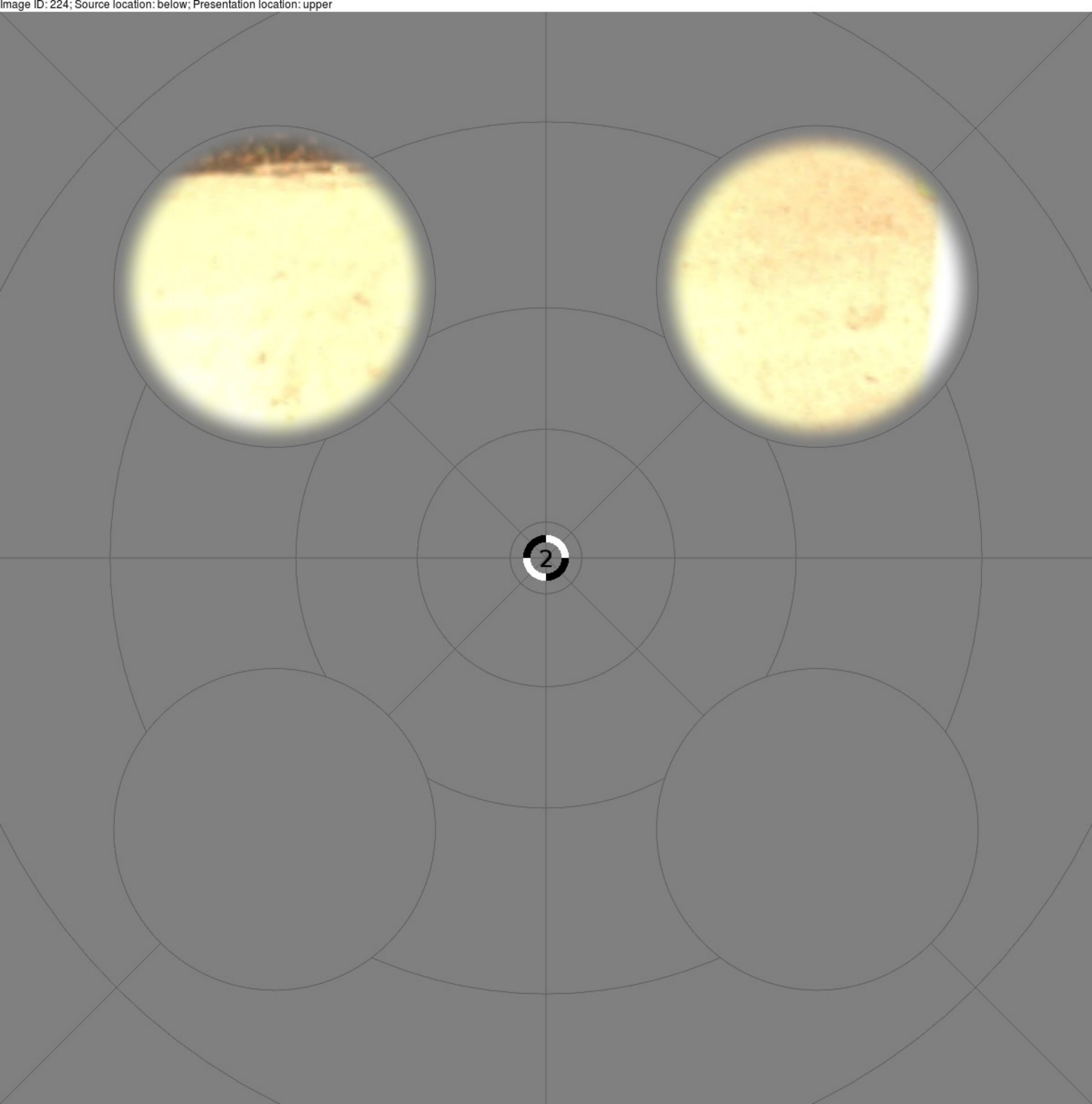

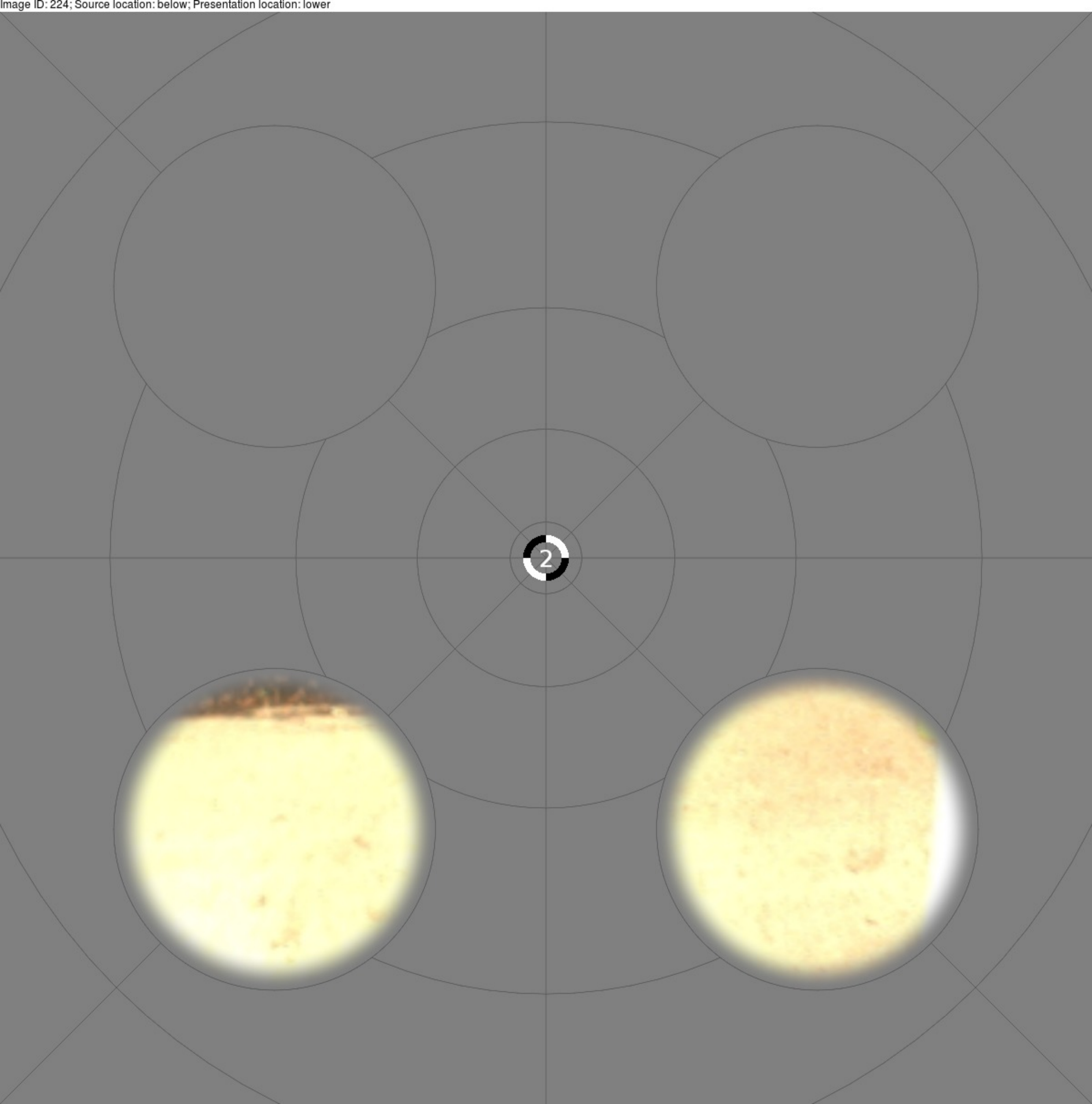

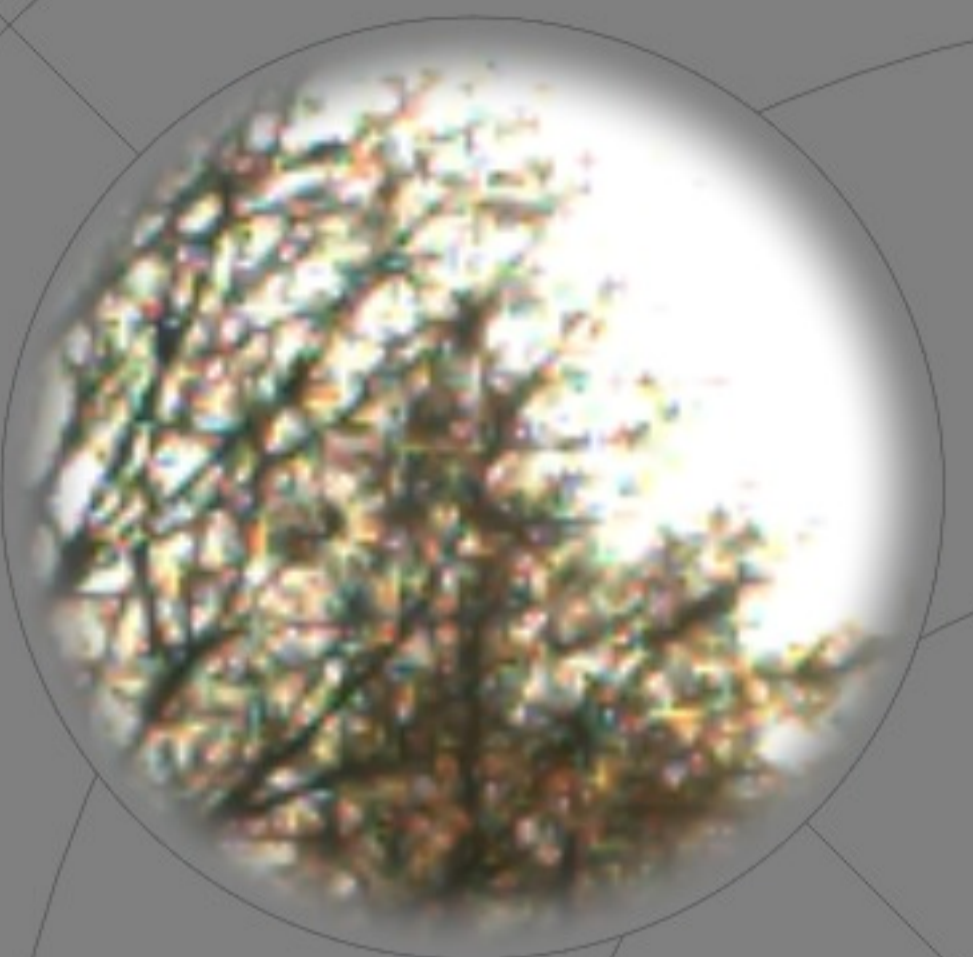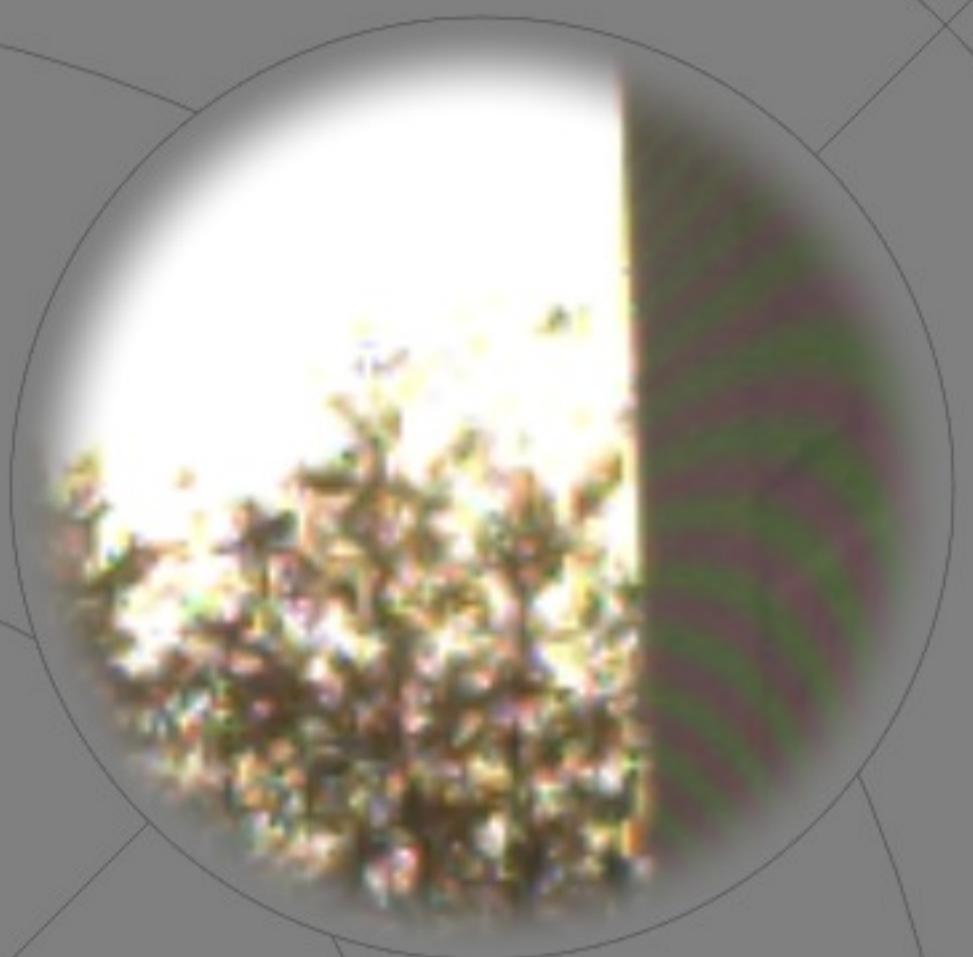

6

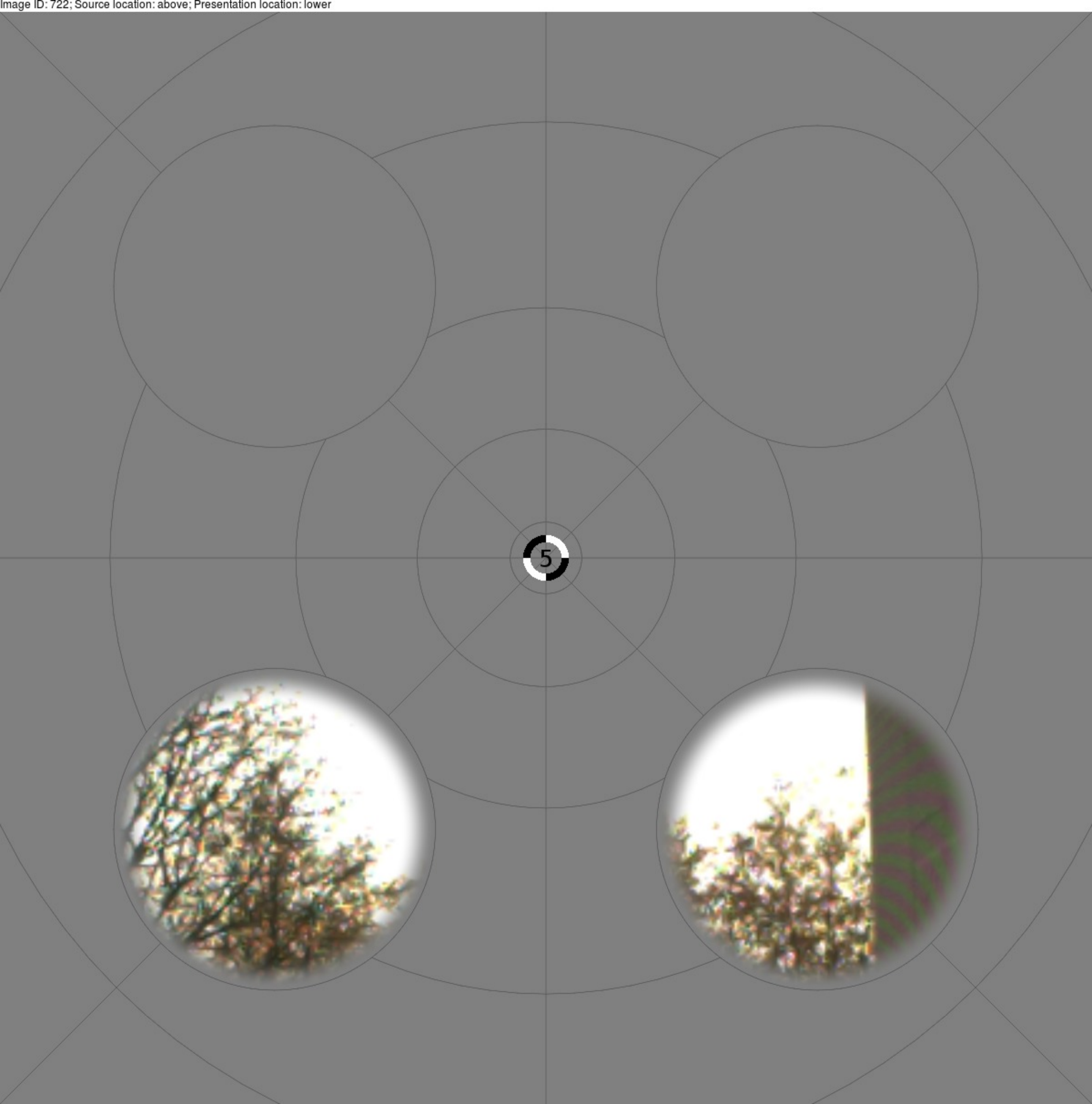

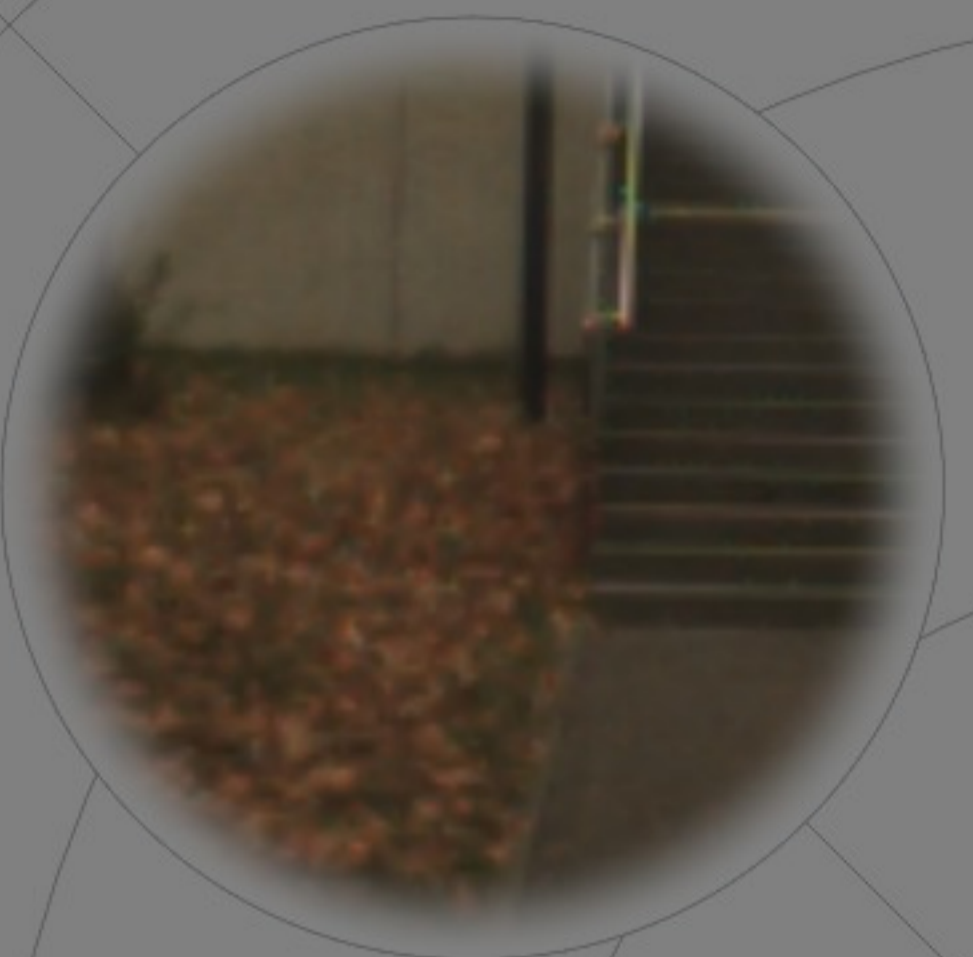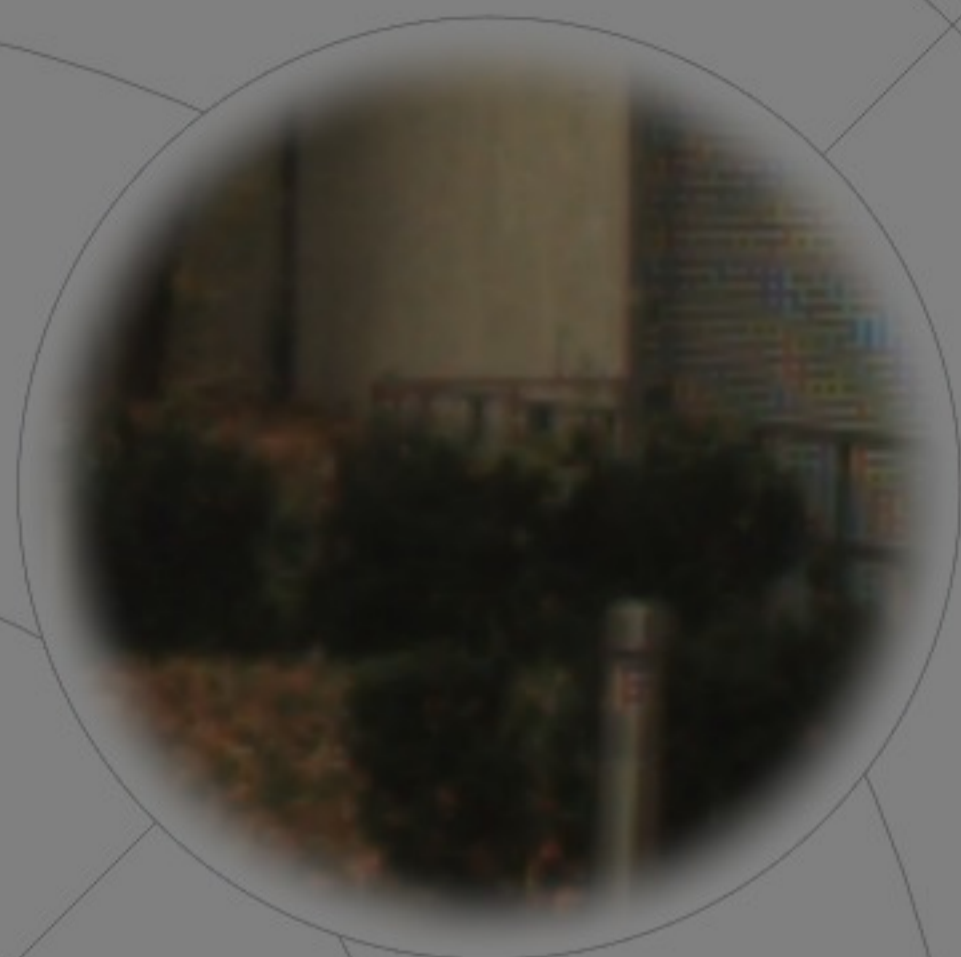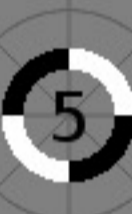

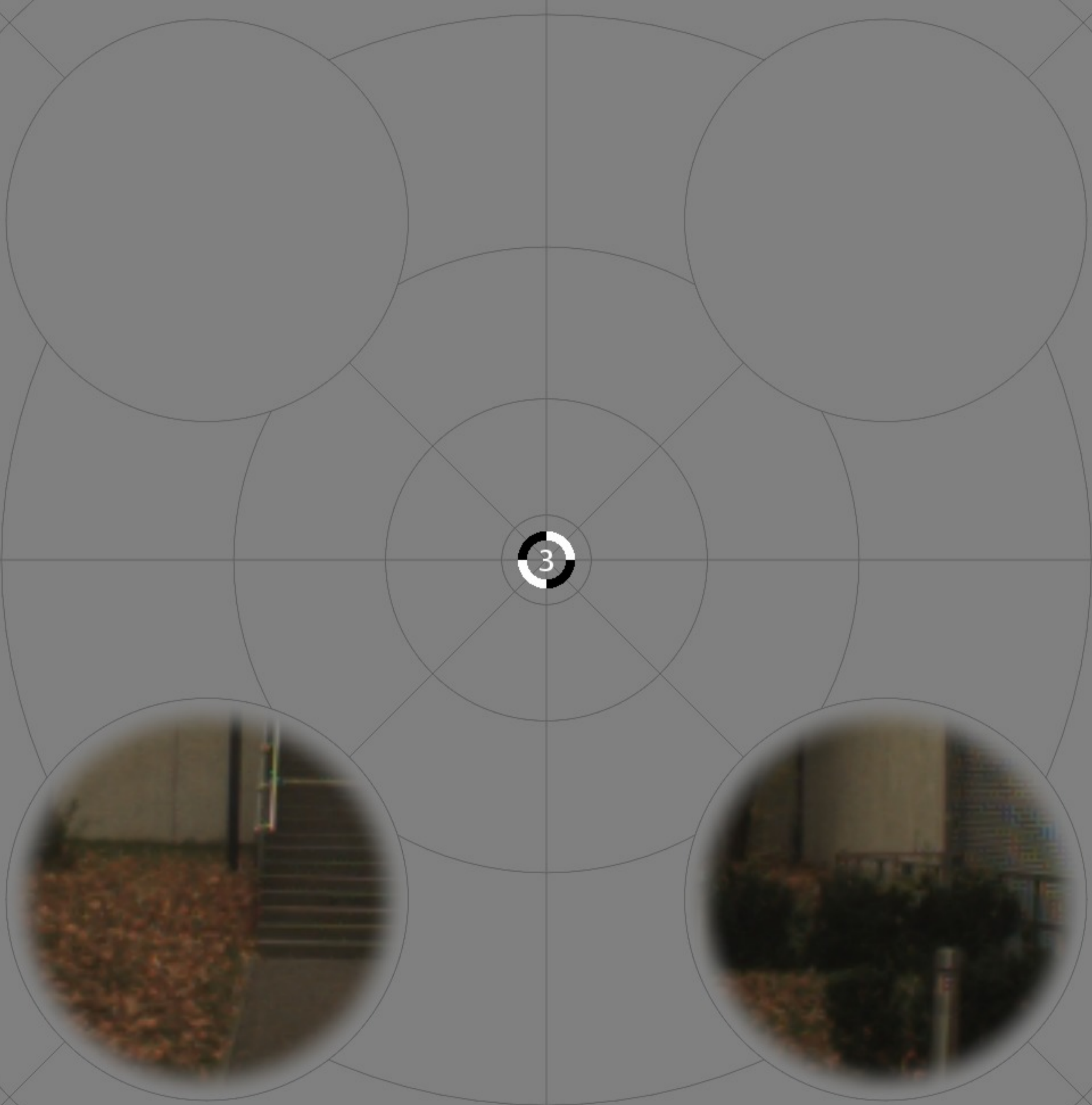

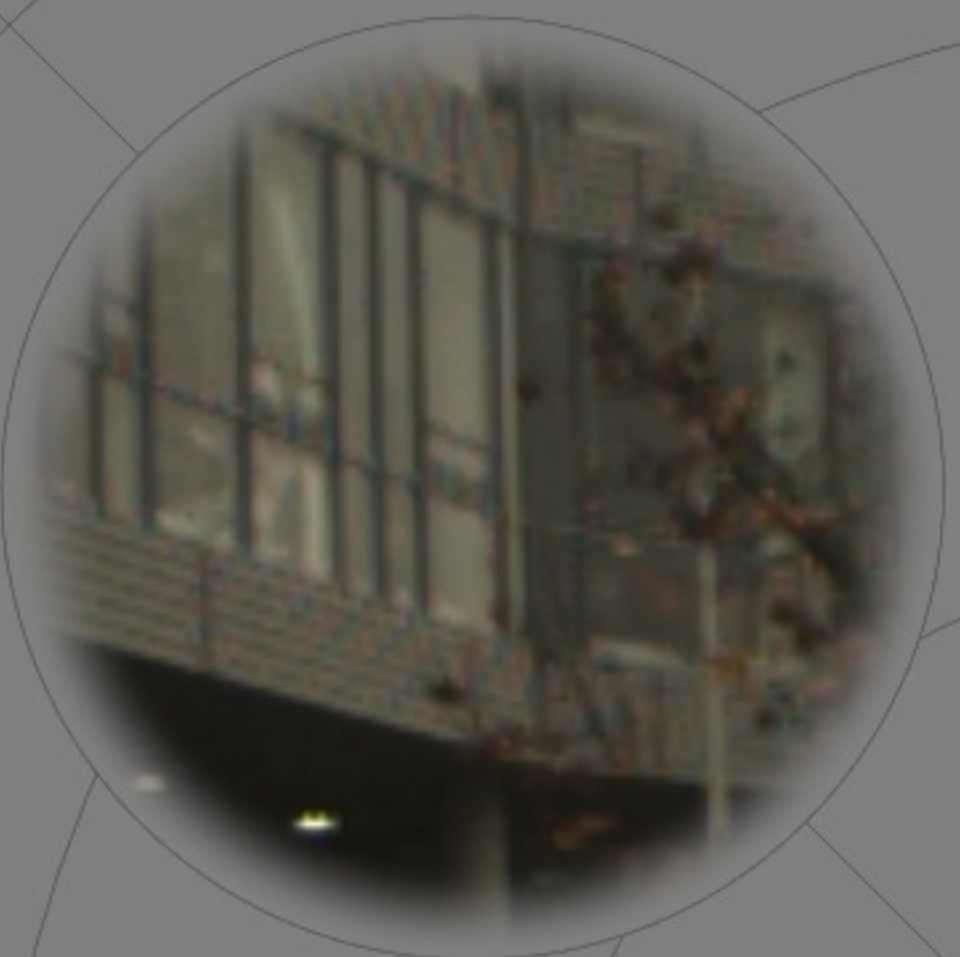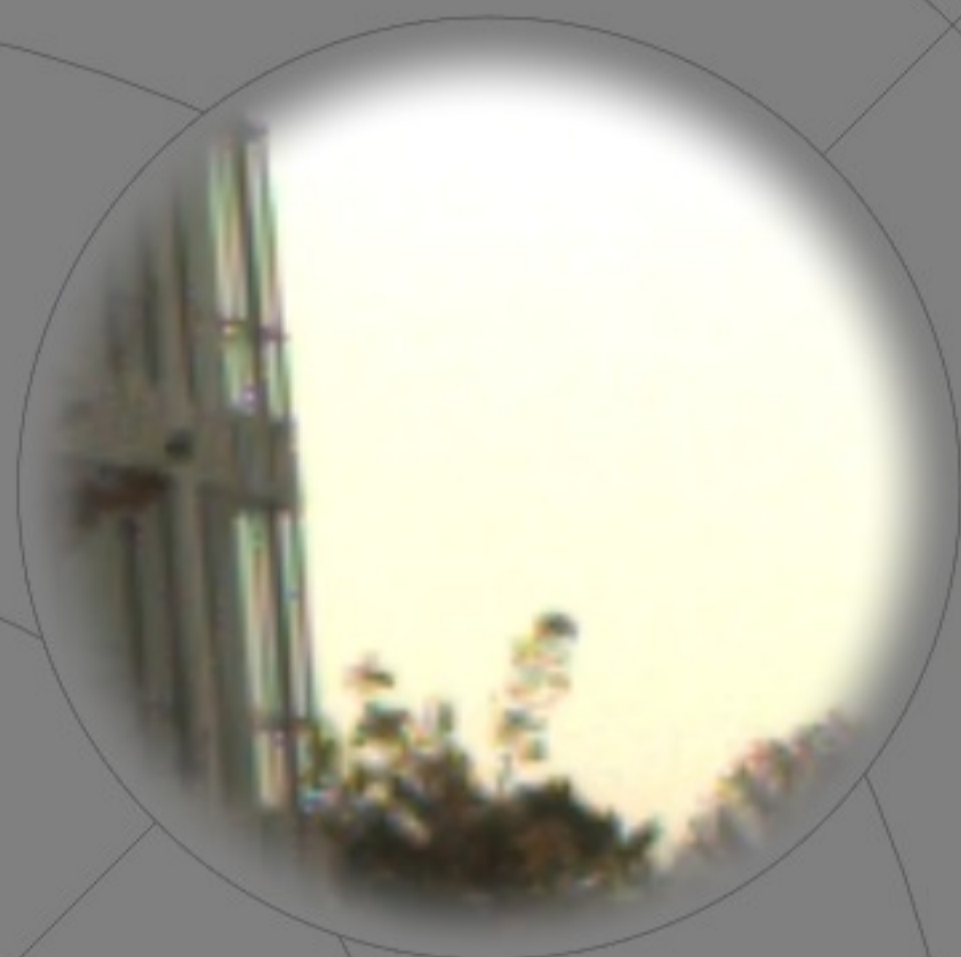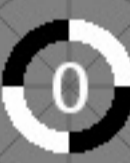

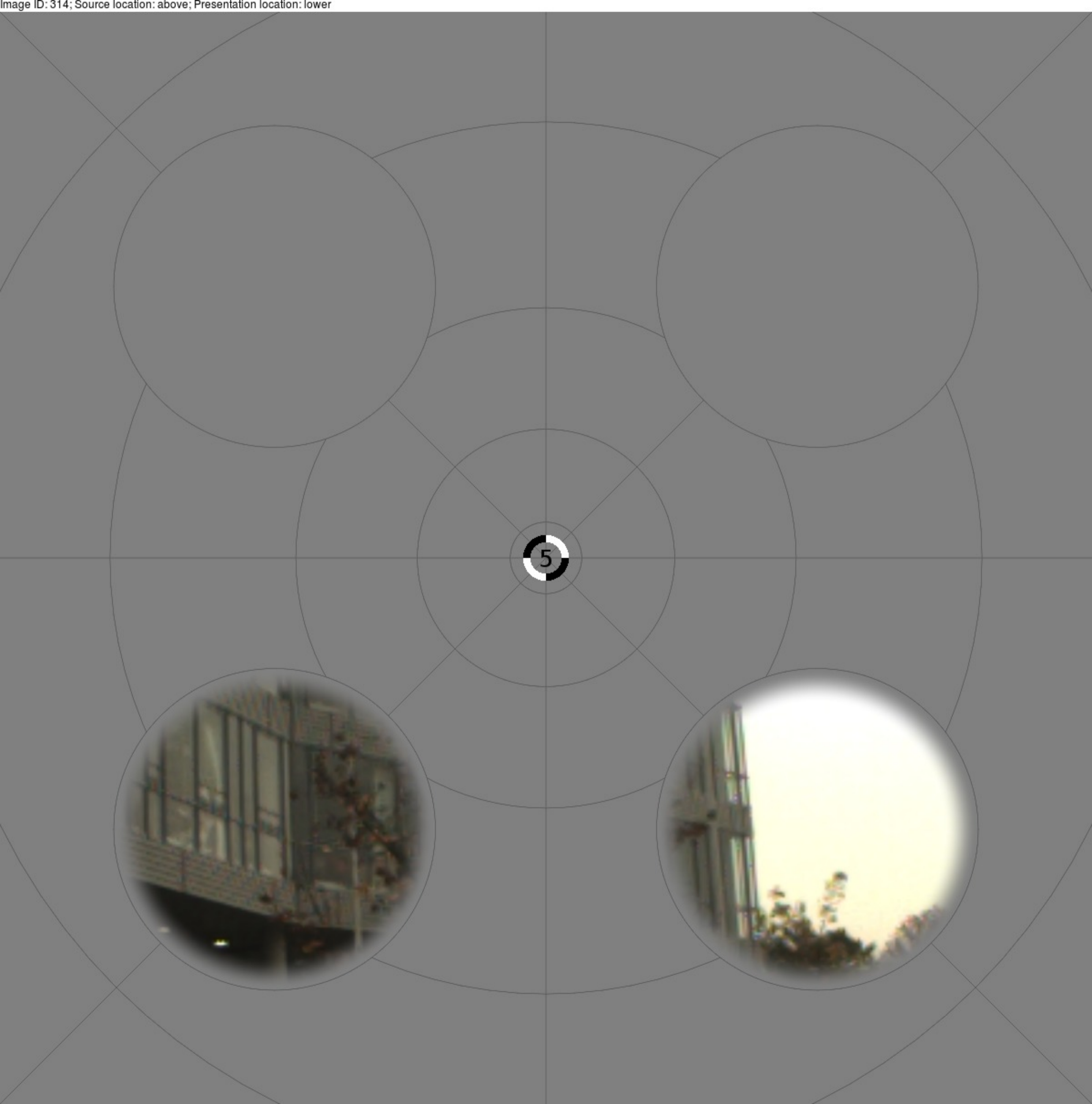

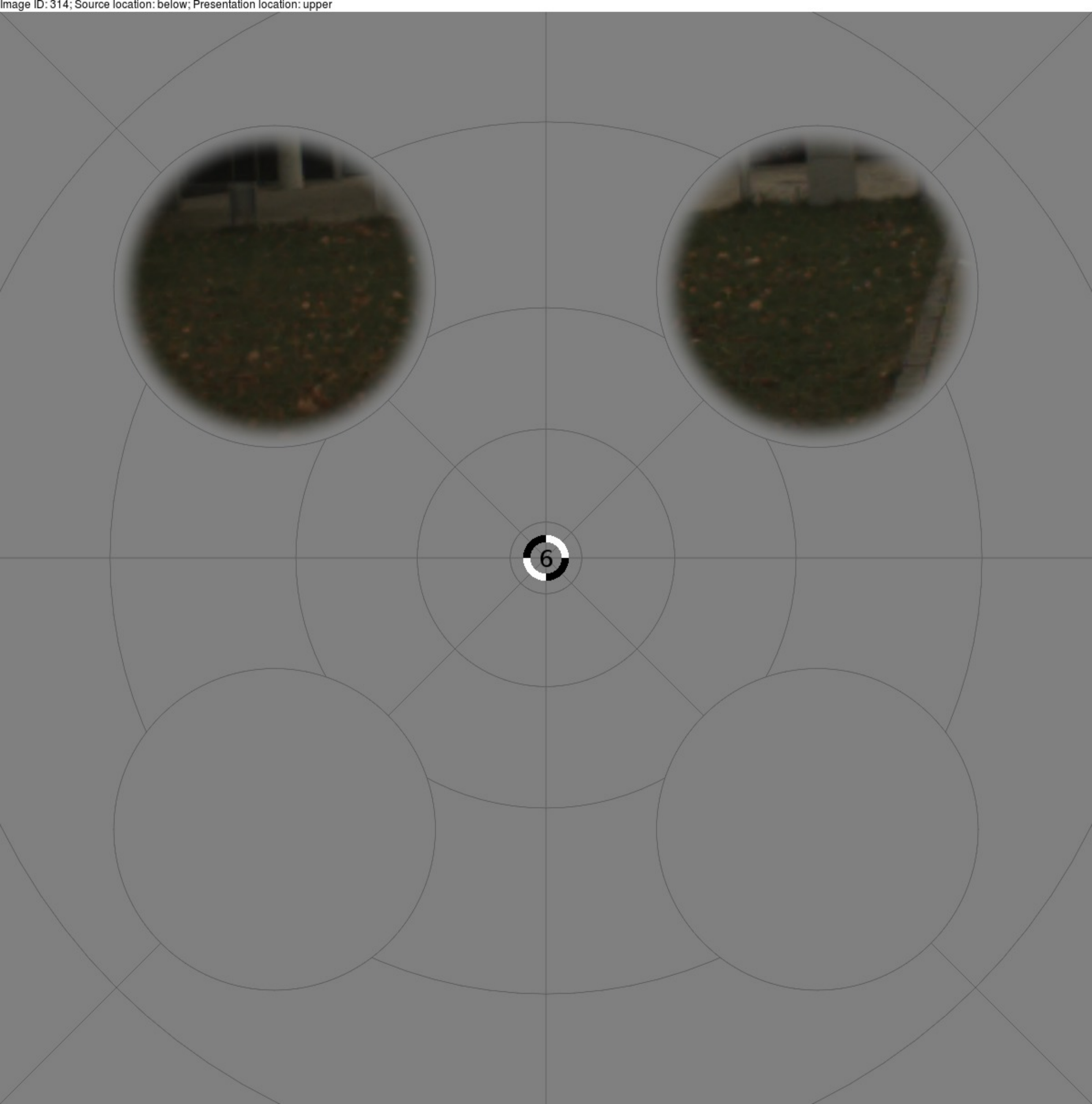

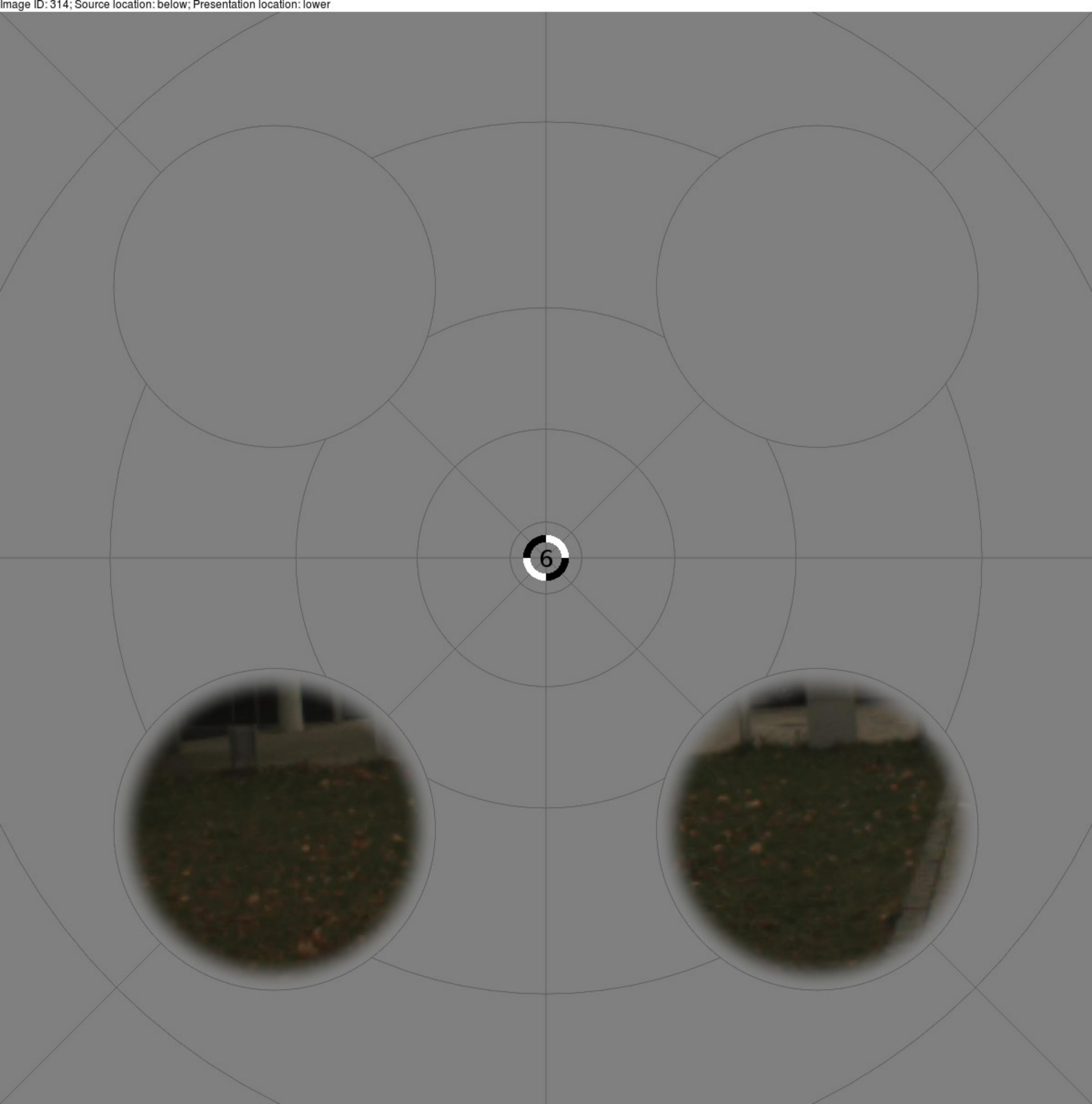

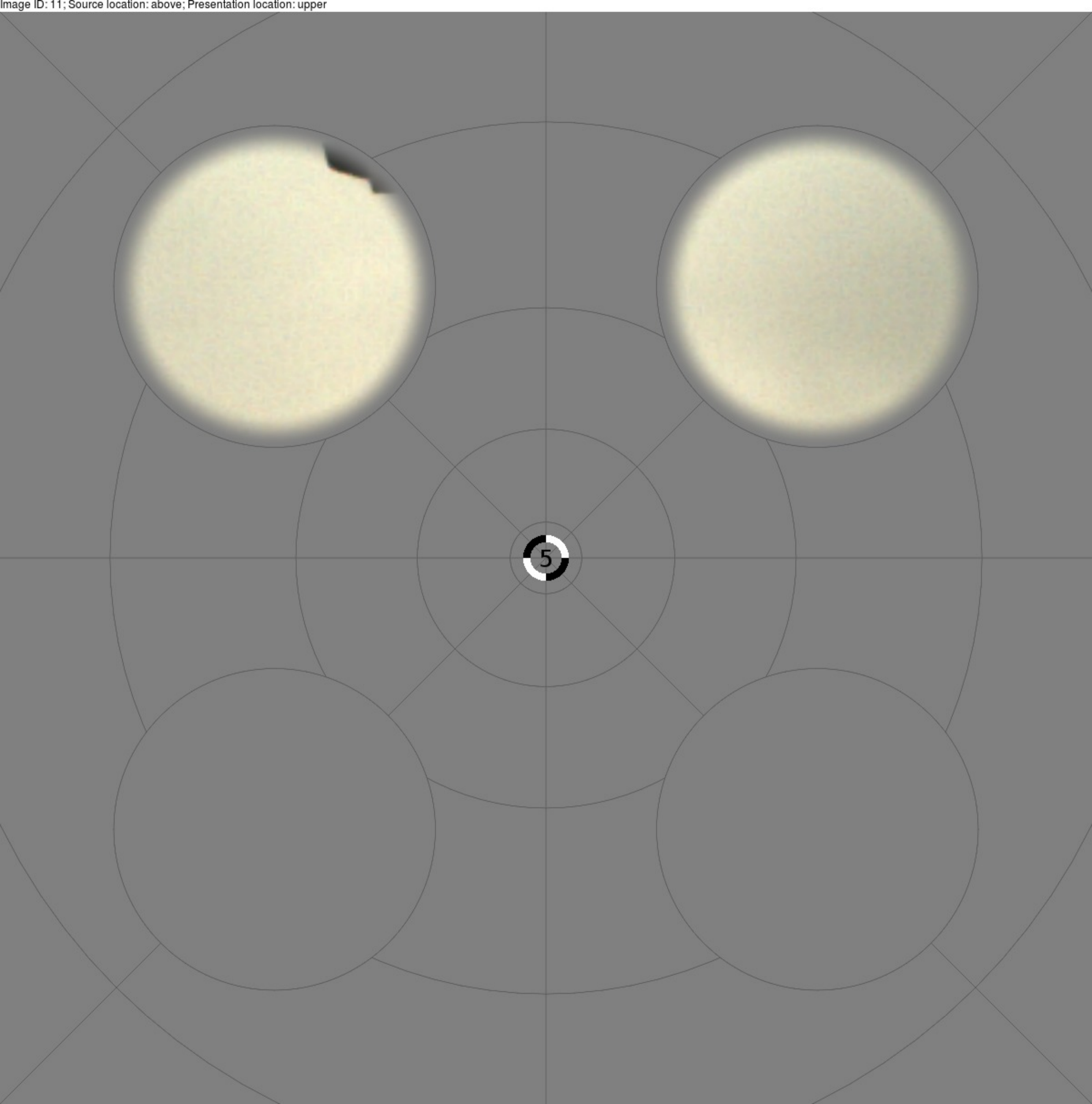

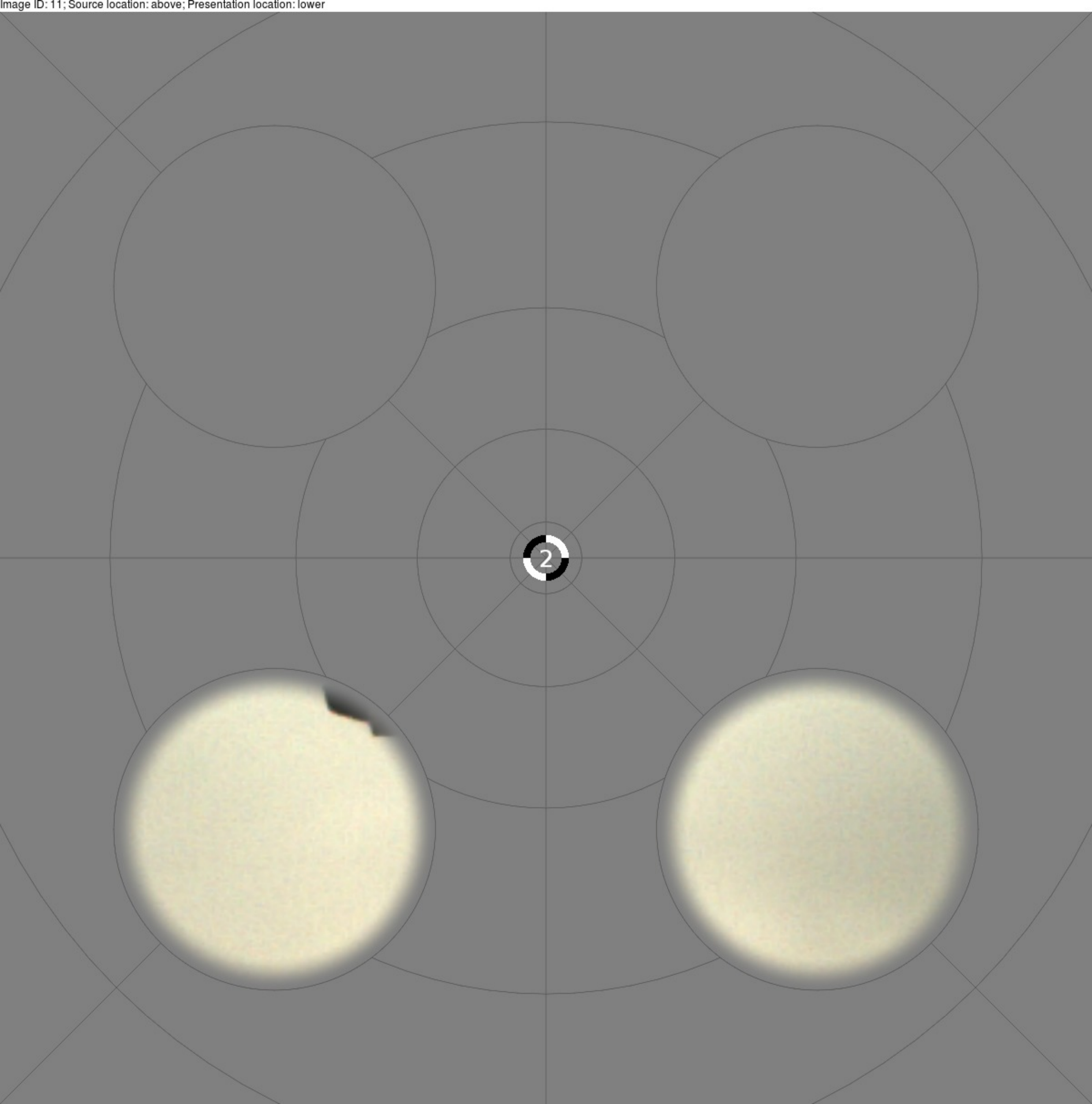

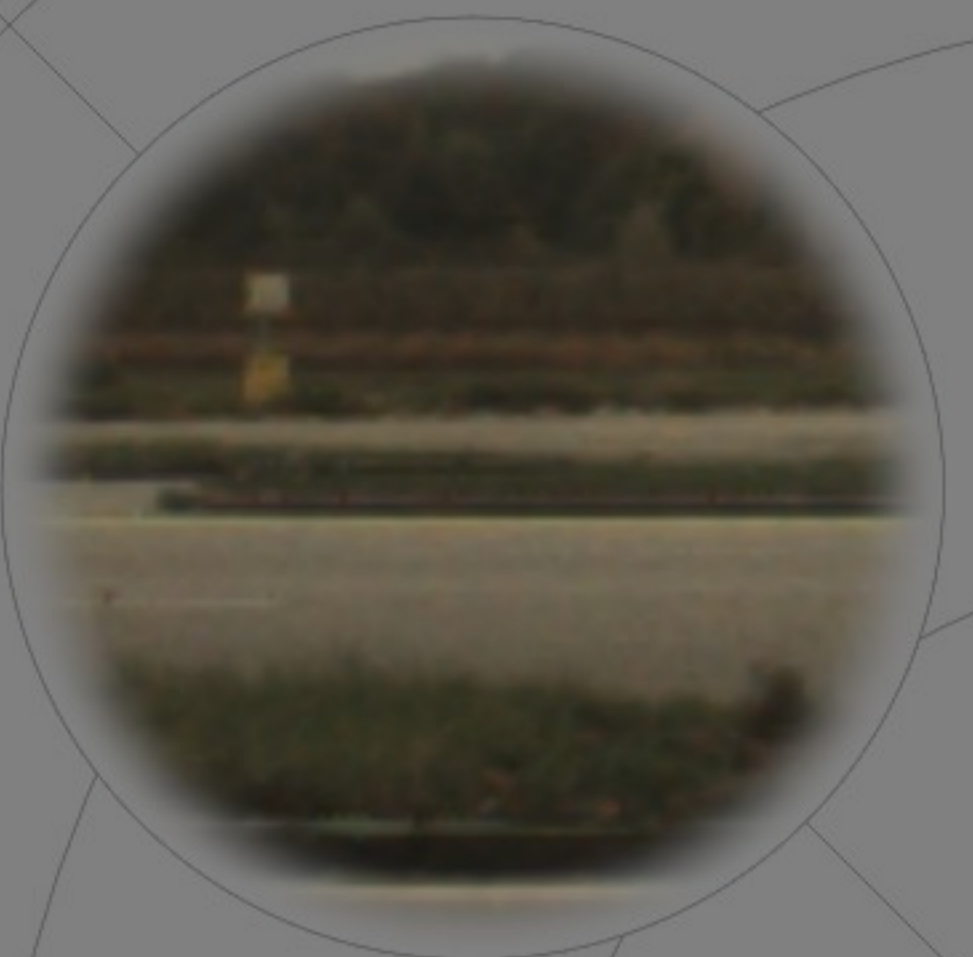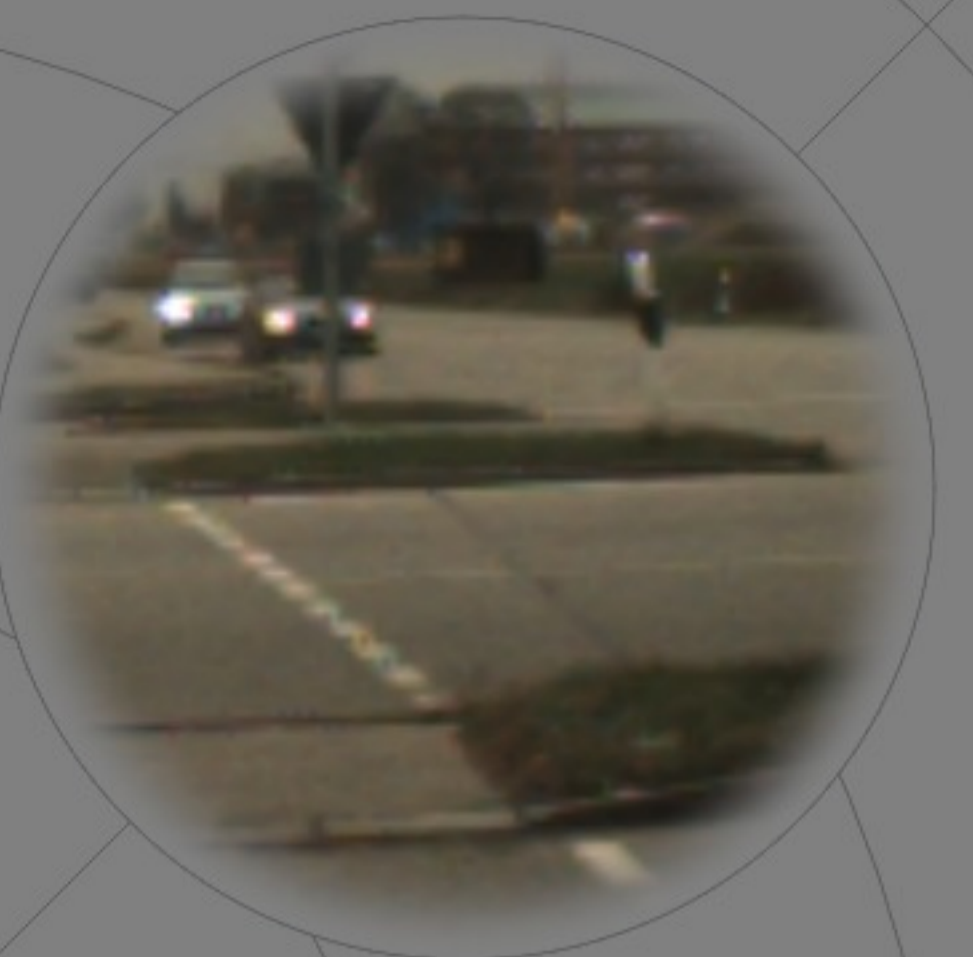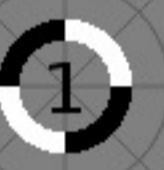

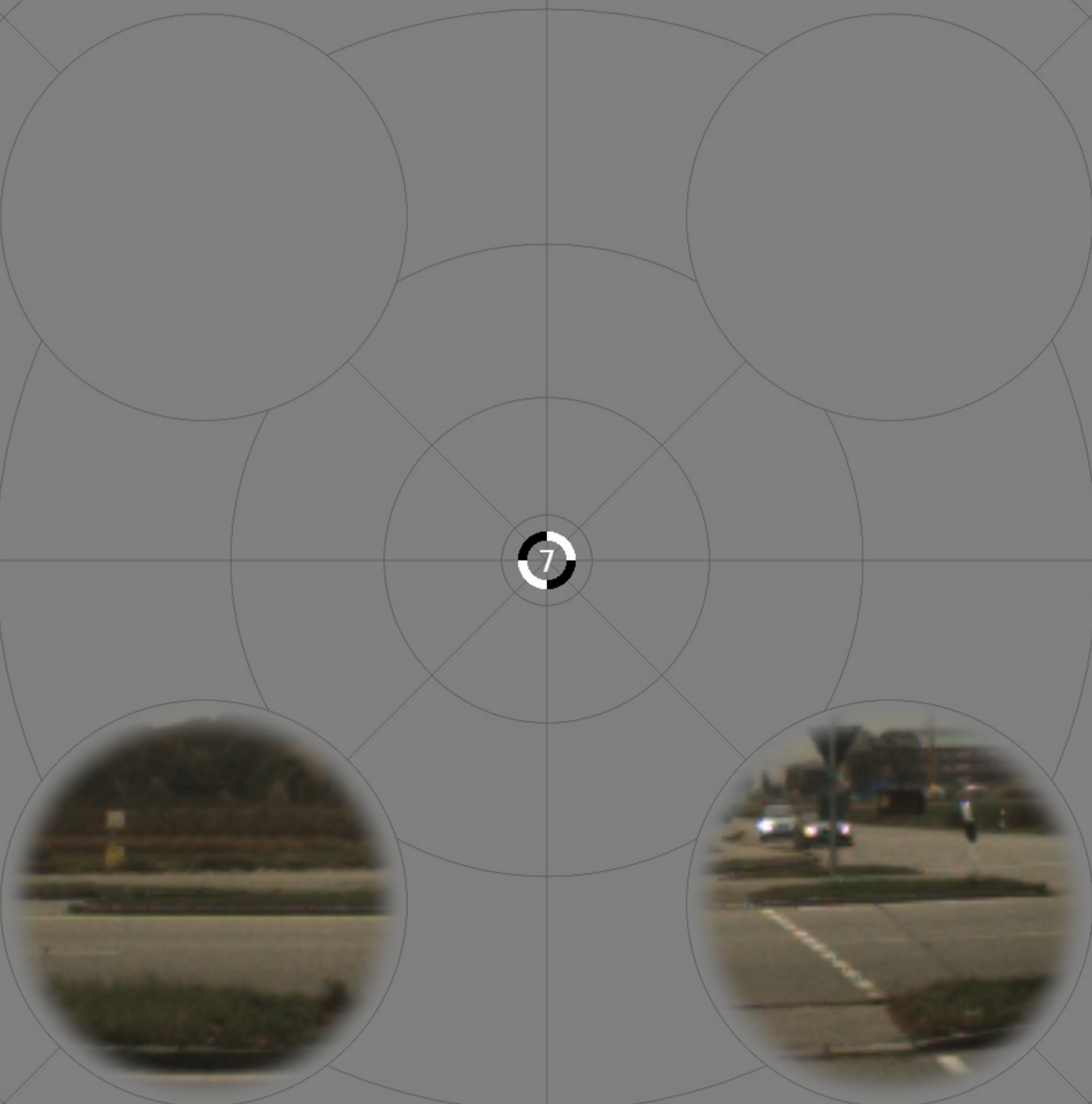

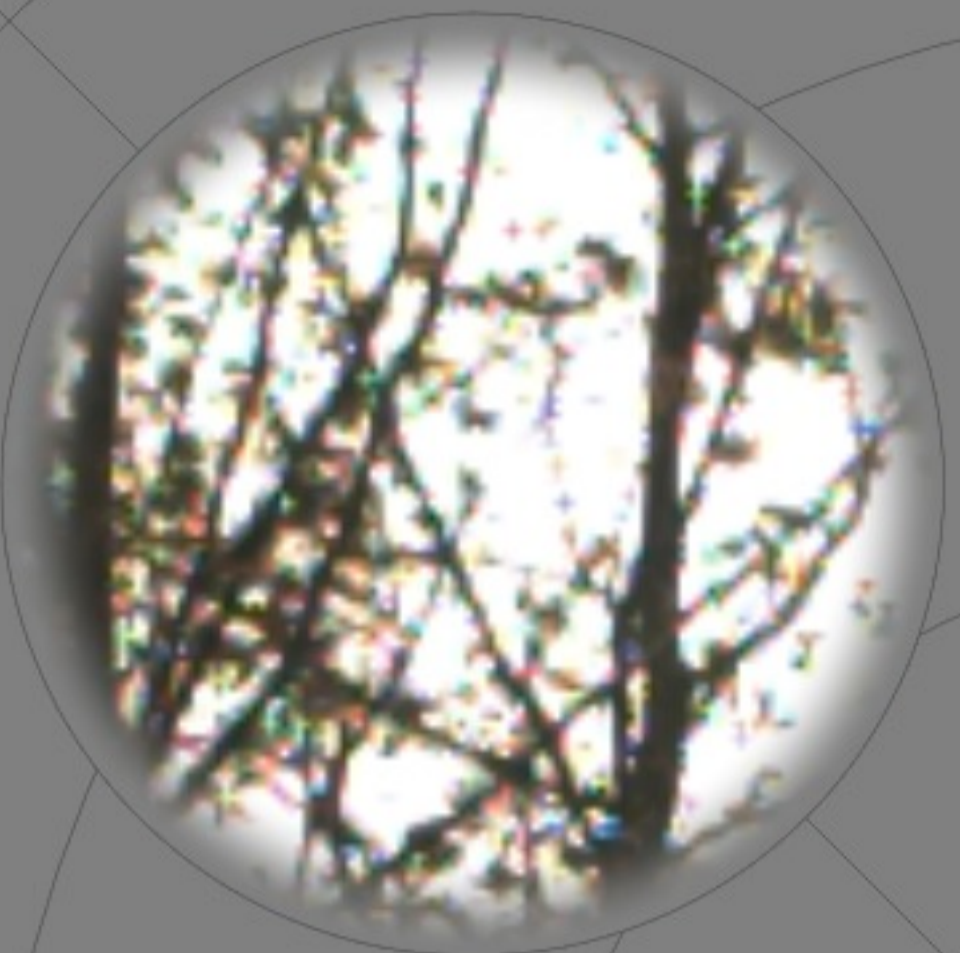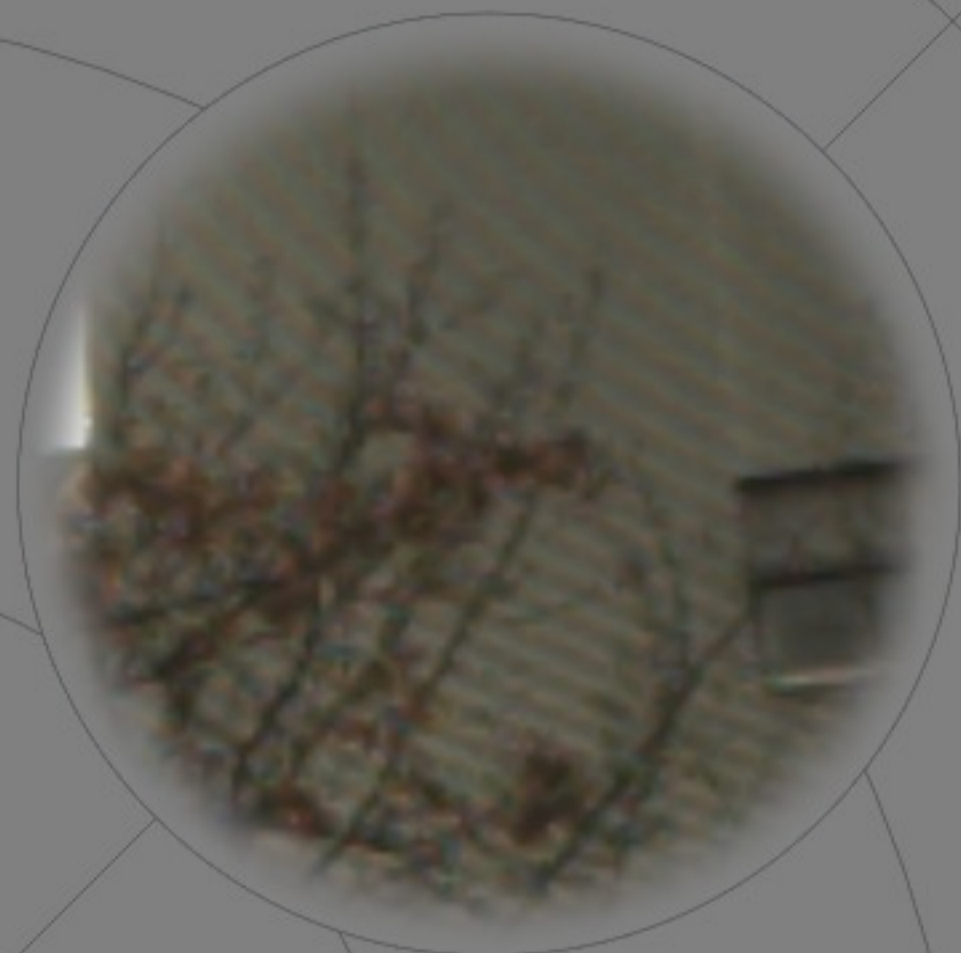

2

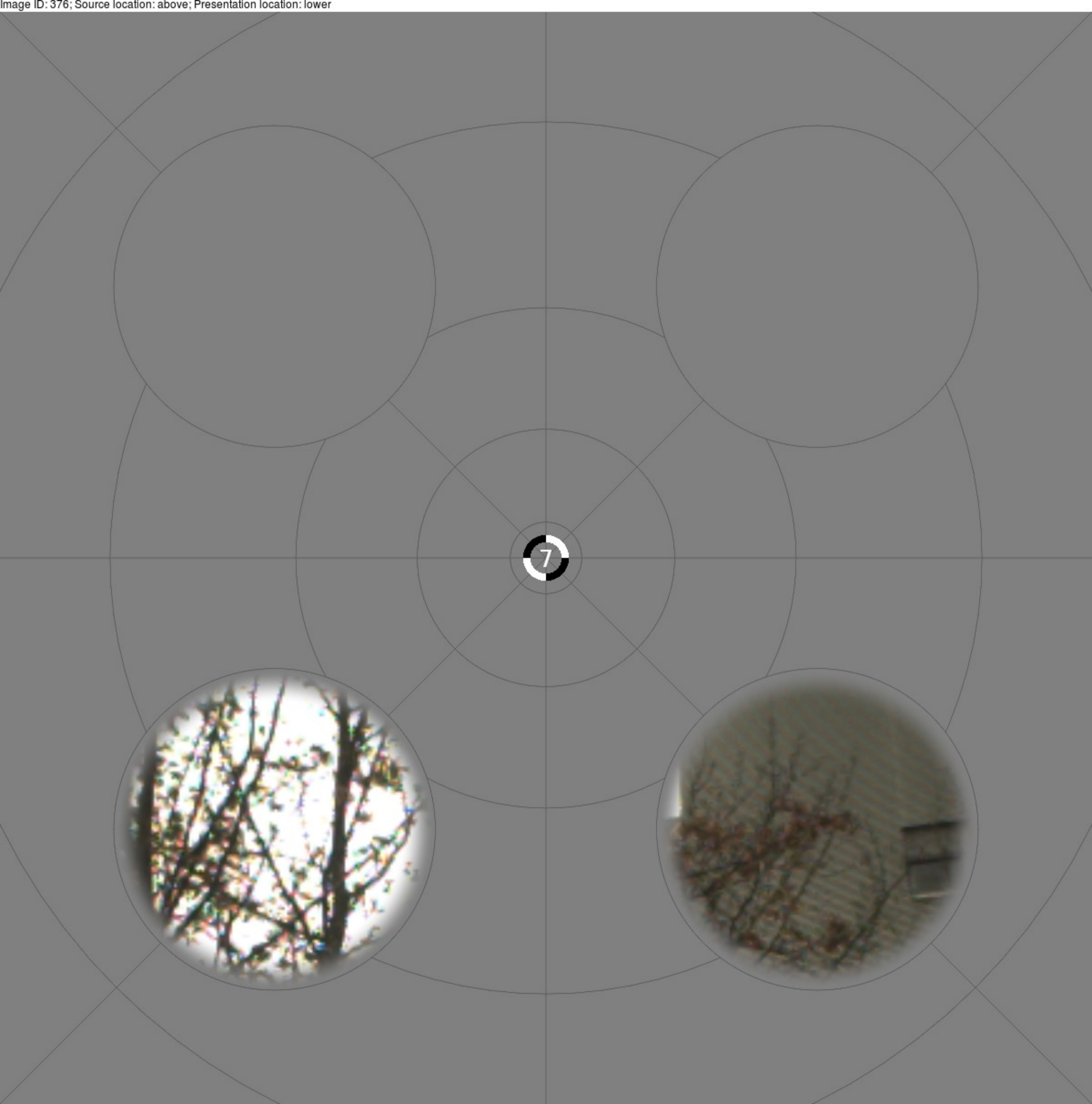

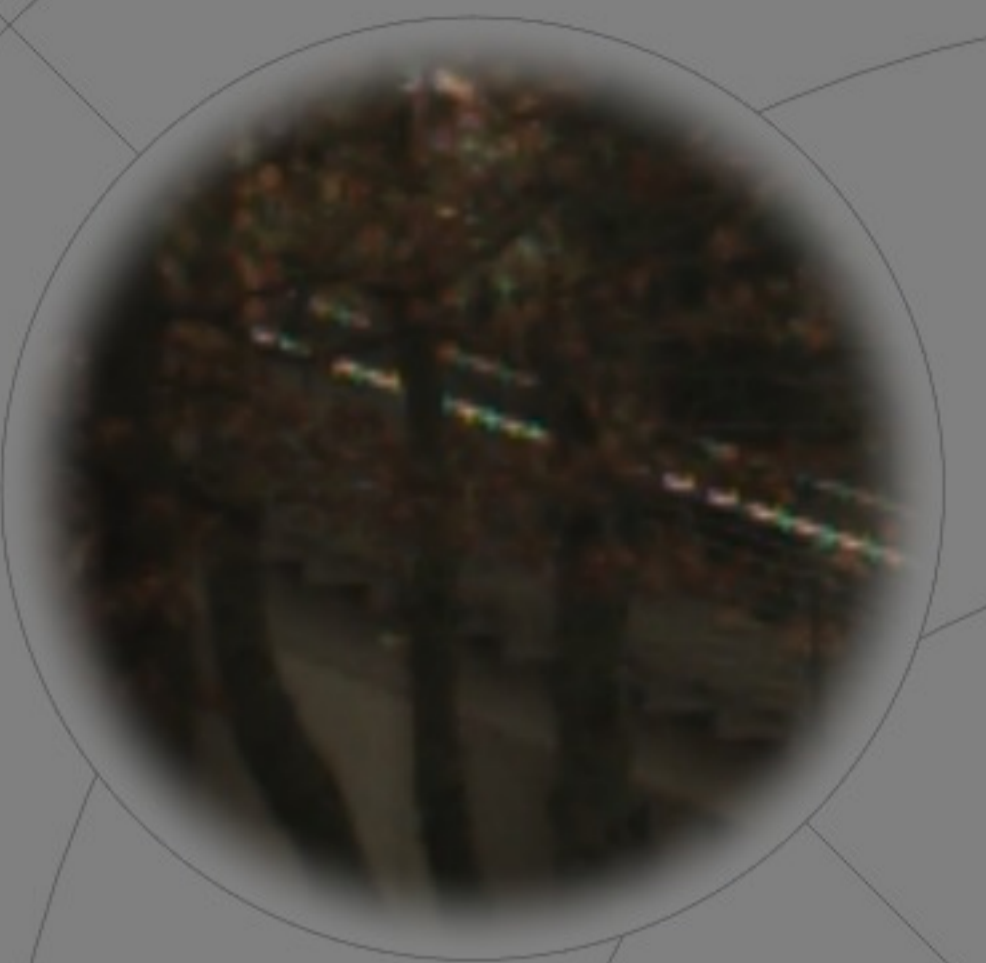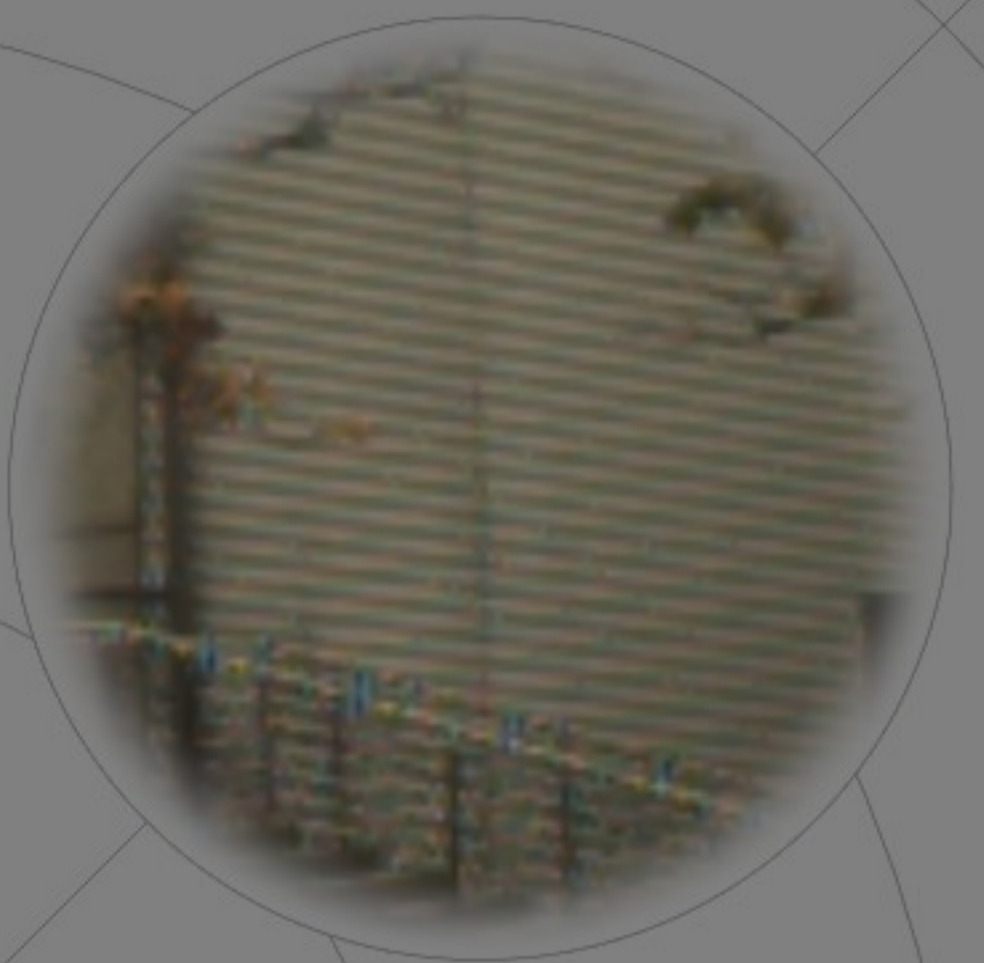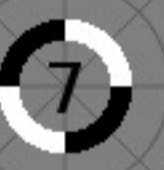

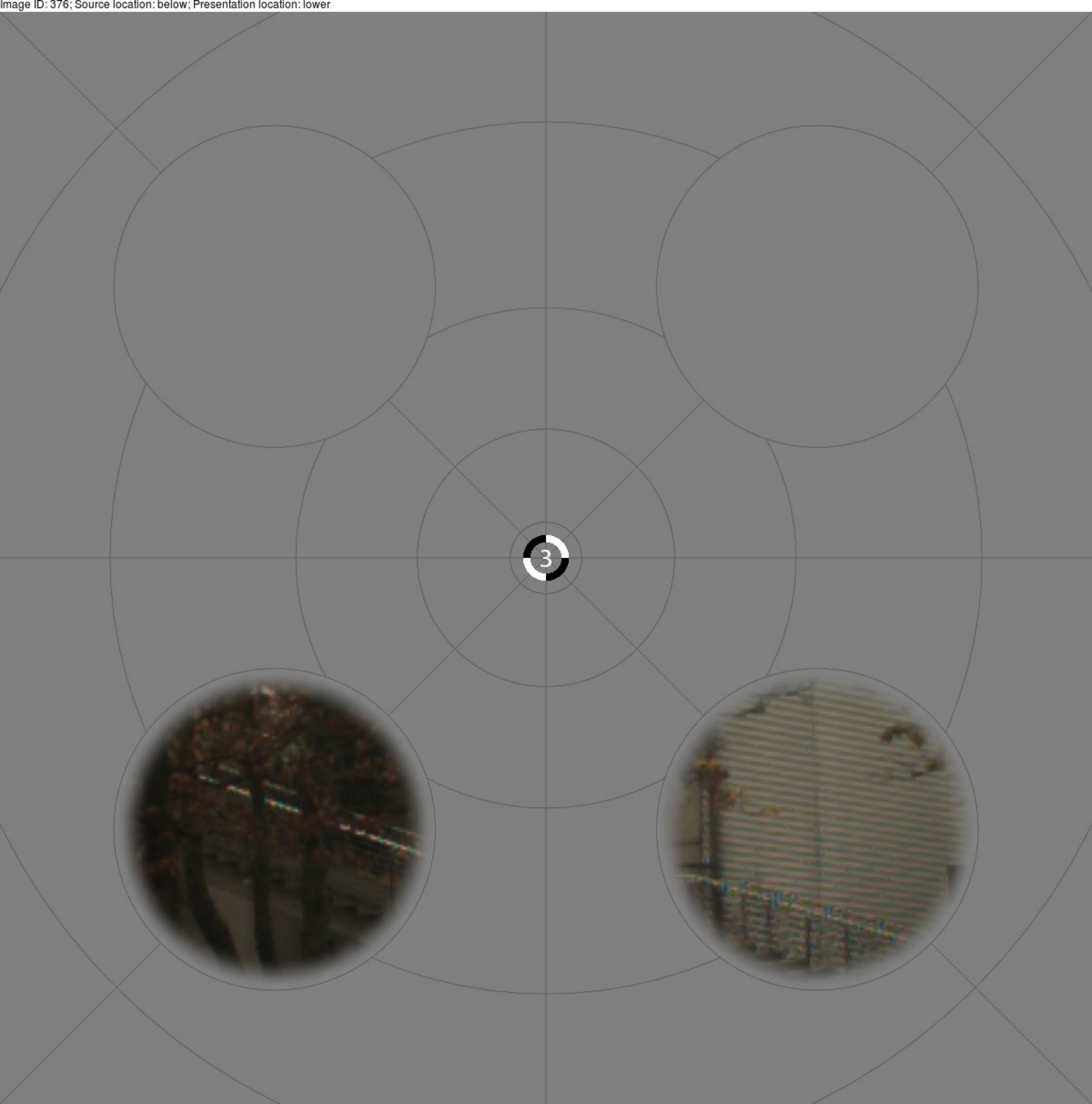

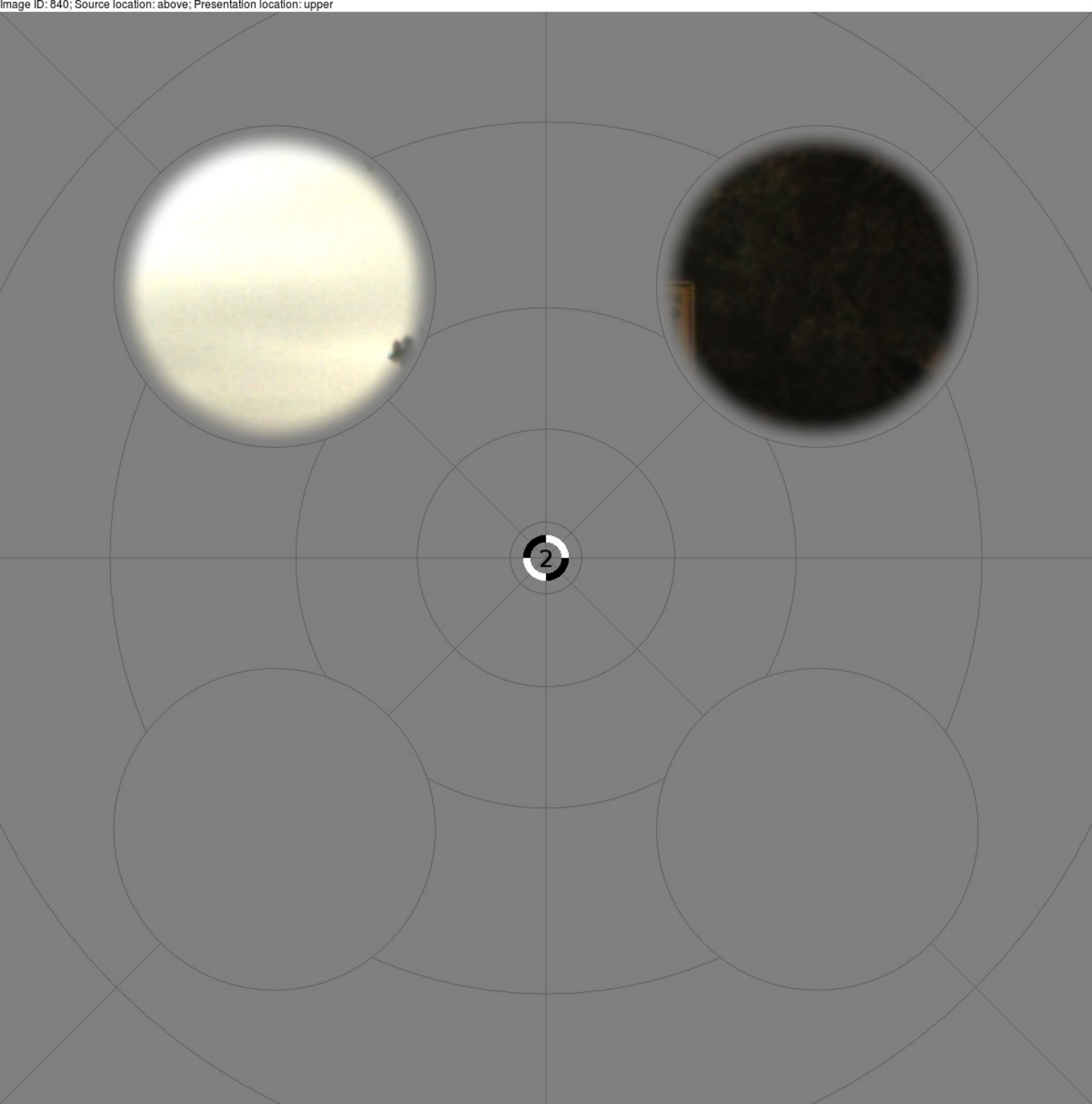

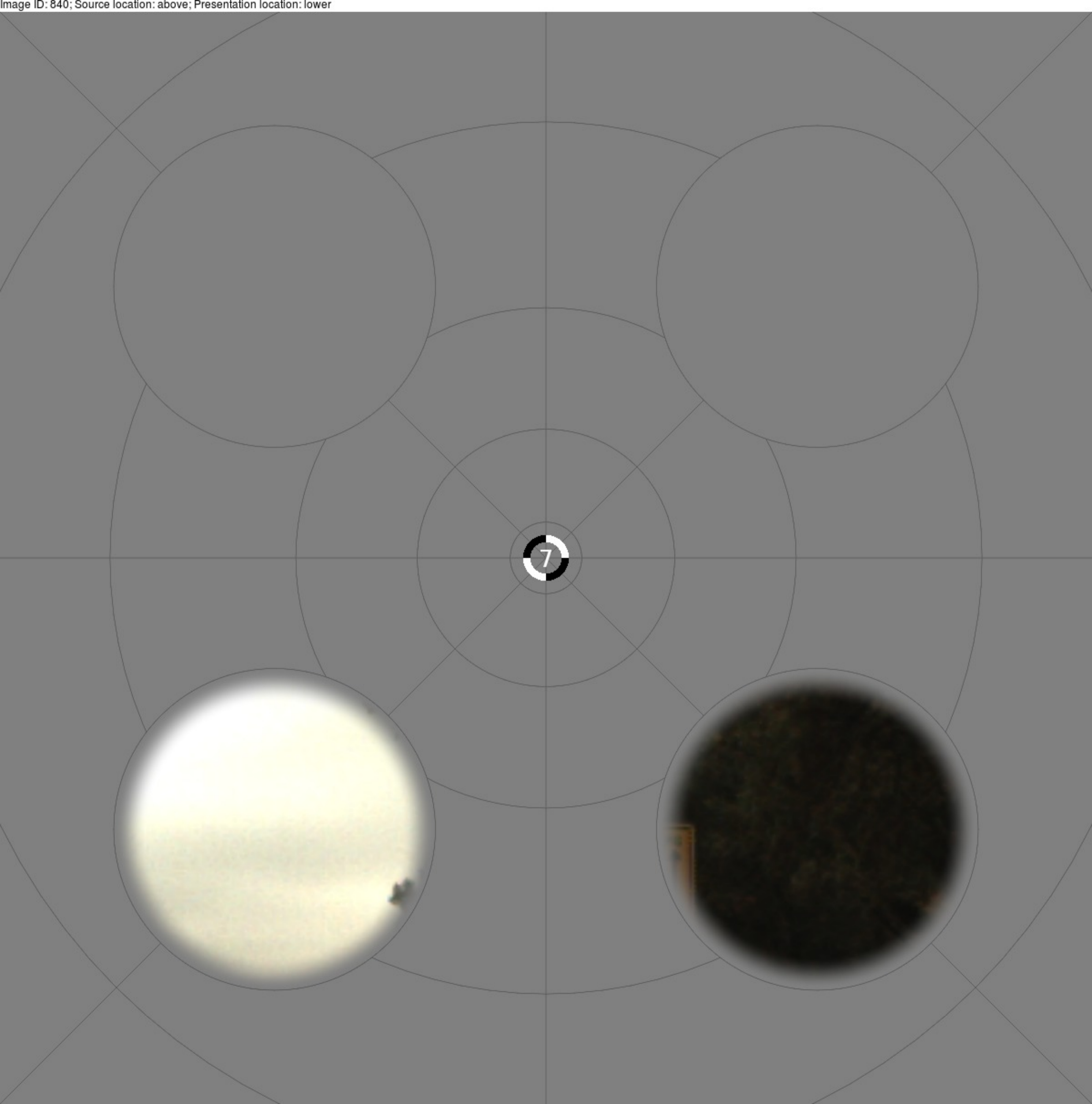

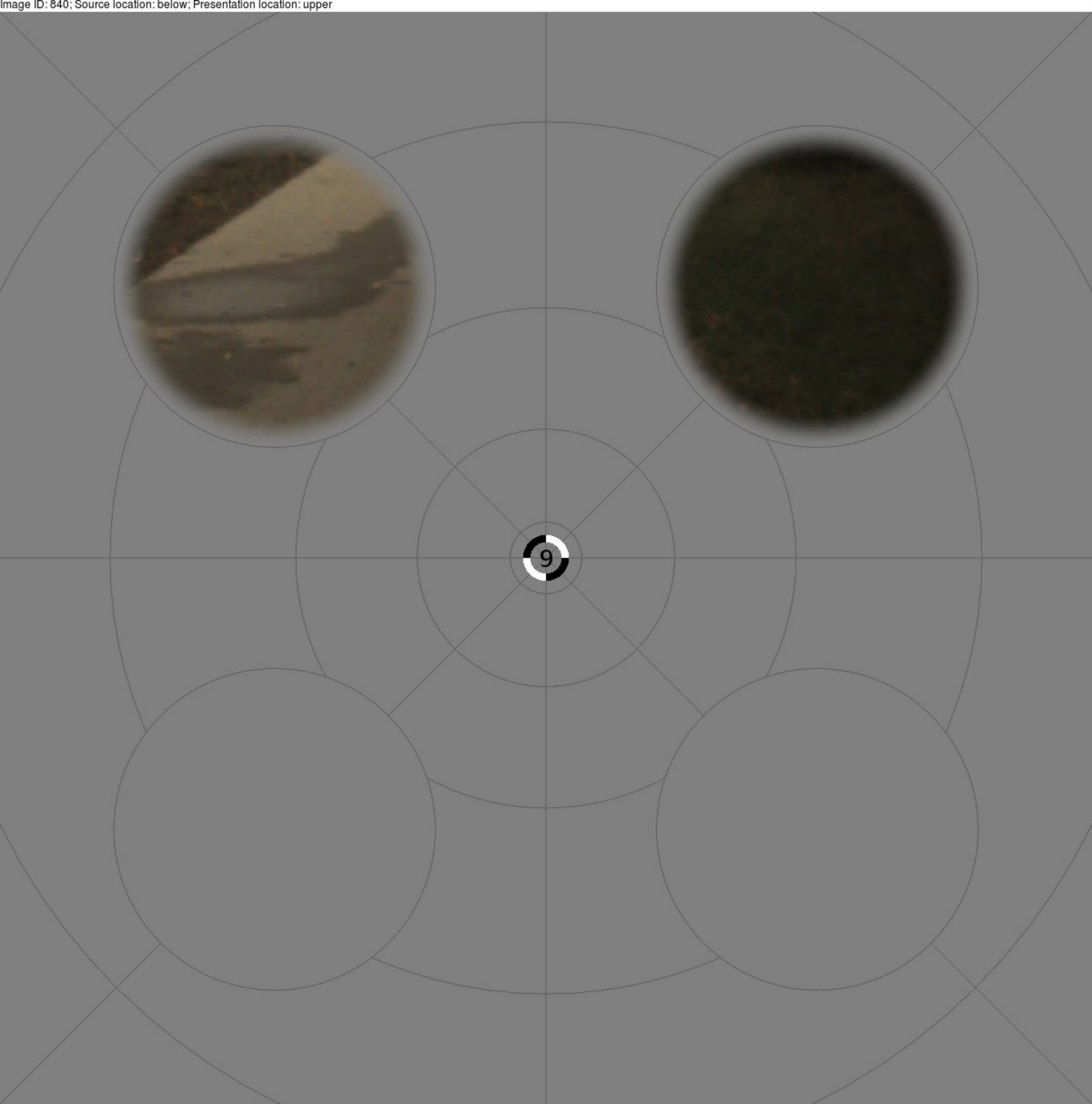

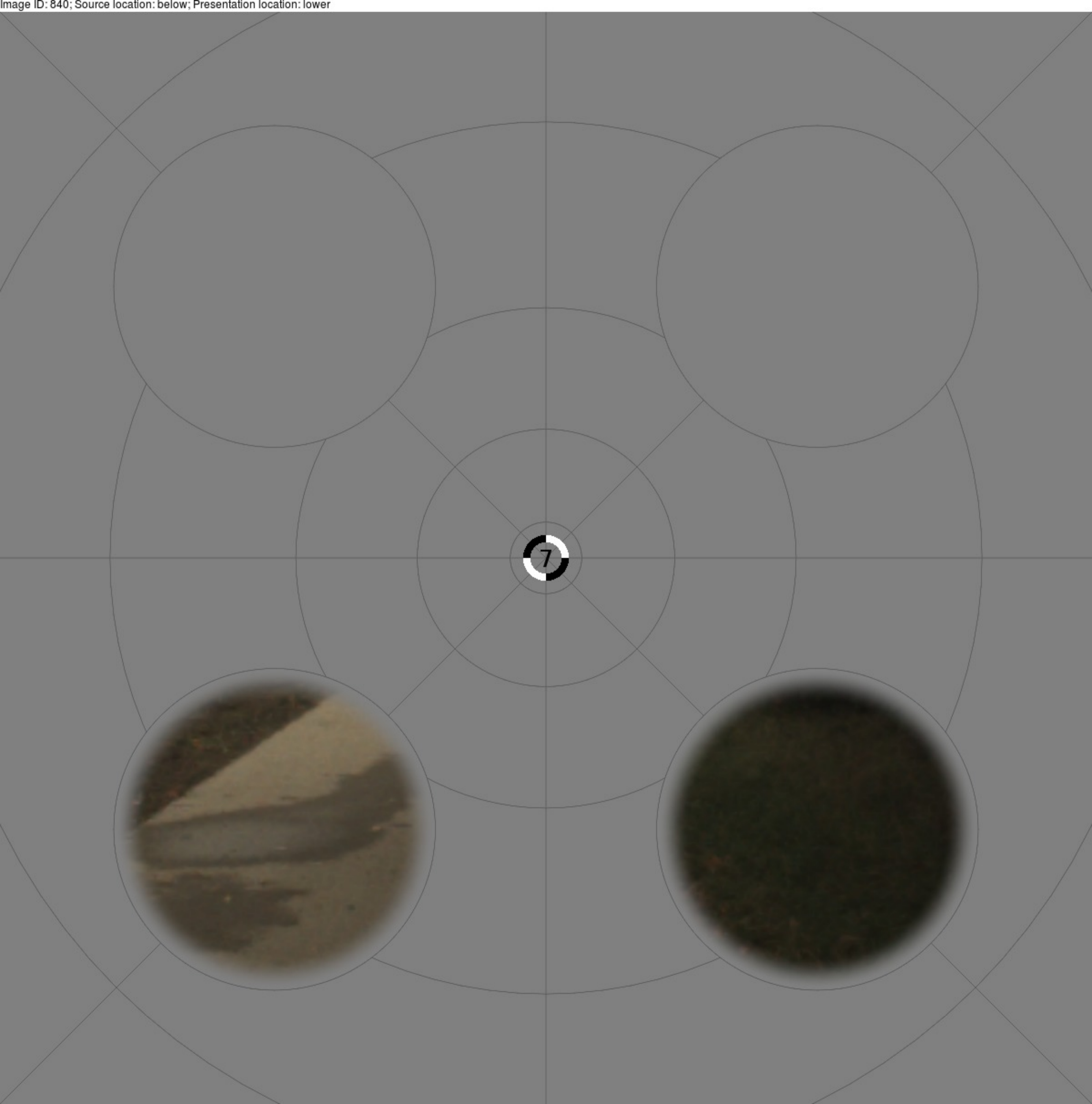

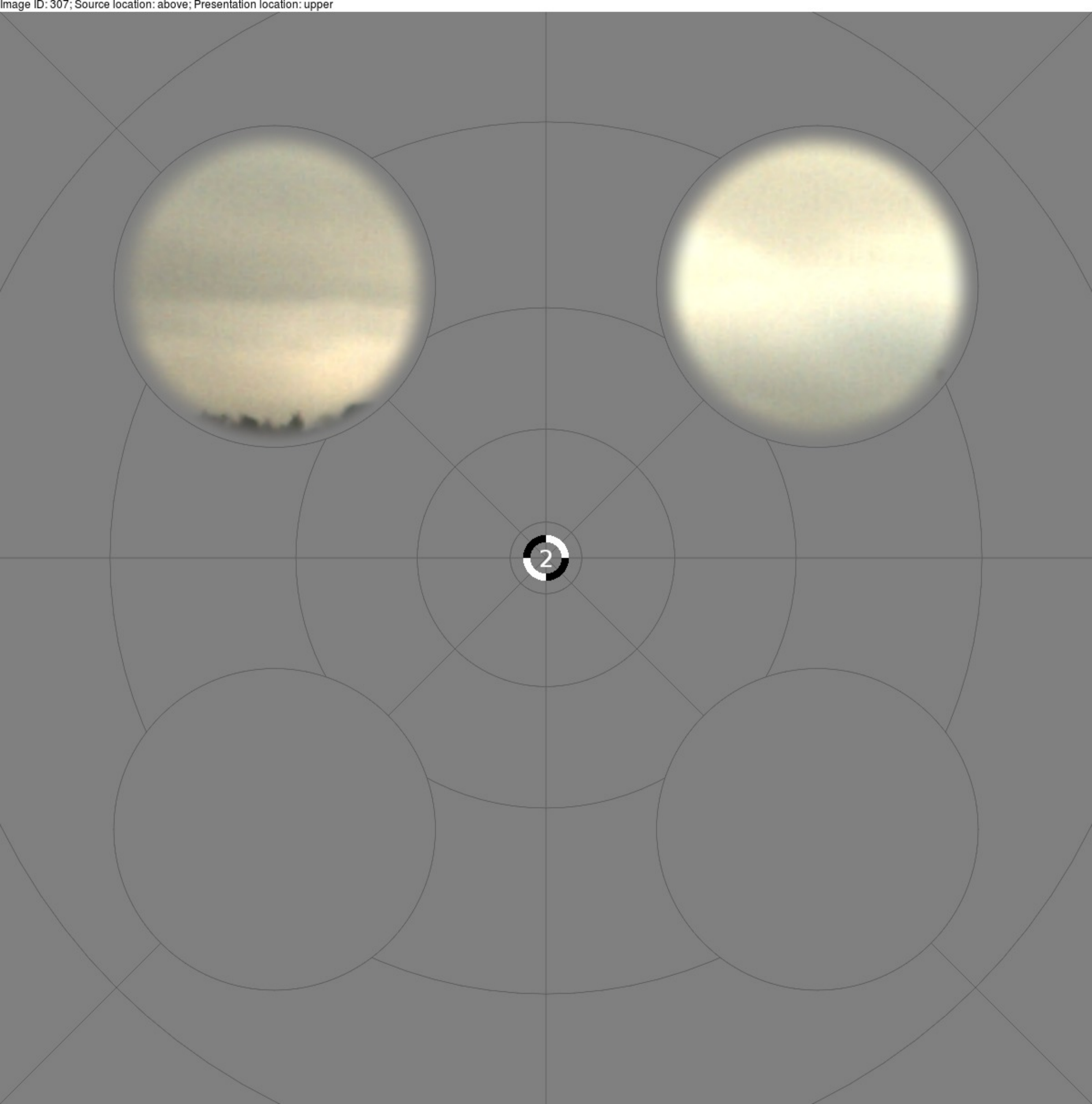

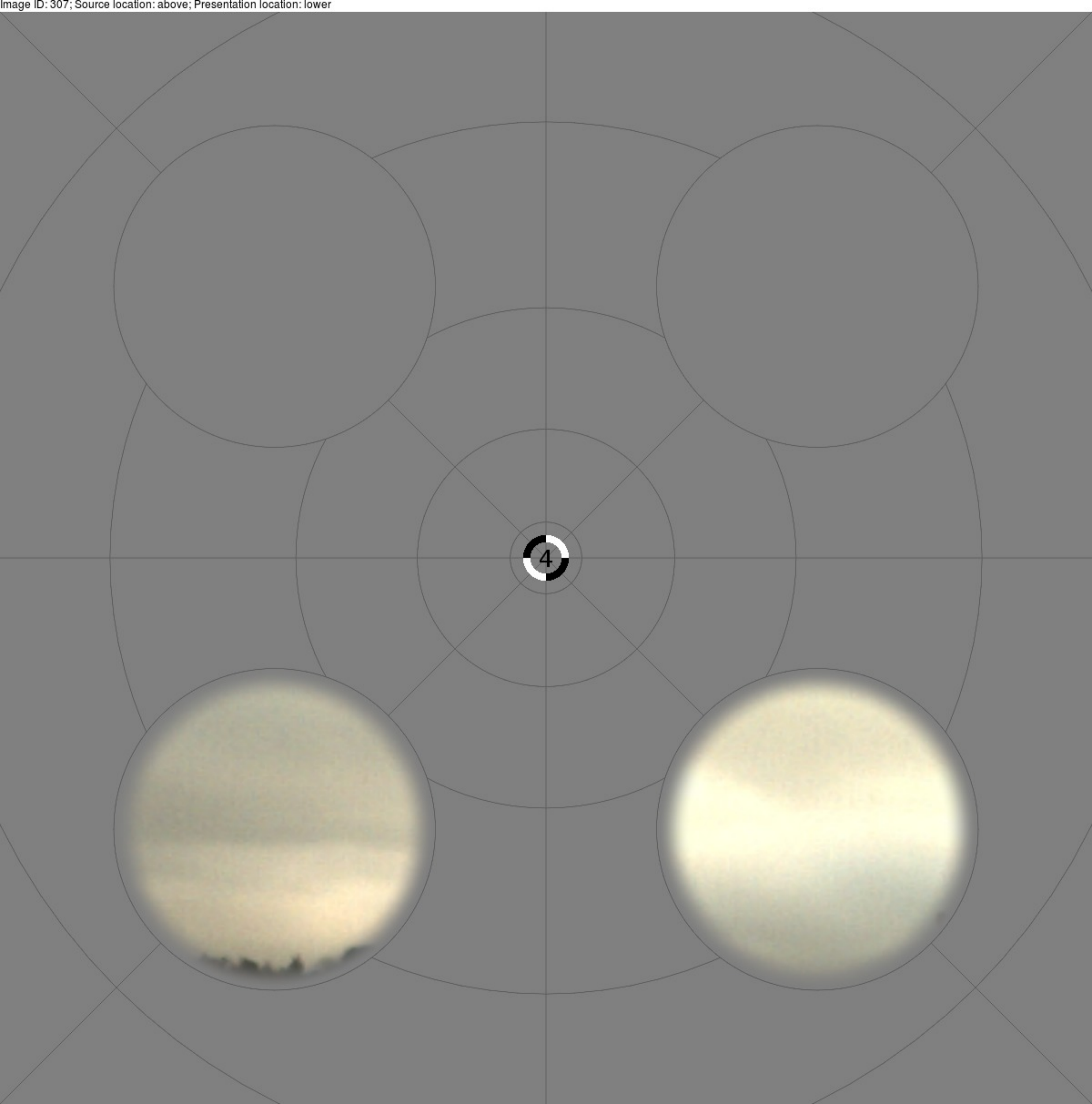

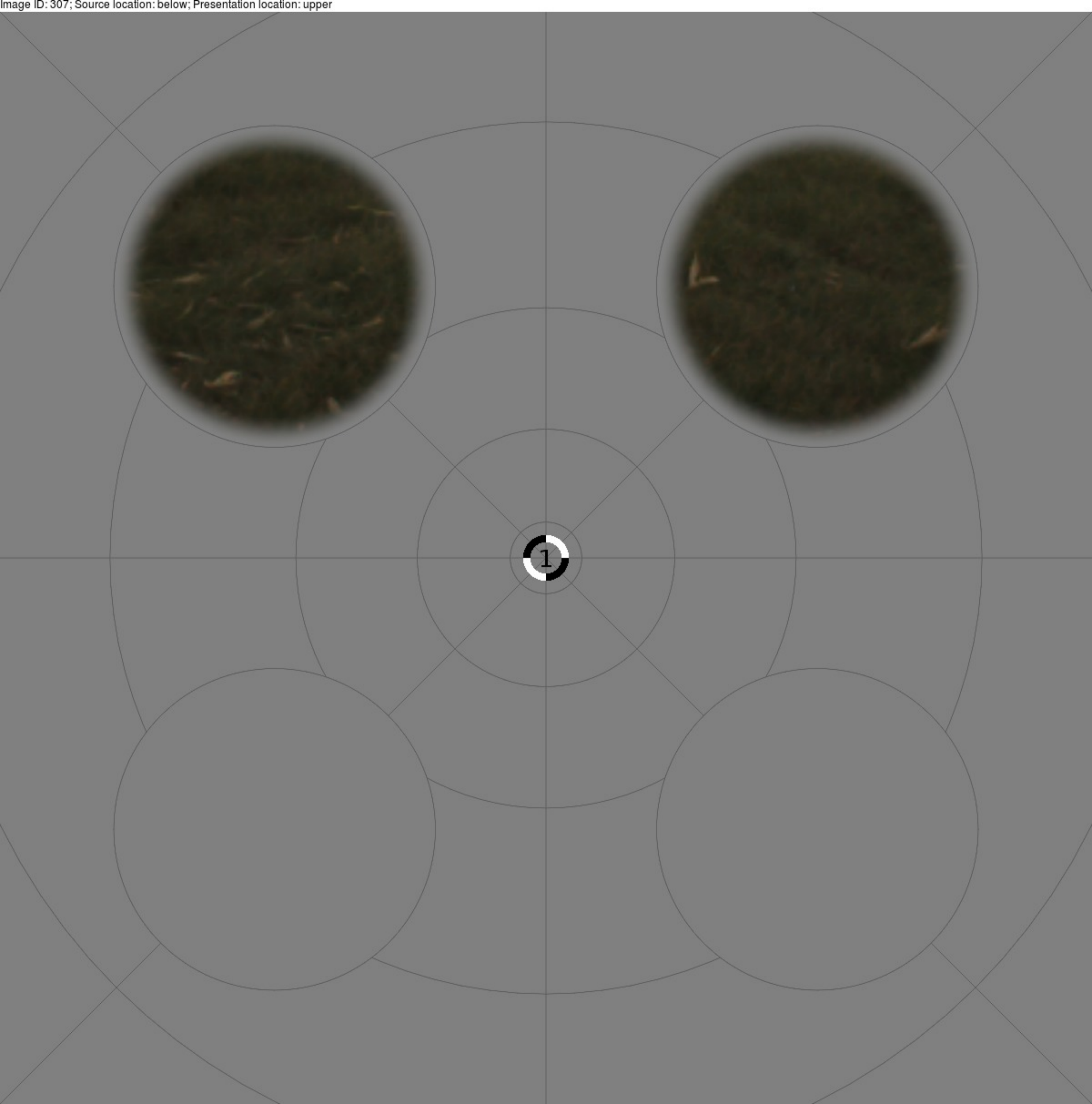

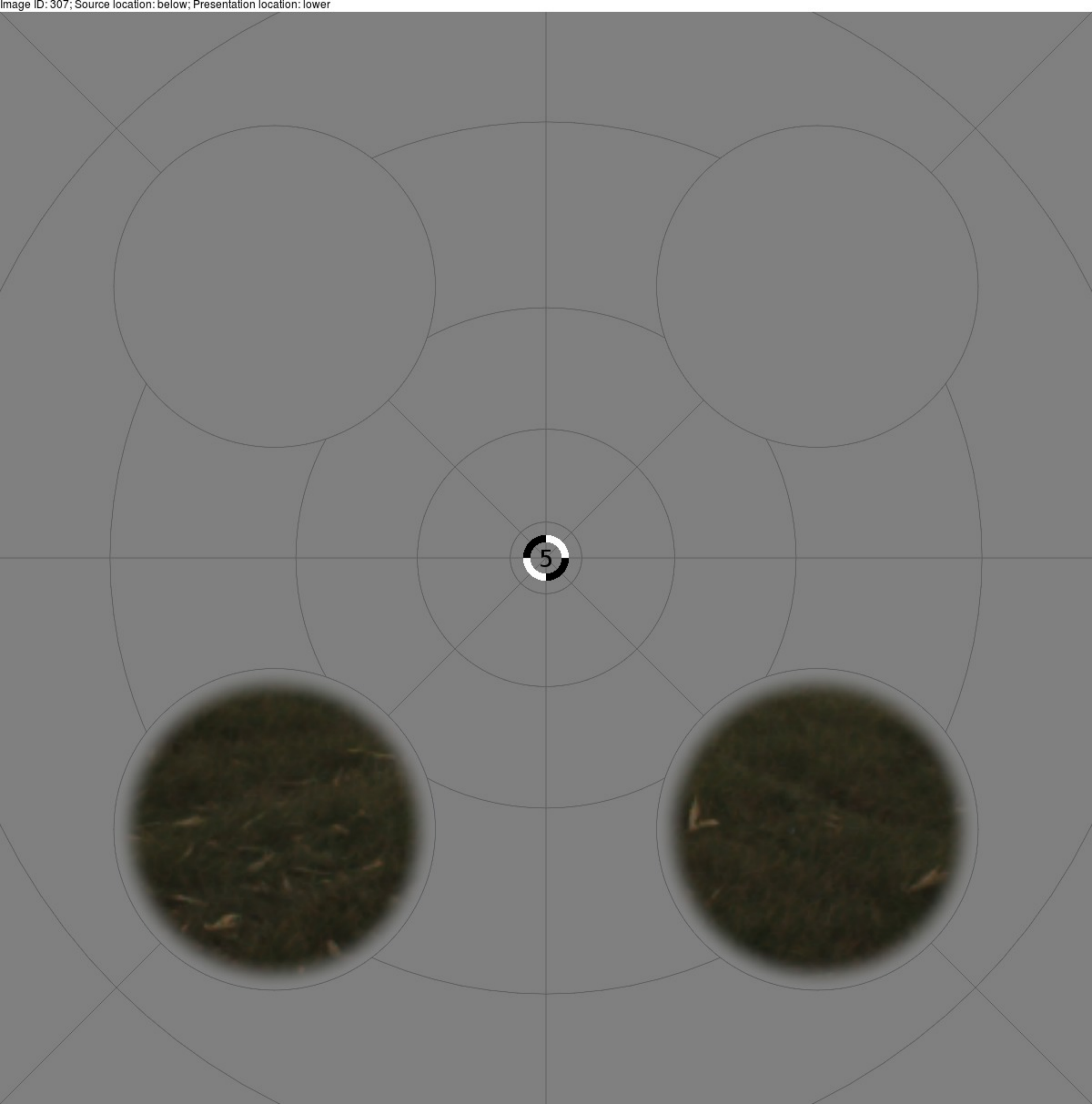

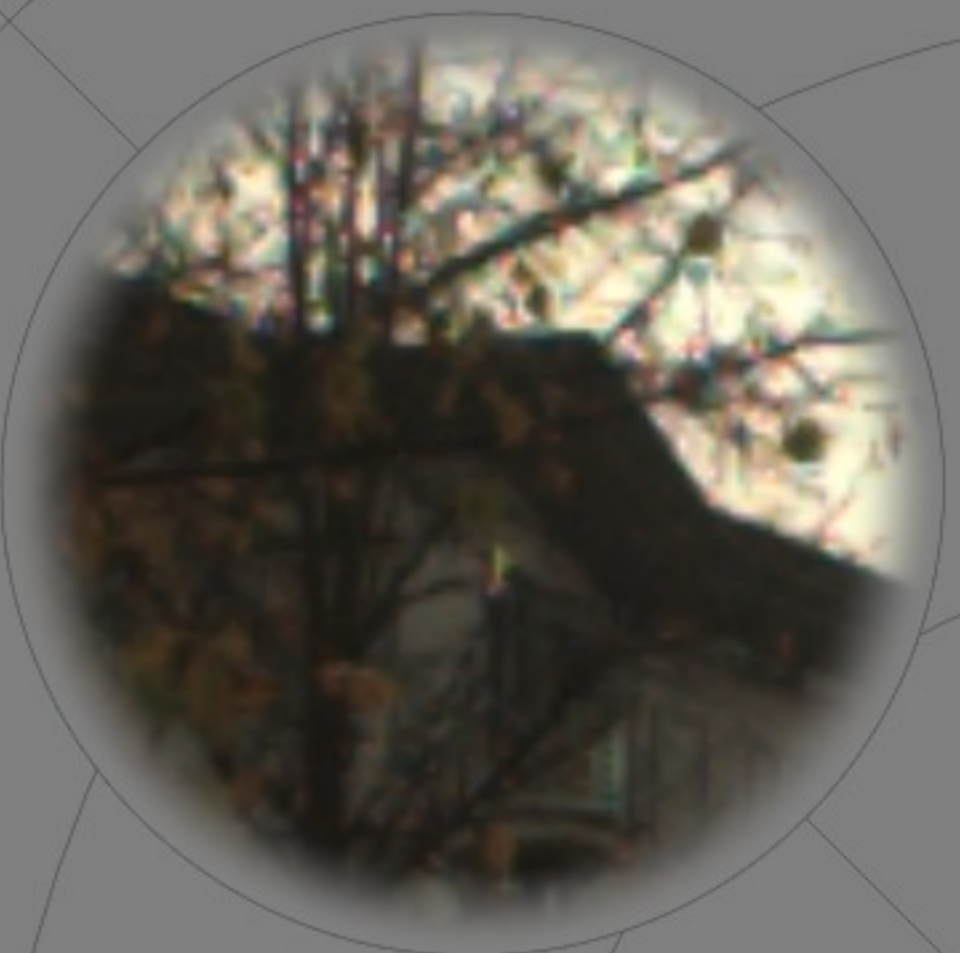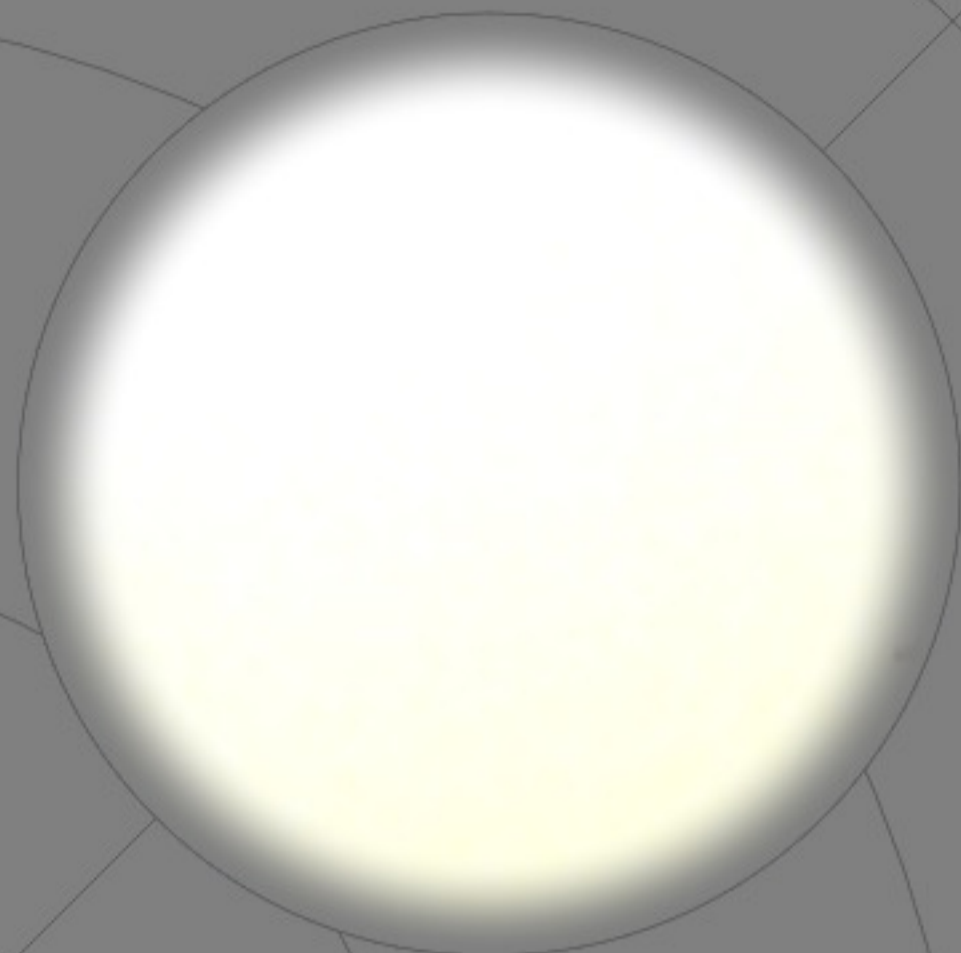

7

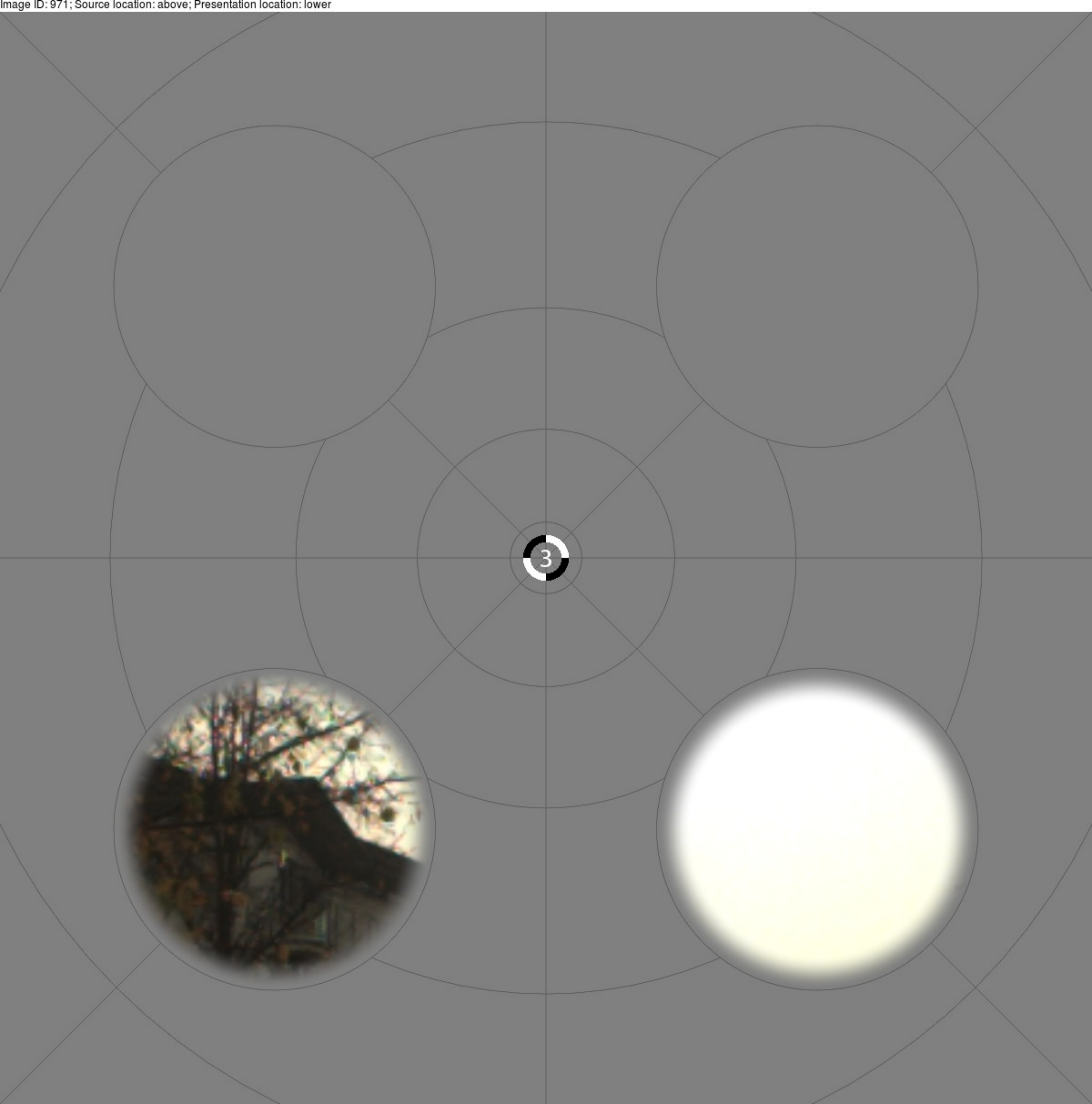

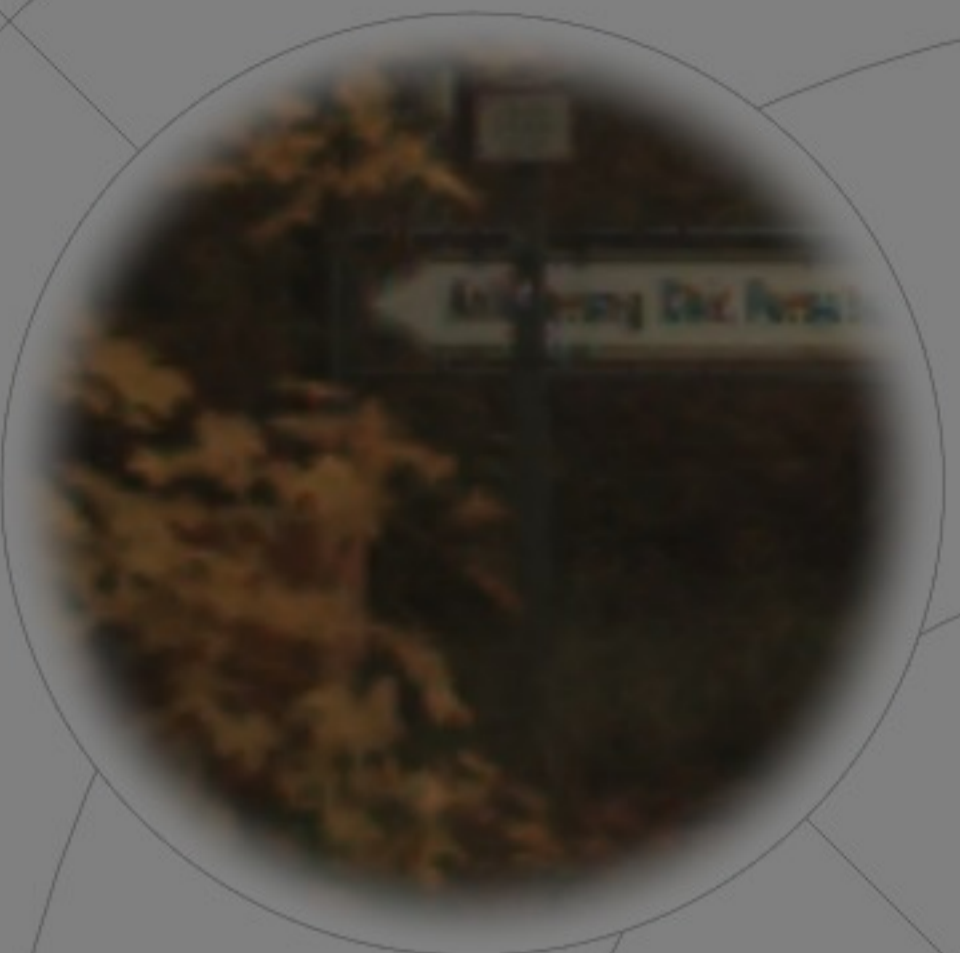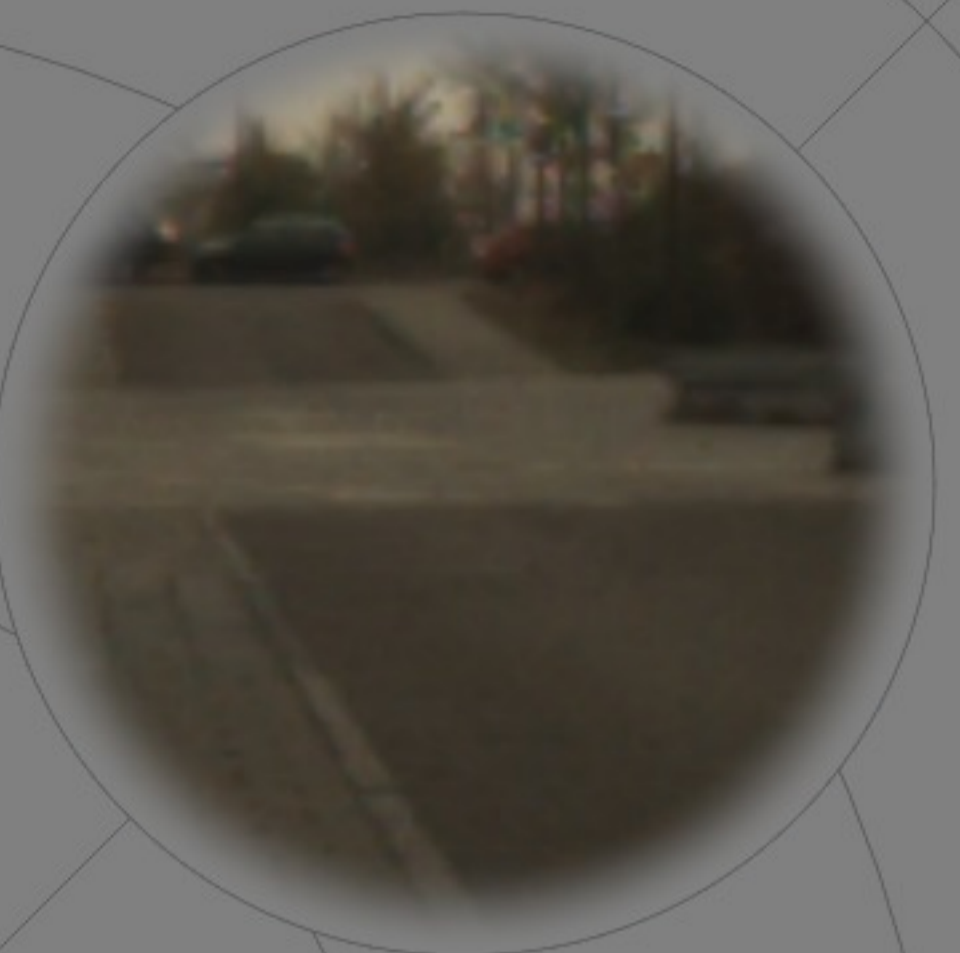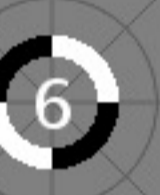

8

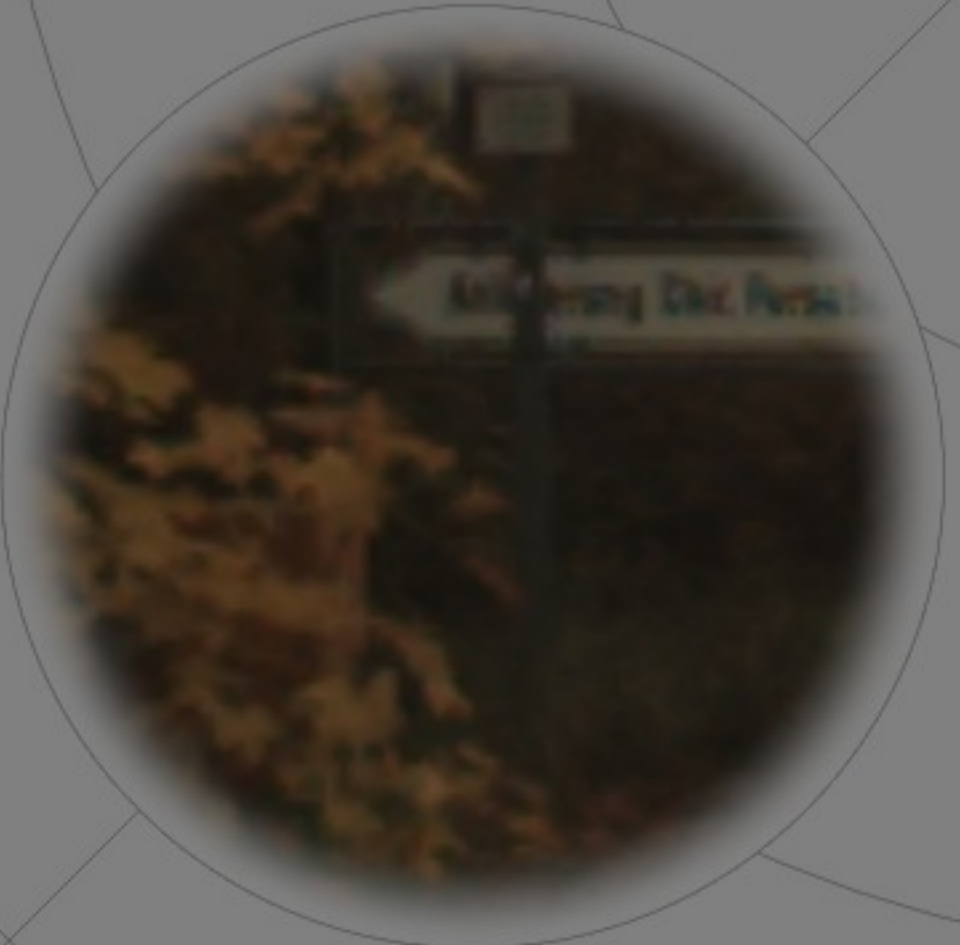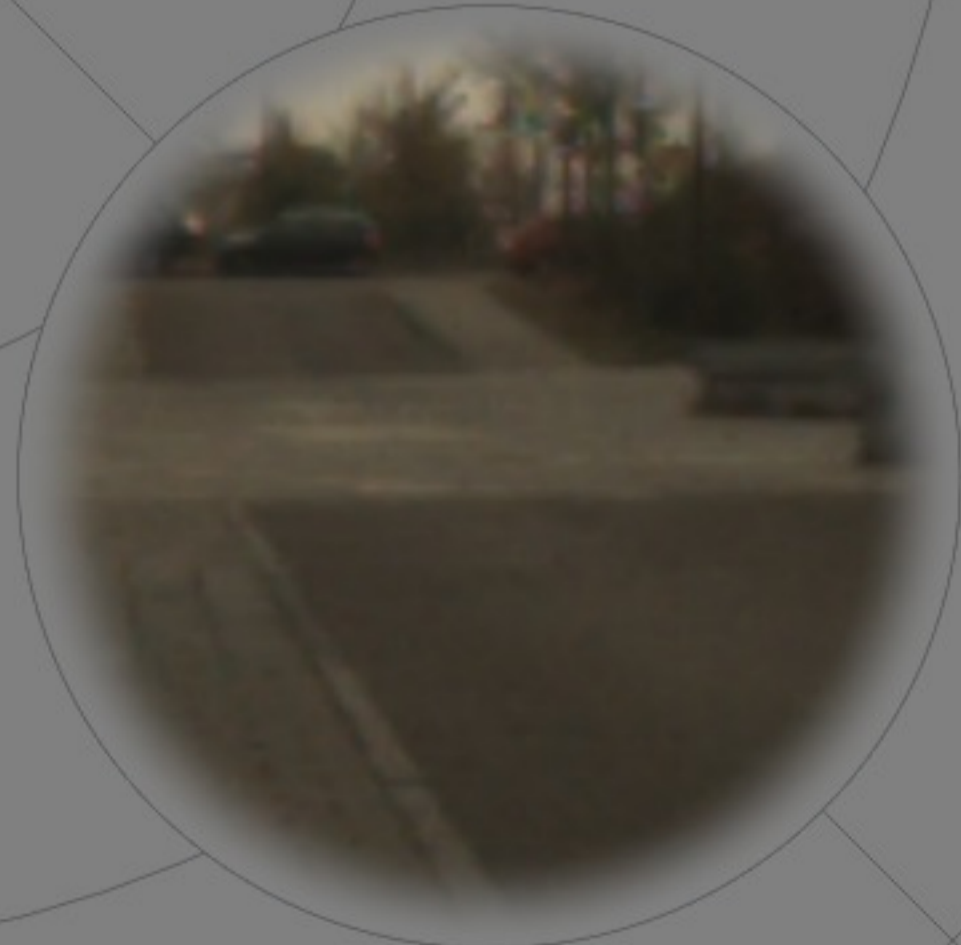

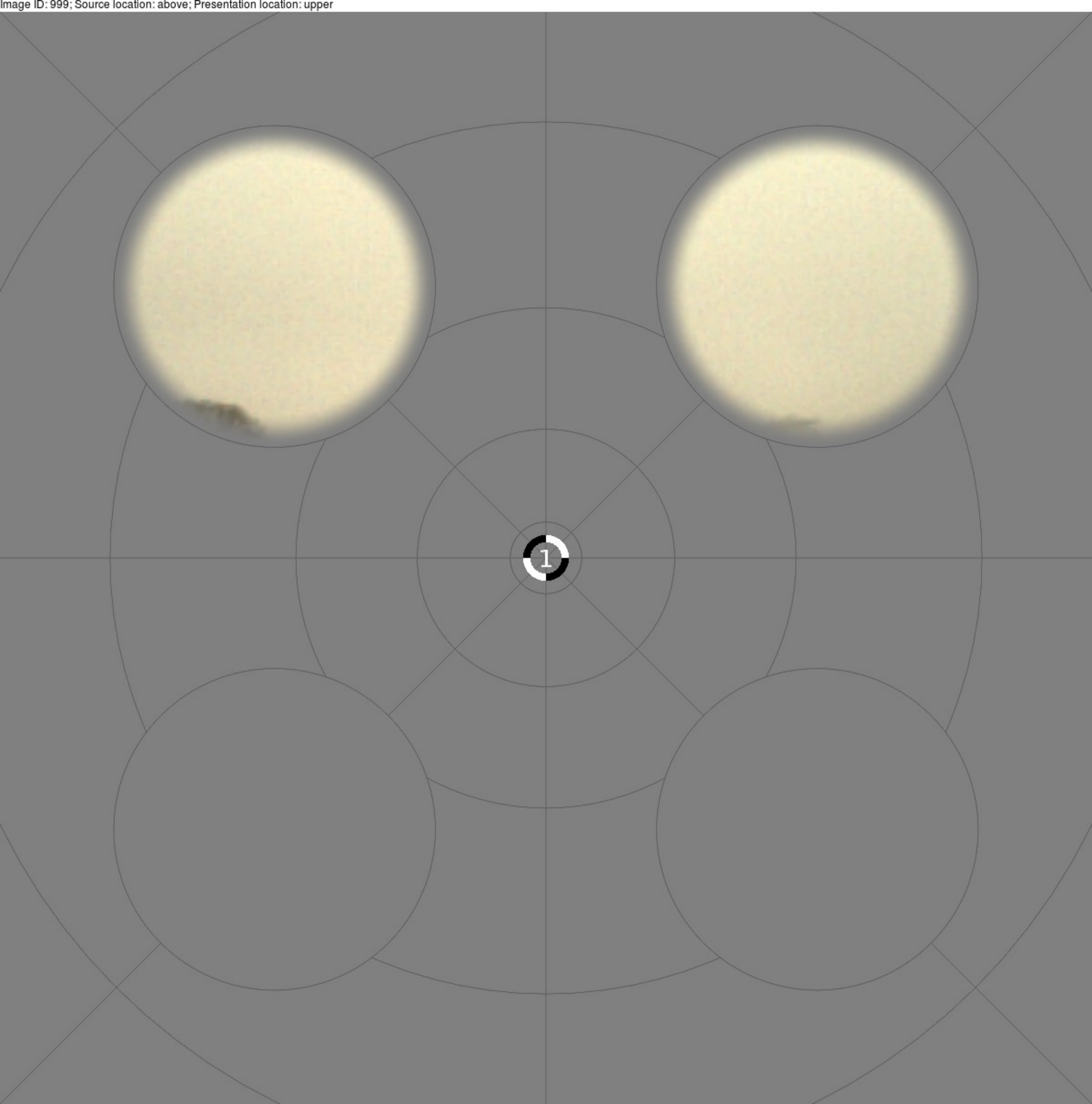

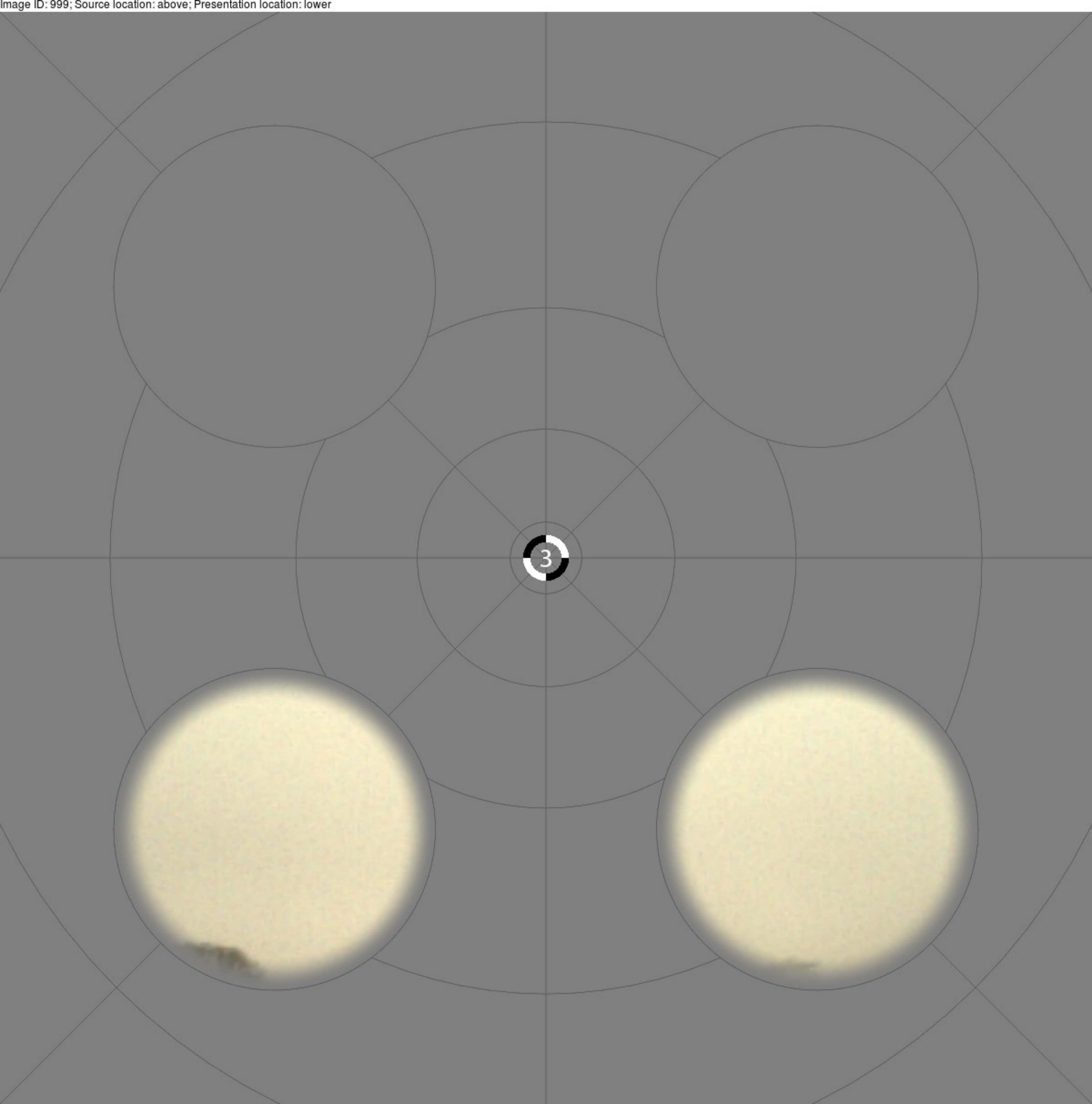

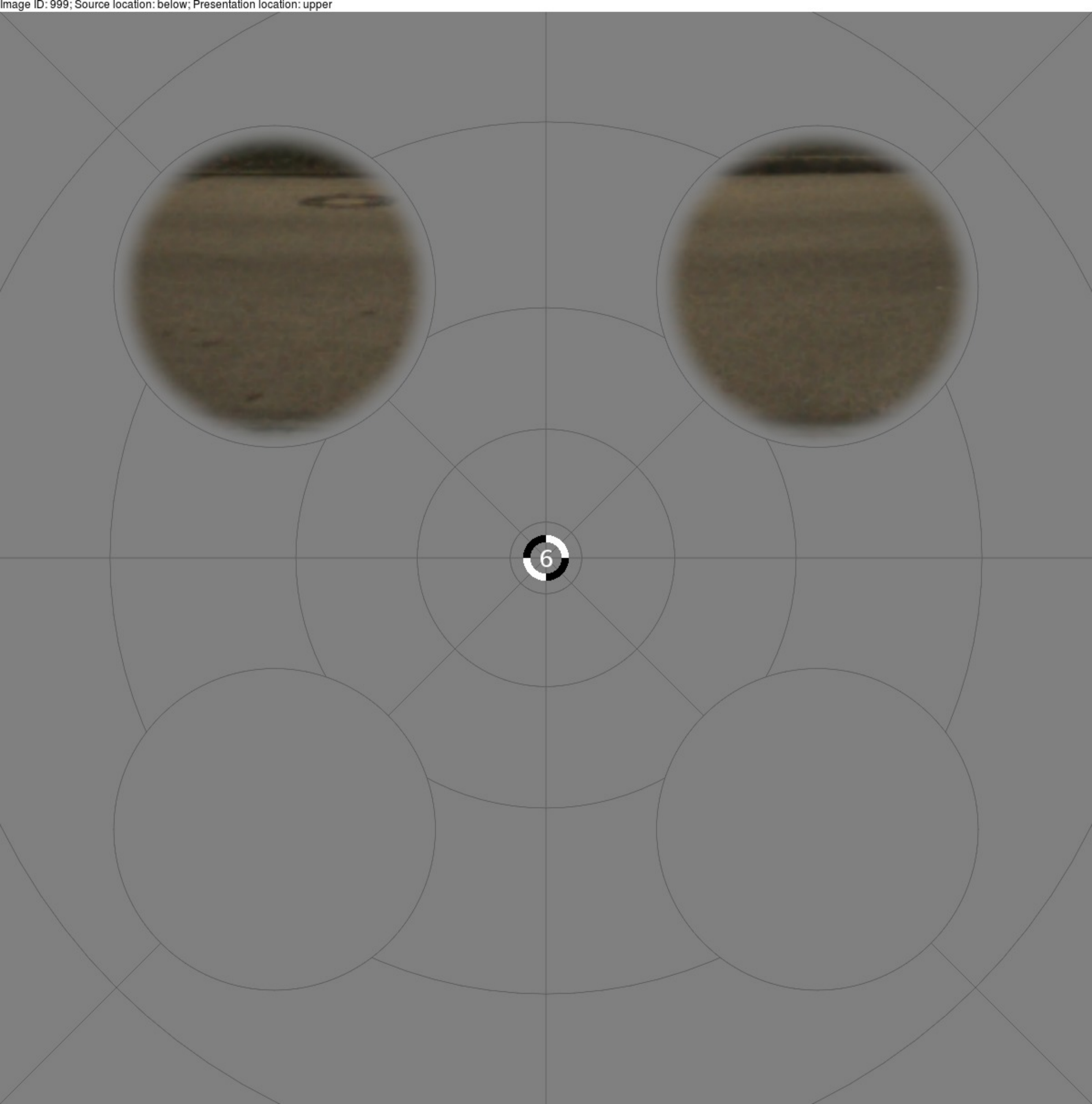

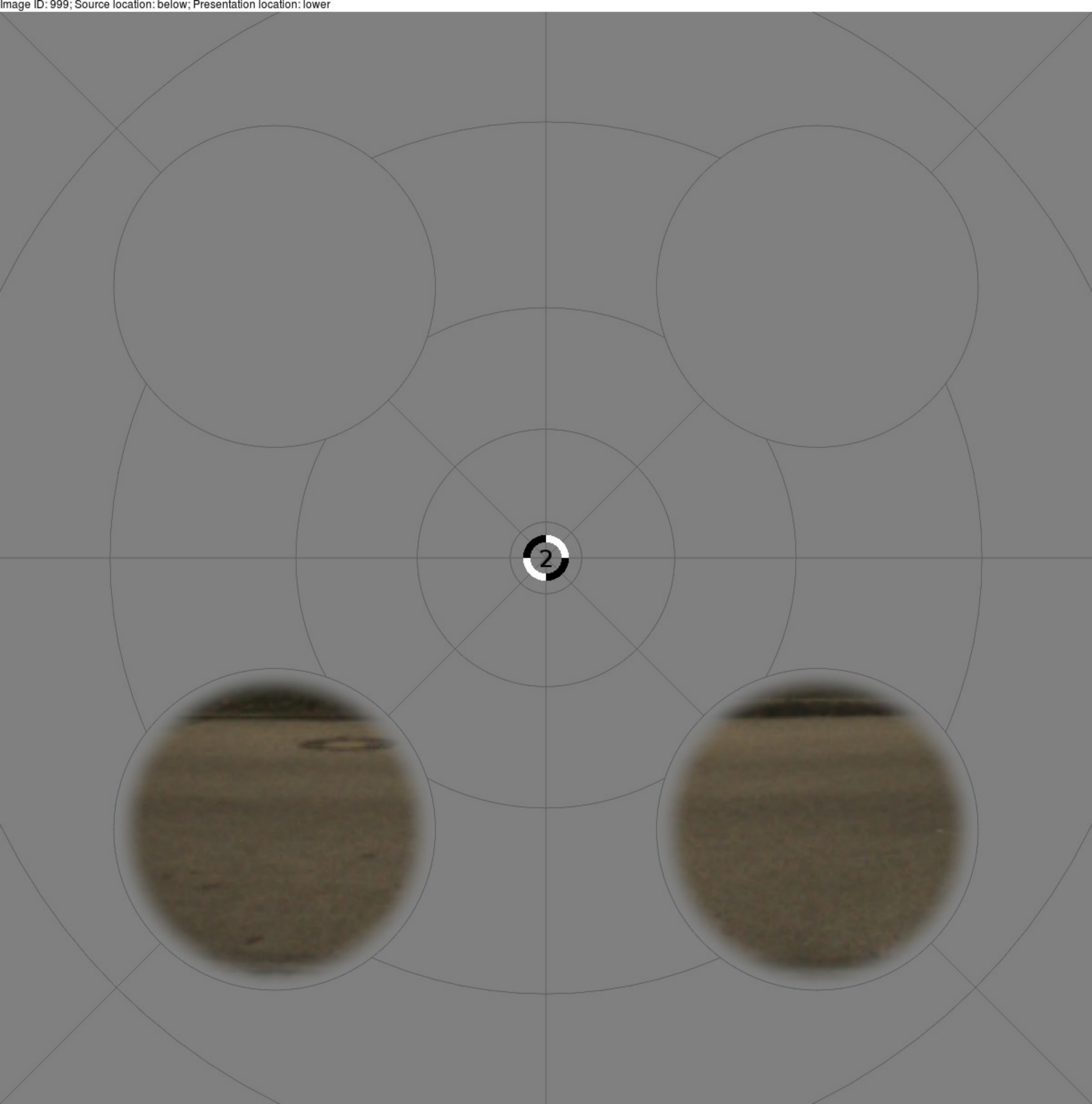

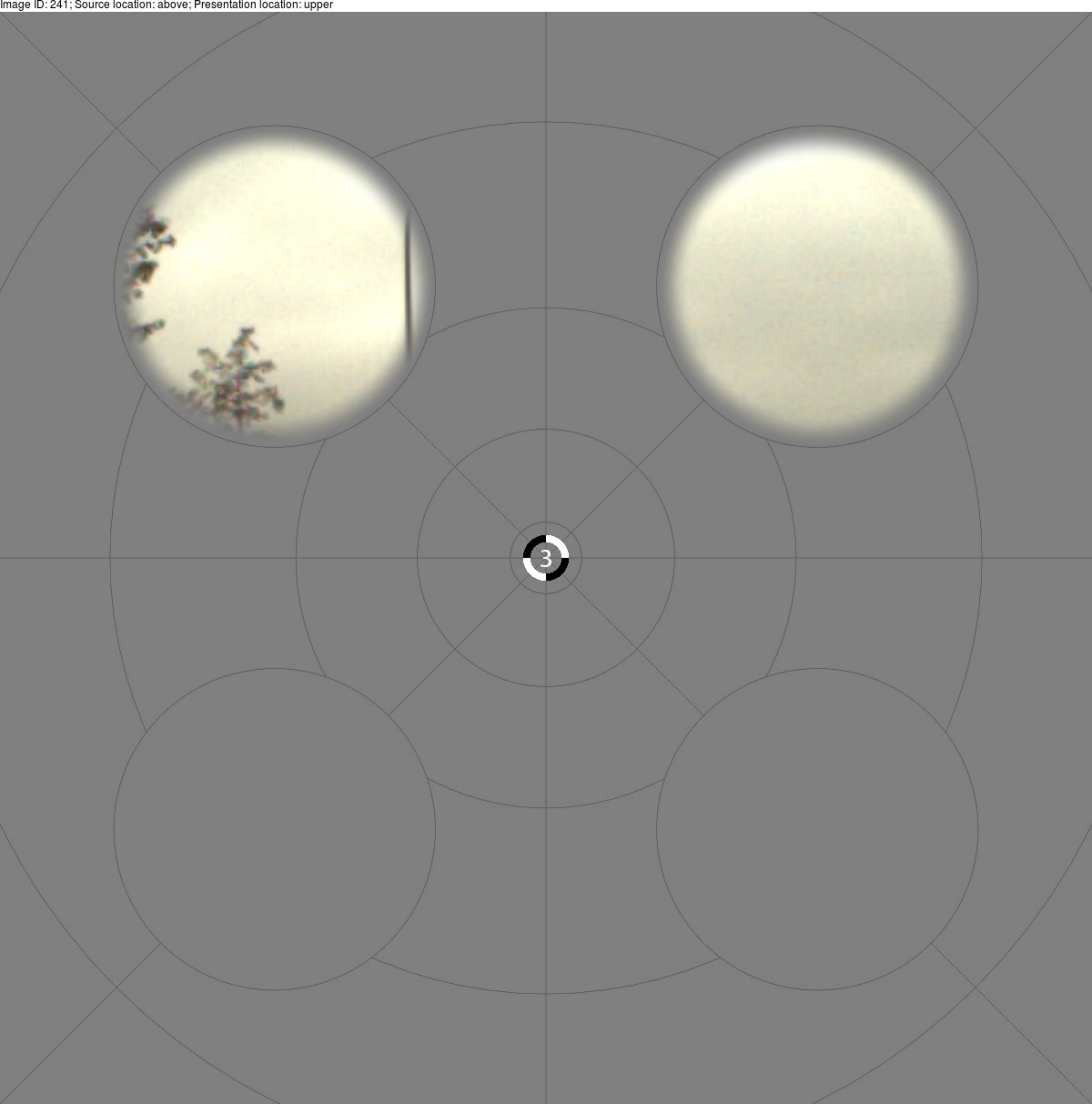

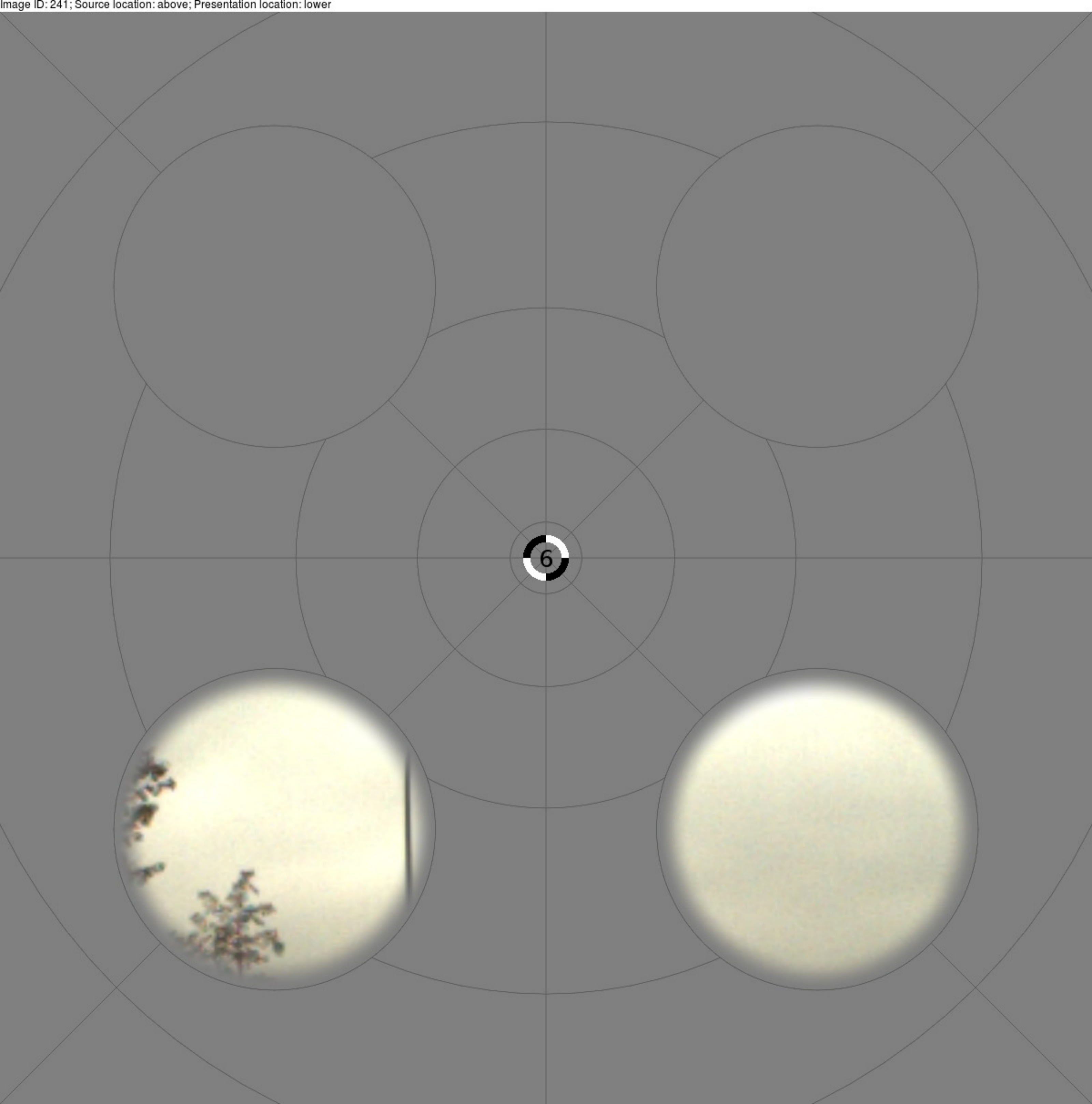

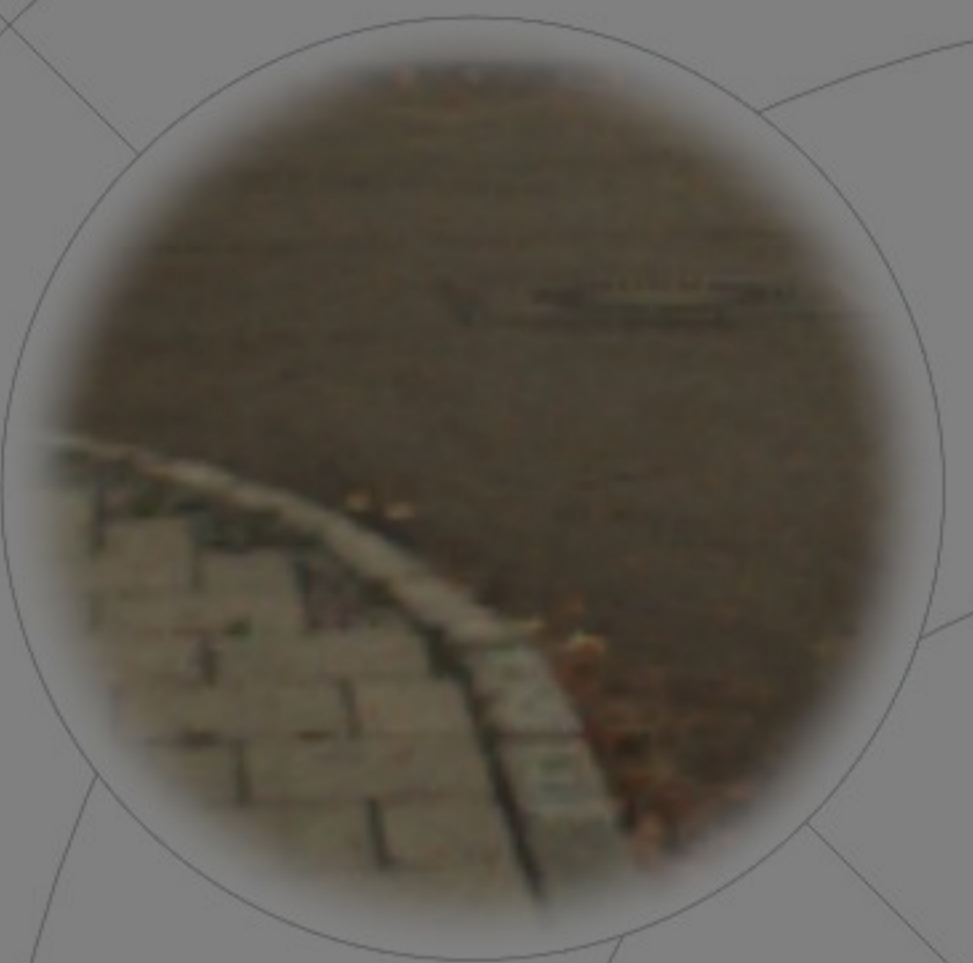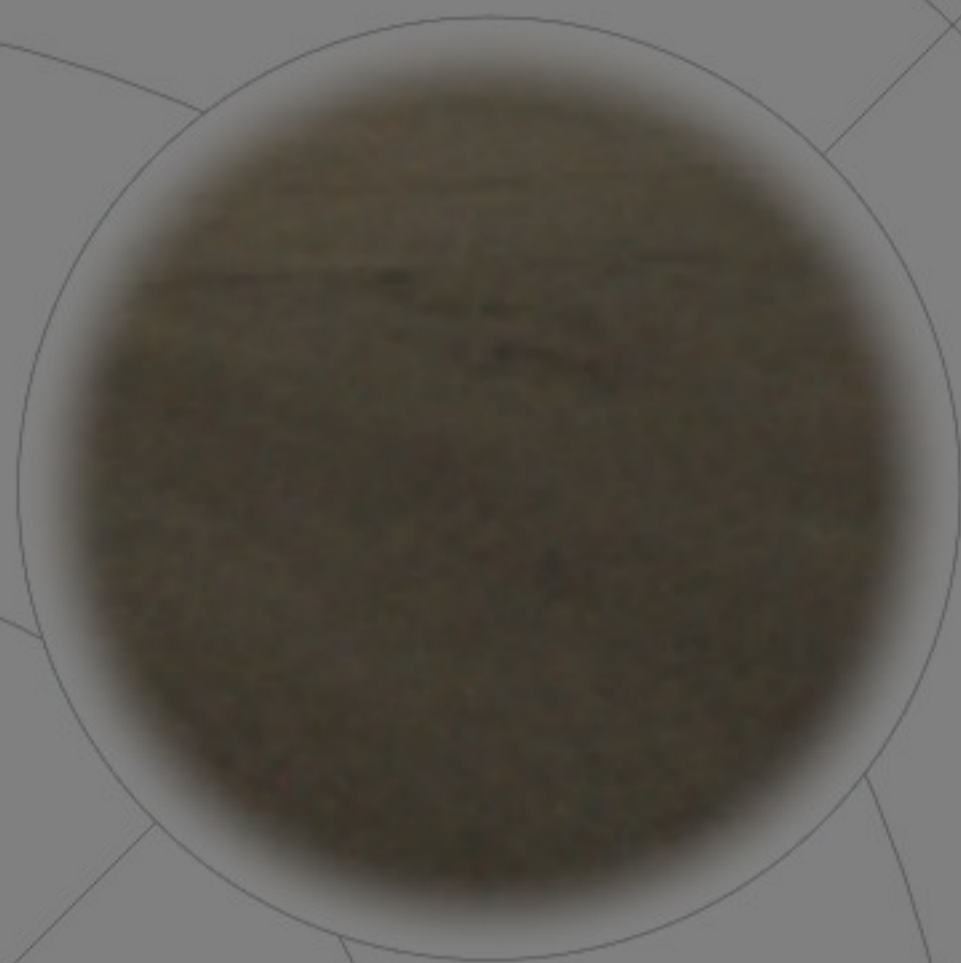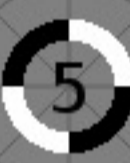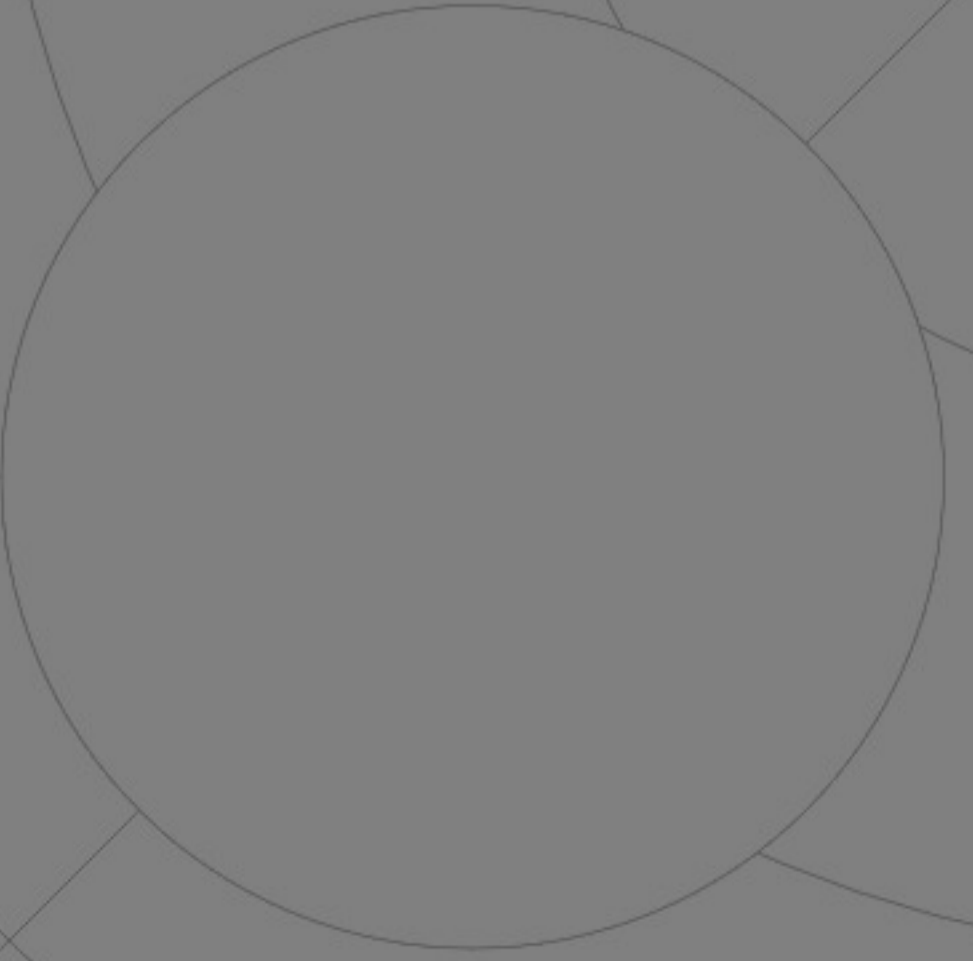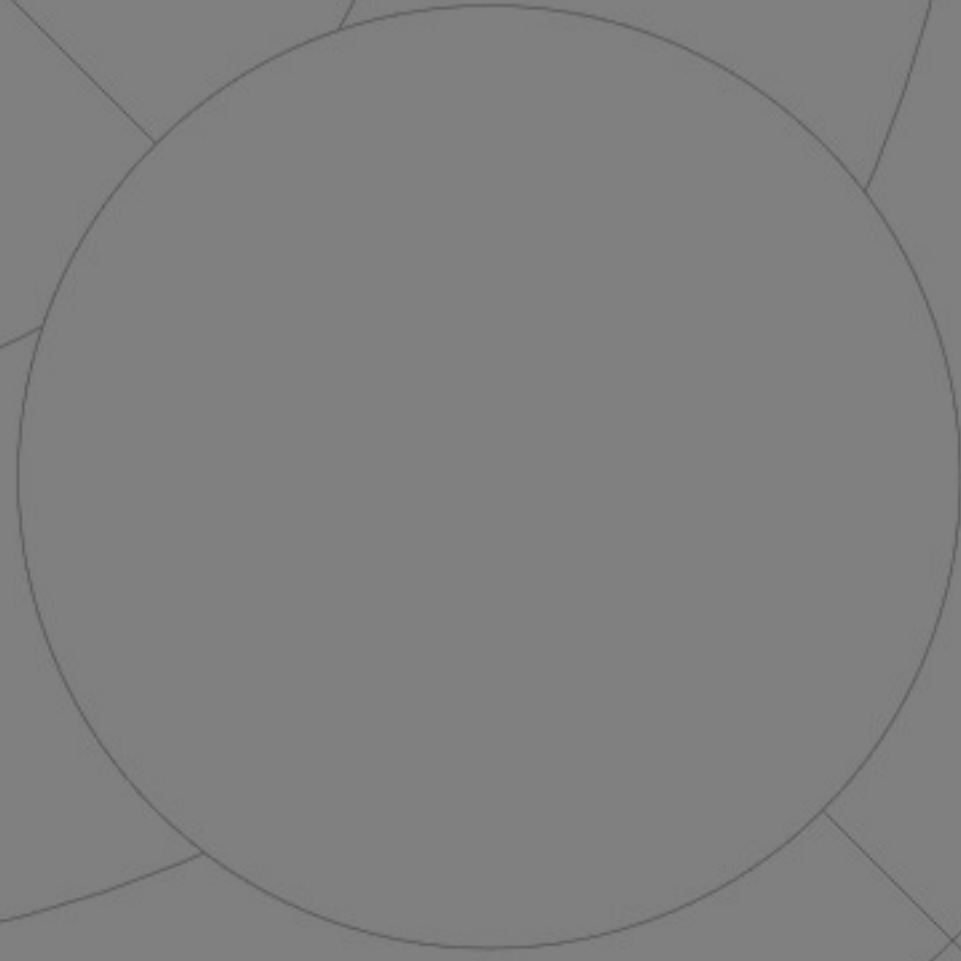

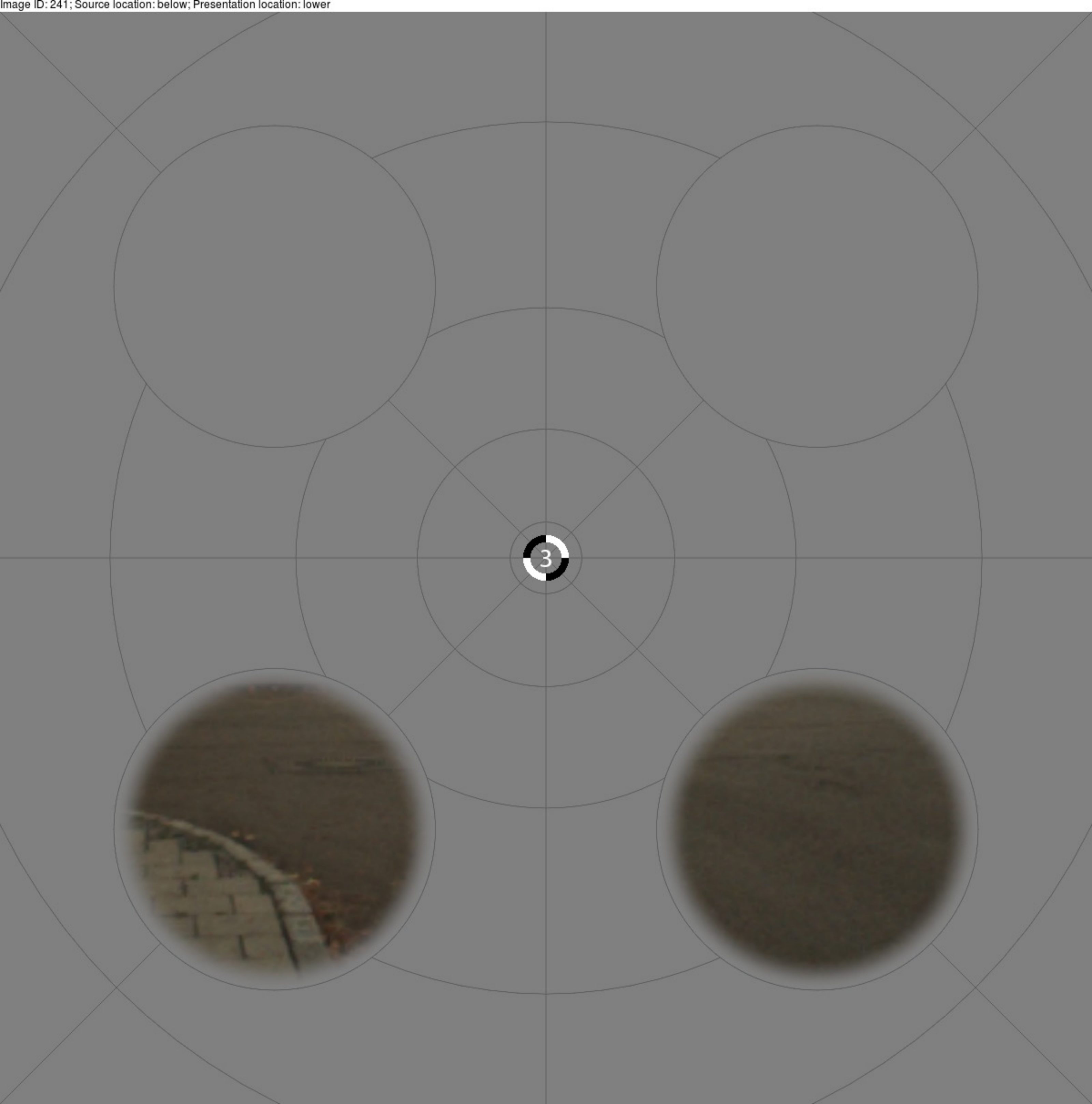

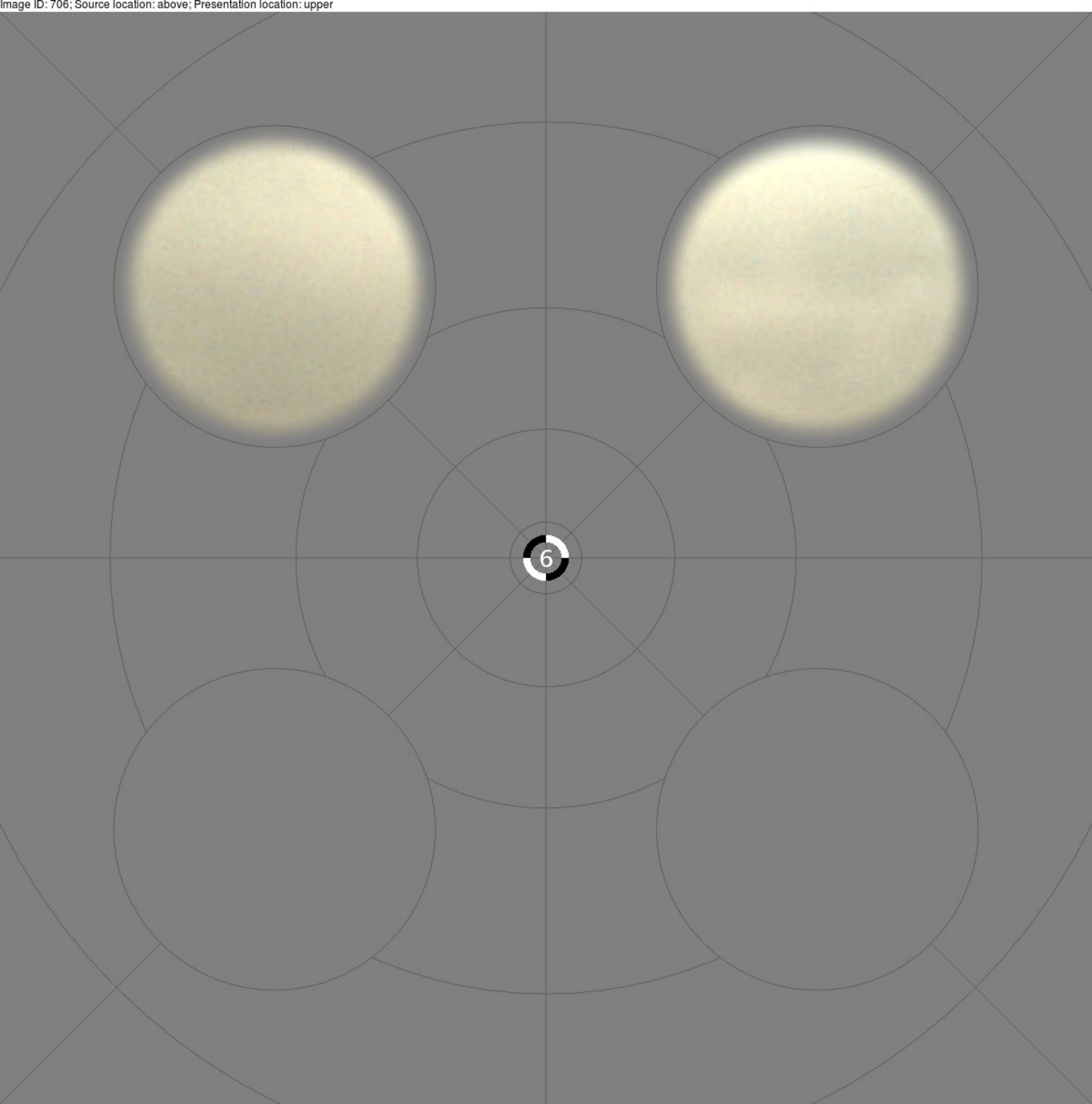

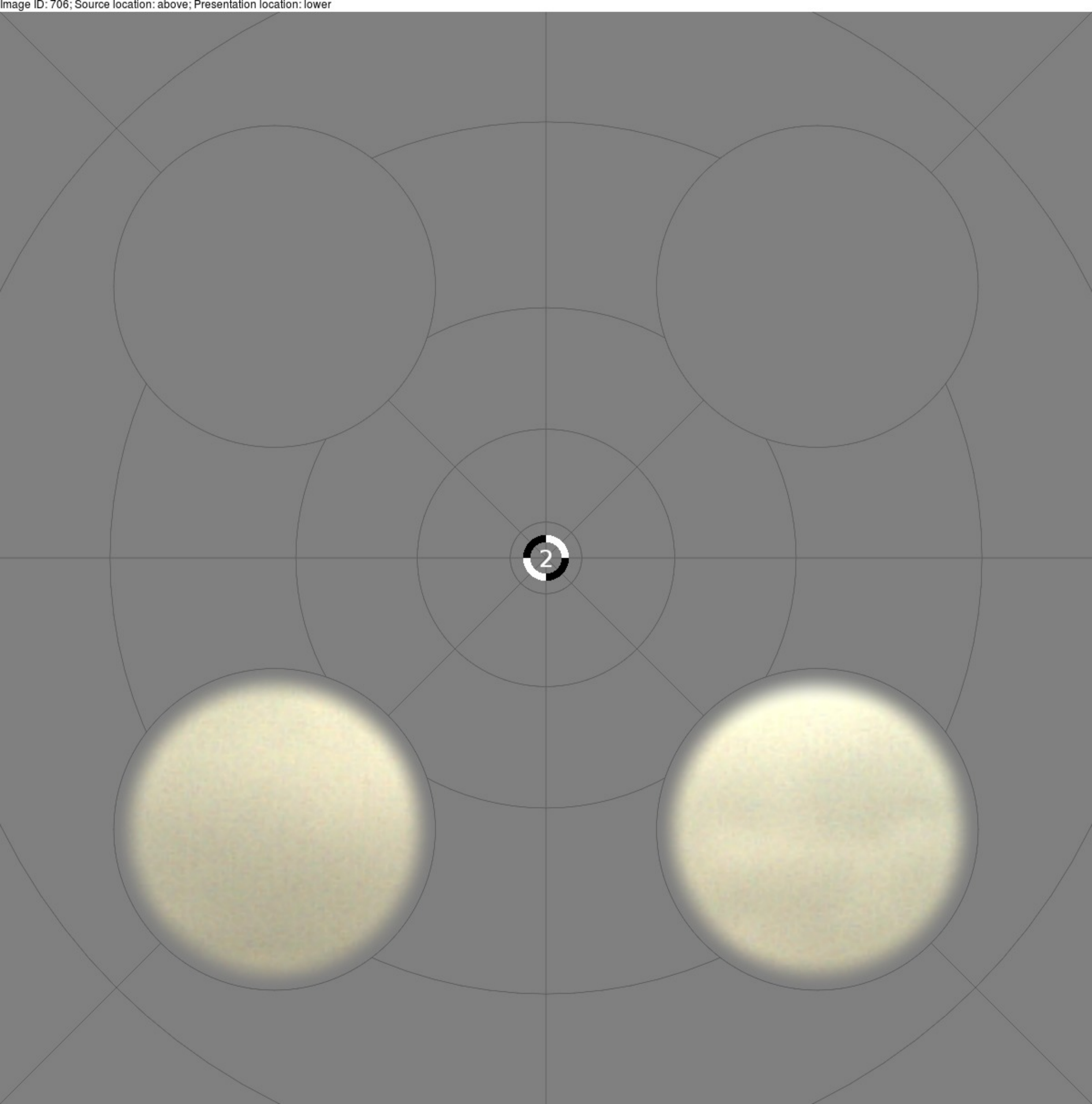

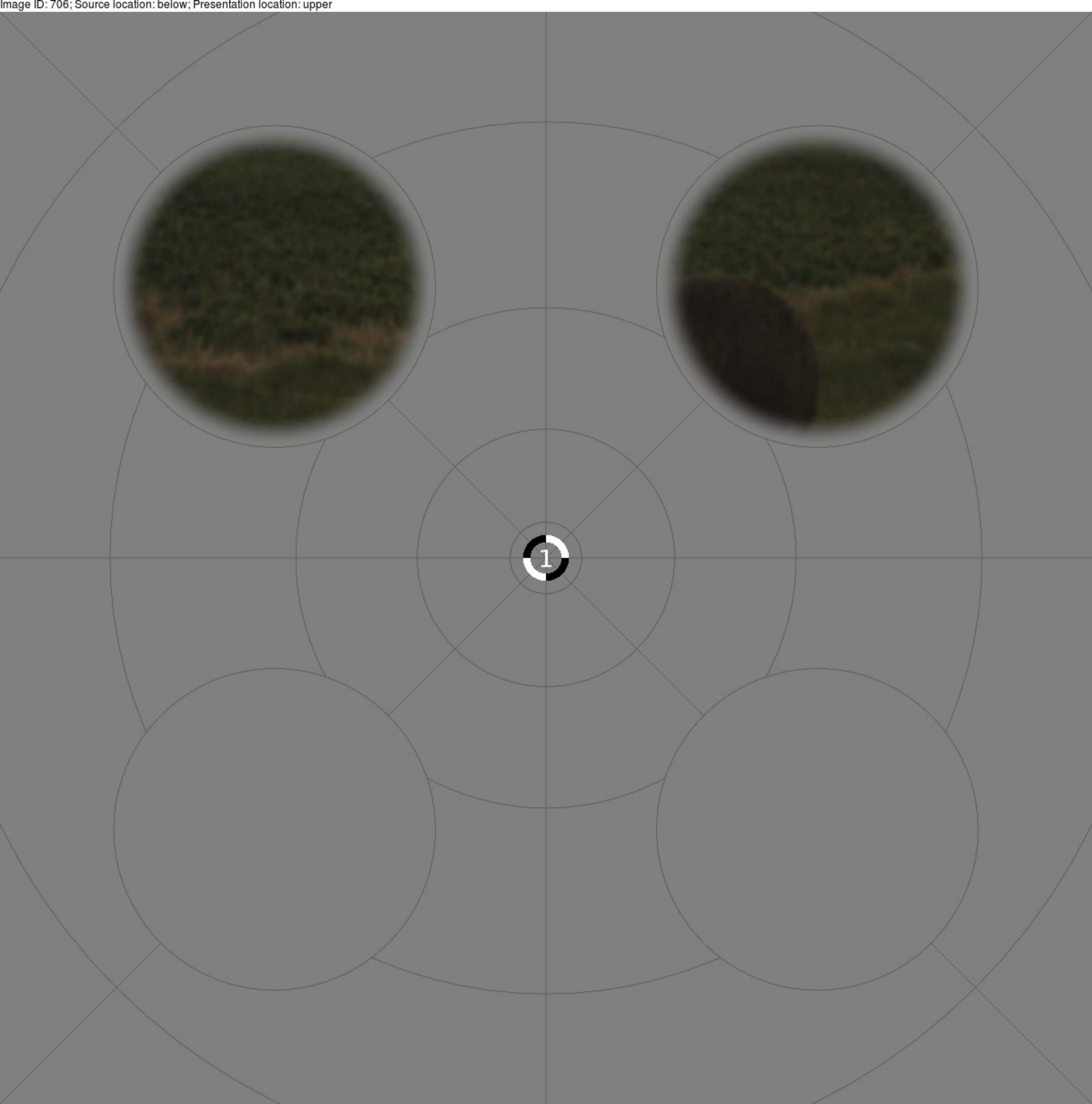

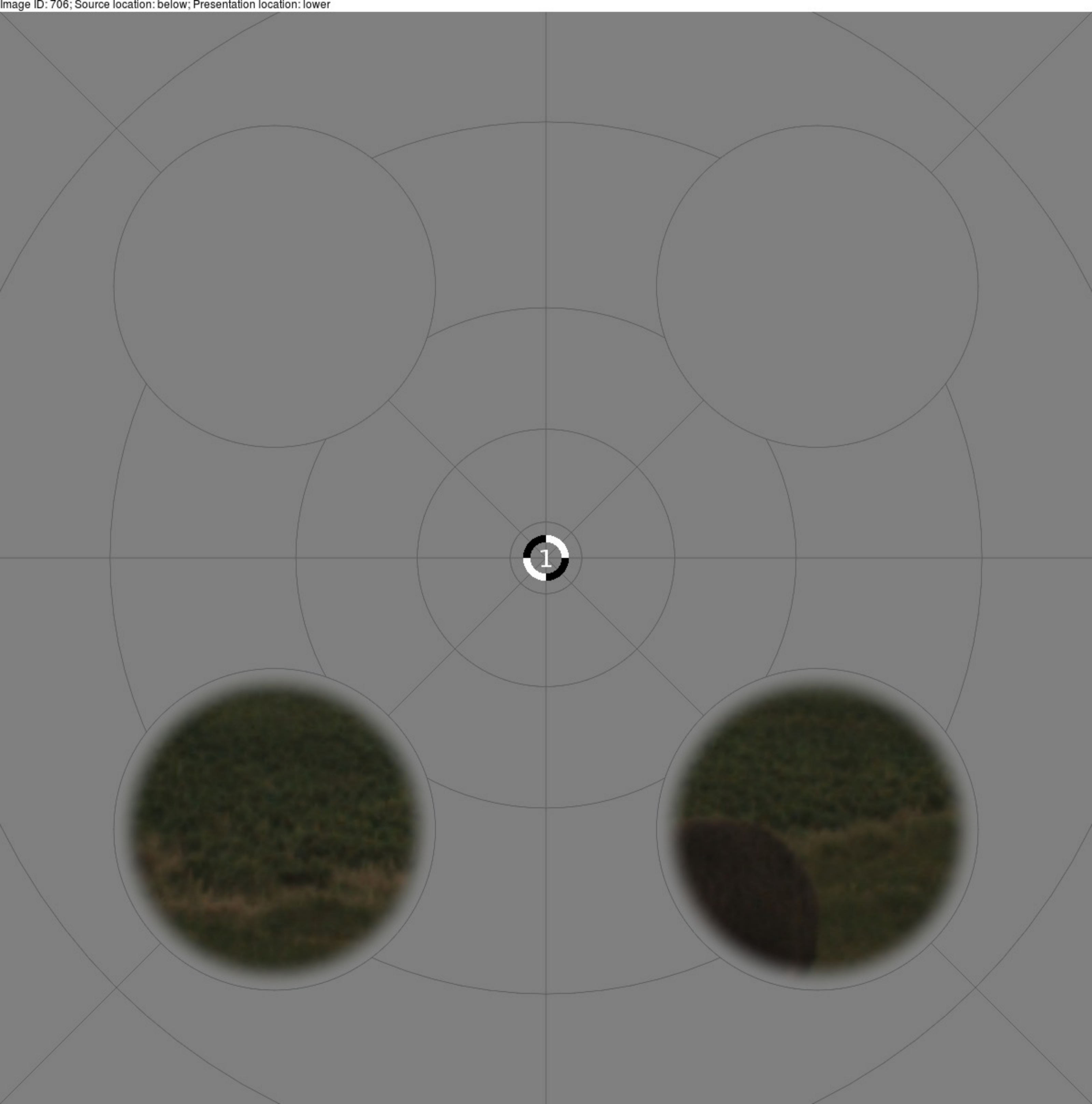

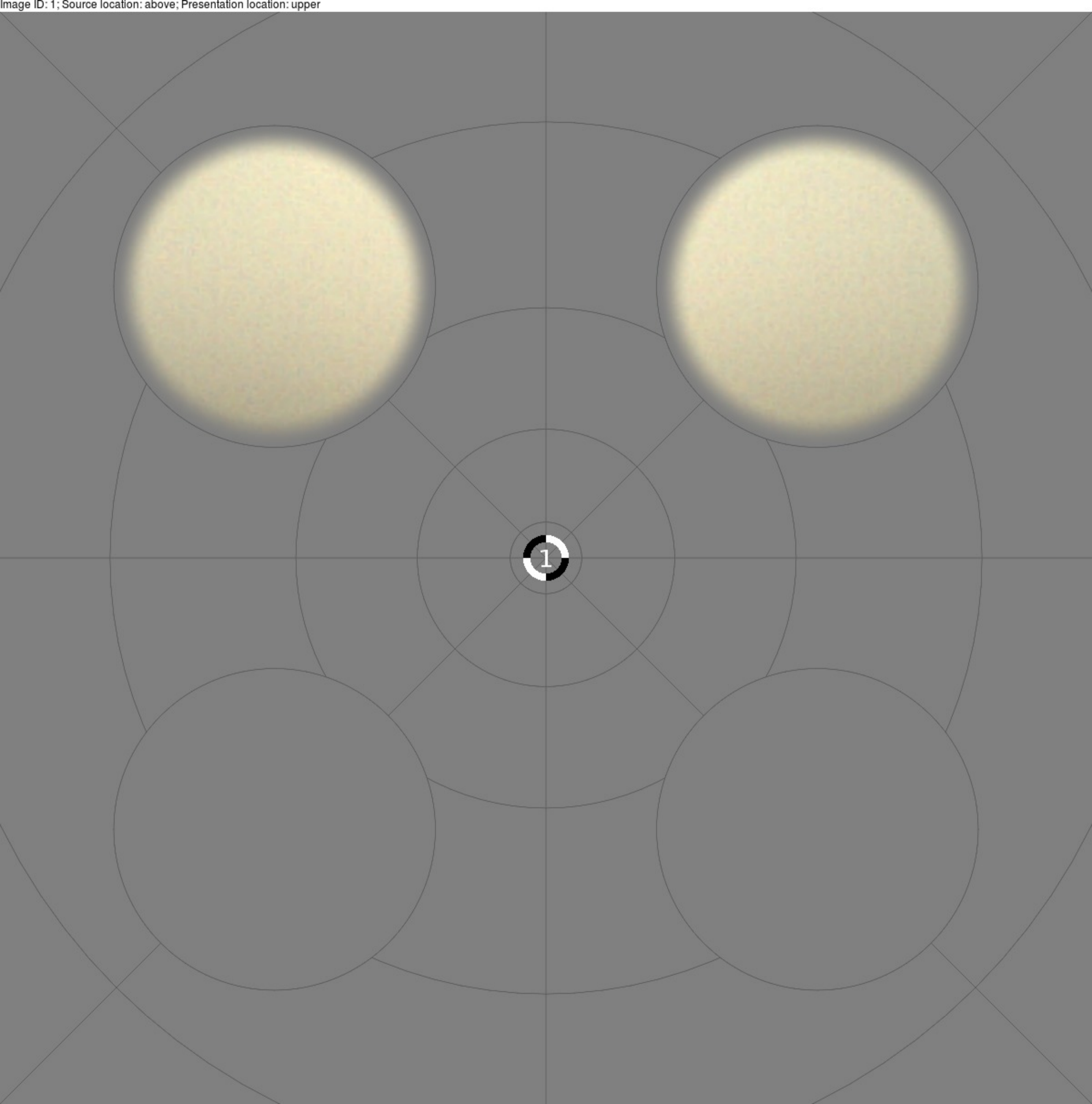

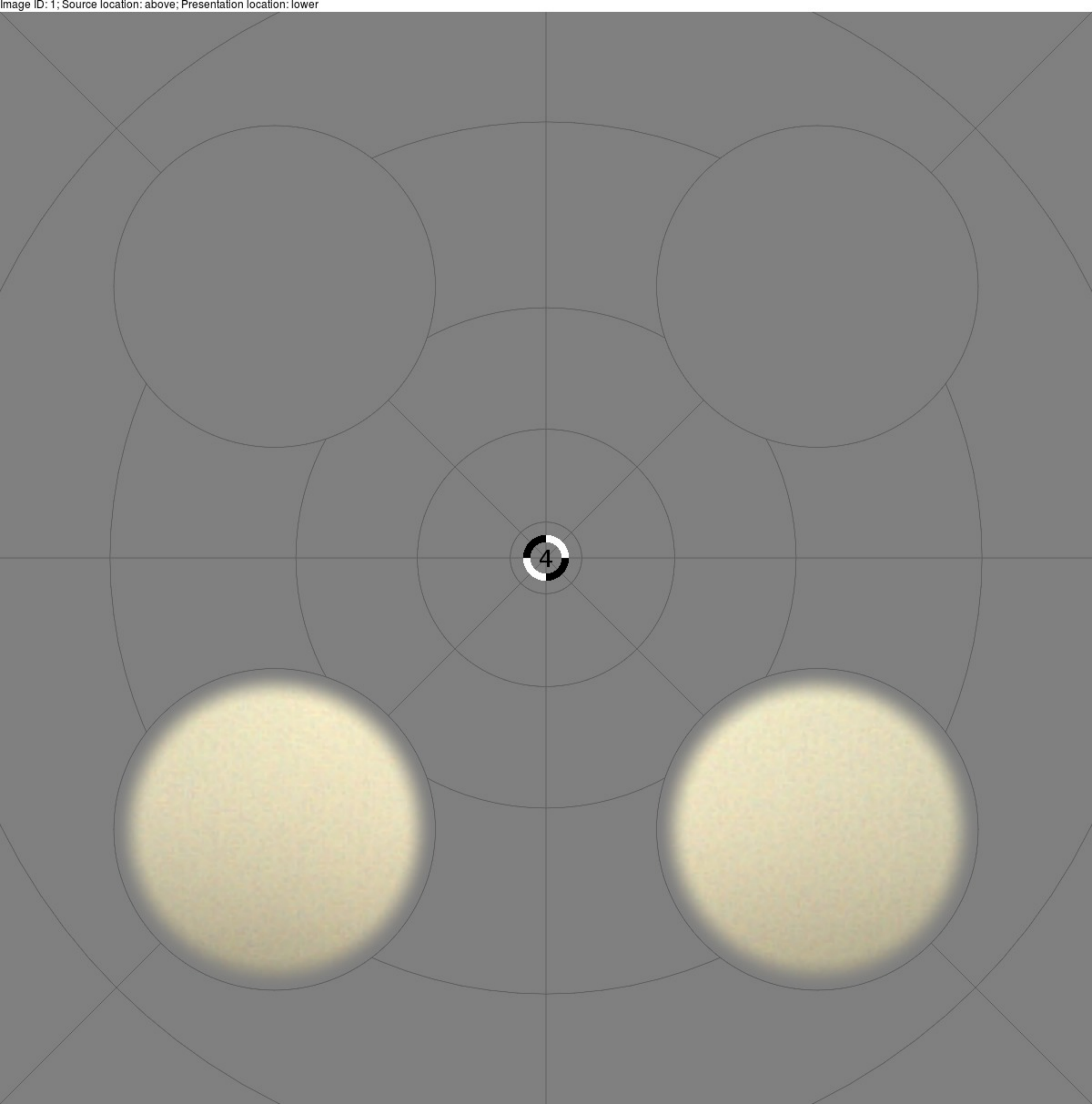

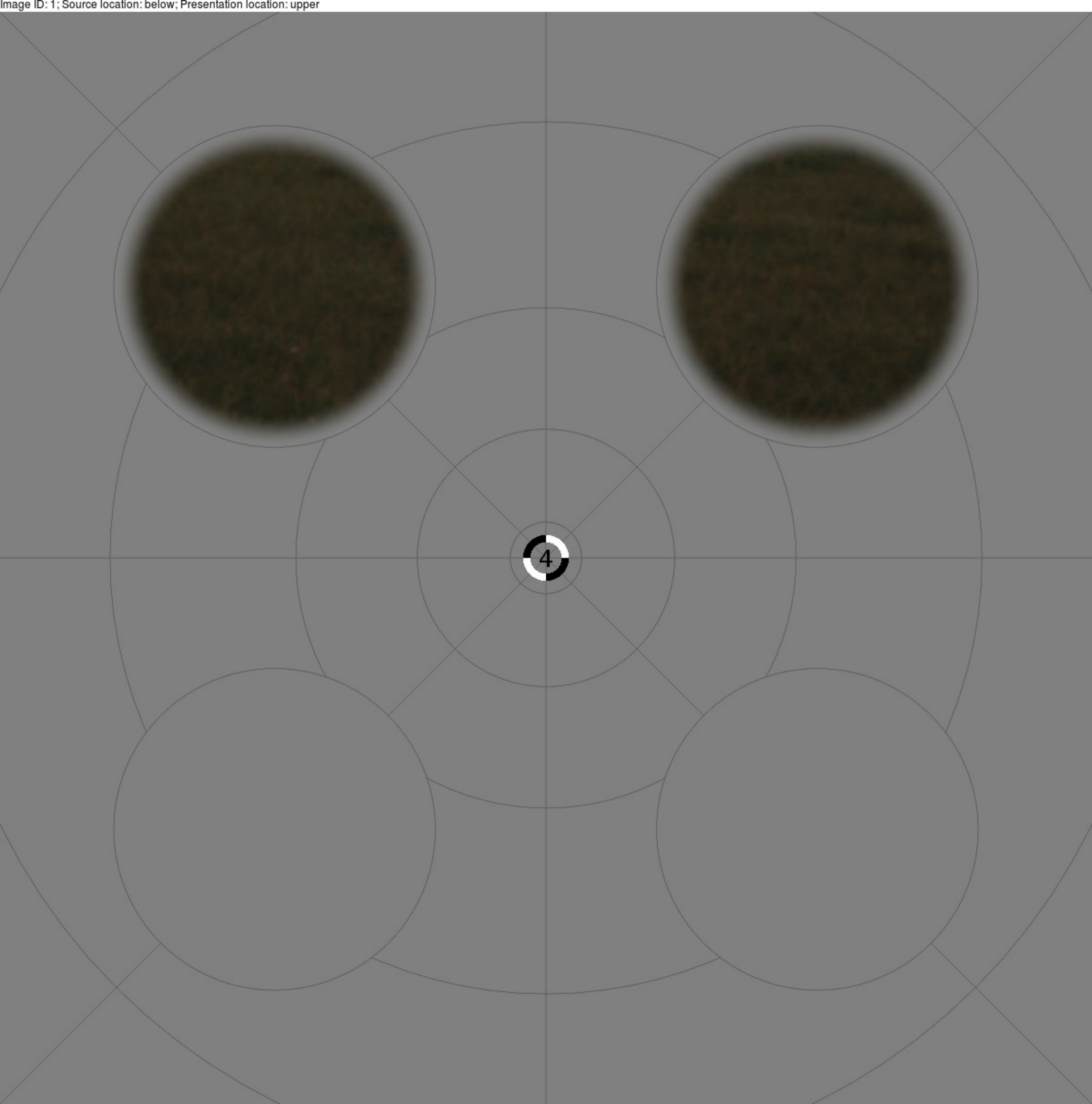

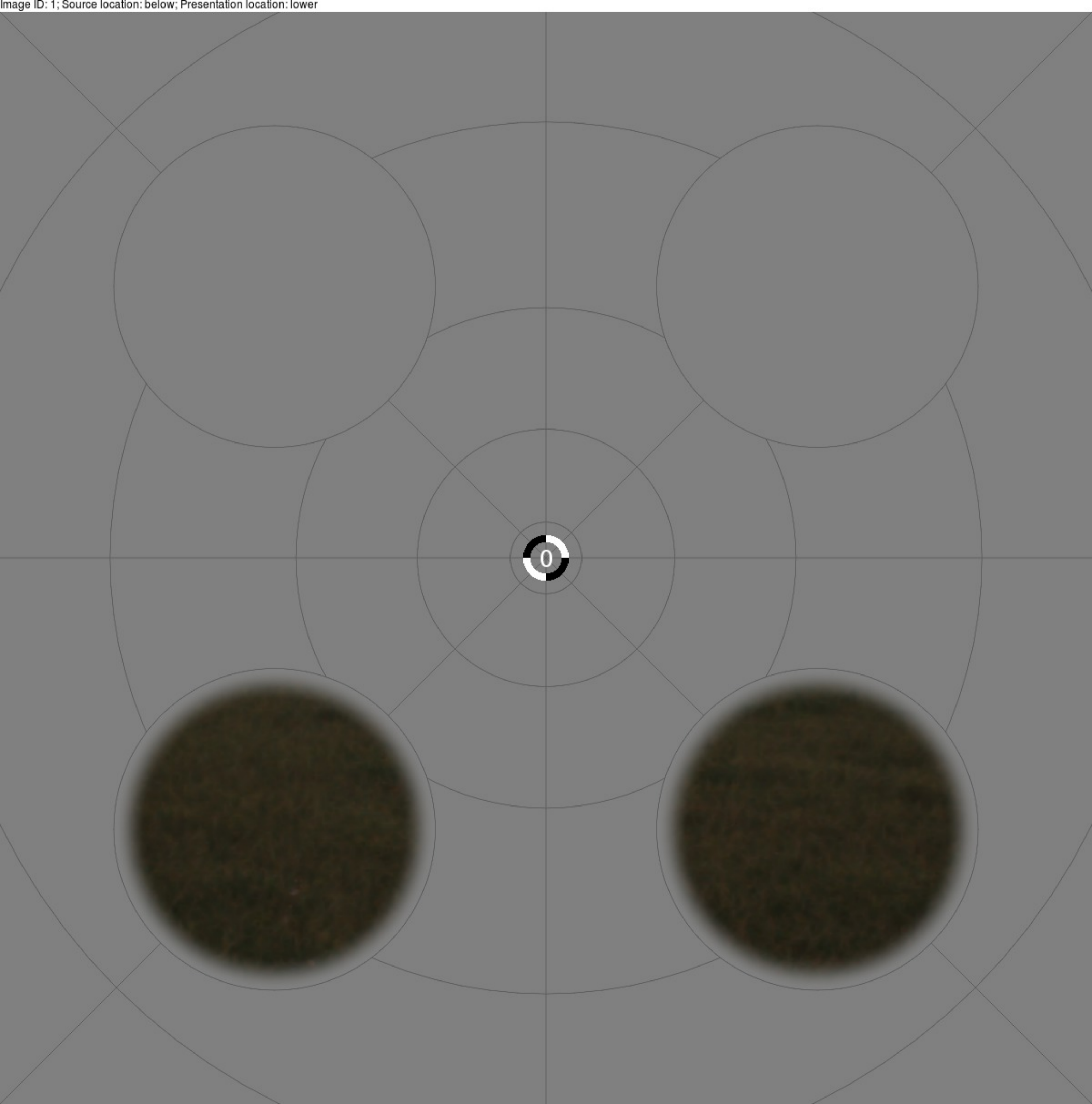

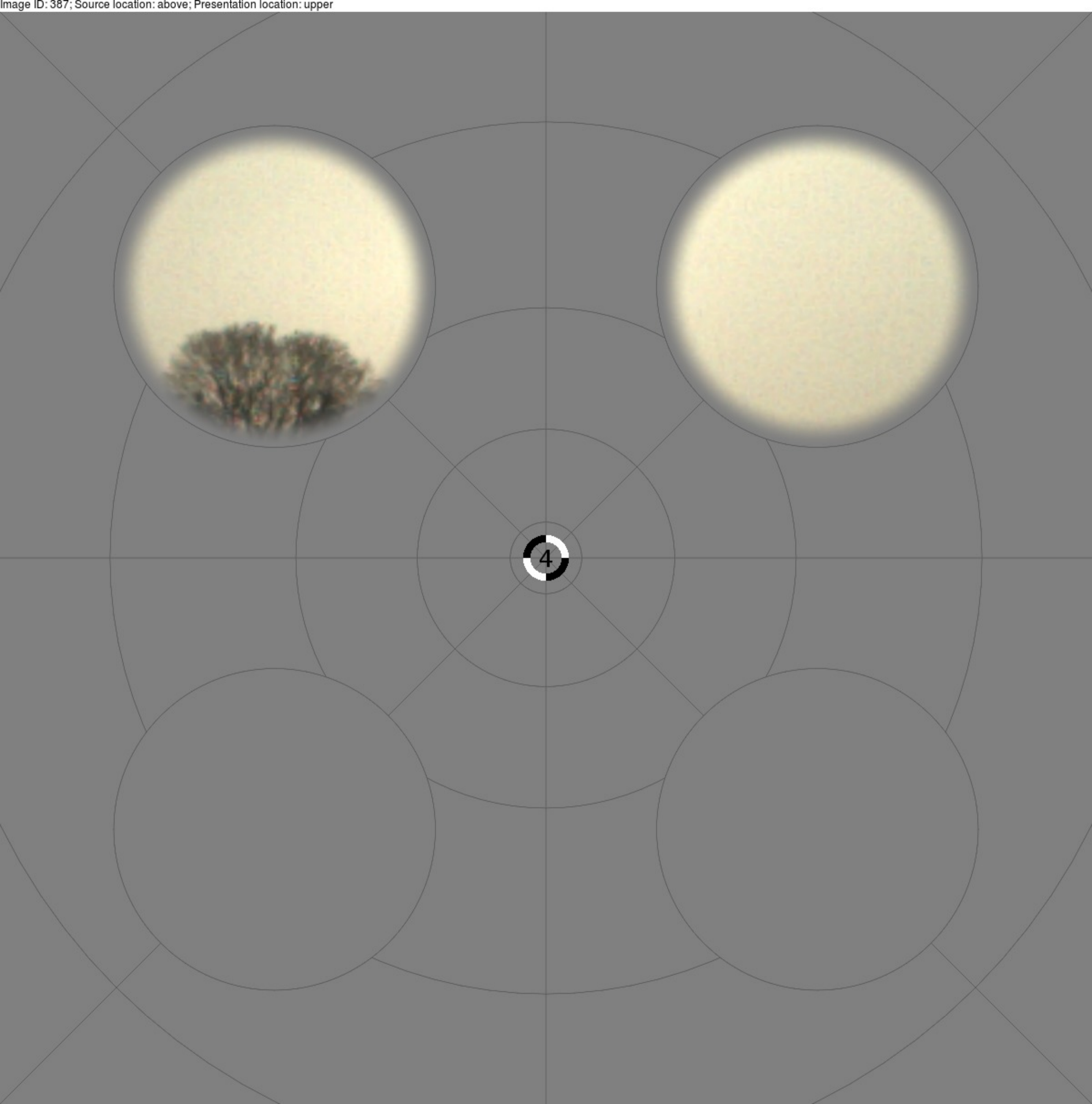

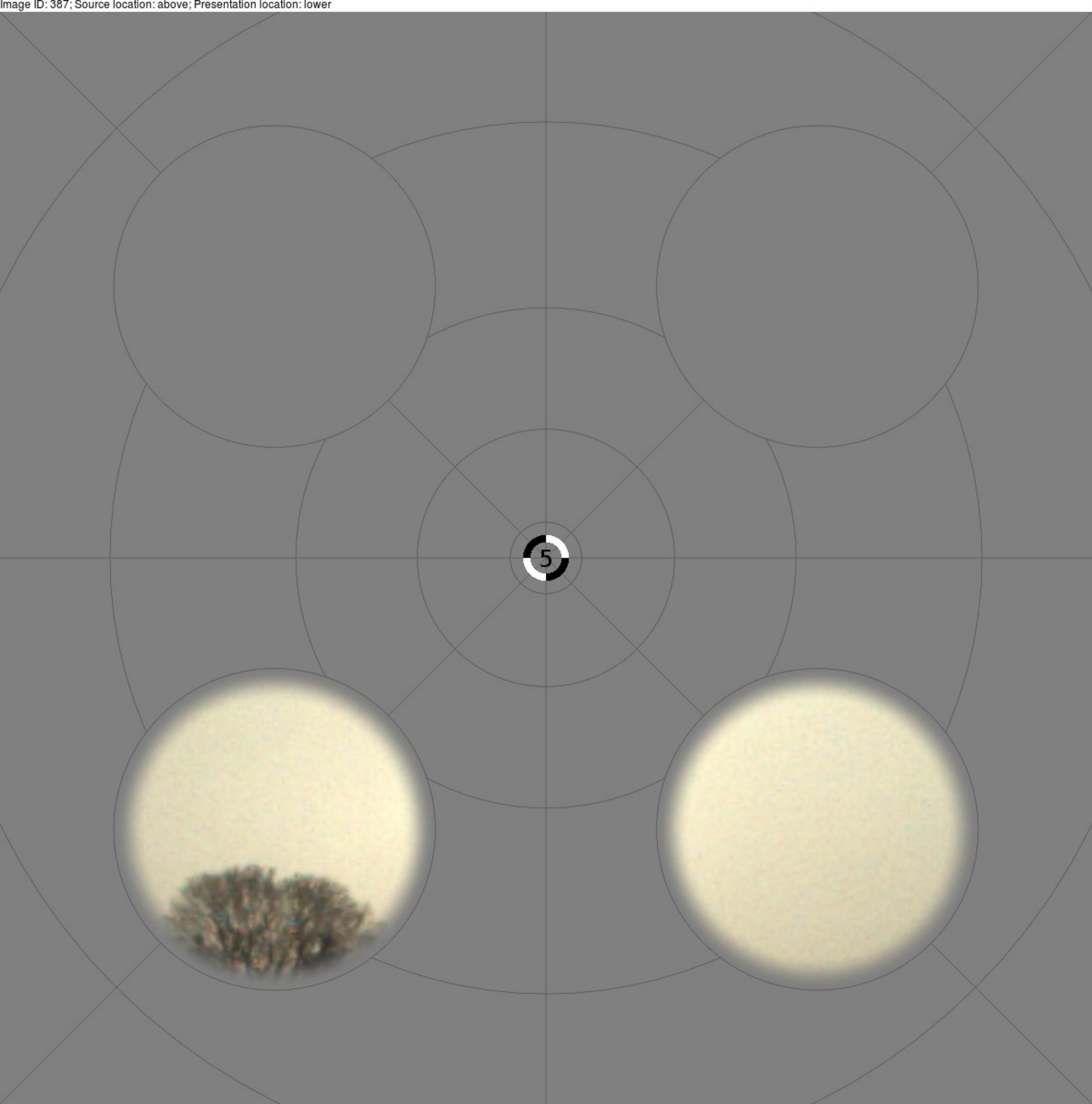

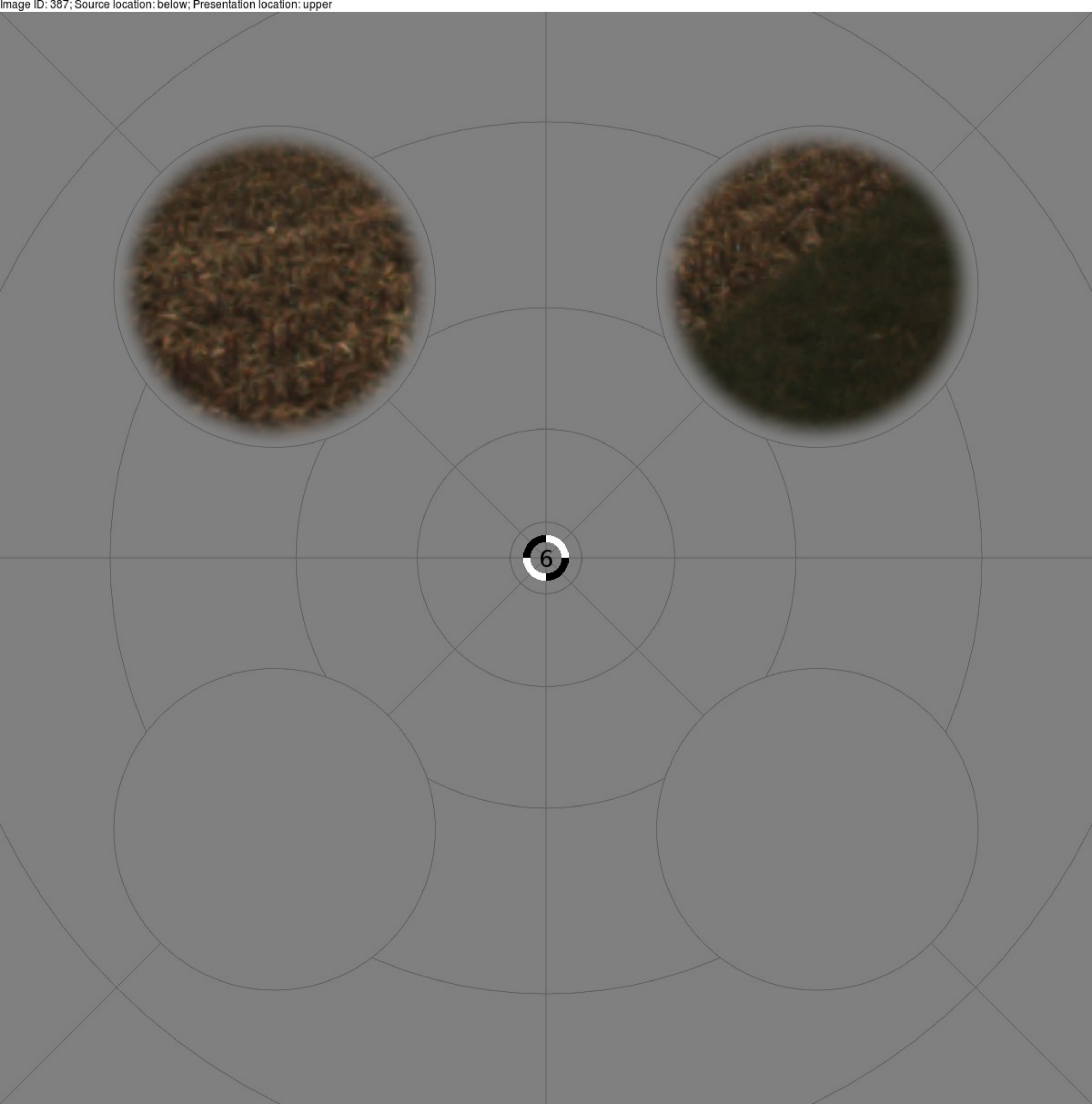

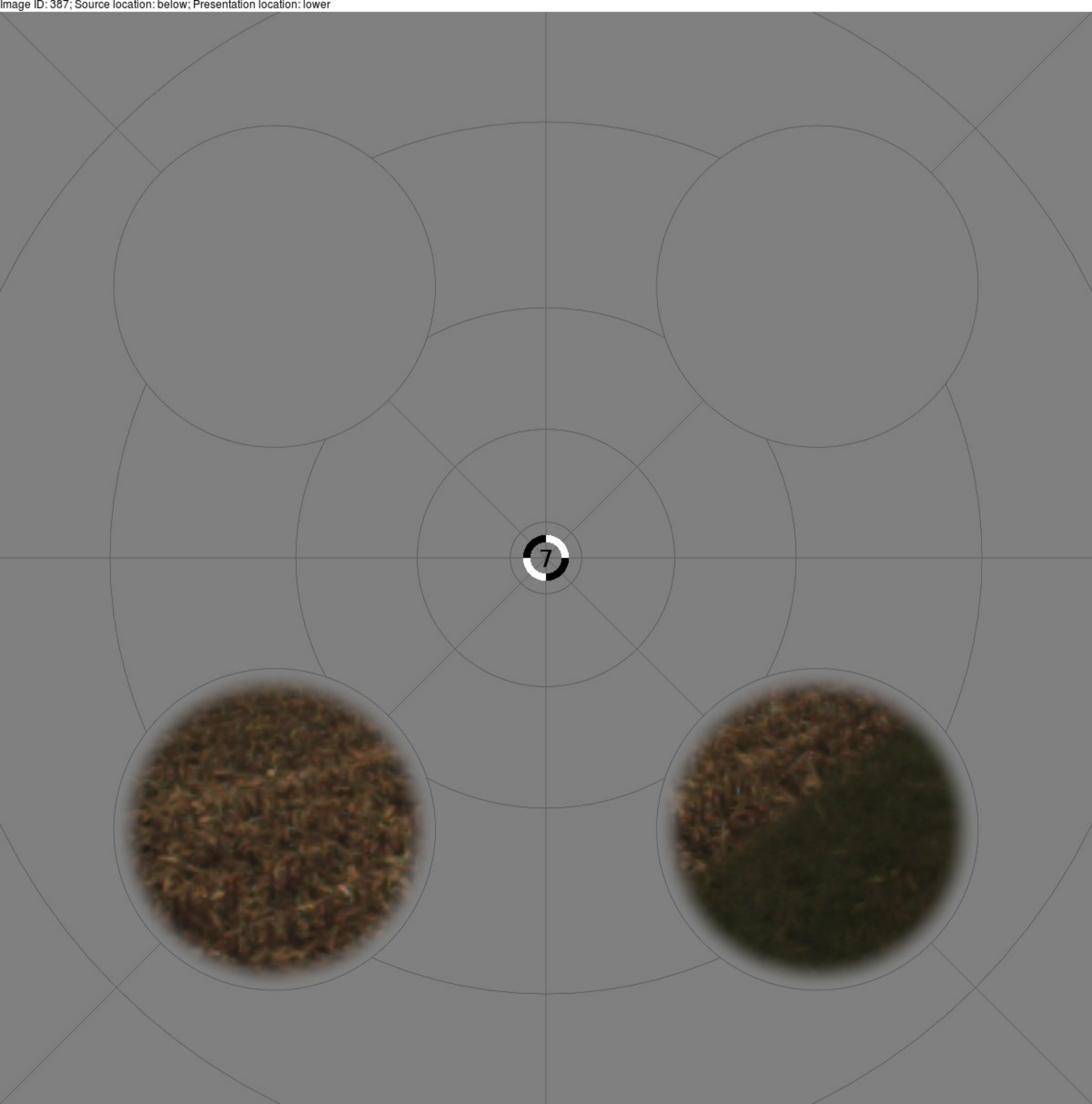

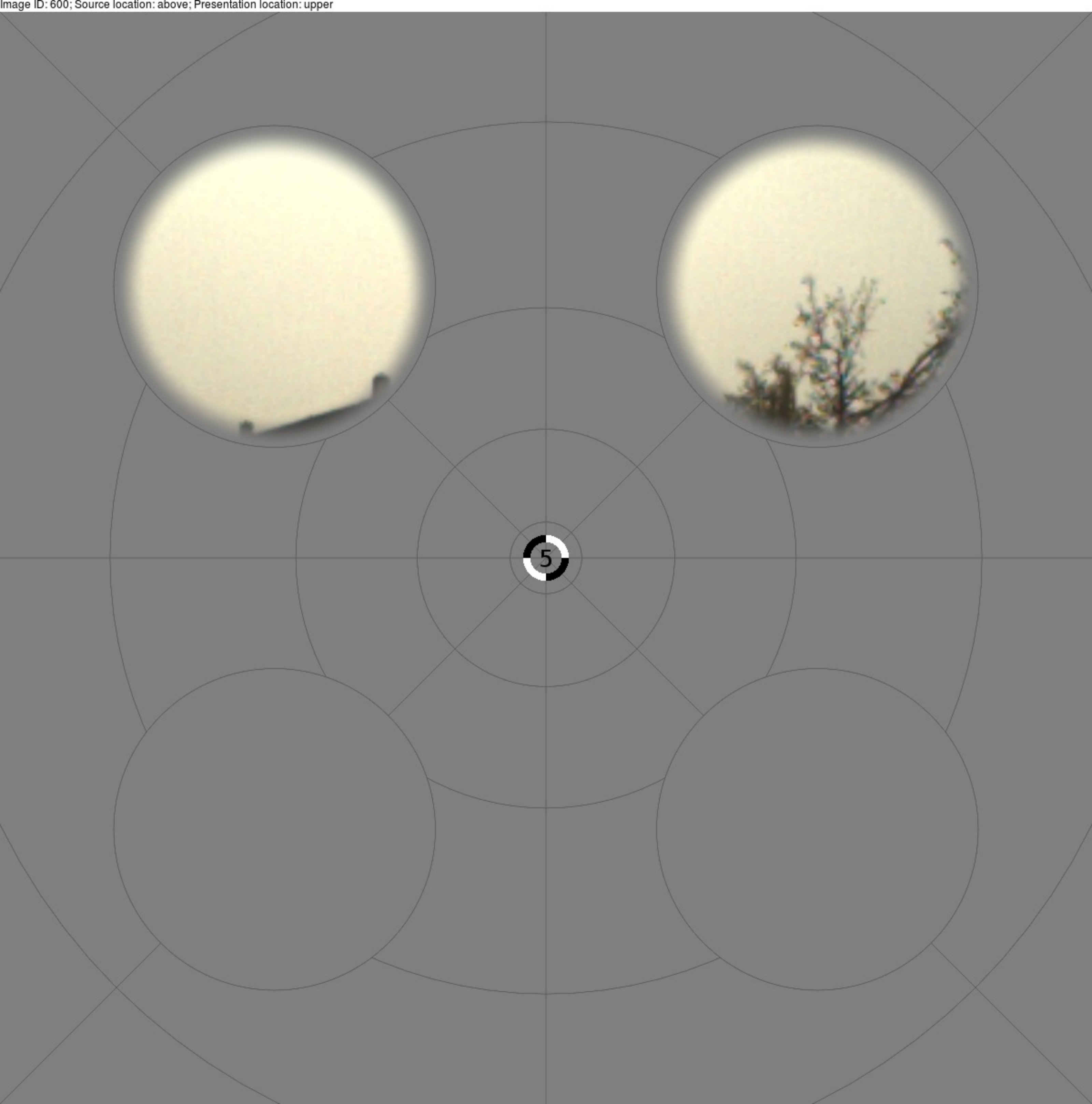

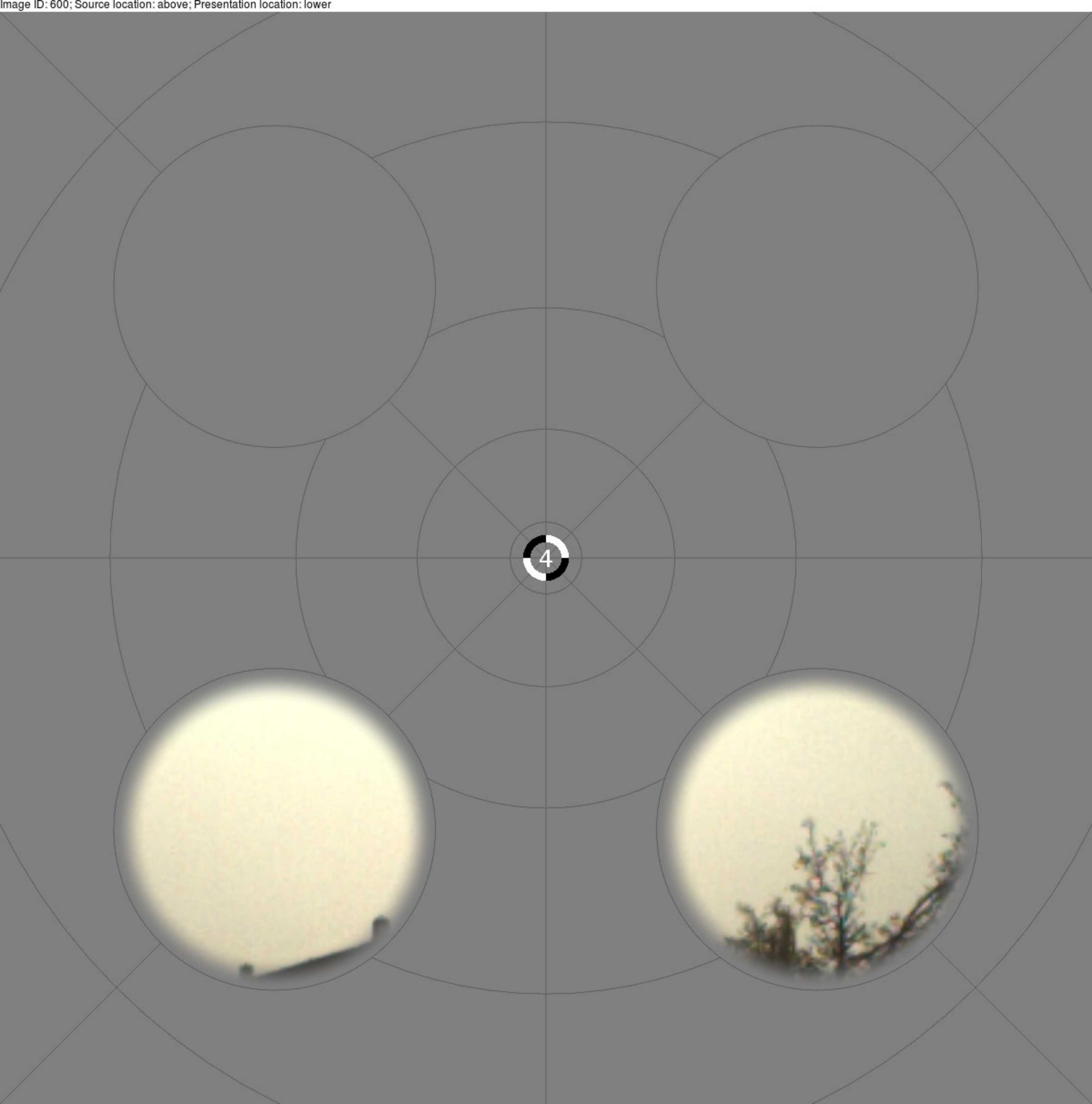

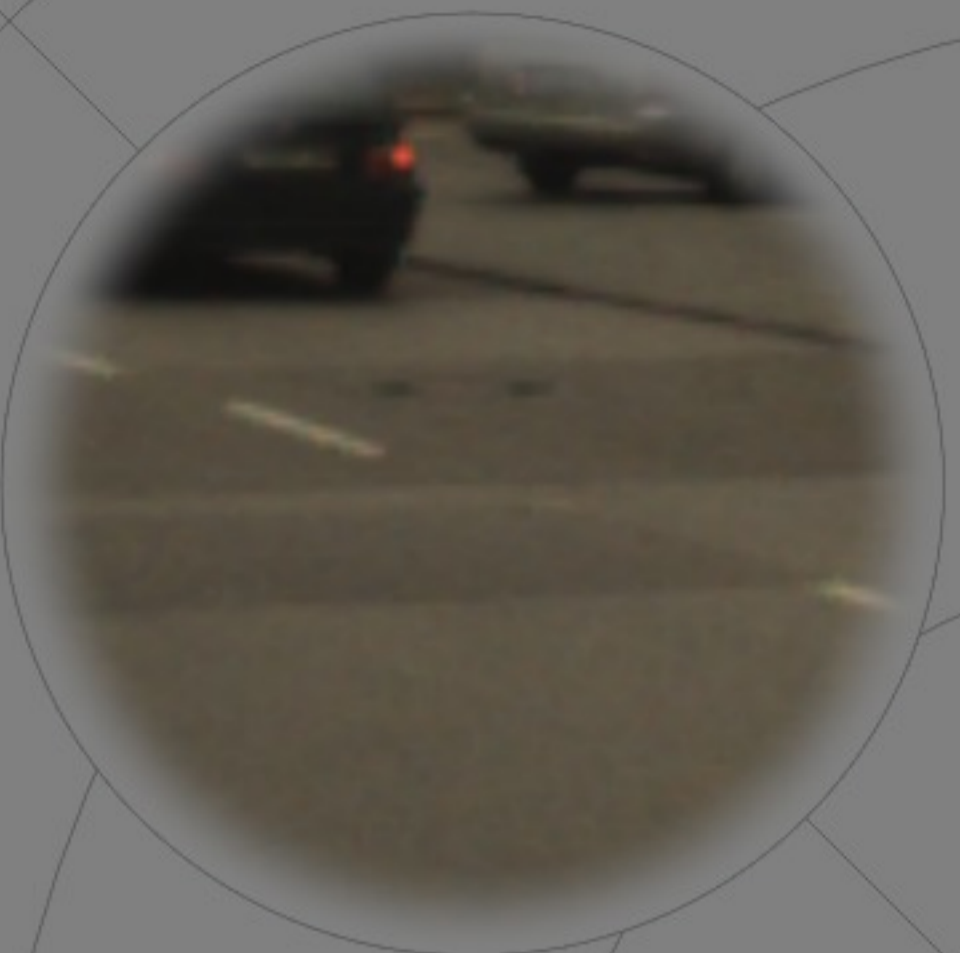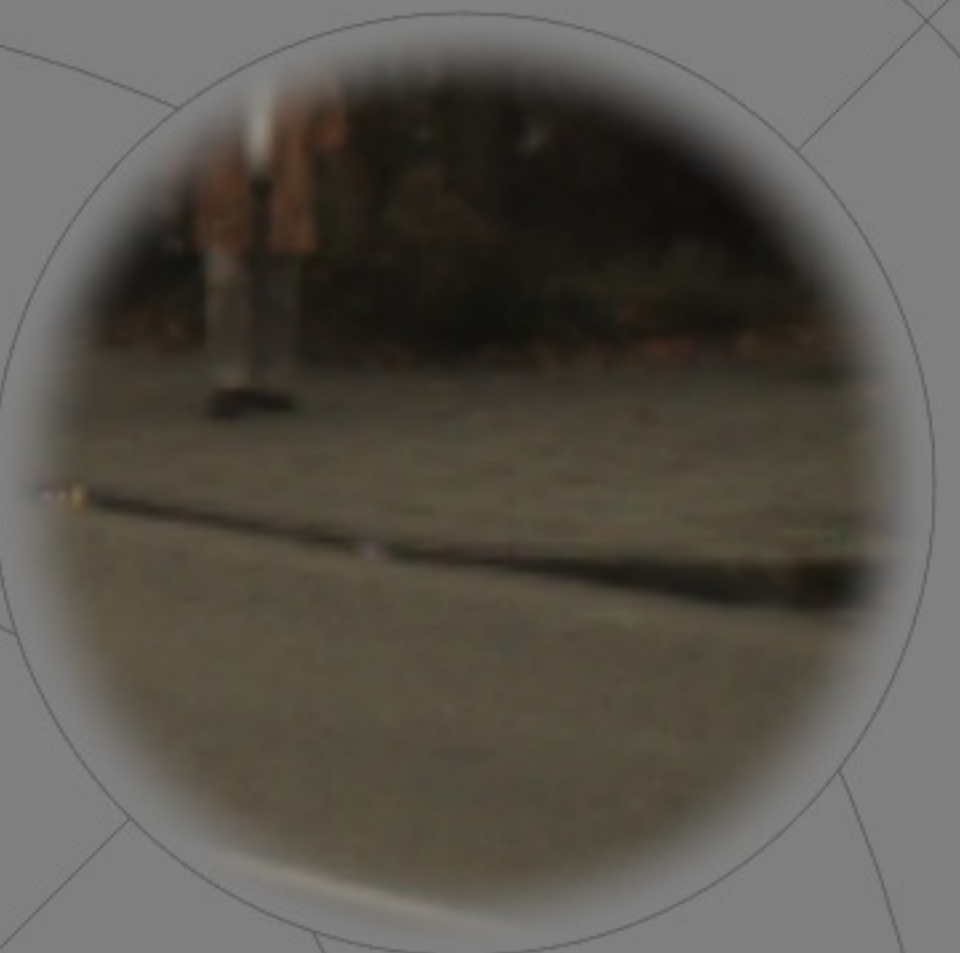

2

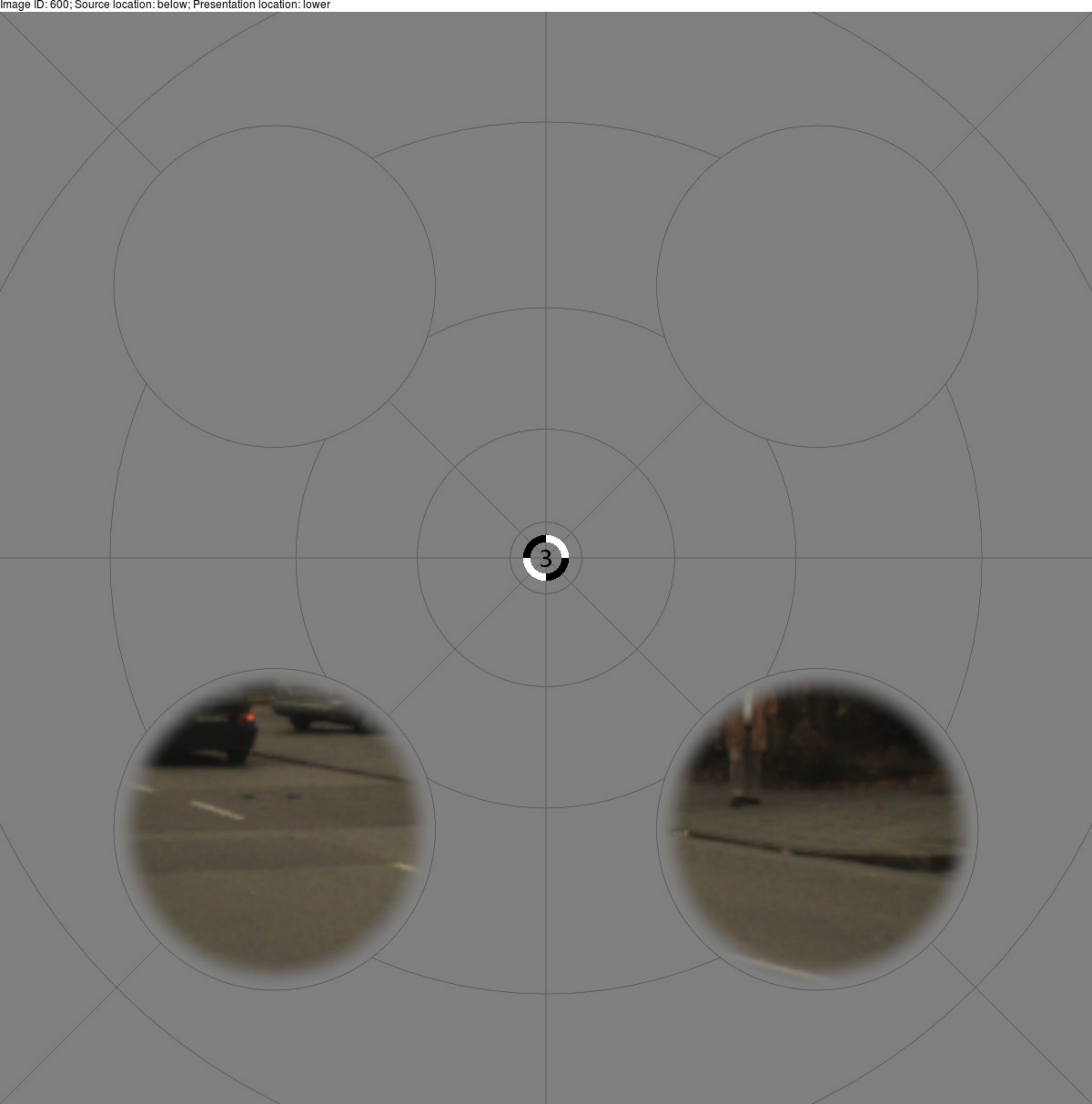

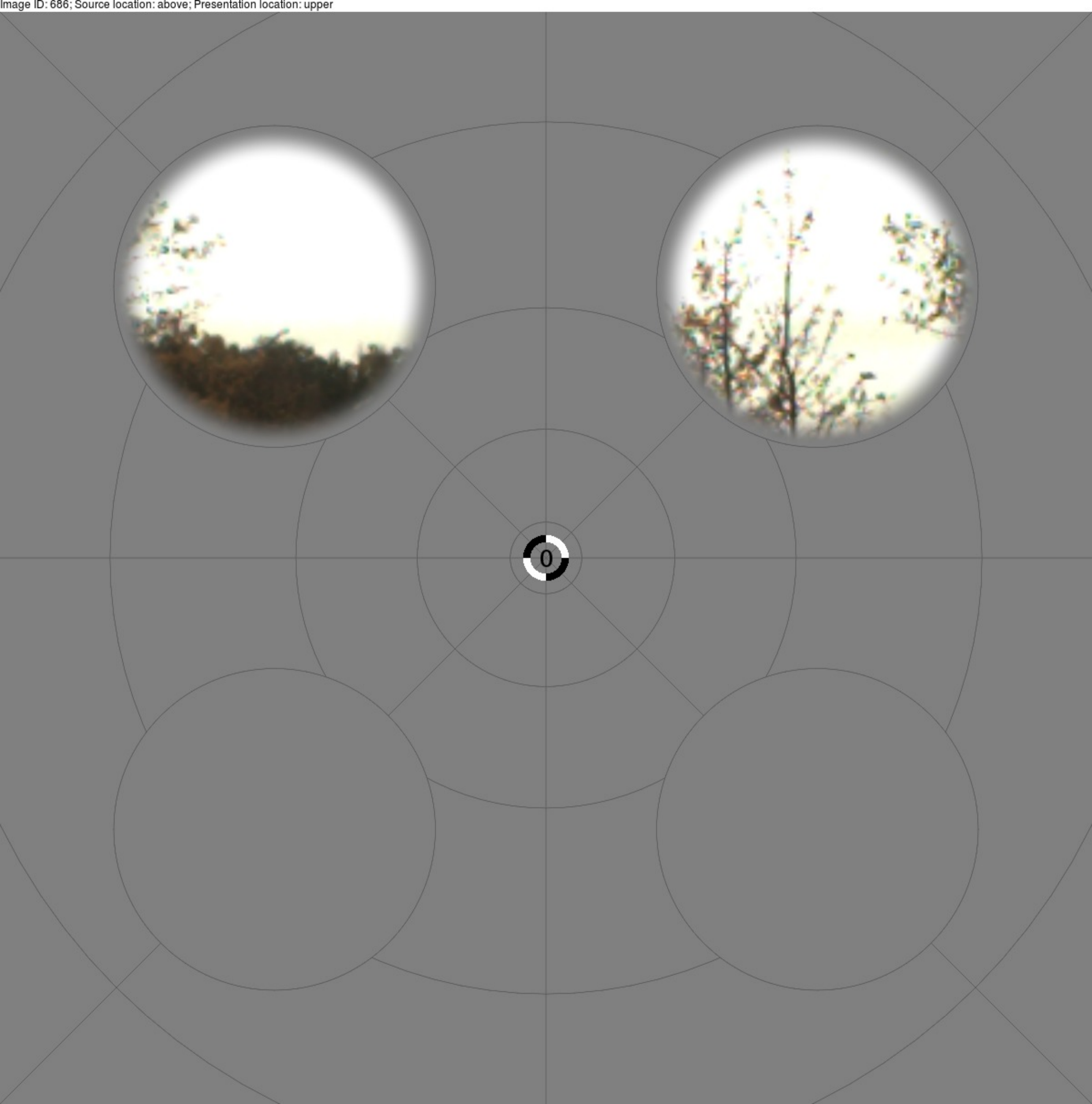

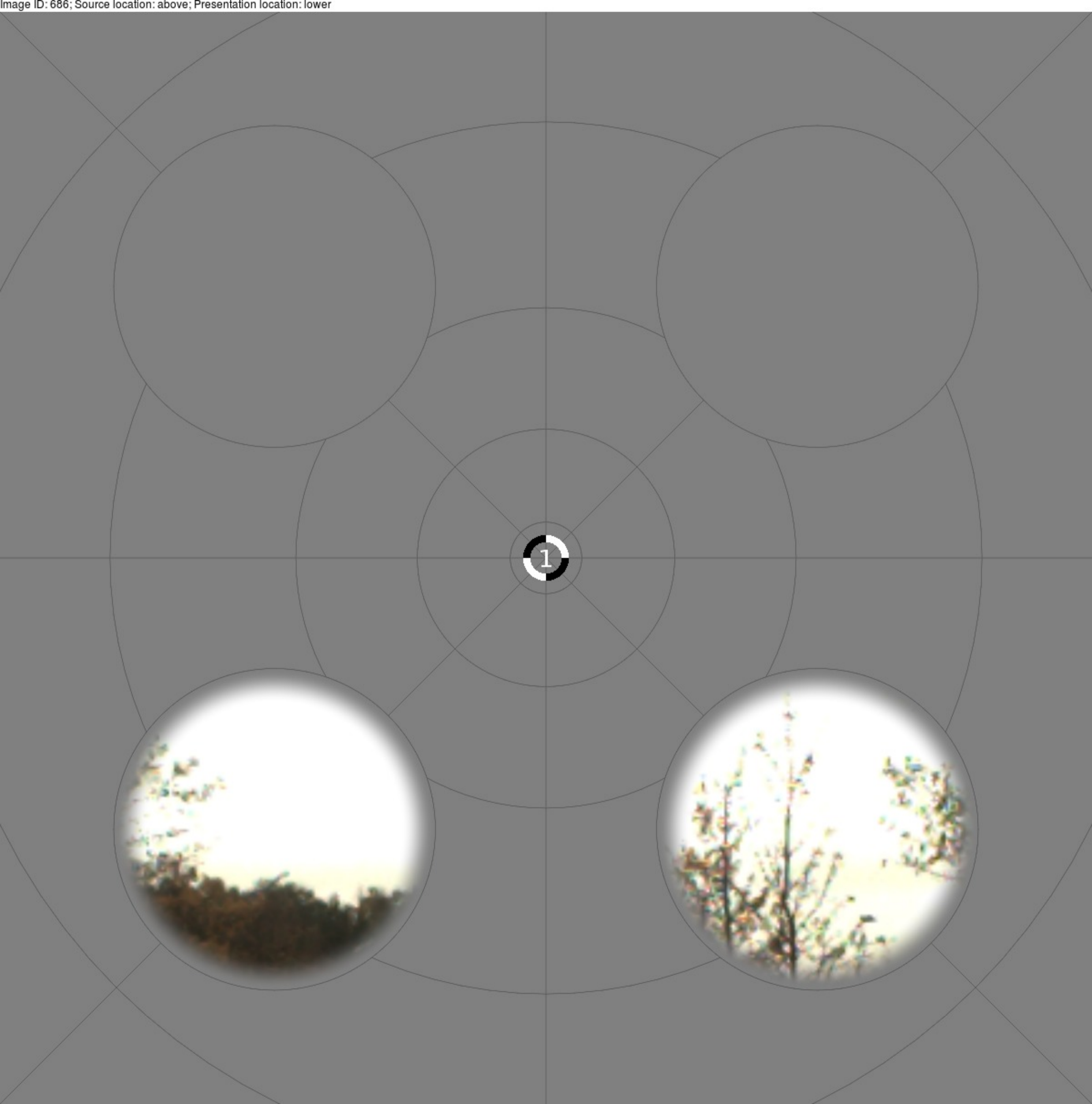

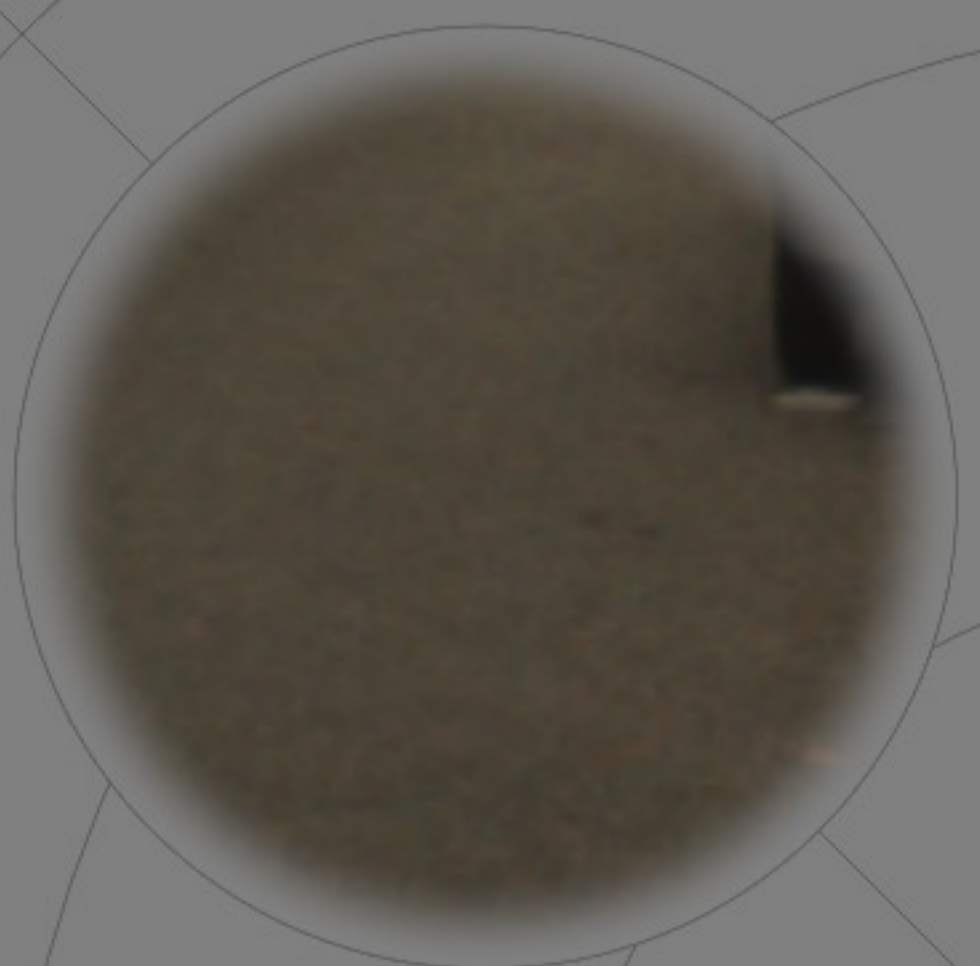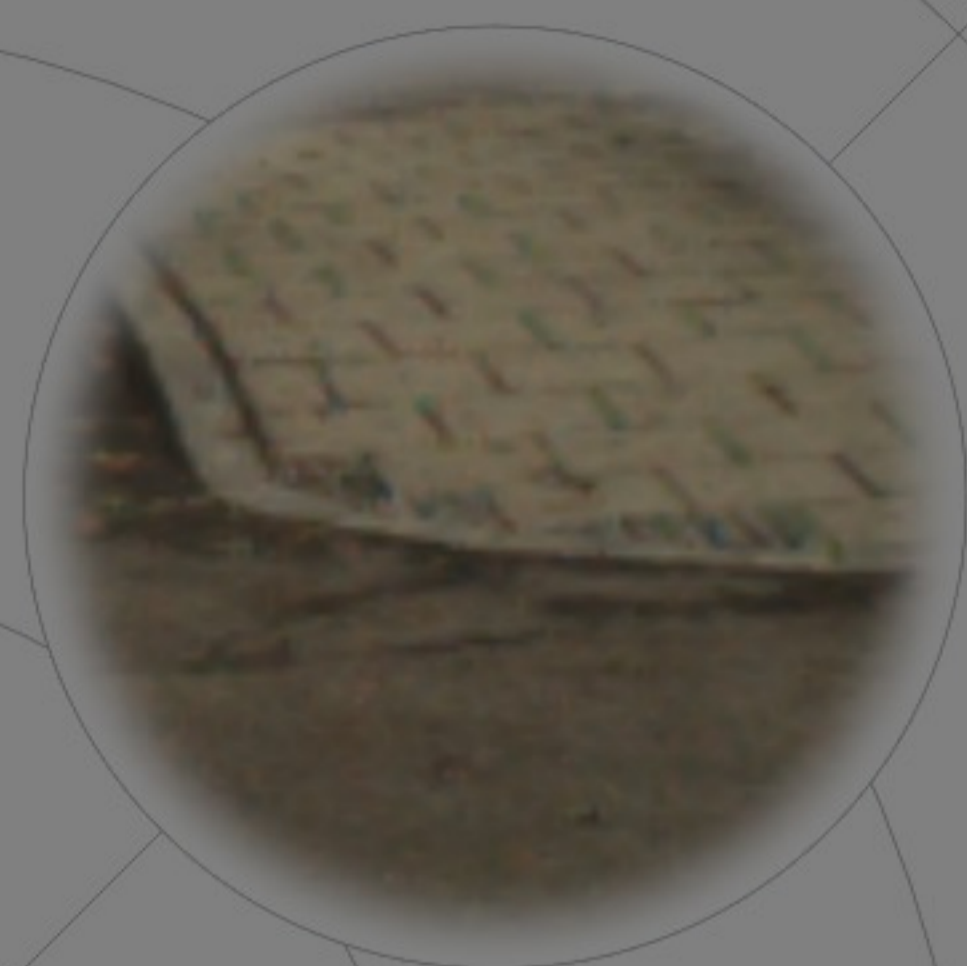

2

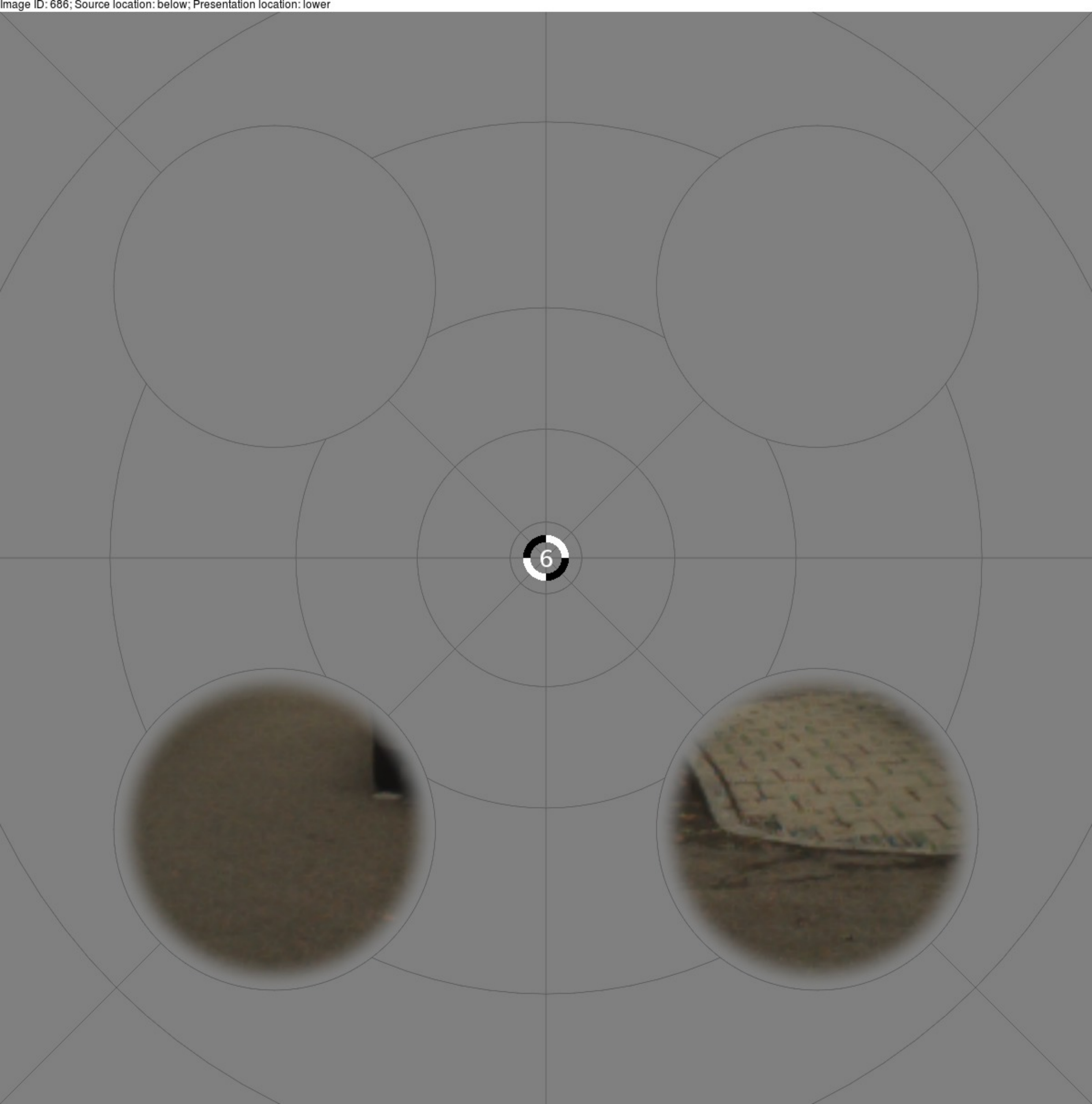

Supplement: Supplemental Information 1 [file peerj-03-1038-s001.pdf]

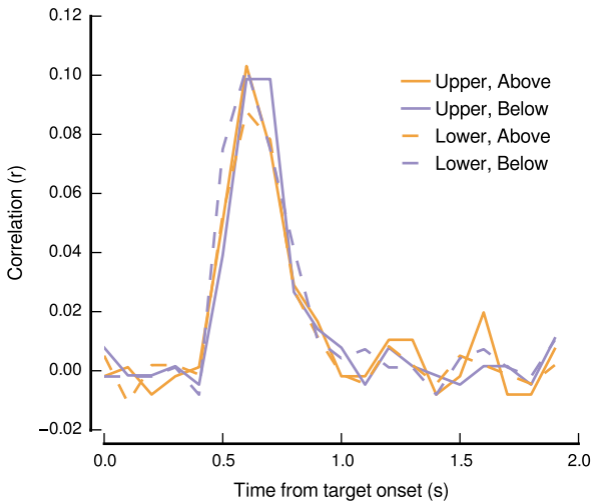

Supplement: Supplemental Information 3 — Performance was quantified as the correlation between target presence and participant response (vertical axis) at different time lags (horizontal axis), for target onsets occurring near to a particular presentation (upper, lower) and source (above, below) location condition. [file peerj-03-1038-s003.pdf]

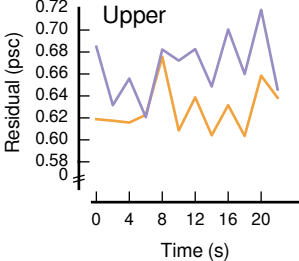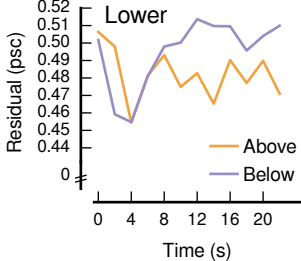

Supplement: Supplemental Information 4 — The horizontal axis shows the time after the trial onset, in seconds. The vertical axis shows the residual error, which was formed from the squared raw residuals in percent signal change units and averaged across participants and visual areas. Panels show the upper and lower presentation conditions, respectively. Note that the vertical scale differs across the panels; the residual error was larger for the upper presentation than the lower. [file peerj-03-1038-s004.pdf]
