# Supplementary material for: Sensitivity to the visual field origin of natural image patches in human low-level visual cortex
Source: PeerJ. 2015 Jun 23;3:e1038. doi: 10.7717/peerj.1038 (PMC4485252; doi:10.7717/peerj.1038)
Supplement: Supplemental Information 2 [file peerj-03-1038-s002.pdf]

Supplementary Table 1. Complete ANOVA output for the three-way design (visual area × presentation location × source location).

Effect	Statistic	p
Visual area	$F_{1.60,9.57} = 0.356$	0.669
Presentation location	$F_{1,6} = 4.45$	0.079
Source location	$F_{1,6} = 4.95$	0.068
Visual area × presentation location	$F_{2,12} = 0.74$	0.499
Visual area × source location	$F_{2,12} = 11.04$	0.002
Presentation location × source location	$F_{1,6} = 11.19$	0.016
Visual area × presentation location × source location	$F_{1.9,11.4} = 1.52$	0.260
